# Genome analyses of the sunflower pathogen *Plasmopara halstedii* provide insights into effector evolution in downy mildews and *Phytophthora*

**DOI:** 10.1186/s12864-015-1904-7

**Published:** 2015-10-05

**Authors:** Rahul Sharma, Xiaojuan Xia, Liliana M. Cano, Edouard Evangelisti, Eric Kemen, Howard Judelson, Stan Oome, Christine Sambles, D. Johan van den Hoogen, Miloslav Kitner, Joël Klein, Harold J. G. Meijer, Otmar Spring, Joe Win, Reinhard Zipper, Helge B. Bode, Francine Govers, Sophien Kamoun, Sebastian Schornack, David J. Studholme, Guido Van den Ackerveken, Marco Thines

**Affiliations:** Biodiversity and Climate Research Centre (BiK-F), Georg-Voigt-Str. 14-16, 60325 Frankfurt (Main), Germany; Institute of Ecology, Evolution and Diversity, Goethe University, Max-von-Laue-Str. 9, 60323 Frankfurt (Main), Germany; Senckenberg Gesellschaft für Naturforschung, Senckenberganlage 25, 60325 Frankfurt (Main), Germany; Center for Integrative Fungal Research (IPF), Georg-Voigt-Str. 14-16, 60325 Frankfurt (Main), Germany; The Sainsbury Laboratory, Norwich Research Park, Norwich, NR4 7UH UK; Sainsbury Laboratory, University of Cambridge, Cambridge, CB2 1LR UK; Max Planck Institute for Plant Breeding Research, Carl von Linne´ Weg 10, Cologne, 50829 Germany; Department of Plant Pathology and Microbiology, University of California, Riverside, CA 92521 USA; Plant-Microbe Interactions, Department of Biology, Utrecht University, Padualaan 8, NL-3584 CH Utrecht, The Netherlands; Biosciences, University of Exeter, Stocker Road, Exeter, EX4 4QD UK; Laboratory of Phytopathology, Wageningen University, Droevendaalsesteeg 1, NL-6708PB Wageningen, The Netherlands; Department of Botany, Faculty of Science, Palacký University Olomouc, Šlechtitelů 11, 78371 Olomouc, Czech Republic; University of Hohenheim, Institute of Botany 210, D-70593 Stuttgart, Germany; Merck-Stiftungsprofessur für Molekulare Biotechnologie, Fachbereich Biowissenschaften and Buchmann Institute for Molecular Life Sciences (BMLS), Goethe Universität Frankfurt, Max-von-Laue-Str. 9, 60438 Frankfurt am Main, Germany; Present address: Department of Plant Pathology, North Carolina State University Raleigh, Raleigh, NC 27695 USA; Integrative Fungal Research (IPF), Biodiversity and Climate Research Centre (BiK-F), Senckenberganlage 25, D-60325 Frankfurt am Main, Germany

**Keywords:** Comparative genomics, Core effectors, Downy mildew, Evolution, Microsatellites, Obligate biotroph, Oomycetes, Phytohormones, Plant pathogen, Promoters, RxLR effectors

## Abstract

**Background:**

Downy mildews are the most speciose group of oomycetes and affect crops of great economic importance. So far, there is only a single deeply-sequenced downy mildew genome available, from *Hyaloperonospora arabidopsidis*. Further genomic resources for downy mildews are required to study their evolution, including pathogenicity effector proteins, such as RxLR effectors. *Plasmopara halstedii* is a devastating pathogen of sunflower and a potential pathosystem model to study downy mildews, as several *Avr*-genes and *R*-genes have been predicted and unlike *Arabidopsis* downy mildew, large quantities of almost contamination-free material can be obtained easily.

**Results:**

Here a high-quality draft genome of *Plasmopara halstedii* is reported and analysed with respect to various aspects, including genome organisation, secondary metabolism, effector proteins and comparative genomics with other sequenced oomycetes. Interestingly, the present analyses revealed further variation of the RxLR motif, suggesting an important role of the conservation of the dEER-motif. Orthology analyses revealed the conservation of 28 RxLR-like core effectors among *Phytophthora* species. Only six putative RxLR-like effectors were shared by the two sequenced downy mildews, highlighting the fast and largely independent evolution of two of the three major downy mildew lineages. This is seemingly supported by phylogenomic results, in which downy mildews did not appear to be monophyletic.

**Conclusions:**

The genome resource will be useful for developing markers for monitoring the pathogen population and might provide the basis for new approaches to fight *Phytophthora* and downy mildew pathogens by targeting core pathogenicity effectors.

**Electronic supplementary material:**

The online version of this article (doi:10.1186/s12864-015-1904-7) contains supplementary material, which is available to authorized users.

## Background

Oomycetes include devastating pathogens of plants and animals that can be found in almost all ecosystems and show a variety of different lifestyles [1–3]. They often cause serious infections on their hosts and are responsible for huge economic losses [4]. Understanding the evolution of these pathogens and their virulence mechanisms is key to developing strategies towards the sustainable control of the diseases that they cause.

The oomycete *Plasmopara halstedii* is an obligate biotroph that causes the economically important downy mildew disease of sunflower [4]. The life cycle of *Pl. halstedii*, described in earlier studies [4], is typical for a downy mildew pathogen. Disease symptoms include stunting and chlorosis, alteration of the secondary metabolism of the infected plant, reduced biomass production, damping off, and reduced seed yield, leading to reduced oil yield [4, 5]. Considering the devastating effects on the sunflower crop, it is crucial to develop genomic resources for *Pl. halstedii* to achieve a better understanding of its infection biology and reveal new strategies for avoiding the loss of sunflower crop to this pathogen.

Like all downy mildews, *Pl. halstedii* is an obligate biotrophic pathogen and thus cannot be grown apart from its living host. Other sequenced obligate biotrophic oomycetes include the downy mildew *Hyaloperonospora arabidopsidis* [6] and the white rusts *Albugo candida* [7] and *Albugo laibachii* [3], which are all pathogens of Brassicaceae, including *Arabidopsis thaliana*. Apart from downy mildews the order Peronosporales also includes cultivable pathogens, such as the well-studied hemibiotrophic pathogen *Phytophthora infestans*, the causative agent of potato late blight, which triggered the Irish potato famine in the mid-19th century [8–10]. Genome sequences have been published for several *Phytophthora* species, e.g. *Ph. ramorum* [11], *Ph. sojae* [11], *Ph. infestans* [12], *Ph. lateralis* [13] and *Ph. capsici* [14]. In addition, the necrotrophic phytopathogen *Pythium ultimum* [15] and the fish pathogen *Saprolegnia parasitica* [16] have been sequenced. These genome sequences have provided interesting insights into the evolution of oomycete pathogens with reference to their lifestyles, particularly the loss or gain of pathways or genes responsible for a certain lifestyle [3, 6, 15, 17], e.g. in the evolution of biotrophy [6]. In this study, the assembled genome sequence of *Pl. halstedii* was analysed and compared to eight deeply sequenced oomycete genomes (*Al. laibachii*, *Hy. arabidopsidis, Ph. capsici*, *Ph. infestans*, *Ph. ramorum*, *Ph. sojae*, *Py. ultimum,* and *Sa. parasitica*). The genome of *Pseudoperonospora cubensis*, which causes downy mildew of cucurbit, has also been reported in the past [18], but the quality of its assembly, while sufficient for general aspects regarding cucurbit downy mildew pathogenicity, does not allow for in-depth comparative analyses, leaving only one downy mildew genome, from *Hy. arabidopsidis,* available for such studies.

The obligate biotrophic downy mildews constitute the most species-rich group within the oomycetes [1], and are derived from *Phytophthora*-like ancestors [19–22]. So far, their monophyly could not be ascertained, even though Runge et al. [22] obtained high support for a grouping of the two major lineages of the downy mildews included in their study. Based on phylogenomic investigations with limited taxon sampling, downy mildews were inferred to be the sister-group to *Phytophthora* [23, 24], contradicting earlier reports in which downy mildews were proposed to be nested within *Phytophthora* [19, 20, 22].

A hallmark of downy mildews, *Phytophthora* species, and other oomycetes is the presence of a distinct core set of around 60 phospholipid modifying and signalling enzymes (PMSE), which might be important for pathogenic interaction [6, 11, 12, 15, 16, 25–27]. With the exception of *Sa. parasitica*, the previously sequenced oomycetes lack the classical phospholipase C (PLC). The role of the PSME in plant pathogenicity has not been fully explored, and currently, most plant-pathogen interaction studies in oomycetes focus on effector proteins [6, 28–30].

During infection, plant pathogenic oomycetes secrete an arsenal of effector proteins that target intracellular or extracellular host processes and enable sustained colonisation [28]. A range of Nep1-like proteins (NLPs) was identified, which seem to be typical for the Peronosporales, and are either inducing cell death during the switch from biotrophy to necrotrophy, or, in case of downy mildews are thought to be involved in other processes [6]. Protease inhibitors are secreted in the extracellular space (apoplast) where they interact with and inhibit plant proteases to repress or induce defence reactions [29]. The production of protease inhibitors in oomycetes was first described in the potato late blight pathogen *Ph. infestans* with two major structural classes: (1) Kazal-like serine protease inhibitors (EPIs) [31, 32] and (2) cystatin-like cysteine protease inhibitors (EPICs) [33]. Further transcriptome sequencing revealed the presence of both structural classes in other oomycetes [34, 35]. Preliminary transcriptome analysis in *Pl. halstedii* reported one Kazal-like EPI effector and one cystatin-like EPIC effector [36].

A common feature of both the downy mildews and *Phytophthora* is the presence of RxLR effector proteins [6, 11, 12]. Studies on the evolution of oomycetes revealed a high degree of positive selection in putative secreted effector proteins [28, 30]. Of the RxLR effectors identified in oomycetes, 44 % contain a conserved 3D structural motif based on the WY-fold [37]. The WY-fold is reported to be restricted to the proteomes of peronosporalean oomycetes [38].

A few studies have reported pathogenicity related genes in the genome of other downy mildew pathogens apart from *Hy. arabidopsidis* and *Ps. cubensis*, e.g. in grape downy mildew, *Plasmopara viticola* [39] and sunflower downy mildew, *Plasmopara halstedii* [4, 36, 40]. Thus, more genomic resources for downy mildews are required for performing comparative genomic analyses with the aim to elucidate the evolution of this group of pathogens, especially in terms of pathogenicity effectors. But also some other aspects of oomycete genomics, such as secondary metabolism and hormone synthesis, have previously been neglected, despite their potential roles in pathogenicity. To contribute towards filling this knowledge gap, the genome and transcriptome of *Pl. halstedii* were sequenced and analysed.

The aims of this study include: (i) Conducting comparative genomic analyses with deeply-sequenced oomycete genomes for elucidating evolutionary patterns of these pathogens, (ii) *In-silico* prediction and annotation of the gene space and promoters of *Pl. halstedii*, with a focus on pathogenicity-related genes, and those involved in secondary metabolism and hormone production, (iii) Expression-profiling of the candidate pathogenicity related genes with respect to certain stages of infection, (iv) Elucidation of effector evolution, in particular the evolution of RxLR-like effectors and their canonical motifs in downy mildews and *Phytophthora* species.

## Results

### General genome features

The genome assembly of *Pl. halstedii* was performed using small-insert libraries with insert sizes of 300 bp and 800 bp and large-insert mate-pair libraries with insert sizes of 8 kbp and 20 kbp. Illumina sequencing of the four libraries with insert sizes of 300 bp, 800 bp, 8 kbp, and 20 kbp generated 42.92, 36.13, 73.89 and 70.56 millions of paired-end reads, respectively. Illumina standard adapter and primer sequences were removed from these reads and further quality control (QC) trimming was performed using an average phred quality score cut-off of 20 and a minimum length threshold of 72 bp. Using these filters, 72.03 %, 57.20 %, 44.71 % and 50.57 % paired-end reads were retained for the libraries with insert sizes of 300 bp, 800 bp, 8kbp and 20 kbp, respectively. All reads were assembled using the Velvet [41] genome assembler v1.2.09 and the resulting scaffolds were compared to the NCBI nt (nucleotide) database using Blast to check for bacterial and host plant contamination (Additional file 1). A local database of genomes of possible contaminants was created and a mapping of the QC filtered reads on this database was performed. Contamination filters filtered around one per cent of the raw reads, which mapped mostly to bacterial genomes and the genome of the host plant, sunflower. All reads which mapped to the contaminant genomes were not used in the genome assembly.

**Fig. 1:**
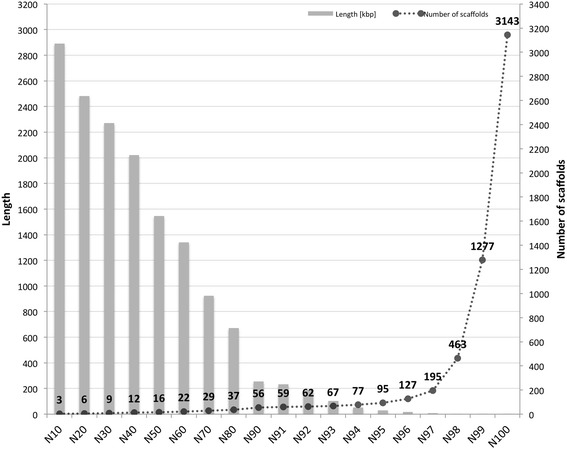
Genome assembly quality assessment in terms of length of the shortest scaffold in each N-class and the number of scaffolds. The quality of the genome assembly was assessed by first sorting all 3143 nuclear scaffolds length-wise from the largest to the smallest scaffold. Then N-classes were defined, where N represents the percentage of genome covered by considering the assembled genome size. The length given for each N-class represents the length of the smallest scaffold present in that particular N-class. The number of scaffolds represents the number of scaffolds present in the respective N-class. The sharp rise after N98 represents the unresolved small contigs, the majority of which are repeat elements

Filtered reads were used to generate the final genome assembly, with a total length of 75.3 Mb, with an N_50_ scaffold length of 1.54 Mb. This assembly consisted of 3162 scaffolds, comprising 7857 contigs in total. The longest contig was 297.2 kb and the N_50_ contig length was 58.1 kb. To assess the quality of this genome assembly, the number of scaffolds and the length of the shortest scaffold of the respective class from N_10_ to N_100_ were plotted (Fig. 1). 95 % of the nuclear genome was assembled in only 95 scaffolds, indicating a highly contiguous genome assembly.

**Fig. 2:**
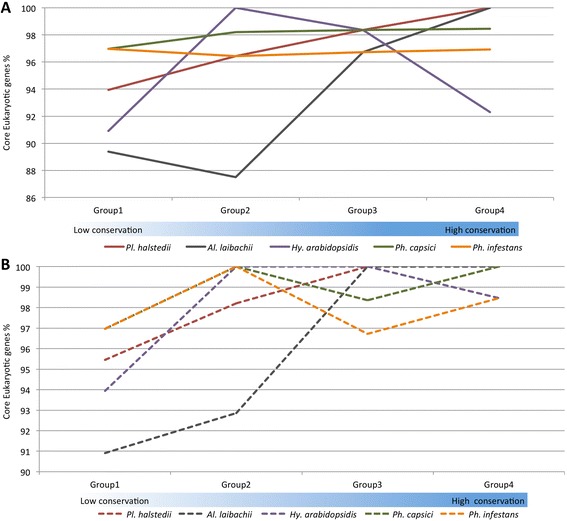
Genome completeness and continuity assessments in terms of core housekeeping genes. Genome completeness in terms of core eukaryotic genes was assessed using the CEGMA pipeline. The CEGMA pipeline has categorized 458 core genes into 4 groups on the basis of their conservation, from the least conserved group 1 to the most conserved group 4. **a** Genome completeness in terms of complete mapping. **b** Genome completeness estimations in terms of partial mapping

The completeness of the genome assembly was assessed using the CEGMA pipeline [42]. This confirmed the presence of 98.41 % of the core conserved genes by partial mapping and 97.18 % by complete mapping. Similar analyses on other sequenced oomycete genomes (Table 1) revealed that the assembled genome of *Pl. halstedii* is slightly more complete than those of *Al. laibachii* and *Hy. arabidopsidis,* while it is similar in completeness to the assembled genomes of *Phytophthora* spp., *Py. ultimum* and *Sa. parasitica* (Fig. 2). In addition, genome comparisons revealed that the genomes of the obligate biotrophic oomycetes (*Hy. arabidopsidis*, *Pl. halstedii* and *Al. laibachii*) are more AT-rich than the other species (Table 1).

**Table 1:** Genetic features of oomycete genomes

	*Pl. halstedii*	*Al. laibachii*	*Hy. arabidopsidis*	*Ph. capsici*	*Ph. infestans*	*Ph. ramorum*	*Ph. sojae*	*Py. ultimum*	*Sa. parasitica*
Assembled genome size (Mb)	75.32	32.76	78.89	64.02	228.54	66.65	82.6	44.91	53.09
N50 scaffold size (Mb)	1.54	0.06	0.33	0.7	1.58	0.3	7.6	0.83	0.28
N50 count	16	130	70	29	38	63	4	19	46
Longest scaffold size (Mb)	3.42	0.58	1.23	2.71	6.92	1.24	13.39	1.82	1.61
Number of scaffolds	3,162	3,827	3,044	917	4,921	2,576	83	975	1,442
Genes	15,469	13,804	14,321	19,805	17,787	16,066	26,584	15,322	20,088
CDS	40,334	43,014	28,165	42,673	49,146	40,639	63,242	39,949	79,762
Gaps (N %)	11.32	0	10.22	12.47	16.81	18.35	3.96	4.72	9.33
Repeat elements (%)	39 %	22 %	43 %	19 %	74 %	28 %	39 %	7 %	40 %
Secretome^a^	631 (631)	262 (672)	649 (1054)	1141 (1176)	1501 (1588)	1339 (1523)	1978 (1867)	926 (843)	1256 (1255)
Genome									
AT %	54.70	55.65	52.78	49.57	49.03	46.14	45.39	47.69	41.54
GC %	45.29	44.34	47.21	50.42	50.96	53.85	54.60	52.30	
Coding sequences									
AT %	54.02	54.29	46.87	46.62	45.99	41.95	41.55	43.4	37.67
GC %	45.96	45.71	53.11	53.37	54.02	58.05	58.45	56.6	62.33
CEGMA									
Complete KOG mapping (%)	97.18 %	93.40 %	95.39 %	98.00 %	96.76 %	96.44 %	98.02 %	97.13 %	97.20 %
Partial KOG mapping (%)	98.41 %	95.94 %	98.10 %	98.83 %	98.03 %	98.45 %	99.24 %	97.58 %	98.79 %

^a^Numbers in bracket represent the published secretome size

Gene prediction was performed using both *ab-initio* (without using RNA-seq data support) and evidence-based (RNA-Seq transcript mapping) approaches. This combined approach (Additional file 2: Figure S1) resulted in the prediction of a total of 15,469 protein encoding genes. Standalone Panther [43] protein class information and InterPro [44] protein family and domain information considering all protein encoding genes, revealed a plethora of pathogenicity related proteins, as given in Table 2. The *Pl. halstedii* genome was predicted to encode 631 secreted putative pathogenicity effectors, comparable to other biotrophic oomycete genomes screened in the same way (Table 1).

**Table 2:** Candidate pathogenicity related genes in oomycetes genomes

	*Pl. halstedii*	*Hy. arabidopsidis*	*Ph. infestans*	*Py. ultimum*	*Al. laibachii*
ATP-binding cassette (ABC) transporter^a^	32	35 (53)	**127 (156)**	112 (173)	26 (41)
Phospholipase^a^	17	23 (13)	**43 (36)**	25 (20)	19 (13)
Lipase^a^	24	30 (10)	**57 (19)**	36 (31)	23 (12)
Cysteine protease^a^	54	51 (7)	64 (33)	**75 (42)**	48 (16)
Serine protease^a^	62	73 (34)	106 (60)	**149 (85)**	52 (−)
Aspartic protease^a^	15	14 (9)	19 (12)	**34 (22)**	16 (10)
Cutinase^b^	2	2 (2)	**4 (4)**	0 (0)	3 (2)
NPP1-like (necrosis-inducing proteins)^b^	19	21 **(29)**	**27** (27)	7 (7)	0 (0)
Pectate lyases^b^	3	8 (8)	**36 (30)**	15 (15)	0 (1)
Cytochrome P450s^b^	14	18 (16)	30 (19)	**44 (41)**	3 (3)
Pectin esterase^b^	5	4 (4)	**11 (11)**	0 (0)	0 (0)
Elictins like^b^	16	16 (1)	45 (40)	**46 (24)**	9 (3)
RxLR effector family candidates^c^	274	134	**505**	0	49
Crincklers (CRN family candidates)^c^	77	20	**196**	26	3

^a^Generated using PANTHER; ^b^From InterproScan; ^c^Generated manually; Numbers in bracket represent the published number of predicted genes. Numbers in bold represent the highest number of genes

### Heterozygosity

The rate of heterozygosity in the sequenced isolate of *Pl. halstedii* is very low, which is consistent with multiple generations of inbreeding (‘selfing’) through homothallism [45]. Only 120 sites per Mb have a major allele frequency between 0.45 and 0.55 (Additional file 2: Figure S2). Although the global rate of heterozygosity is very low for the whole genome, we found two regions with apparently high rates of heterozygosity that correspond to predicted gene models, namely *PHALS_03871* on “Scaffold_2386” [ENA accession: CCYD01002371] (Additional file 2: Figure S3) and *PHALS_09122* on “Scaffold_350” [ENA accession: CCYD01000349] (Additional file 2: Figure S4). Each aligned sequence read-pair supports one of two apparent haplotypes (Additional file 2: Figure S5). However, it is possible that these loci represent paralogs sharing almost identical sequences that erroneously assembled into single gene models. This may also explain the increased depth of coverage in this region. The extremely low levels of heterozygosity precluded an assessment of ploidy levels throughout the genome.

### Phylogenetic analyses

**Fig. 3:**
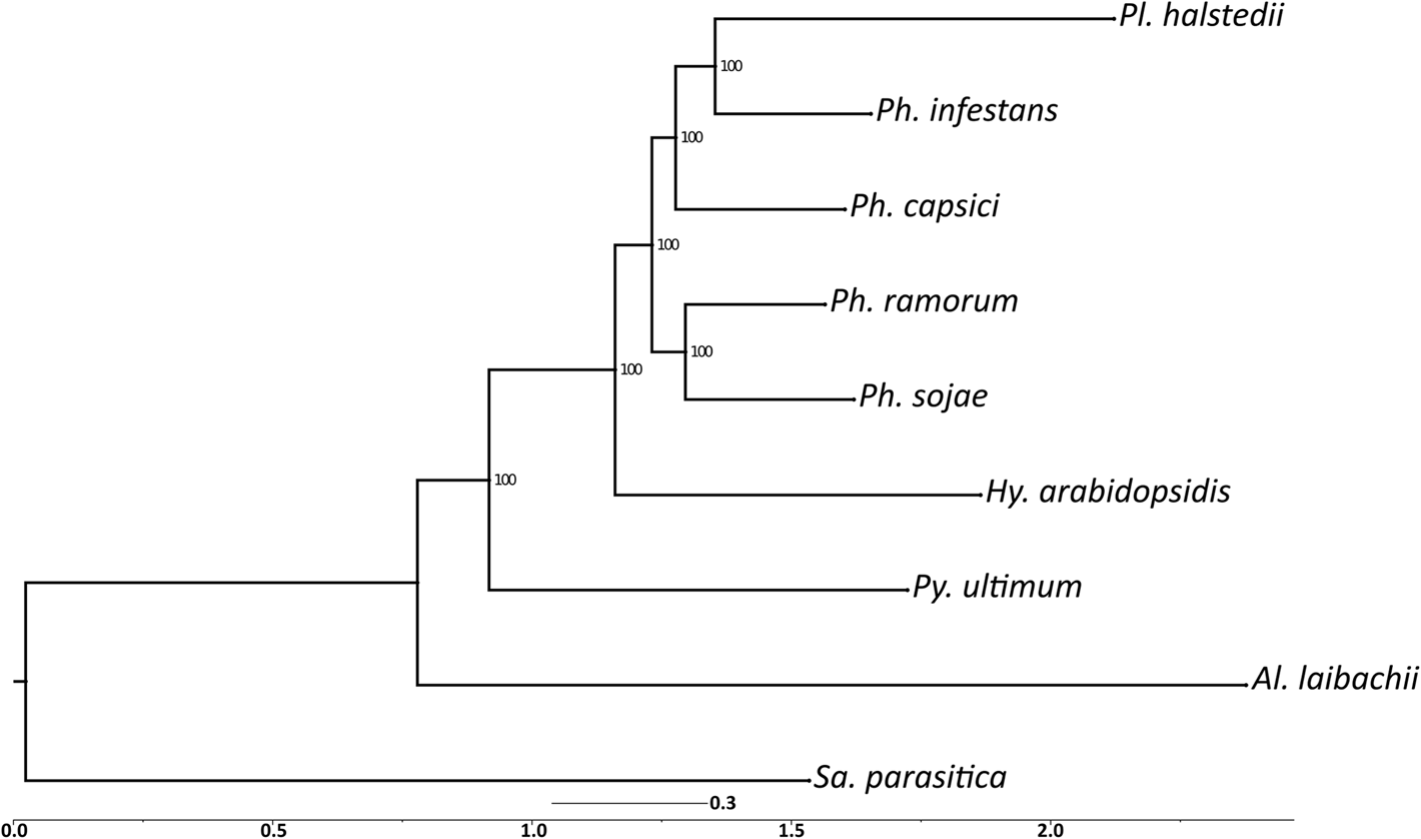
Phylogenetic relationship of deeply sequenced oomycetes. The phylogenetic analysis was done by considering the core orthologous genes predicted by the CEGMA pipeline. Multiple sequence alignments were performed using Mafft and phylogenetic relationships were inferred using the Maximum Likelihood algorithm as implemented in RAxML. Number on branches correspond to support values from 1000 bootstrap replicates

The sequences of core housekeeping genes identified by the CEGMA pipeline were used to infer phylogenetic relationships among these sequenced oomycete genomes. A total of 393 such core genes were found in the nine oomycete genomes. A phylogenetic tree, generated using RAxML (Fig. 3), revealed that *Pl. halstedii* is nested within the *Phytophthora* spp. with maximum bootstrap support, while *Hy. arabidopsidis* was placed as a sister-group of *Phytophthora* and *Pl. halstedii*, thus refuting monophyly of the downy mildews. It should be noted, however, that the sparse taxon sampling renders it possible that the phylogenetic position of the different species is an artefact resulting from highly divergent mutation rates. The nexus file containing the tree and alignment has been submitted to Dryad Digital Repository (doi:10.5061/dryad.qg3ft) and a local server (dx.doi.org/10.12761/SGN.2015.7).

### Repeat elements and microsatellite markers

Both *ab-initio* and reference-based repeat element prediction approaches were used to reveal that repeat elements make up 38.93 % of the genome of *Pl. halstedii*. Further characterization using computational methods resulted in the prediction of 7643 gypsy elements, 2183 TY1_Copia elements and 230 LINE elements. A total of 112 simple sequence repeat (SSR) markers (109 nuclear and three mitochondrial) of potential use in population genetic studies were identified (Additional file 3). Dinucleotide motifs were the most abundant type of repeats (89.36 %), followed by tri- (8.16 %), penta- (1.05 %), and tetra-nucleotide motifs (0.6 %) (Additional file 4). None of the most frequent nucleotide motifs represented more than 1 % of the total SSRs (Additional file 5).

### Orthology

**Fig. 4:**
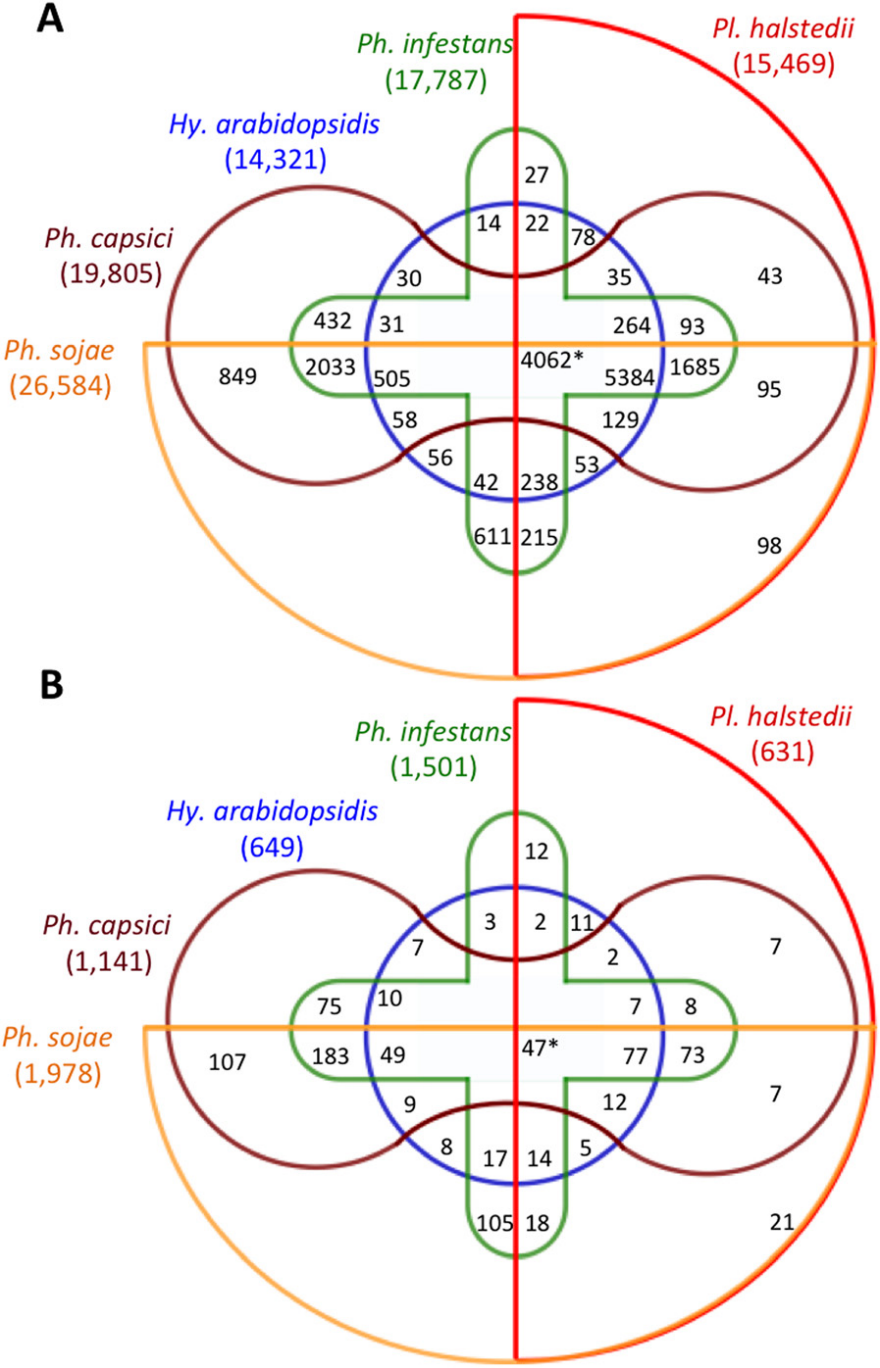
Number of ortholog groups within oomycete genomes. The number of ortholog groups among the genomes of *Hy. arabidopsidis*, *Ph. capsici*, *Ph. infestans*, *Ph. sojae*, and *Pl. halstedii*. **a** Number of ortholog groups found within the five genomes considering all protein-coding genes. **b** Number of ortholog groups within the five genomes considering all PSEP-encoding genes. Numbers in brackets represent the total number of genes tested in the analyses. Asterisks denote 1:1 orthologs among the five genomes

Orthology analysis was performed using the protein sequences of two obligate biotrophic members of the Peronosporaceae (*Hy. arabidopsidis* and *Pl. halstedii*) and three hemibiotrophic *Phytophthora* species. A total of 5384 orthologs were detected in the five genomes. Out of these 4062 were 1:1 orthologs (Fig. 4a). A similar analysis was performed considering the nine deeply sequenced oomycete genomes. These analyses revealed 3316 shared orthologs, of which 1737 were 1:1 orthologs.

A total of 631 putative secreted effector protein (PSEP)-encoding genes were predicted in the genome of *Pl. halstedii*. By applying these methods to the other available downy mildew genome, we could predict 649 PSEP-encoding genes in *Hy. arabidopsidis.* This suggests that both of these downy mildew pathogens require an almost identical amount of PSEP-encoding genes for biotrophic colonization of their phylogenetically divergent hosts. Orthology analyses revealed that the nine deeply sequenced oomycete genomes share five orthologs of PSEP-encoding genes (Additional file 6). Orthology searches considering the PSEPs in the plant parasitic Peronosporaceae (*Hy. arabidopsidis*, *Ph. capsici*, *Ph. infestans*, *Ph. sojae*, and *Pl. halstedii*) resulted in 77 PSEP orthologs (Fig. 4b) shared by the five genomes (Additional file 7). Our analyses suggest that there are in total 65 (Additional file 8) orthologs of PSEP-encoding genes among the four *Phytophthora* spp. and two downy mildew pathogen genomes. These orthologs code for 3, 1, 2 and 26 proteins classified as serine protease, Nep1-like proteins (NLPs), proteinase inhibitors and RxLR-like, respectively (Additional file 8). No secreted CRN was found conserved among the Peronosporaceae. However, orthology analyses of 49 CRNs of *Pl. halstedii* derived from protein encoding genes supported by gene predictions revealed the presence of orthologs of 32 CRNs of *Pl. halstedii* not predicted to be secreted in at least one of the eight other deeply sequenced oomycetes. Out of these 32 orthologs, six were present in *Pl. halstedii* and the four *Phytophthora* genomes and four were shared by *Pl. halstedii* and *Hy. arabidopsidis.* Only two orthologs were found among the two downy mildews and four *Phytophthora* genomes.

Notably, 183 PSEP-encoding gene orthologs were found in three deeply sequenced *Phytophthora* genomes but in none of the downy mildew genomes (Fig. 4b). Only 11 PSEPs were unique for the downy mildews and absent from the *Phytophthora* genomes (Additional file 9).

### Genome architecture

**Fig. 5:**
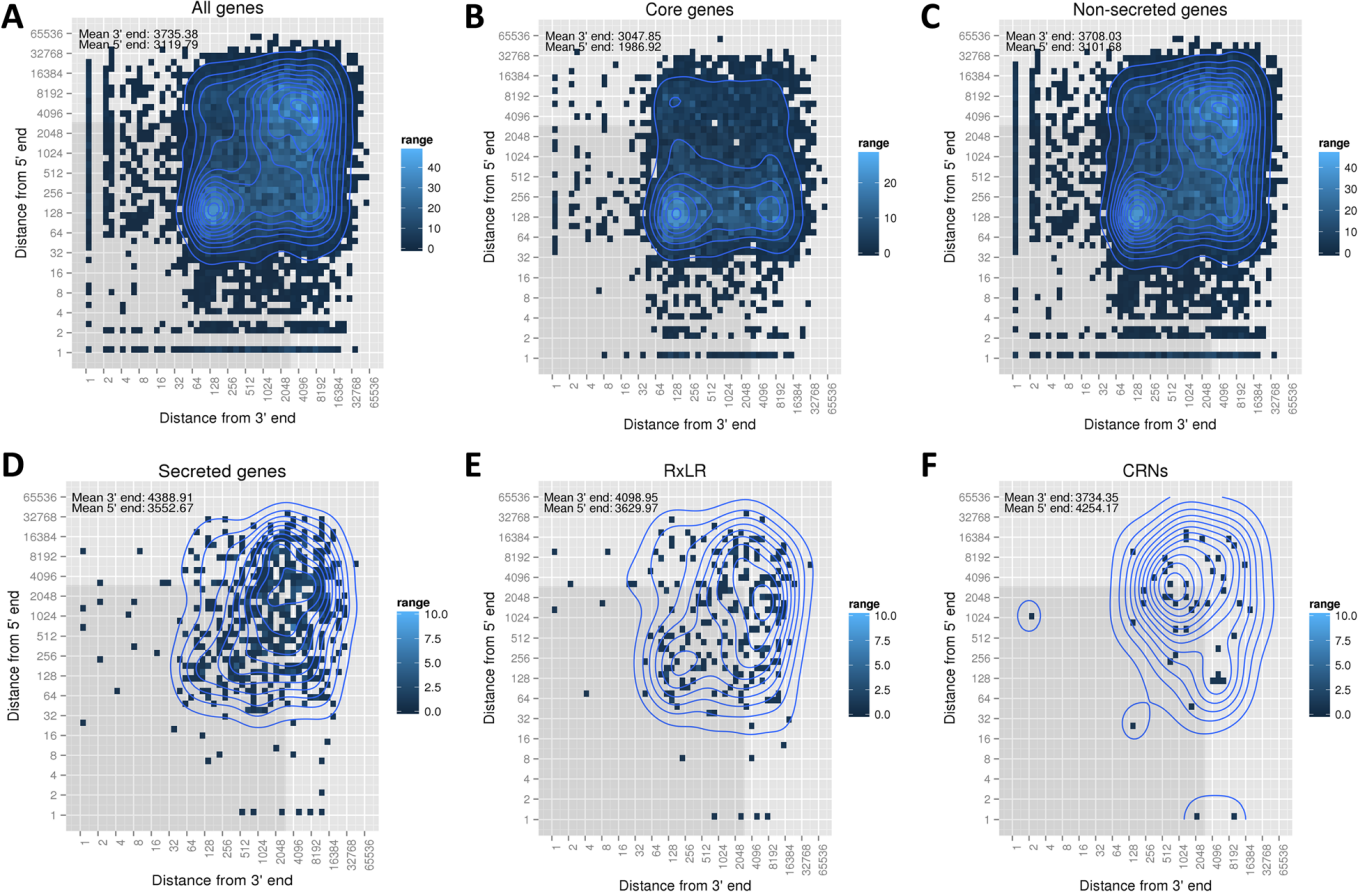
Heat maps illustrating gene density of the *Pl. halstedii* genome. Gene density as estimated by calculating the 5′ and 3′ flanking distances of (**a**) all protein encoding genes, (**b**) core genes (**c**) non-secreted protein encoding genes (**d**) secreted protein encoding genes, (**e**) candidate RxLR-like protein encoding genes, (**f**) CRN-like protein encoding genes. Grey shading highlights the area with both 5′ and 3′ distances below 3 kb

The lengths of gene flanking regions were calculated to estimate gene density in the *Pl. halstedii* genome. This revealed that the overall means of 5′ and 3′ flanking distances are 3.1 kb and 3.7 kb, respectively (Fig. 5a). The core ortholog genes of *Pl. halstedii* had mean 5′ and 3′ flanking distances of 1.9 kb and 3.0 kb (Fig. 5b). Similar analyses were specifically performed for all genes that encode PSEP, RxLR-like, and Crinkler (CRN)-like protein encoding genes (Fig. 5). The majority of such genes are localized in regions sparser in genes (3′ or 5′ gene flanking distance more than 4 kb) in comparison to non-effector protein-encoding genes.

### Promoters

The intergenic regions upstream of *Pl. halstedii* start codons span a wide size range, which is consistent with organization of the genome into gene dense region (GDR) and gene-sparse region (GSR). About 51 % of such regions are < 2 kb, with a median size of 418 nt (Additional file 2: Figure S6), suggesting that the typical *Pl. halstedii* promoter is compact. This value is based on scaffolds containing at least 100 genes, 13,349 genes in total, to avoid bias due to gaps in the assembly. The fraction of closely spaced (<2 kb) genes in *Pl. halstedii* is smaller than that of *Ph. infestans* (51 % versus 67 %), despite the much larger genome of the latter [46].

**Fig. 6:**
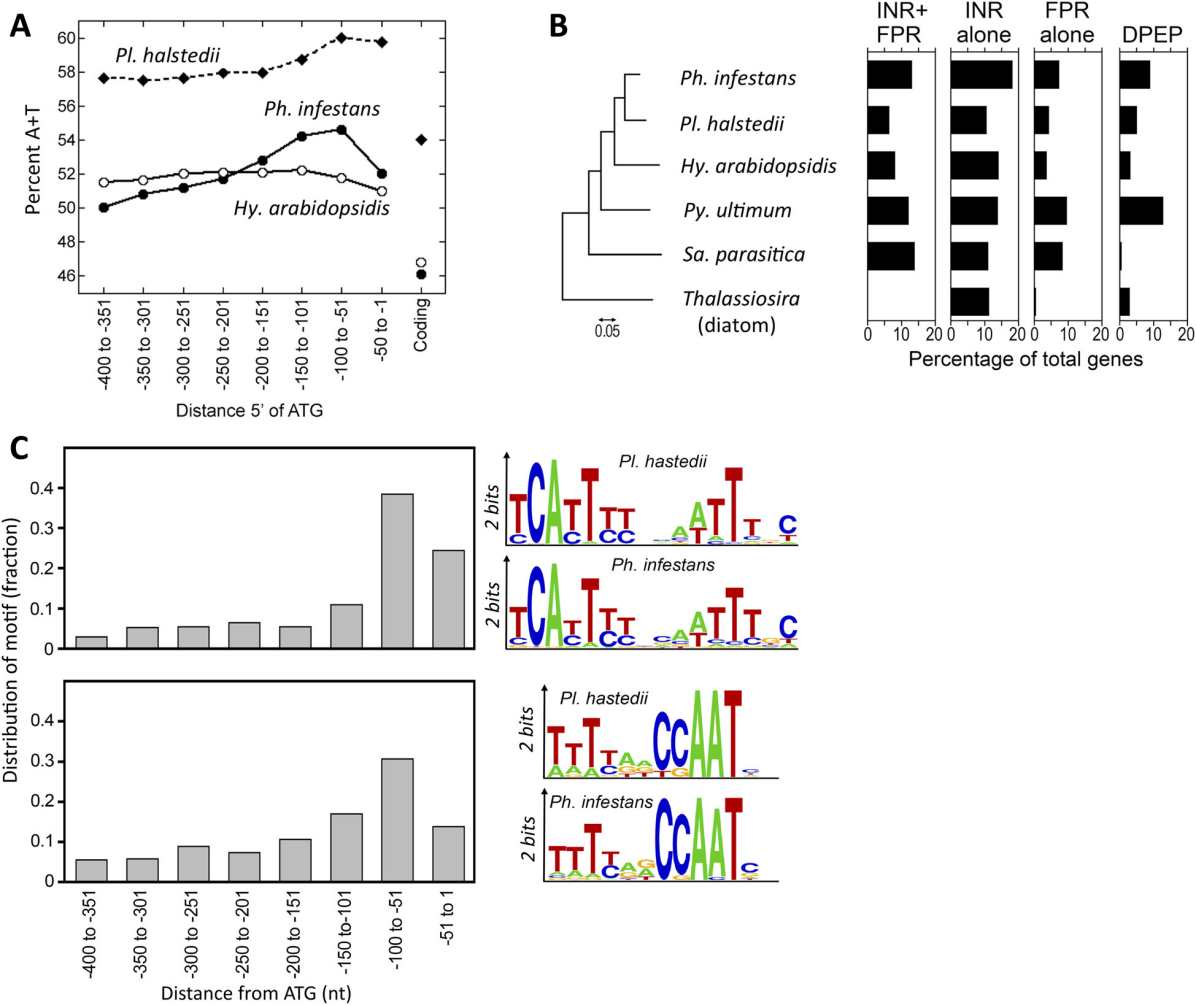
Features of promoters. **a** A + T content of coding regions and 50-nt intervals within promoters from *Pl. halstedii, Ph. infestans, and Hy. arabidopsidis.* **b** Distribution of motifs in different Straminipila. Searches for the INR + FPR supra-motif, INR, FPR, and DPEP were performed in five oomycetes *(Ph. infestans, Pl. halstedii, Hy. arabidopsidis, Py. ultimum, Sa. parasitica)* and the diatom *Thalassiosira pseudonana.* Bars show the percentage of promoters within each species that contain the motifs within 200 nt of the start codon, corrected for false discovery. The figure on the left is a neighbor-joining tree based on ribosomal RNA and internal transcribed spacer (ITS) sequences. **c** Positional bias of INR + FPR supra-motif and CCAAT within *Pl. halstedii* promoters. The right of the panel compares the content of the two motifs in *Ph. infestans* and *Pl. halstedii*

Both coding and promoter regions in *Pl. halstedii* are more AT-rich than those of most other sequenced oomycetes (Fig. 6a). The A + T content within promoters peaks at nearly 60 % about 75 nt upstream of the translation start, the region in which the transcription start site would be expected.

Prior studies of *Phytophthora* spp. identified core promoter elements [46] with a fair degree of conservation in other oomycetes [47]. These include the 7-nt Initiator (INR), 7-nt FPR, and 7-nt DPEP elements. Frequently, the INR and FPR elements [47] co-occur in a 16-nt INR + FPR supra-motif. All of these elements were present in *Pl. halstedii* (Fig. 6b and c). However, the motifs were less frequently detected in *Pl. halstedii* compared to *Ph. infestans.* For instance, the INR + FPR supra-motif was detected two times less in the downy mildew. A similar reduction in the frequency of this element was observed in *Hy. arabidopsidis.*

About 2/3 of *Pl. halstedii* promoters lacked any recognizable core promoter motif. Attempts to identify additional core promoter motifs using maximum expectation methods (e.g. the MEME program [48]), using motif sizes between 6 and 12 nucleotides, were unsuccessful.

Few of the regulatory sites identified in *Phytophthora* were found conserved in *Pl. halstedii.* Two recent studies predicted general and stage-specific transcription factor binding sites (TFBSs) in *Ph. infestans,* with a total of 113 motifs identified with high confidence [47, 49]*.* Approximately 47 of the motifs appeared to be over-represented in *Pl. halstedii* promoters compared to shuffled promoter sequences (*P* < 0.01 by Fisher’s Exact Test). Furthermore, most of the hits in *Pl. halstedii* lacked the positional or orientational biases seen for most TFBSs in *Ph. infestans.* Most of such motifs may not be functionally conserved in *Ph. infestans* and *Pl. halstedii*, however, since 35 also appeared to be over-represented in promoters from *Drosophila*.

One TFBS that did appear to be conserved between the species was the CCAAT box. This binds an evolutionarily conserved transcription factor that influences initiation and core motif recognition [50]. On average, the CCAAT motif in *Pl. halstedii* resides slightly upstream of motifs such as INR + FPR (Fig. 6c) or the INR alone. Even though attempts to identify additional *Pl. halstedii* TFBSs using MEME with total promoters were unsuccessful, it may be possible to identify such motifs in future studies by evaluating the promoters of co-regulated genes.

### Pathways related to phospholipid signalling

Screening the genome for homologs of genes encoding phospholipid modifying and signaling enzymes (PMSEs) showed that nearly all PMSE genes as identified from other oomycetes have an ortholog in *Pl. halstedii* (Additional files 10 and 11) with the exception of two phosphatidylinositol kinase (PIK) genes (one type C and one type E) that are absent*.* In addition, there is a significant reduction in the number of phospholipase D (PLD) genes compared to *Ph. infestans*, 6 versus 18 [25]. Also, *Pl. halstedii* is the first oomycete to be sequenced in which the oomycete-specific trans-membrane domains-PLD (TM-PLD) class is absent. The second largest group of PMSEs is made up by the G-protein coupled receptor (GPCR)-phosphatidylinositol phosphate kinases (PIPKs), known as GPCR-PIPKs (GKs), with 12 members in most oomycetes. This group is highly conserved as illustrated in Additional file 2: Figure S7 for GK9. As in most oomycetes, there are no gene predictions supporting the presence of a phospholipase C (Additional file 11).

### Secondary metabolites

An antiSMASH [51] analysis of genes and gene clusters involved in the biosynthesis of secondary metabolites detected the presence of four loci encoding nonribosomal peptide synthetases (NRPS) [52]. *PhalNRPS_1* and *PhalNRPS_4* show a two-domain structure with an adenylation (A) domain with unknown amino or 2-ketocarboxylic acid specificity and a thiolation (T) domain (Additional file 2: Figure S8). BlastP searches revealed that NRPS_1 and NRPS_4 are widespread in fungi and oomycetes. The *PhalNRPS_2* operon encodes an A-T didomain NRPS and has a flanking gene encoding a PQQ binding protein, which might be involved in the reductive release of the T domain-bound thioester (Additional file 2: Figure S8) [53].

*PhalNRPS_3* is an unusual monomodular NRPS with a four-domain composition: An N-terminal thioesterase (TE) domain followed by an A and a T domain and a C-terminal reductase (Red) domain potentially involved in reductive release of the bound thioester (Additional file 2: Figure S8) [53]. The coexistence here of the TE and Red domains is uncommon as both usually act on the C-terminus of the NRPS-build peptide chain. Interestingly, NRPS_3 homologs are also present in other oomycete genomes including *Ph. infestans* and *Ph. sojae*. Although the function of the N-terminal TE domain is unknown it might be involved in precursor supply for the A domain.

### Phytohormones

Phytohormones are a group of metabolites that are also potential virulence determinants. The close coevolution of downy mildews with their hosts renders it possible that the former have acquired the ability to produce hormones to manipulate their host plants in addition to secreting effector proteins. Thus pathways leading to the production of plant hormones were investigated in detail. One of the crucial pathways that can lead to the production of salicylate and auxin originates from the shikimate pathway that catalyses the stepwise conversion of erythrose-4-phosphate and phosphoenolpyruvate to chorismate. Comparable to other oomycetes and plants, in *Pl. halstedii* all steps are catalysed by the pentafunctional AROM complex. Examination of genes involved in the salicylate biosynthesis pathway that branches from chorismate via isochorismate to salicylate neither identified an isochorismate synthase nor an isochorismate pyruvate lyase, rendering it unlikely that salicylate is synthesized by *Pl. halstedii*. Even though all enzymes are present in the genome to enable synthesis of L-tryptophan from chorismate, an important precursor of auxin production (Additional file 2: Figure S9), none of the enzymes involved in indole-3-acetic acid biosynthesis was found. It thus seems highly unlikely that auxin derivatives are synthesized by *Pl. halstedii*.

We could further identify all enzymes needed for the biosynthesis of geranyl-gernayl-PP from acetyl-CoA but no key enzymes for diterpenoid biosynthesis (Additional file 2: Figure S10). Therefore the biosynthesis of gibberellins by *Pl. halstedii* is unlikely. The pathogen has the potential to synthesize phytoene from geranyl-geranyl-PP via phytoene synthase but none of the genes encoding the downstream enzymes such as phytoene desaturase was identified. It is therefore unlikely that *Pl. halstedii* is able to synthesize complex carotenoids, abscisic acid derivatives or strigolactones (Additional file 2: Figure S10).

Despite the absence of most genes encoding proteins involved in the production of phytohormones, candidates encoding all key steps of cytokinin production were present in the genome of *Pl. halstedii* (Additional file 2: Figure S10). We can therefore speculate that *Pl. halstedii* produces cytokinins. In addition, all the enzymes required to produce brassinolide from campesterol (Additional file 2: Figure S11) seem to be present. Based on the identification of two potential cholesterol transporters we hypothesize that sterols are obtained from the plant and converted into campesterol. Brassinolide together with cytokinins might be important virulence factors and contribute to the dwarfing and stunting of infected sunflower plants. The potential capacity of plant hormone biosynthesis in *Pl. halstedii* has been summarised in Additional file 12.

### Necrosis and ethylene-inducing peptide 1 (Nep1)-like proteins (NLPs)

*Plasmopara halstedii* has 19 NLP genes (Additional file 13) and one NLP pseudogene (Additional file 14). Of these 19 genes, there are two type 1 NLPs and 17 type 1a NLPs, which are distinguished based on substitutions in the cation binding pocket that is required for induction of necrosis by cytotoxic NLPs [54]. Features of these NLPs have been listed in Additional file 15. Remarkably, one of its type 1 NLPs (PHALS_06084) is very closely related to the non-cytotoxic *Hy. arabidopsidis* HaNLP3 protein [55], including the presence of the N-terminal Q-rich region and a second disulphide bridge (Additional file 2: Figure S12). Multiple sequence alignments and sequence features of all NLPs are given in Additional file 2: Figure S13.

*Plasmopara halstedii*-specific expansion of type 1a NLPs, similar to that observed in *Hy. arabidopsidis* [55], is depicted in Additional file 2: Figure S14, which shows two expanded groups of *Pl. halstedii* NLPs. Group 1 clusters with *Phytophthora* NLPs, while group 2 clusters with several NLPs of *Bremia lactucae* [56], the lettuce downy mildew pathogen, representing a potential apomorphy of the group of downy mildews with pyriform haustoria. Three NLPs (PHALS_01213*,* PHALS_05247*,* and PHALS_13274) group together with HaNLP5 and type 1a NLPs of other Peronosporaceae. PHALS_01213 is most closely related to *Br. lactucae* NLP6 (Additional file 2: Figure S14). While the expanded HaNLPs group in one clade, the PhalNLPs display two divergent species–specific expansions (Additional file 2: Figure S14).

The low number of *PhalNLP* pseudogenes is remarkable compared to the situation in the *Hy. arabidopsidis* genome where 14 *HaNLP* genes and 15 *HaNLP* pseudogenes were identified [55], and in the *Phytophthora sojae* genome where 33 *PsNLP* genes and 37 *PsNLP* pseudogenes were identified [57]. In *Pl. halstedii* 19 *PhalNLP* genes were identified, yet only one *PhalNLP* pseudogene (Additional file 2: Figure S15); the latter has a premature stop codon when compared to its most closely related *PhalNLP* gene PHALS_14423 (Additional file 2: Figure S16).

In total, 15 *PhalNLPs* showed induced expression during infection. In particular, members of group 2 are up-regulated during infection, compared to their expression in spores, suggesting the identified *PhalNLP* proteins in *Pl. halstedii* play a role during the infection process (Additional file 2: Figure S17).

### Protease inhibitors

Protease-inhibiting proteins are virulence factors that impair the function of plant proteinases targeted against pathogenicity effectors. The genome of *Pl. halstedii* has a total of 19 genes encoding putative Kazal-like EPI effectors and 4 putative cystatin-like EPIC effectors (Table 3; Additional file 15). Sequence alignments of the *Pl. halstedii* Kazal-like inhibitor domains and 139 Kazal-like domains from six other oomycete pathogens show that these sequences contain the six conserved cysteines with the consensus pattern C-X_3,4-_C-X_7_-C-X_6_-Y-X_3_-C-X_6_-C-X_9,12,13,14_-C (Additional file 2: Figure S18). Some of the serine-like inhibitor effectors in *Ph. infestans* exhibit atypical Kazal-like domains that lack cysteines C3 and C6. We identified an independent deletion of cysteines C2 and C4 in serine-like inhibitor effectors from *Pl. halstedii* that also results in atypical Kazal-like domains (e.g. in PHALS_09920).

**Table 3:** Summary of protease inhibitor effectors from seven pathogenic oomycete species

Description	No. of protease inhibitors effectors	No. of Kazal-like inhibitor effectors	Highest No. of Kazal-like domains	No. of cystatin-like inhibitor effectors	Highest No. of cystatin-like domains
*Ph. infestans* ^*a*^	41	33	7	8	1
*Py. ultimum* ^*b*^	21	15	5	6	1
*Hy. arabidopsidis* ^*c*^	5	1	4	4	1
*Al. laibachii* ^*d*^	10	8	2	2	2
*Pl. halstedii* ^*e*^	23	19	5	4	1
*Sa. parasitica* ^*f*^	14	8	5	6	3
*Aphanomyces euteiches* ^*g*^	2	1	5	1	2

^a-d,f^Pathogenic oomycete species with available whole genome sequences

^e^Genome sequence and effector annotation is described in this study

^g^Oomycete species where there are only expressed sequence tag (EST) data [102]. This genome may contain more protease inhibitors that were not detected in the transcriptome analysis

The N-terminal Trunk (G), the Loop1 (QxVxG) and Loop2 (W) motifs representative of cystatin-like cysteine inhibitors are conserved in four of the predicted cystatin-like proteins from *Pl. halstedii* (Additional file 2: Figure S19). All Kazal-like and cystatin-like sequences are listed in Additional file 16 and Additional file 17, respectively.

### Crinkler (CRN)-like proteins

The genome of *Pl. halstedii* was searched for CRN protein-coding genes. For predicting CRNs, both regular expression and Hidden Markov Model (HMM) methods were used [12]. A total of 49 CRNs were predicted from the protein-coding genes (Additional file 18). Out of these 49 CRNs, 4 had both the canonical LFLAK and HVLVVVP motifs.

In a second approach a total of 200 and 139 sequences were derived from open reading frames (ORFs) using full length and LFLAK HMMs, respectively, which were similar by Blast and HMM alignments to previously known CRNs. A total of 125 CRN-like proteins matched both HMMs (Additional file 2: Figure S20). Manual curation based on high-confidence motifs known from previous studies reduced this number to 55. In total, 28 CRN motifs encoded in ORFs (genome stretches with an ATG followed by at least 30 codons before a stop codon) and were not predicted as protein-coding genes (Additional file 19). A total of 77 putative CRNs were identified in the genome of *Pl. halstedii* by merging the outputs from both translated ORFs containing high-confidence CRN signatures and iterative HMM predictions from translated predicted genes (Additional file 2: Figure S20). Consistent with previous studies [15, 40], only a handful of putative CRNs were predicted to be secreted. Out of the 77 putative CRNs, only 2 bore classical secretion signals based on our secretome prediction pipeline (Additional file 1). However, in total 11 CRNs were having a signal peptide probability (SignalP v2) greater than 0.5. All 15 CRNs of *Pl. halstedii* reported in a previous study [40] were also present in this set of 77 CRNs.

### RxLR-like proteins

**Fig. 7:**
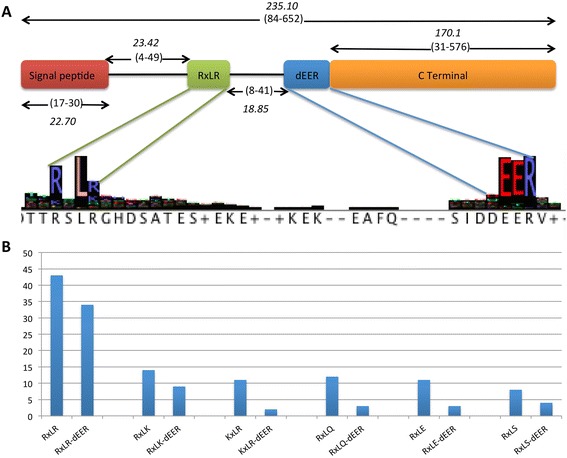
Features of RxLR-dEER-like effectors and frequency of the RxLR and RxLR-dEER-like proteins in the genome of *Pl. halstedii*: **a** Sequence features of the RxLR-dEER-like proteins were calculated from predicted putative RxLR-like proteins. Numbers in brackets represent the minimum and maximum values of distances and number in italics represents the corresponding mean value. Multiple sequence alignments were performed by using Mafft and sequence logos were generated using jalview. **b** Bar plot representing the number of RxLR-like and RxLR-dEER-like proteins in the predicted secretome of *Pl. halstedii*

Candidate secreted proteins with RxLR-dEER-like domains were predicted using both regular expression and HMM-based methods (Additional file 2: Figure S21, S22A-B). A total of 260 candidate RxLR-like proteins were predicted from PSEP-en coding genes (Additional file 20). Out of these 260 RxLR-like proteins, 21 sequences had the exact RxLR-dEER motif. Features and occurrences of the RxLR-dEER like proteins were further analysed (Fig. 7a) and revealed an overrepresentation of some RxLR-dEER variants, e.g. KxLR-dEER and RxLK-dEER (Fig. 7b).

Fourteen RxLR-like proteins (Additional file 21) were found in ORFs that were not predicted as protein coding genes, as these resided in highly repetitive regions. These 14 putative RxLR-like effectors represent high conservation of RxLR-dEER motifs (Additional file 2: Figure S22C). Out of these 14, 7 were masked as repeat elements by the repeat element masking pipeline. The other 7 RxLR-like protein-coding ORFs were surrounded by repeat-rich regions and their start codon positions were not well supported. By combining both predictions from protein sequences and translated ORFs from repetitive regions, a total of 274 RxLR-like proteins were predicted. Out of these 274, 34 had both the canonical RxLR and dEER motifs.

To determine whether the *Pl. halstedii* genome encodes RxLR-like proteins with WY-folds, HMMER was used to search the predicted proteome using the WY-fold HMM. In total 132 proteins out of the 15,469 protein sequences were predicted to have at least one WY-fold (Additional file 22). Among these, 16 proteins were predicted to be secreted, of which 8 contained RxLR-like motifs. The number of WY-folds present in each protein ranged from 1 to 7. In the predicted secretome, all WY-fold containing proteins had no known functional annotations except PHALS_02683, which was annotated as similar to a reverse transcriptase.

### Conservation of RxLR-like effectors within *Phytophthora* species and downy mildew pathogens

**Fig. 8:**
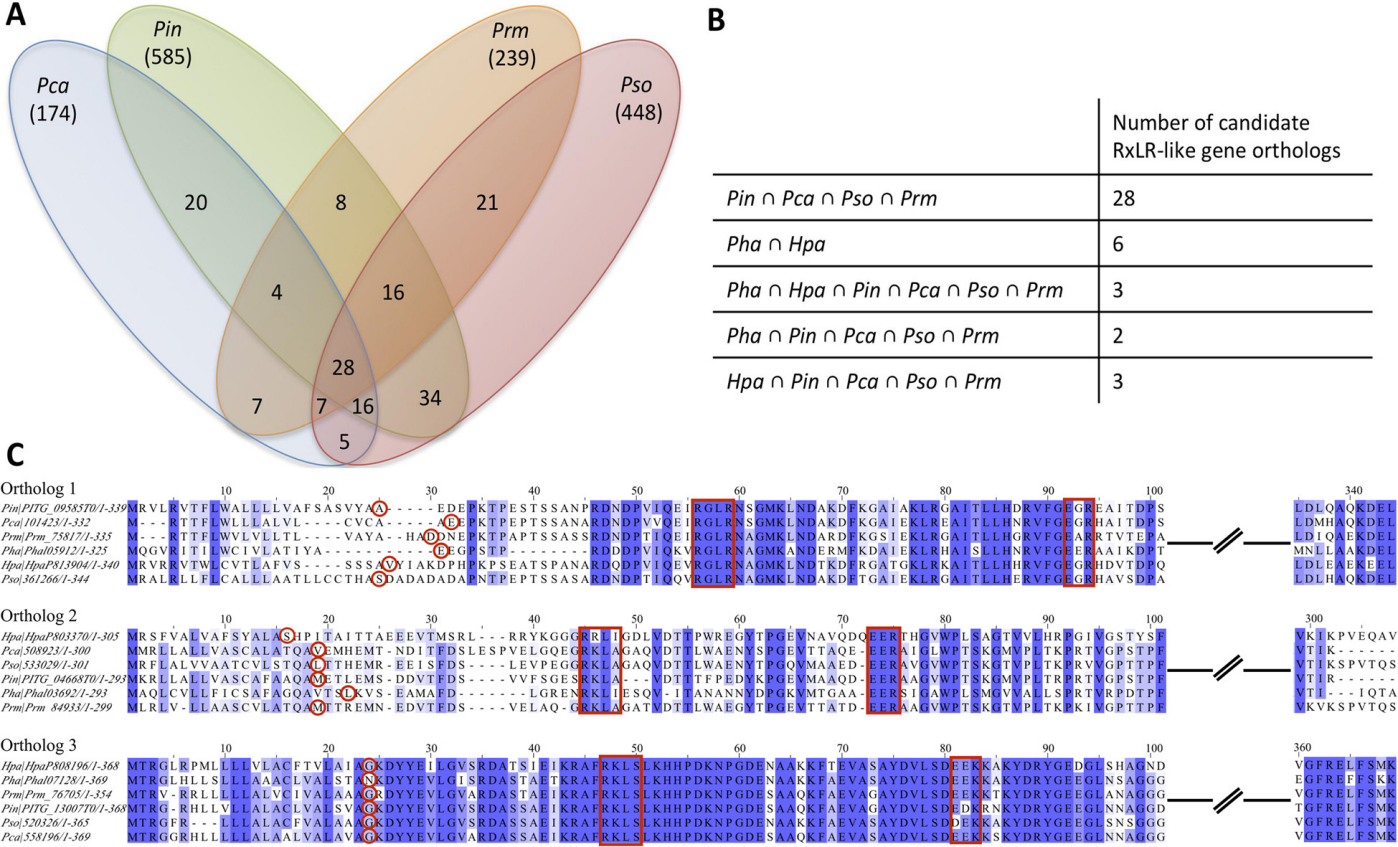
Orthologs of RxLR-dEER-like proteins within downy mildew pathogen genomes and *Phytophthora* spp*.* genomes: High confidence RxLR-dEER-like proteins from the secretome of downy mildew and *Phytophthora* spp. genomes were predicted and orthology analyses were performed with OrthoMCL to predict orthologs of RxLR-dEER-like proteins. *Pha, Hpa, Pca, Pin, Pso,* and *Prm* refer to *Pl. halstedii*, *Hy. arabidopsidis*, *Ph. capsici, Ph. infestans*, *Ph. sojae*, and *Ph. ramorum*, respectively. **a** Venn diagram showing the number of orthologs among the four *Phytophthora* spp*.* genomes. **b** Table summarising the number of orthologs shared by downy mildews and *Phytophthora* spp*.* genomes. **c** Sequence alignments of the three candidate orthologs of putative RxLR-dEER proteins among the six genomes. Multiple sequence alignments were performed using Mafft and alignment graphics were generated using Jalview. Cleavage sites predicted by SignalP are highlighted by red circles, RxLR/dEER-like motifs are highlighted by red boxes

To look for orthologs of RxLR-like proteins among oomycete genomes, a subset of high-confidence RxLR-like proteins (categories AAA, AA and A in Additional file 20; the categorisation method is explained in Additional file 1) was generated for *Pl. halstedii* as for the four *Phytophthora* spp. and *Hy. arabidopsidis*. Orthology predictions generated 28 orthologs (Table 4; multiple sequence alignments are shown in Additional file 2: Figure S23) of RxLR-like proteins that were found in the four *Phytophthora* spp. genomes (Fig. 8a). Multiple sequence alignments of these 28 orthologs revealed high sequence conservation (Additional file 2: Figure S23). However, only six such orthologs (Additional file 23) were found in *Hy. arabidopsidis* and *Pl. halstedii* (Fig. 8b). Interestingly, only three high confidence RxLR-like proteins had orthologs in each of the six genomes (Additional file 24). Their multiple sequence alignments revealed a high degree of conservation (Fig. 8c; Additional file 2: Figure S24-S26), and all were predicted to be targeted to the cytoplasm after secretion and signal peptide cleavage. InterProScan searches revealed that *Pl. halstedii* protein sequences of Ortholog1 (PHALS_05912), Ortholog2 (PHALS_03692) and Ortholog3 (PHALS_07128) (Fig. 8c) contain a tetratricopeptide-like helical domain (IPR019734), a pectate lyase catalytic domain (IPR004898) and a DnaJ domain (IPR001623), respectively. Only one of the three orthologous genes (represented by PHALS_05912) had previously been annotated as a putative RxLR-effector (Additional file 24).

**Table 4:** 28 Orthologs of putative RxLR-like secreted proteins in four *Phytophthora* spp.

Ortholog count	*Ph. capsici*	*Ph. infestans*	*Ph. ramorum*	*Ph. sojae*	Annotation representative gene	*Ph. infestans* Annotations
1	Pca_16635	PITG_07736T0; PITG_19803T0; PITG_13535T0; PITG_13537T0; PITG_13536T0; PITG_13534T0; PITG_07766T0	Prm_76660; Prm_78801; Prm_78158; Prm_81834; Prm_76663; Prm_76672	Pso_287018	PITG_07736T0	Secreted RxLR effector peptide protein, putative
2	Pca_102742	PITG_14880T0; PITG_14884T0; PITG_13847T0	Prm_74367; Prm_79110; Prm_86912; Prm_79108; Prm_79107; Prm_74387; Prm_79119; Prm_85872	Pso_285707; Pso_285703	PITG_14880T0	RXLR effector family protein, putative
3	Pca_13936; Pca_13937; Pca_13953	PITG_06305T0; PITG_06290T0	Prm_83582; Prm_77765; Prm_85589; Prm_77763; Prm_77786; Prm_74178	Pso_286631; Pso_354880	PITG_06305T0	Secreted RxLR effector peptide protein, putative
4	Pca_14162; Pca_39353	PITG_05841T0; PITG_05846T0; PITG_06308T0; PITG_11952T0; PITG_15679T0	Prm_73724; Prm_86166; Prm_73707	Pso_285308; Pso_286675	PITG_05841T0	Secreted RxLR effector peptide protein, putative
5	Pca_10713	PITG_07566T0; PITG_07569T0	Prm_81825; Prm_81822; Prm_81823; Prm_78748	Pso_336774; Pso_286958	PITG_07566T0	Secreted RxLR effector peptide protein, putative
6	Pca_5670; Pca_133116; Pca_107349	PITG_17063T0; PITG_18404T0	Prm_81907; Prm_81908	Pso_284378	PITG_17063T0	Secreted RxLR effector peptide protein, putative
7	Pca_121504; Pca_19144; Pca_536383	PITG_15556T0	Prm_82880	Pso_288650; Pso_288648; Pso_288647	PITG_15556T0	Secreted RxLR effector peptide protein, putative
8	Pca_572048	PITG_13093T0	Prm_86463	Pso_356035; Pso_288906; Pso_358111; Pso_292791; Pso_288815	PITG_13093T0	Secreted RxLR effector peptide protein, putative
9	Pca_14853; Pca_15117; Pca_19651	PITG_12276T0; PITG_11839T0	Prm_76339	Pso_288968	PITG_12276T0	Secreted RxLR effector peptide protein, putative
10	Pca_538116; Pca_97196	PITG_07556T0; PITG_07558T0	Prm_77948; Prm_77945	Pso_353461	PITG_07556T0	Secreted RxLR effector peptide protein, putative
11	Pca_548556	PITG_12952T0; PITG_10654T0; PITG_02900T0	Prm_80526	Pso_284479	PITG_12952T0	Secreted RxLR effector peptide protein, putative
12	Pca_20942	PITG_18986T0	Prm_76324	Pso_286791; Pso_286793; Pso_286162	PITG_18986T0	Secreted RxLR effector peptide protein, putative
13	Pca_119793	PITG_15032T0	Prm_78009	Pso_286223; Pso_286248; Pso_286221	PITG_15032T0	Secreted RxLR effector peptide protein, putative
14	Pca_118417; Pca_124413	PITG_06087T0	Prm_81609	Pso_286934	PITG_06087T0	Secreted RxLR effector peptide protein, putative
15	Pca_124376	PITG_06099T0; PITG_06094T0	Prm_81610	Pso_286931	PITG_06099T0	Secreted RxLR effector peptide protein, putative
16	Pca_116645	PITG_18405T0; PITG_10640T0	Prm_81911	Pso_284377	PITG_18405T0	Secreted RxLR effector peptide protein, putative
17	Pca_101904	PITG_15226T0; PITG_15225T0	Prm_83274	Pso_285899	PITG_15226T0	Secreted RxLR effector peptide protein, putative
18	Pca_4454	PITG_10116T0	Prm_74395; Prm_74378	Pso_288795	PITG_10116T0	Secreted RxLR effector peptide protein, putative
19	Pca_549194	PITG_18397T0; PITG_18117T0	Prm_81902	Pso_476203	PITG_18397T0	Putative uncharacterized protein
20	Pca_101012	PITG_04099T0	Prm_85073	Pso_286058	PITG_04099T0	Secreted RxLR effector peptide protein, putative
21	Pca_101423	PITG_09585T0	Prm_75817	Pso_361266	PITG_09585T0	Secreted RxLR effector peptide protein, putative
22	Pca_129643	PITG_15287T0	Prm_78400	Pso_286050	PITG_15287T0	PexRD1 secreted RxLR effector peptide, putative
23	Pca_19601	PITG_11947T0	Prm_78163	Pso_246483	PITG_11947T0	Secreted RxLR effector peptide protein, putative
24	Pca_508923	PITG_04668T0	Prm_84933	Pso_533029	PITG_04668T0	Polysaccharide lyase, putative
25	Pca_536039	PITG_09824T0	Prm_77012	Pso_329838	PITG_09824T0	Metalloprotease family M12A, putative
26	Pca_546134	PITG_13256T0	Prm_83882	Pso_354514	PITG_13256T0	Putative uncharacterized protein
27	Pca_558196	PITG_13007T0	Prm_76705	Pso_520326	PITG_13007T0	Putative uncharacterized protein
28	Pca_129113	PITG_15142T0	Prm_85377	Pso_286249	PITG_15142T0	Secreted RxLR effector peptide protein, putative

### Expression profiling of genes encoding RxLR and CRN-like proteins

In order to obtain insight into effector expression during infection, RNA-Seq analysis of samples corresponding to newly formed spores, and early and late stages of infection on cotyledons was performed. Genes encoding CRNs and RxLR effectors were expressed at all three stages. Some genes were up-regulated during the early stage of infection (102 putative RxLR effectors and 15 CRNs; Additional file 2: Figure S27A and S27C, Additional file 25), while others were up-regulated in spores and late stages (181 putative RxLR effectors and 34 CRNs; Additional file 2: Figure S27b and S27D, Additional file 26). Taken together, these results suggest that stage-specific sets of CRN and RxLR effector candidates are expressed during the infection of sunflower by *Pl. halstedii*.

## Discussion

### Genome features

The assembled genome *Pl. halstedii* has a size of 75.3 Mb. This size is similar to that of the other sequenced downy mildew pathogen, *Hy. arabidopsidis*, which has an assembled genome size of 78.9 Mb. According to a recent study [4], the estimated genome size of *Pl. halstedii* is 100 Mb, which is comparable to that of *Hy. arabidopsidis* [6]*. Phytophthora* spp. assemblies range from 64 Mb for *Ph. capsici* to more than 200 Mb for *Ph. infestans* and other clade 1c species [58]. The significantly bigger genome of *Ph. infestans* compared to other oomycetes is due to repeat-driven genome expansion, with a repeat element content of 74 % [12]. In contrast only 39 % of the *Pl. halstedii* genome was comprised of repeat elements, comparable to the 43 % reported for *Hy. arabidopsidis* [6]. The N_50_ scaffold size for the genome assembly of *Pl. halstedii* is 1.54 Mb, which reflects its high quality of genome. Genome completeness with respect to 248 core eukaryotic genes is similar in the eight deeply sequenced oomycete genomes, reflecting an adequate coverage of the gene space.

To study the genome architecture of *Pl. halstedii*, 5′ and 3′ flanking distances of all genes were calculated. These studies revealed that CRN, RxLR and other PSEP-encoding genes were localized in more gene-sparse regions than non-PSEP-encoding genes, particularly than the core housekeeping genes. This situation is similar to other genomes of other oomycete pathogens [3, 12].

### Phylogenetic analyses

Unexpectedly, the two downy mildew species included in the analysis, *Pl. halstedii* and *Hy. Arabidopsidis,* did not show a sister group relationship (Fig. 3). While *Hy. arabidopsidis* was placed outside of the clade containing *Phytophthora* species, as inferred by some earlier studies [23, 59], *Pl. halstedii* was found to be nested within *Phytophthora*, rendering the genus paraphyletic. Reasons for the disjunction of the downy mildew species could be either that downy mildews are a polyphyletic assemblage or that the limited taxon sampling, in conjunction with the comparatively high mutation rates in downy mildews, leads to an incorrect estimate of phylogenetic relationships. Both multigene analyses with a larger taxon sampling [22] and morphological data [21] suggest the nesting of a potentially monophyletic downy mildew clade within a paraphyletic genus *Phytophthora*. It seems likely that the question of downy mildew monophyly cannot be resolved until representatives from all major clades of downy mildews and *Phytophthora*, especially of *Phytophthora* clades inferred to be closely related to downy mildews [22], have been sequenced.

### Heterozygosity

Within both downy mildews and *Phytophthora* spp., homothalism has evolved several times [60], and might be an adaptation towards ensuring sexual reproduction and the formation of durable resting spores even when hosts plants are sparsely distributed. As a consequence of homothalism of *Pl. halstedii* [45], rates of sexual recombination between two independent strains are expected to be very low, resulting in pathogen strains with extremely low heterozygosity, similar to the situation in *Ph. lateralis* [13]. This is supported by the present study, which reports only 0.0012 % of sites with a major allele frequency of 0.45 to 0.55. As only a few sunflower genotypes are grown commercially, selfing after rare events of outcrossing or parasexual recombination [61] might lead to abundant pathogen genotypes adapted to a variety of sunflower genotypes lacking the R-genes that would match their effector complement [62, 63].

### Promoter analyses

How effectors and other genes are regulated in oomycetes is still very unclear and motif identification apart from a few basic patterns proved to be difficult. Some motifs have been described with respect to general oomycete promoter structure [47, 49], and only a few stage-specific transcription factor binding sites have been identified [64], so far.

### Phospholipid signaling

*Pl. halstedii* harbours a set of PMSEs that is well conserved in respect to the ones identified in other oomycetes. A slight reduction in numbers is observed in PIKs whereas a large reduction is found in the number of PLDs. The latter resulted in the absence of one class of oomycete specific PLDs, the TM-PLD. Among the strongly conserved PMSE are the GKs. In *Phytophthora* spp. Twelve GKs are encoded, suggestive of a unique signaling pathway that bypasses G-protein mediated signaling by direct PIPK activation [65]. So far it is unclear, which role these receptors play in pathogenicity, but it has been speculated that they are important for sensing the host environment [27]. However, experimental support is necessary for testing this hypothesis.

### Secondary metabolites and phytohormones

Secondary metabolites, and phytohormones in particular, are important virulence determinants in several bacterial and fungal pathogens, e.g. in *Xenorhabdus* spp. [66], *Ustilago maydis* [67] and *Fusarium oxysporum* [68]. However, while some studies have examined the primary metabolites of oomycetes, in particular fatty acids [69, 70], little is currently known about the capability of oomycetes to produce secondary metabolites. The identification of few NRPS and PKS genes in *Pl. halstedii* and other oomycetes is suggestive of the production of only few secondary metabolites; however, it has been reported that some fungal PKS can produce a variety of different compounds [71]. Monomodular NRPS composed of A-T-TE or A-T-Red domains are responsible for the biosynthesis of quinone [72] or pyrazine structures [73] composed of two amino or 2-ketocarboxylic acids. As these compounds often show biological activity [72], compounds derived from these enzymes in *Pl. halstedii* might act as virulence factors or protect the host environment against competitors and might thus be promising targets for future research.

*Plasmopara halstedii* seems to lack the capacity to produce most phytohormone classes, with the exception of cytokines and brassinolides. Even though these two classes of phytohormones might contribute to the disease phenotype, it has been shown in previous studies that the stunting effects in systemically-infected sunflowers are likely to be mostly auxin-mediated [74] Depletion of IAA by IAA oxidase activity [75] is a hallmark of this process, but it remains unclear how the pathogen interferes with the host to incite this remarkable disturbance of phytohormone signaling.

### Candidate effectors

All pathogenic and endosymbiotic oomycete and fungal species secrete a plethora of proteins into their hosts to manipulate host defence reactions and to enable the formation of an interface for plant-microbe interaction in (hemi-) biotrophic species. Most genes encoding secreted effectors are fast-evolving and show limited sequence conservation, even though some effectors have been reported to be conserved among species within a certain pathogen group [6, 13, 76]. Positive selection studies in both oomycete and fungal plant pathogens have shown that effector-encoding genes are under higher selection pressure than non-effector genes [3, 28, 30, 58, 76].

### NLPs

High numbers of NLP genes have been found in the genomes of *Phytophthora* species [11, 12, 77]. The members of this family that induce host cell death have been suggested to function during their switch from biotrophy to necrotrophy [78, 79]. It was thus unexpected that while most branches of the NLP family were not present one clade of NLPs was expanded in *Hy. arabidopsidis* [6]. However, no evidence could be found with respect to an induction of necrosis by any downy mildew NLP tested, suggesting an alternative role during disease development [6]. Similar to the genome of *Hy. arabidopsidis*, most branches of the NLP family were absent in *Pl. halstedii*, but twice as many branches of NLPs were retained. Two of these have expanded into small protein subfamilies independent from *Hy. arabidopsidis*. Several of these NLPs were found upregulated during infection as compared to spores, highlighting their potential importance for pathogenicity in this biotrophic pathogen.

### Crinkler-like proteins

CRN proteins are an ancient class of proteins that has been reported to contain effectors targeted to the nucleus of the host cell [80]. Genes encoding CRNs have been found in the genomes of all sequenced oomycete plant pathogens and are especially abundant in *Phytophthora* species [12]. In the genome of *Pl. halstedii* 77 CRN-like proteins were predicted, an amount similar to *Hy. arabidopsidis*. Surprisingly, 75 out of 77 proteins with a CRN signature in *Pl. halstedii* were not predicted to contain a classical secretion signal, and the function of these proteins remains enigmatic. It is also noteworthy that while a few other pathogenicity-related genes, such as three proteases, an NLP, two proteinase inhibitors, and 26 RxLR-like proteins had orthologs in all Peronosporaceae, no universally conserved secreted CRN-like gene was identified. However, orthology of 32 CRNs of *Pl. halstedii* with at least one oomycete species was revealed. The function of these conserved CRNs in pathogenicity or cellular processes within the pathogen remains to be tested.

### RxLR-like proteins

A hallmark of a large fraction of cytoplasmic effectors in downy mildew and *Phytophthora* species is the presence of a N-terminal RxLR-dEER motif [81–83]. While these effectors seem to be abundant in the crown lineages of Peronosporaceae, canonical RxLR effectors are apparently absent in the genomes of two necrotrophic pathogens, *Py. ultimum* of the Pythiaceae and the oomycete fish pathogen *Sa. Parasitica* of the Saprolegniaceae. However, the presence of possibly convergently evolved RxLR-like effectors in the genome of *Sa. parasitica* and their translocation into the fish cells has been demonstrated [84]. Even though *Albugo* species seem to contain a few secreted proteins with RxLR-like motifs [3, 7], their function is currently unclear. It seems possible that they have evolved independently in the Albuginaceae, which represent an ancient biotrophic lineage [1, 85]. In the genome of *Pl. halstedii* a total of 274 RxLR-like proteins coding genes were predicted, out of which only 34 were having the RxLR-dEER motif typical for *Phytophthora* species. Interestingly, the genome of *Pl. halstedii* also encodes many variants of RxLR-like effectors (Fig. 7b), including putative KxLR-dEER and RxLK-dEER effectors. A few proteins with a QxLR motif, previously reported from *Pseudoperonospora cubensis* [18], were also observed, but none of these had a predicted dEER motif. This highlights that while in *Phytophthora* species the majority of cytoplasmic effectors of the RxLR-type seem to have a highly conserved RxLR-motif, conservation is much lower in downy mildews, which seem to have evolved a huge variety of motifs, rendering their identification less straightforward. However, the dEER motif seems to be of much higher conservation. In *Pl. halstedii* a significant number of secreted, dEER-motif containing proteins were predicted, which showed variant RxLR motifs, or which, similar to the effector ATR5 from *Hy. arabidopsidis* [86] did not show any recognisable RxLR-motif. This raises doubts regarding the importance of the biochemical features of the RxLR-motif for protein translocation into host cells, in line with recent findings of Yaeno and Shirasu [87], and might be suggestive of a combined action of RxLR-like and dEER-like motif, either host-independent [88] or involving additional proteins.

### Conservation of PSEPs within downy mildews and *Phytophthora* species

Orthology analyses were conducted for the PSEPs of *Phytophthora* and downy mildew pathogens. In total there were 65 orthologs predicted among *Phytophthora* and downy mildew genomes, in terms of PSEP-encoding genes. Functional annotations of these orthologs suggest that they contain peptide bond-degrading enzymes, NLPs, proteinase inhibitors and RxLR-like proteins. These genes might represent a core set of effectors which is required for infection. No secreted CRN was found in the set of 65 PSEP orthologs, which was expected, as these analyses were conducted only on the PSEP-encoding genes. However, we could identify six orthologs of high confidence RxLR-like protein encoding genes shared by the downy mildew pathogens *Hy. arabidopsidis* and *Pl. halstedii*. Similarly, 28 orthologs of such candidate RxLR-like proteins were predicted among the four *Phytophthora* species. This suggests the presence of a conserved core set of candidate RxLR-like effectors, which potentially target basic hubs in plant defence pathways, similar to the PEP1 effector, which remained conserved in smut genomes [76, 89] and acts as an inhibitor of conserved plant peroxidases. It is notable that only the RxL part of the RxLR seems to be highly conserved within potential core effectors of *Phytophthora* and downy mildews, similar to the situation in *Plasmodium* [90], in which the same residues seem to play a vital role for delivering its effectors to the host cytoplasm [90–92].

## Conclusion

Overall it was found that the assembled genome of *Pl. halstedii* is extremely low in heterozygosity, presumably due to its homothallism, and shows a similar amount of genome completeness in terms of CEGMA genes like other deeply-sequenced oomycete genomes. It is noteworthy that the genomes of obligate biotrophic oomycetes are apparently more AT-rich than those of related species with other lifestyles. Interestingly, phylogenomic analyses seem to refute downy mildew monophyly, although it cannot be ruled out at present that this is an artefact from low taxon sampling. However, the high degree of distinctiveness and independent evolution is supported by orthology analyses, which revealed a higher degree of gene conservation among *Phytophthora* species than downy mildews. Core promoter structures in oomycetes remain obscure, as apart from the CCAAT-box, no additional conserved motifs could be identified. Notably *Pl. halstedii* seems to have the capacity to produce phytohormones of the classes brassinolids and cytokines. However, it remains to be tested in future studies, whether the production of phytohormones by *Pl. halstedii* is important for its pathogenicity. Its genome codes for some secondary metabolite clusters, including an unusual NRPS with both TE and Red domains, but their products and their role in pathogenicity have yet to be identified. Conservation of candidate effector protein encoding genes in the genome of *Phytophthora* and downy mildew pathogens was observed, suggesting a core set of effector proteins that might play a key role in the pathogenicity of these pathogens. However, while 183 PSEP-encoding orthologs were present in *Phytophthora* spp. genomes but absent from downy mildews, only 11 PSEPs were present in downy mildews but absent from *Phytophthora*. Functional annotations of the predicted gene models revealed several variants of the RxLR motif, while the RxL part of RxLR motif within *Phytophthora* and downy mildew pathogen genomes was mostly conserved. In addition, several proteins in which only the dEER motif is conserved were identified. Taking into consideration the additional variation of the RxLR-motif found in this study, this is suggestive of a major role of the dEER motif in the function of the RxLR-dEER-like effectors.

## Methods

### Genome assemblies, repeat elements masking and gene predictions

In total four genomic DNA libraries were sequenced from the DNA extracted from *Plasmopara halstedii* zoosporocysts (methods used for DNA isolation and extraction have been described in Additional file 1). Adapter and primer sequences from reads were removed using Trimmomatic [93]. Filtering parameters estimation and filtering was done using the FastQFS tool [94]. All genomic reads were filtered using an average read quality threshold of 26 phred score and a length cut-off of 72 bp. Bacterial or other contamination in the sequenced reads were removed from the raw reads prior to the analyses (Additional file 1). Genome assemblies were performed using the Velvet v1.2.10 [41] genome assembler. The best assembly was determined by comparing the N_50_ scaffold size, the largest scaffold size, the number of scaffolds, the percentage of reads mapped to the assembled genome and CEGMA [42] genome completeness analysis. A k-mer of length 59 generated the best genome assembly considering above mentioned assembly quality parameters. The resulting set of assembled scaffolds were scanned for repeat elements (Additional file 1). Gene predictions on the masked genome were performed by both transcript mapping based and *ab-initio* based methods (Additional file 1) as described before [95]. The resulting consensus gene set was subjected to expression profiling by using the three RNA-Seq libraries (Additional file 1). A plot representing the length distribution of protein sequences (Additional file 2: Figure S28) was generated using the hist() function of R [96]. Methods used for the investigation of heterozygosity levels and for the development of SSR markers have been described in the Additional file 1.

Functional annotations of the predicted genes were performed by using the Blast2GO software [97] (Additional file 1). Putative secreted effector protein (PSEP)-encoding genes were predicted using SignalP [98] along with other tools (Additional file 1). Methods used for the functional annotation of secondary metabolite synthase producing genes, genes involved in certain pathways, phospholipid modifying and signaling enzymes (PMSE)-encoding genes, NLPs, protease inhibitors, CRNs and RxLR-like protein encoding genes have been described in Additional file 1.

### Orthology analyses

Orthology analyses were done using the OrthoMCL [99] software, using an e-value cut-off of e^−5^ and a 50 % identity cut-off to define orthologous proteins among nine oomycete genomes. Subsequently perl and shell scripts were used to extract the 1:1 orthologs. Orthologs in terms of PSEPs were identified by running OrthoMCL on all predicted PSEPs encoded by the nine genomes using same input parameters.

### Phylogenetic analysis

A phylogenetic analysis was conducted on the core ortholog genes predicted using the CEGMA pipeline [42]. Multiple sequence alignments were performed using MAFFT [100] and subsequently concatenated. Maximum likelihood phylogenetic analysis was done with RAxML [101], with 1000 bootstrap replicates and default settings.

### Promoter analyses

To study the promoter architecture of *Pl. halstedii*, intergenic regions were extracted from the scaffolds. Only scaffolds which contained at least 100 genes were used, to avoid bias due to gaps in the assembly. Sequences upstream of genes were extracted and A + T contents were calculated. To measure the frequency of incidence of the 7-nt Initiator (INR), 7-nt FPR, 7-nt DPEP and 16-nt INR + FPR supra-motif elements in *Pl. halstedii,* position-specific probability matrices (PSPMs) corresponding to the motifs in *Ph. infestans* were used to scan 400 nucleotides of DNA upstream of 5000 *Pl. halstedii* genes, and then PSPMs corresponding to the *Pl. halstedii* matches were used to rescan the *Pl. halstedii* promoters. Control searches were performed against databases of twice-shuffled promoter DNA. The genome of *Pl. halstedii* was also searched for other known *Phytophthora* TFBSs, using Fisher’s Exact Test with a *P* < 0.01 threshold to test the significance of any hits, compared to searches of shuffled promoter sequences. MEME [48] was used to search for novel motifs in the *Pl. halstedii* promoter sequences.

### Data Access

Genomic data files have been uploaded to a local server dx.doi.org/10.12761/SGN.2015.7. The study has been registered in the ENA database under the study accession number PRJEB6932. Raw genomic reads and RNA-Seq reads have been uploaded to the ENA database and are available via the same study accession numbers. Genomic sequence reads from all four sequenced libraries are available via accession numbers ERR583679 to ERR583682 and the three RNA-seq libraries are deposited under the accession numbers ERR583683 to ERR583685. Genome assembly scaffolds and annotation were deposited under the accessions CCYD01000001-CCYD01003162.

## Additional files

Additional file 1:
**Additional Material and Methods section.** (DOCX 128 kb) Additional file 2:
**Supplementary figures.** (PDF 34119 kb) Additional file 3:
**Simple sequence repeats within the genome of** ***Plasmophara halstedii*** **.** (XLSX 26 kb) Additional file 4:
**Statistics of repetitive motifs in** ***Pl. halstedii*** **genome.** (DOCX 38 kb) Additional file 5:
**Repeat motifs and their frequencies.** (DOCX 57 kb) Additional file 6:
**Five candidate orthologs among the nine genomes.** (XLSX 40 kb) Additional file 7:
**77 candidate orthologs among the five genomes.** (XLSX 36 kb) Additional file 8:
**65 candidate orthologs among the six genomes.** (XLSX 42 kb) Additional file 9:
**11 orthologs among the downy mildew genomes.** (XLSX 40 kb) Additional file 10:
**PMSE genes among the oomycete genomes.** (DOCX 50 kb) Additional file 11:
**PMSE gene ids of** ***Pl. halstedii.*** (XLSX 12 kb) Additional file 12:
**Potential capacity of biosynthesis of plant hormones in** ***Pl. halstedii.*** (DOCX 40 kb) Additional file 13:
**19 candidate NLPs of** ***Pl. halstedii.*** (XLSX 58 kb) Additional file 14:
**Properties of candidate NLPs of** ***Pl. halstedii.*** (DOCX 72 kb) Additional file 15:
**Protease inhibitors effector families predicted in the genome of** ***Plasmopara halstedii.*** (DOCX 95 kb) Additional file 16:
**Kazal-like domain features among the different genomes.** (XLSX 47 kb) Additional file 17:
**Cystatin-like domain features among the different genomes**. (XLSX 44 kb) Additional file 18:
**Candidate CRNs of** ***Pl. halstedii,*** **predictions based on gene models.** (XLSX 46 kb) Additional file 19:
**Candidate CRNs of** ***Pl. halstedii***, **predictions based on ORFs generated from the un-masked genome.** (XLSX 38 kb) Additional file 20:
**Candidate RxLR-like effectors of** ***Pl. halstedii***, **predictions based on gene models.** (XLSX 98 kb) Additional file 21:
**Candidate RxLR-like effectors of** ***Pl. halstedii***, **predictions based on ORFs generated from the un-masked genome.** (XLSX 11 kb) Additional file 22:
**Proteins with positive score for WY-fold hidden Markov model.** (XLSX 13 kb) Additional file 23:
**Six orthologs of candidate RxLR-like proteins among the downy mildew genomes.** (XLSX 34 kb) Additional file 24:
**Three orthologs of candidate RxLR-like proteins among the downy mildew and** ***Phytophthora spp.*** **genomes.** (XLSX 32 kb) Additional file 25:
**Gene expression values of up-regulated candidate RxLR-like proteins and CRNs of** ***Pl. halstedii.*** (XLSX 14 kb) Additional file 26:
**Gene expression values of down-regulated candidate RxLR-like proteins and CRNs of** ***Pl. halstedii.*** (XLSX 65 kb)

## Abbreviations

CEGMA: Core eukaryotic gene mapping approach
CRN: Crinkler
DPE: Downstream promoter element
EPICs: Cystatin-like cysteine protease inhibitors
EPIs: Kazal-like serine protease inhibitors
FPR: Flanking promoter region
GKs: G-protein coupled receptor (GPCR)-phosphatidylinositol phosphate kinase (PIPK)
GPCR: G-protein coupled receptor
HMM: Hidden markov model
INR: Initiator
Kb: Kilobase
LINE: Long interspersed elements
Mb: Megabase
NCBI: National center for biotechnology information
NLPs: Necrosis and ethylene-inducing peptide 1(Nep1)-like proteins
NRPS: Nonribosomal peptide synthetases
NeP1: Necrosis and ethylene-inducing peptide 1
ORF: Open reading frame
PIK: Phosphatidylinositol kinase
PIPK: Phosphatidylinositol phosphate kinase
PLC: Phospholipase C
PLD: Phospholipase Ds
PMSE: Phospholipid modifying and signalling enzymes
PSEP: Putative secreted effector protein
QC: Quality control
RAxML: Randomized axelerated maximum likelihood
SSR: Simple sequence repeats
TFBSs: Transcription factor binding sites
TM-PLD: Trans-membrane domains-PLD
