# Supplementary material for: Genome analyses of the sunflower pathogen Plasmopara halstedii provide insights into effector evolution in downy mildews and Phytophthora
Source: BMC Genomics. 2015 Oct 5;16:741. doi: 10.1186/s12864-015-1904-7 (PMC4594904; doi:10.1186/s12864-015-1904-7)
Supplement: Additional file 1: — Additional Material and Methods section. (DOCX 128 kb) [file 12864_2015_1904_MOESM1_ESM.docx]

**Supplementary File 1 – Supplementary Material and Methods**

**Plant and oomycete material**

Sunflower plants from the *Helianthus annuus* cultivar ‘Giganteus’ were grown in a climate chamber at 22°C with 55% humidity and 16 h light per day. Sunflower plants 4-6 days old were infected with *Plasmopara halstedii* (single zoospore strain OS-Ph8-99-BlA4) by whole seedling inoculation with a suspensions of freshly harvested zoosporocysts (1-3 x 10^5^ per ml) for 2 h at 16°C. Infected cotyledons were collected 12 days post inoculation (dpi), were rinsed thoroughly in 2% NaClO, washed with sterile water, and sporulation was induced by incubating the cotyledons in darkness with 100% humidity at 16°C. After 4-6 h zoosporocystophores appeared on the cotyledon surface.

**DNA extraction**

*Plasmopara halstedii* zoosporocysts were harvested by rinsing sporulating cotyledons with sterile water and pelleted by centrifugation. The genomic DNA was isolated as described previously [[1](#_ENREF_1)] with minor modifications. In brief, sporangium pellets were resuspended in a lysis buffer (50mM Tris pH 8.0, 200 mM NaCl, 0.2 mM EDTA, 0.5% SDS, 100 mg/ml Proteinase K) and vortexed with glass beads for 15 min. After incubation for 30 min at 37°C, RNase A was added followed by another 15 min incubation. Then the lysate was mixed with phenol and chloroform. After centrifugation (19000g, 2 min) and precipitation with 100% ethanol, the DNA pellet was washed twice with 70% ethanol. Finally the dried DNA pellet was dissolved in TE buffer. The DNA quantity and quality was determined by spectrometry as well as estimated by TBE gel electrophoresis.

**RNA extraction**

Uninfected sunflower cotyledons were incubated within a zoosporocyst suspension (10^5^ zoosporocysts/ml) for one hour in darkness. After this time, some of the cotyledons were taken out and frozen immediately. The rest of the cotyledons were taken out as well, placed on wet filter papers in Petri dishes and incubated in the darkness for an additional 3 h and one day at 16°C, respectively. Furthermore, sunflower cotyledons 12 dpi were harvested and incubated in five individual Petri dishes with soaked paper for 1, 3, 6, 12 and 24h. At the time point of 24h incubation, the zoosporocysts on the cotyledons were rinsed off. All of these treatments were directly used for RNA isolation. RNA was extracted by using the NucleoSpin® RNA Plant kit (MACHEREY-NAGEL GmbH & Co. KG.) The RNA quality was controlled by spectrometry as well as being determined on a 1.5% agarose gel stained with ethidium bromide.

**Library preparation and sequencing**

Two paired-end shotgun libraries (300 kb and 800 kb insert sizes), two mate-pair libraries (8 kb and 20 kb insert sizes), and three RNA-Seq libraries corresponding to early stages of infection (1 h, 4h, and 24 h post infection), late stages of infection (the different time points after the induction of sporulation), and pelleted zoosporocysts were produced by MWG Eurofins (Germany). Sequencing was done on an Illumina HiSeq 2000 sequencer with 100 bp read length by the same company.

**Contamination filtering**

An initial assembly was tested for contamination by bacteria or other organisms. For this all scaffolds from the initial assembly were aligned to the NCBI NT database (latest available) locally (ftp://ftp.ncbi.nlm.nih.gov/blast/db/) using standalone Blast v2.2.28+ [[2](#_ENREF_2)]. A database of all possible contaminants was generated and Bowtie2 [[3](#_ENREF_3)] was used to map the raw reads onto this database. All reads not mapping to potential contaminants were again used for Velvet assemblies using several k-mer lengths and k-mer coverage cut-offs.

**Repeat element masking**

Repeat elements were masked using RepeatModeler (http://www.repeatmasker.org/RepeatModeler.html). RECON [[4](#_ENREF_4)] and RepeatScout v1 [[5](#_ENREF_5)] were used to perform *de-novo* repeat element prediction. Repbase library version 20130422 [[6](#_ENREF_6)] was imported to RepeatModeler for reference-based repeat element searches. Tandem repeat finder (trf) [[7](#_ENREF_7)] was used inside the RepeatModeler pipeline for generating a set of tandem repeats. The final set of predicted repeat elements were then masked in the genome assembly using RepeatMasker (http://www.repeatmasker.org/).

**Gene prediction**

Gene predictions were done using both *ab-initio* and transcript-guided gene prediction tools. Transcripts were generated by first mapping the RNA-Seq reads to the assembled genome by using TopHat2 [[8](#_ENREF_8)]. Using this mapping information Cufflinks [[9](#_ENREF_9)] generated a set of transcripts. GeneMark-ES [[10](#_ENREF_10)] was used to generate an initial set of gene models. Using Augustus [[11](#_ENREF_11)] another set of gene models was generated for which the highly confident gene set generated from GeneMark-ES was used as training set (Supplementary Figure 1). The sam mapping file generated byTopHat2 was used by Augustus as an intron/exon hint file.

Alignments of transcripts generated by Tophat2 were done using PASA [[12](#_ENREF_12)] and Gmap [[13](#_ENREF_13)]. The gene sets from GeneMark-ES and Augustus, as well as transcript alignments from PASA and Gmap were imported to the EvidenceModeler [[14](#_ENREF_14)] package for consensus gene model predictions. Higher weight was given to the RNA-Seq alignment predictions than to *ab-initio* based predictions. RNA-Seq mapping was repeated on the gene-masked and repeat-masked genome and from this the set of gene models was complemented using Transdecoder (<http://transdecoder.sourceforge.net/>). Only those genes were considered further which were having a length equal to or more than 150 nt.

**Functional annotations**

Functional annotations of the generated genes were done using Blast2GO [[15](#_ENREF_15)]. KOG [[16](#_ENREF_16)] mapping was done locally by using BlastP [[2](#_ENREF_2)] with an e-value cut-off of e^-5^. Gene ontology (GO) [[17](#_ENREF_17)] and InterPro [[18](#_ENREF_18)] ids were assigned using Blast2GO tool. Pfam [[19](#_ENREF_19)] protein family analysis was also done locally using an e-value cut-off of e^-3^. Protein clustering was performed by using SCPS [[20](#_ENREF_20)] with the TribeMCL [[21](#_ENREF_21)] clustering algorithm. KEGG [[22](#_ENREF_22)] analyses were done by using the KAAS [[23](#_ENREF_23)] online webserver and enzyme commission (EC) numbers were assigned using perl scripts. Protein family analyses were done by using the standalone Panther protein family mapping tool pantherScore v1.03, with the PANTHER database v9 [[24](#_ENREF_24)].

**Heterozygosity**

The genome was surveyed for heterozygosity based on alignments of genomic sequence reads against the repeat-masked *Pl. halstedii* reference genome assembly. The alignment was performed using the mem algorithm of BWA version 0.7.5a [[25](#_ENREF_25), [26](#_ENREF_26)] with default settings. Then the alignment was converted into the pileup format using SAMtools [[27](#_ENREF_27)]. Sequence reads that could match equally well to multiple genomic locations were deleted by using the ‘-q 1’ option in the SAMtools view function. This step was necessary in order to avoid false heterozygosity inference from alignment artifacts resulting from sequence reads originating from genomic repeats or paralogs. From the SAMtools pileup file, Perl scripts were used to examine each nucleotide site in the alignment and perform a census of the aligned nucleotides at that site. If all aligned sequence reads were in complete consensus, the proportion of the major allele was considered to be 1. If any sequence reads disagreed with the consensus at that site, then we calculated the proportion of reads that agreed with the most frequent nucleotide at that site (i.e. the major allele). Heterozygous sites would be expected to generate a major-allele-frequency proportion close to 0.5 whilst homozygous sites would fall close to 1; therefore, in a diploid genome with significant levels of heterozygosity, a bimodal frequency distribution with peaks close to 0.5 and close to 1 would be expected. Frequency distributions were visualized as a histogram using the hist() function in R [[28](#_ENREF_28)].

**SSR marker development**

A total of 19 mitochondrial and 3162 nuclear scaffolds were screened for di-, tri-, tetra-, penta-, and hexanucleotide repeats using the program Msatcommander 0.8.2 [[29](#_ENREF_29)], with minimum repeats set to 10, 7, 6, 5, and 4, respectively. All other parameters were kept at their default values. Primers were designed using the Msatcommander 0.8.2 workflow, which includes Primer3 [[30](#_ENREF_30)]. All predicted primer pairs were checked if they border a given SSR array using the output files from Msatcommander and GMATo (Genome-wide Microsatellite Analyzing Tool) [[31](#_ENREF_31)]. False predictions were corrected using Primer3web 4.0.0 [[32](#_ENREF_32), [33](#_ENREF_33)] and primer positions in the original scaffold were checked using Mega 6.06 [[34](#_ENREF_34)]. Additional markers were designed in Primer3web 4.0.0, after selecting SSR arrays with a high number of repetitions detected by GMATo (a minimum of 10 repeats for all screened motives in nuclear scaffolds and a minimum of 6 dinucleotide repeats in mitochondrial scaffolds). Statistical analyses of repetitive motifs in the mitochondrial and the nuclear genome were performed using GMATo.

**Secretome prediction**

Protein sequences with extracellular secretion signals were predicted using SignalP v2 [[35](#_ENREF_35)]. Proteins were considered to be secreted if the signal peptide probability was more than or equal to 0.90 and a cleavage site was within first 40 amino acids. These predictions were further refined using TargetP v1 [[36](#_ENREF_36)], and candidate secreted proteins predicted to be targeted to mitochondria were discarded. Subsequently, these candidate secreted proteins were checked for trans-membrane domains using TMHMM [[37](#_ENREF_37)]. Only those candidate secreted proteins were considered as putative secreted effector proteins (PSEPs) that were having at most one predicted trans-membrane domain.

**Prediction of secondary metabolite producing genes and metabolic pathways**

Genes for secondary metabolite production were annotated using the antismash software package [[38](#_ENREF_38), [39](#_ENREF_39)]. To identify biochemical pathways in *Pl. halstedii*, InterProScan in combination with KEGG maps was used to get an overview of potentially present or absent secondary pathways. Once pathways had been identified, proteins of interest crucial for those pathways were again analysed using NCBI BlastP and hits were manually curated. In case enzymes were not identified by InterProScan in pathways of interest, genes were downloaded from TAIR and NCBI and tBlastn searches were carried out to confirm their absence or to identify missed or wrongly annotated gene models. According to this manual annotation, gene models were curated and candidates were re-analysed using InterProScan and again blasted to NCBI. An e-value cut-off was set at e^-4^ and all alignments were manually inspected.

As Cytochrome P450 enzymes are difficult to characterize on a computational level, the fungal Cytochrome P450 Database was used in two-way blast searches (http://p450.riceblast.snu.ac.kr).

**Phospholipid analyses**

The genome of *Pl. halstedii* was screened for the homologs of phospholipid modifying and signaling enzymes (PMSE) encoding genes that are present in other oomycetes genomes. A database of *Ph. infestans* PMSE proteins was created and both BlastP and tBlastn searches were performed with an e-value cutoff of e^-20^. Alignments were manually inspected and PMSE-encoding gene homologs were assigned in the genome of *Pl. halstedii*. To illustrate their phylogeny, *PhPIPKD9* was integrated in a phylogenetic tree with all *GKs* from five representatives oomycetes: *Hy. arabidopsidis*, *Ph. infestans*, *Ph. ramorum*, *Ph. sojae*, *Py. ultimum*, and the single non-oomycete *GK* from *Dictostelium discoidum* (*DdRpkA*). Multiple sequence alignments were performed by using Mafft [[40](#_ENREF_40)]. Phylogenetic analyses were performed by using RAxML [[41](#_ENREF_41)] with 1000 bootstrap replicates. Alignment of *PhPIPKD9* with other GK9s were done using Mafft and alignments graphics were generated using Jalview [[42](#_ENREF_42)].

**NLPs**

Homologues of NLPs in the genome of *Pl. halstedii* were predicted using BlastP with the *Ph. sojae* NLP proteins. InterPro and Pfam domain information was also used to further confirm these predictions. Signal peptides were removed before multiple sequence alignments in MEGA5 [[43](#_ENREF_43)], using default settings. Phylogenetic analyses were performed using the Neighbour Joining algorithm as implemented in MEGA5 [[43](#_ENREF_43)], with 100 bootstrap replicates. All non *Pl. halstedii* NLPs were taken from [[44](#_ENREF_44)]. The genome of *Pl. halstedii* was also scanned for pseudogenes of NLPs. A database of predicted *Pl. halstedii* NLPs was created by removing the signal peptide and additional domains (Q-rich region, Jacalin-like domain). Pseudogenes were searched in the repeat masked genome by using tBlastn and Ugene (http://ugene.unipro.ru/) [[45](#_ENREF_45)]. Nucleotide sequences were extracted from the repeat-masked nuclear genome sequence using the hit location information provided by the output of tBlastn. All sequences longer than 500 nt were used to build a phylogenetic tree, together with the DNA sequences of the predicted *Pl. halstedii* candidate NLPs. The sequences from tBlastn searches with a premature stop-codon in the corresponding *NLP* gene were further analysed to fully reconstruct the pseudogenes.

**Protease inhibitors**

To find putative sequences with similarity to known effectors in the oomycete plant pathogen *Ph. infestans* blast searches were carried out with low complexity filters using BLAST version 2.2.25+ [[46](#_ENREF_46)]. The proteome database of *Pl. halstedii* was searched for protease inhibitors using the known protease inhibitors of *Ph. infestans* as query; representative domains were confirmed using InterProScan [[47](#_ENREF_47)]. Subsequently, it was checked whether there were open reading frames (ORFs) present in the genome with a signature of protease inhibitors but not included in the predicted gene models. For this, a tBlastn search against the masked assembly was done using the *Pl. halstedii* predicted protease inhibitor effectors as query. The tBlastn search revealed the presence of only one ORF present in scaffold 322 positions 1602141 to 1602479 of the assembly that was not included in the gene calls. This ORF was named as Ph_322_1 and putatively encodes for a cystatin-like cysteine protease inhibitor protein that is lacking a start codon due to its presence on a contig break. The predicted protease inhibitors were scanned for the presence of signal peptides (with a HMM score for signal peptide probability of >0.9 and a NN cleavage site within 10-40 amino acids from the starting Methionine) using SignalP, v2 [[48](#_ENREF_48)], and for the absence of transmembrane domains with TMHMM, version 2.0 [[37](#_ENREF_37)], as described earlier [S Raffaele, J Win, LM Cano and S Kamoun [49](#_ENREF_49)]. For those proteins missing signal peptides DNA STRIDER version 1.4f6 [[50](#_ENREF_50)] was used for verification. Amino acid sequences of the regions that corresponded to the Kazal-like or cystatin-like domains were used to build sequence alignments using MUSCLE version 3.6 [[51](#_ENREF_51)] with the option ‘-clw’ to generate outputs in CUSTALW format and ‘-stable’ to restrict the order of the sequences in the output as presented in the input file. To confirm the conservation of the motifs and active residues from both protease inhibitor families predicted in *Pl. halstedii* the sequences of inhibitor effector domains from seven pathogenic oomycetes, *Al. laibachii, Aphanomyces euteiches*, *Hy. arabidopsidis, Ph. infestans*, *Py. ultimum*, and *Sa. parasitica* and were included in the alignments, as well as known inhibitor domains from the non-oomycete species, *Carica papaya*, *Gallus gallus*, *Homo sapiens*, *Mus musculus*, *Pacifastacus leniusculus*, *Sarcophaga peregrine*, and *Toxoplasma gondii*. For visualization of the alignments jalview [[42](#_ENREF_42)] was used, with the colour option based on percentage of identity.

**Crinkler (CRN) protein predictions**

Two approaches were used to identify candidate CRN proteins in the genome of *Pl. halstedii*. In the first approach a regular expression was used by keeping the LFLAK motif conserved and at-most one mismatch was allowed in the recombination motif HVLVVVP. An HMM was trained from this set and whole proteome was searched using HMMER v3 [[52](#_ENREF_52)] with an e-value cut-off of e^-0.05^. In another approach at-most one mismatch was allowed in the conserved LFLAK motif and no mismatch was allowed in the recombination motif HVLVVVP. A HMM was again trained and the whole predicted proteome was scanned. Candidate sets of CRNs generated from these predictions were then merged into a single set.

In the second approach open reading frames in the genome of *Pl. halstedii* were screened for signatures of CRN-like proteins. ORFs were predicted using the EMBOSS package [[53](#_ENREF_53)], ‘getorf’ with a minimum size cut-off of 100 nt and a maximum size cut-off of 6000 nt, additionally translating only the regions between start and stop codons (-find 1). ORFs with similar sequences to known CRNs were identified using BlastP (1e^-4^) against a database of 963 previously reported CRNs from *Ph. infestans* (454), *Ph. ramorum* (64), *Ph. sojae* (207) [[54](#_ENREF_54)] and *Ph. capsici* (237) [[55](#_ENREF_55)]. In order to generate an HMM for recognising candidate CRNs, first the 963 previously reported CRNs [[54](#_ENREF_54), [55](#_ENREF_55)] were scanned for signal peptides using SignalP [[56](#_ENREF_56)]. The sequences with signal peptides were aligned with MUSCLE (v3.8.31) [[51](#_ENREF_51)] and visualised with Seaview [[57](#_ENREF_57)] to confirm the position of the initial methionine and discard poorly aligned sequences. A full length HMM model was then generated from these filtered sequences using the hmmbuild command of HUMMER. Subsequently, hmmsearch (-T 0) was used to identify which of *Pl. halstedii* sequences identified as being similar to CRN sequences by BLAST and also to the full length CRN HMM or the LFLAK HMM from [[54](#_ENREF_54)]. Further filtering was done manually by checking the presence of LFLAK/LYLAK motif in the generated set. Other CRN domains [[54](#_ENREF_54)] were identified with hmmsearch (-T 0). Predicted CRNs were aligned by using Mafft and a phylogenetic tree was constructed using FastTree [[58](#_ENREF_58)]. The sets of CRN like proteins from protein coding genes and ORFs were merged to generate a final non-overlapping set of putative CRN-like proteins.

**RxLR protein predictions**

Candidate secreted proteins with RxLR-dEER-like motifs were extracted by using both regular expressions and HMM. An initial set of putative RxLR-dEER-like proteins was generated using perl regular expressions, as described before [[54](#_ENREF_54)]. This initial set of proteins were then used to build a *Pl. halstedii* sequence specific HMM model and searches in the predicted proteome were done iteratively by using HMMER v3 [[59](#_ENREF_59)] (Supplementary Figure 21).

To complement this approach, all ORFs of *Pl. halstedii* from the unmasked genome were scanned for candidate RxLR-like proteins. These searches were done using the methods previously described [[60](#_ENREF_60)]. First, a heuristic approach was taken to identify sequences predicted to contain a signal peptide cleavage site between 10 and 40 from the initial methionine and an RxLR-dEER motif within in the first 100 residues, a method modified from a previous study [[61](#_ENREF_61)]. A second approach was taken using the cropped HMM constructed by Whisson *et al*. (2007) [[60](#_ENREF_60)] and the HMM constructed by Win *et al*. (2007) [[62](#_ENREF_62)] to identify potential RxLRs candidates using hmmsearch (-T 0, v3.0). Both sets of RxLR-like proteins generated from protein sequences and translated ORFs were combined and a final non-overlapping set was generated. Candidate RxLR effectors were classified according to the presence of RxLR-dEER motifs: (AAA) At least two effectors with at-most one mismatch in the RxLR motif and no mismatch in the dEER motif. (AA) No mismatch in the RxLR motif and at-most one mismatch in the dEER motif, and (A) At-most one mismatch in the RxLR motif and no mismatch in the dEER motif.

The proteome of *Pl. halstedii* was searched with HMMER (v 2.3.2) [[52](#_ENREF_52)] using the WY-fold HMM as reported previously [[63](#_ENREF_63)]. All proteins with HMM score > 0.0 were considered to contain this motif.

**Expression profiling**

Samples corresponding to newly formed spores (Spores), early stages of infection (Infection) and the fully established infection (Sporulation) were aligned with the predicted genes of *Pl. halstedii* using SAMtools (http://samtools.sourceforge.net/) and the Burrows-Wheeler Aligner (BWA) (http://bio-bwa.sourceforge.net/). Quantitation was performed using SeqMonk (http://www.bioinformatics.babraham.ac.uk/projects/seqmonk/). Effector candidates were then clustered based on a minimal log fold change of 2 between experimental conditions.

**References**

1. McKinney EC, Ali N, Traut A, Feldmann KA, Belostotsky DA, McDowell JM, Meagher RB: **Sequence-based identification of T-DNA insertion mutations in Arabidopsis: actin mutants act2-1 and act4-1**. *The Plant journal : for cell and molecular biology* 1995, **8**(4):613-622.

2. Altschul SF, Gish W, Miller W, Myers EW, Lipman DJ: **Basic local alignment search tool**. *Journal of molecular biology* 1990, **215**(3):403-410.

3. Langmead B, Salzberg SL: **Fast gapped-read alignment with Bowtie 2**. *Nature methods* 2012, **9**(4):357-359.

4. Bao Z, Eddy SR: **Automated de novo identification of repeat sequence families in sequenced genomes**. *Genome research* 2002, **12**(8):1269-1276.

5. Price AL, Jones NC, Pevzner PA: **De novo identification of repeat families in large genomes**. *Bioinformatics* 2005, **21 Suppl 1**:i351-358.

6. Jurka J, Kapitonov VV, Pavlicek A, Klonowski P, Kohany O, Walichiewicz J: **Repbase Update, a database of eukaryotic repetitive elements**. *Cytogenetic and genome research* 2005, **110**(1-4):462-467.

7. Benson G: **Tandem repeats finder: a program to analyze DNA sequences**. *Nucleic acids research* 1999, **27**(2):573-580.

8. Kim D, Pertea G, Trapnell C, Pimentel H, Kelley R, Salzberg SL: **TopHat2: accurate alignment of transcriptomes in the presence of insertions, deletions and gene fusions**. *Genome biology* 2013, **14**(4):R36.

9. Trapnell C, Williams BA, Pertea G, Mortazavi A, Kwan G, van Baren MJ, Salzberg SL, Wold BJ, Pachter L: **Transcript assembly and quantification by RNA-Seq reveals unannotated transcripts and isoform switching during cell differentiation**. *Nature biotechnology* 2010, **28**(5):511-515.

10. Borodovsky M, Lomsadze A: **Eukaryotic gene prediction using GeneMark.hmm-E and GeneMark-ES**. *Current protocols in bioinformatics / editoral board, Andreas D Baxevanis [et al]* 2011, **Chapter 4**:Unit 4 6 1-10.

11. Stanke M, Schoffmann O, Morgenstern B, Waack S: **Gene prediction in eukaryotes with a generalized hidden Markov model that uses hints from external sources**. *BMC bioinformatics* 2006, **7**:62.

12. Haas BJ, Delcher AL, Mount SM, Wortman JR, Smith RK, Jr., Hannick LI, Maiti R, Ronning CM, Rusch DB, Town CD *et al*: **Improving the Arabidopsis genome annotation using maximal transcript alignment assemblies**. *Nucleic acids research* 2003, **31**(19):5654-5666.

13. Wu TD, Watanabe CK: **GMAP: a genomic mapping and alignment program for mRNA and EST sequences**. *Bioinformatics* 2005, **21**(9):1859-1875.

14. Haas BJ, Salzberg SL, Zhu W, Pertea M, Allen JE, Orvis J, White O, Buell CR, Wortman JR: **Automated eukaryotic gene structure annotation using EVidenceModeler and the Program to Assemble Spliced Alignments**. *Genome biology* 2008, **9**(1):R7.

15. Conesa A, Gotz S, Garcia-Gomez JM, Terol J, Talon M, Robles M: **Blast2GO: a universal tool for annotation, visualization and analysis in functional genomics research**. *Bioinformatics* 2005, **21**(18):3674-3676.

16. Tatusov RL, Fedorova ND, Jackson JD, Jacobs AR, Kiryutin B, Koonin EV, Krylov DM, Mazumder R, Mekhedov SL, Nikolskaya AN *et al*: **The COG database: an updated version includes eukaryotes**. *BMC bioinformatics* 2003, **4**:41.

17. Ashburner M, Ball CA, Blake JA, Botstein D, Butler H, Cherry JM, Davis AP, Dolinski K, Dwight SS, Eppig JT *et al*: **Gene ontology: tool for the unification of biology. The Gene Ontology Consortium**. *Nature genetics* 2000, **25**(1):25-29.

18. Hunter S, Apweiler R, Attwood TK, Bairoch A, Bateman A, Binns D, Bork P, Das U, Daugherty L, Duquenne L *et al*: **InterPro: the integrative protein signature database**. *Nucleic acids research* 2009, **37**(Database issue):D211-215.

19. Finn RD, Tate J, Mistry J, Coggill PC, Sammut SJ, Hotz HR, Ceric G, Forslund K, Eddy SR, Sonnhammer EL *et al*: **The Pfam protein families database**. *Nucleic acids research* 2008, **36**(Database issue):D281-288.

20. Nepusz T, Sasidharan R, Paccanaro A: **SCPS: a fast implementation of a spectral method for detecting protein families on a genome-wide scale**. *BMC bioinformatics* 2010, **11**:120.

21. Enright AJ, Van Dongen S, Ouzounis CA: **An efficient algorithm for large-scale detection of protein families**. *Nucleic acids research* 2002, **30**(7):1575-1584.

22. Kanehisa M, Goto S: **KEGG: kyoto encyclopedia of genes and genomes**. *Nucleic acids research* 2000, **28**(1):27-30.

23. Moriya Y, Itoh M, Okuda S, Yoshizawa AC, Kanehisa M: **KAAS: an automatic genome annotation and pathway reconstruction server**. *Nucleic acids research* 2007, **35**(Web Server issue):W182-185.

24. Mi H, Lazareva-Ulitsky B, Loo R, Kejariwal A, Vandergriff J, Rabkin S, Guo N, Muruganujan A, Doremieux O, Campbell MJ *et al*: **The PANTHER database of protein families, subfamilies, functions and pathways**. *Nucleic acids research* 2005, **33**(Database issue):D284-288.

25. Li H, Durbin R: **Fast and accurate short read alignment with Burrows-Wheeler transform**. *Bioinformatics* 2009, **25**(14):1754-1760.

26. Li H: **Aligning sequence reads, clone sequences and assembly contigs with BWA-MEM**. *Genomics* 2013:1-3.

27. Li H, Handsaker B, Wysoker A, Fennell T, Ruan J, Homer N, Marth G, Abecasis G, Durbin R, Genome Project Data Processing S: **The Sequence Alignment/Map format and SAMtools**. *Bioinformatics* 2009, **25**(16):2078-2079.

28. R Development Core Team R: **R: A Language and Environment for Statistical Computing.** *R Foundation for Statistical Computing* 2013.

29. Faircloth BC: **msatcommander: detection of microsatellite repeat arrays and automated, locus-specific primer design**. *Molecular ecology resources* 2008, **8**(1):92-94.

30. Rozen S, Skaletsky H: **Primer3 on the WWW for general users and for biologist programmers**. *Methods in molecular biology* 2000, **132**:365-386.

31. Wang X, Lu P, Luo Z: **GMATo: A novel tool for the identification and analysis of microsatellites in large genomes**. *Bioinformation* 2013, **9**(10):541-544.

32. Untergasser A, Cutcutache I, Koressaar T, Ye J, Faircloth BC, Remm M, Rozen SG: **Primer3--new capabilities and interfaces**. *Nucleic acids research* 2012, **40**(15):e115.

33. Koressaar T, Remm M: **Enhancements and modifications of primer design program Primer3**. *Bioinformatics* 2007, **23**(10):1289-1291.

34. Tamura K, Stecher G, Peterson D, Filipski A, Kumar S: **MEGA6: Molecular Evolutionary Genetics Analysis version 6.0**. *Molecular biology and evolution* 2013, **30**(12):2725-2729.

35. Nielsen H, Engelbrecht J, Brunak S, von Heijne G: **Identification of prokaryotic and eukaryotic signal peptides and prediction of their cleavage sites**. *Protein engineering* 1997, **10**(1):1-6.

36. Emanuelsson O, Nielsen H, Brunak S, von Heijne G: **Predicting subcellular localization of proteins based on their N-terminal amino acid sequence**. *Journal of molecular biology* 2000, **300**(4):1005-1016.

37. Krogh A, Larsson B, von Heijne G, Sonnhammer EL: **Predicting transmembrane protein topology with a hidden Markov model: application to complete genomes**. *Journal of molecular biology* 2001, **305**(3):567-580.

38. Blin K, Medema MH, Kazempour D, Fischbach MA, Breitling R, Takano E, Weber T: **antiSMASH 2.0--a versatile platform for genome mining of secondary metabolite producers**. *Nucleic acids research* 2013, **41**(Web Server issue):W204-212.

39. Medema MH, Blin K, Cimermancic P, de Jager V, Zakrzewski P, Fischbach MA, Weber T, Takano E, Breitling R: **antiSMASH: rapid identification, annotation and analysis of secondary metabolite biosynthesis gene clusters in bacterial and fungal genome sequences**. *Nucleic acids research* 2011, **39**(Web Server issue):W339-346.

40. Katoh K, Standley DM: **MAFFT multiple sequence alignment software version 7: improvements in performance and usability**. *Molecular biology and evolution* 2013, **30**(4):772-780.

41. Stamatakis A: **RAxML-VI-HPC: maximum likelihood-based phylogenetic analyses with thousands of taxa and mixed models**. *Bioinformatics* 2006, **22**(21):2688-2690.

42. Waterhouse AM, Procter JB, Martin DM, Clamp M, Barton GJ: **Jalview Version 2--a multiple sequence alignment editor and analysis workbench**. *Bioinformatics* 2009, **25**(9):1189-1191.

43. Tamura K, Peterson D, Peterson N, Stecher G, Nei M, Kumar S: **MEGA5: molecular evolutionary genetics analysis using maximum likelihood, evolutionary distance, and maximum parsimony methods**. *Molecular biology and evolution* 2011, **28**(10):2731-2739.

44. Oome S, Van den Ackerveken G: **Comparative and functional analysis of the widely occurring family of Nep1-like proteins**. *Molecular plant-microbe interactions : MPMI* 2014.

45. Okonechnikov K, Golosova O, Fursov M, team U: **Unipro UGENE: a unified bioinformatics toolkit**. *Bioinformatics* 2012, **28**(8):1166-1167.

46. Camacho C, Coulouris G, Avagyan V, Ma N, Papadopoulos J, Bealer K, Madden TL: **BLAST+: architecture and applications**. *BMC bioinformatics* 2009, **10**:421.

47. Goujon M, McWilliam H, Li W, Valentin F, Squizzato S, Paern J, Lopez R: **A new bioinformatics analysis tools framework at EMBL-EBI**. *Nucleic acids research* 2010, **38**(Web Server issue):W695-699.

48. Nielsen H, Krogh A: **Prediction of signal peptides and signal anchors by a hidden Markov model**. *Proceedings / International Conference on Intelligent Systems for Molecular Biology ; ISMB International Conference on Intelligent Systems for Molecular Biology* 1998, **6**:122-130.

49. Raffaele S, Win J, Cano LM, Kamoun S: **Analyses of genome architecture and gene expression reveal novel candidate virulence factors in the secretome of Phytophthora infestans**. *BMC genomics* 2010, **11**:637.

50. Douglas SE: **DNA Strider. An inexpensive sequence analysis package for the Macintosh**. *Molecular biotechnology* 1995, **3**(1):37-45.

51. Edgar RC: **MUSCLE: multiple sequence alignment with high accuracy and high throughput**. *Nucleic acids research* 2004, **32**(5):1792-1797.

52. Eddy SR: **A new generation of homology search tools based on probabilistic inference**. *Genome informatics International Conference on Genome Informatics* 2009, **23**(1):205-211.

53. Rice P, Longden I, Bleasby A: **EMBOSS: the European Molecular Biology Open Software Suite**. *Trends in genetics : TIG* 2000, **16**(6):276-277.

54. Haas BJ, Kamoun S, Zody MC, Jiang RH, Handsaker RE, Cano LM, Grabherr M, Kodira CD, Raffaele S, Torto-Alalibo T *et al*: **Genome sequence and analysis of the Irish potato famine pathogen Phytophthora infestans**. *Nature* 2009, **461**(7262):393-398.

55. Stam R, Jupe J, Howden AJ, Morris JA, Boevink PC, Hedley PE, Huitema E: **Identification and Characterisation CRN Effectors in Phytophthora capsici Shows Modularity and Functional Diversity**. *PLoS ONE* 2013, **8**(3):e59517.

56. Petersen TN, Brunak S, von Heijne G, Nielsen H: **SignalP 4.0: discriminating signal peptides from transmembrane regions**. *Nature methods* 2011, **8**(10):785-786.

57. Gouy M, Guindon S, Gascuel O: **SeaView version 4: A multiplatform graphical user interface for sequence alignment and phylogenetic tree building**. *Molecular biology and evolution* 2010, **27**(2):221-224.

58. Price MN, Dehal PS, Arkin AP: **FastTree 2--approximately maximum-likelihood trees for large alignments**. *PLoS ONE* 2010, **5**(3):e9490.

59. Durbin R. ES, Krogh A. and Mitchison G.: **Biological sequence analysis: probabilistic models of proteins and nucleic acids**: Cambridge University Press.; 1998.

60. Whisson SC, Boevink PC, Moleleki L, Avrova AO, Morales JG, Gilroy EM, Armstrong MR, Grouffaud S, van West P, Chapman S *et al*: **A translocation signal for delivery of oomycete effector proteins into host plant cells**. *Nature* 2007, **450**(7166):115-118.

61. Bhattacharjee S, Hiller NL, Liolios K, Win J, Kanneganti TD, Young C, Kamoun S, Haldar K: **The malarial host-targeting signal is conserved in the Irish potato famine pathogen**. *PLoS pathogens* 2006, **2**(5):e50.

62. Win J, Morgan W, Bos J, Krasileva KV, Cano LM, Chaparro-Garcia A, Ammar R, Staskawicz BJ, Kamoun S: **Adaptive evolution has targeted the C-terminal domain of the RXLR effectors of plant pathogenic oomycetes**. *The Plant cell* 2007, **19**(8):2349-2369.

63. Boutemy LS, King SR, Win J, Hughes RK, Clarke TA, Blumenschein TM, Kamoun S, Banfield MJ: **Structures of Phytophthora RXLR effector proteins: a conserved but adaptable fold underpins functional diversity**. *The Journal of biological chemistry* 2011, **286**(41):35834-35842.
