# Supplementary material for: Genome analyses of the sunflower pathogen Plasmopara halstedii provide insights into effector evolution in downy mildews and Phytophthora
Source: BMC Genomics. 2015 Oct 5;16:741. doi: 10.1186/s12864-015-1904-7 (PMC4594904; doi:10.1186/s12864-015-1904-7)
Supplement: Additional file 2: — Supplementary figures. (PDF 34119 kb) [file 12864_2015_1904_MOESM2_ESM.pdf]

# Supplementary Figure 1

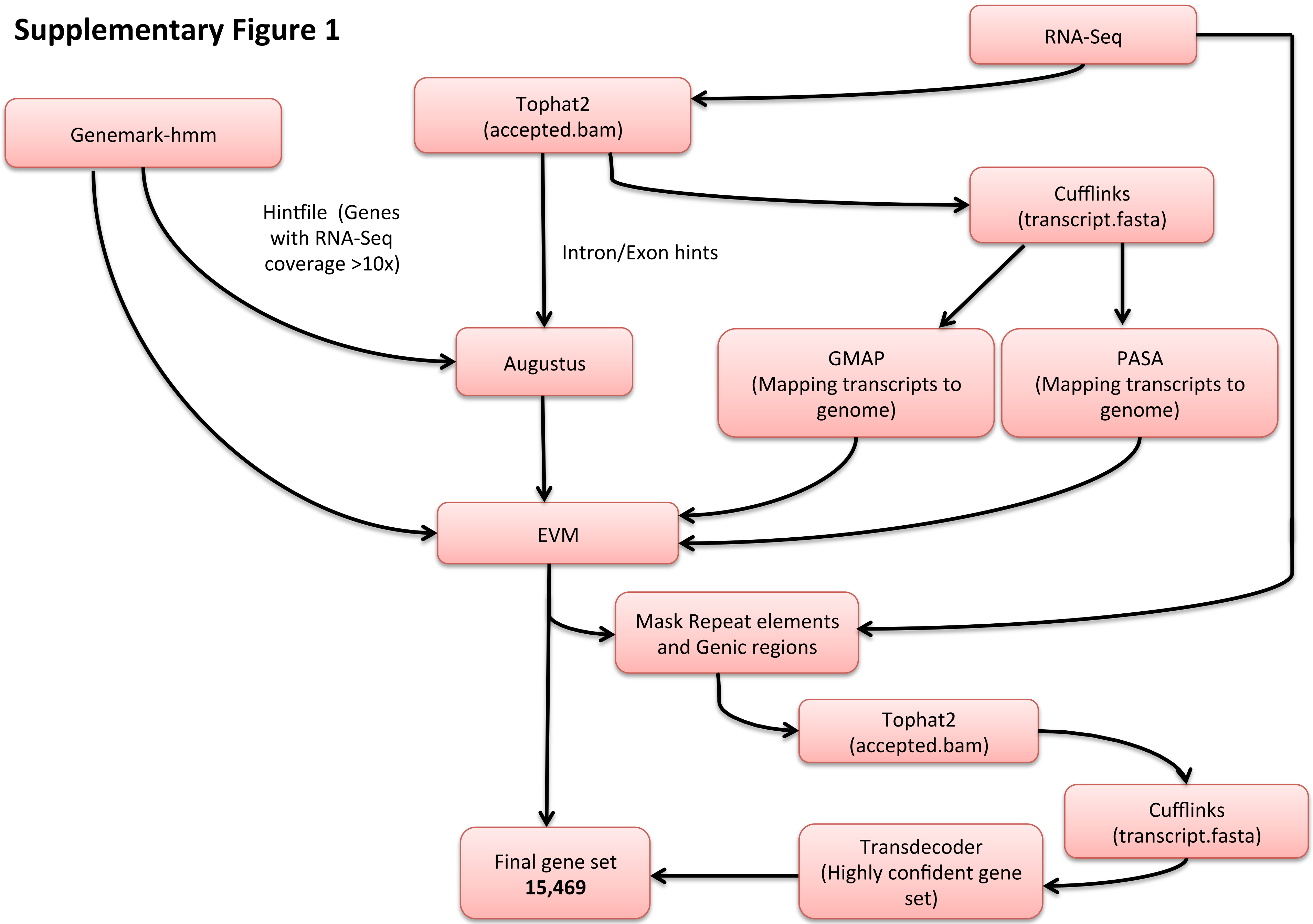


**Supplementary Figure 1. Gene prediction pipeline (adapted from *sharma et. al.* 2015):** Gene prediction pipeline used to generate highly confident gene models using both *ab-initio* based and transcript mapping based approaches.

## Supplementary Figure 2

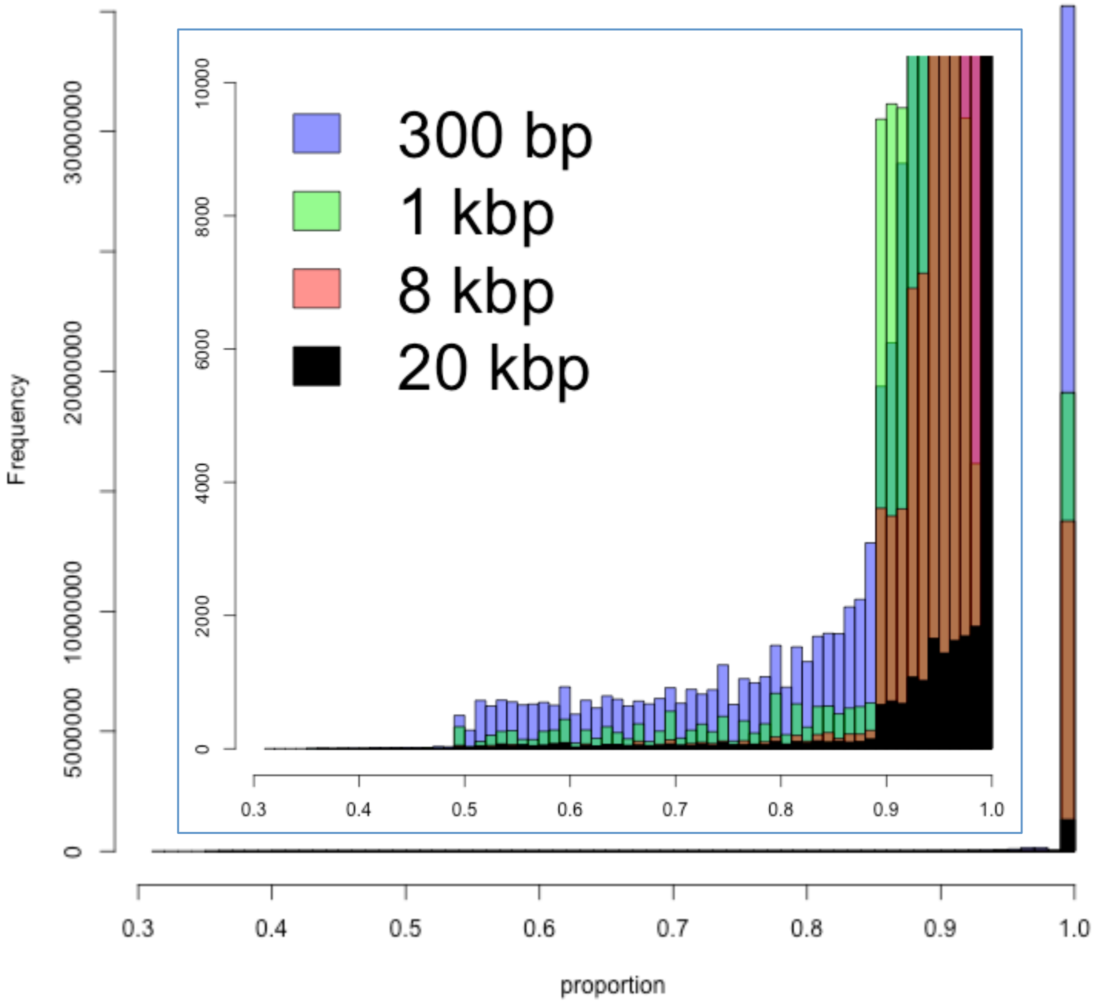


**Supplementary Figure 2. Low levels of heterozygosity in *Pl. halstedii***. The histogram shows the frequency distribution for proportions of aligned reads matching the major allele. Proportions are based alignment of sequence reads against the *P. halstedii* reference assembly. Data are color-coded according to which of the four genomic libraries was used to generate the alignments. The inset shows the same data as the main figure but with the vertical axis truncated at 10,000. Heterozygous sites would be expected to generate a major-allele-frequency proportion close to 0.5 whilst homozygous sites would fall close to 1; therefore, in a diploid genome with significant levels of heterozygosity, we would expect a bimodal frequency distribution with peaks close to 0.5 and close to 1. The alignment of sequence reads against the reference genome was performed with BWA (Li & Durbin, 2009) as described in the Methods section.

# Supplementary Figure 3

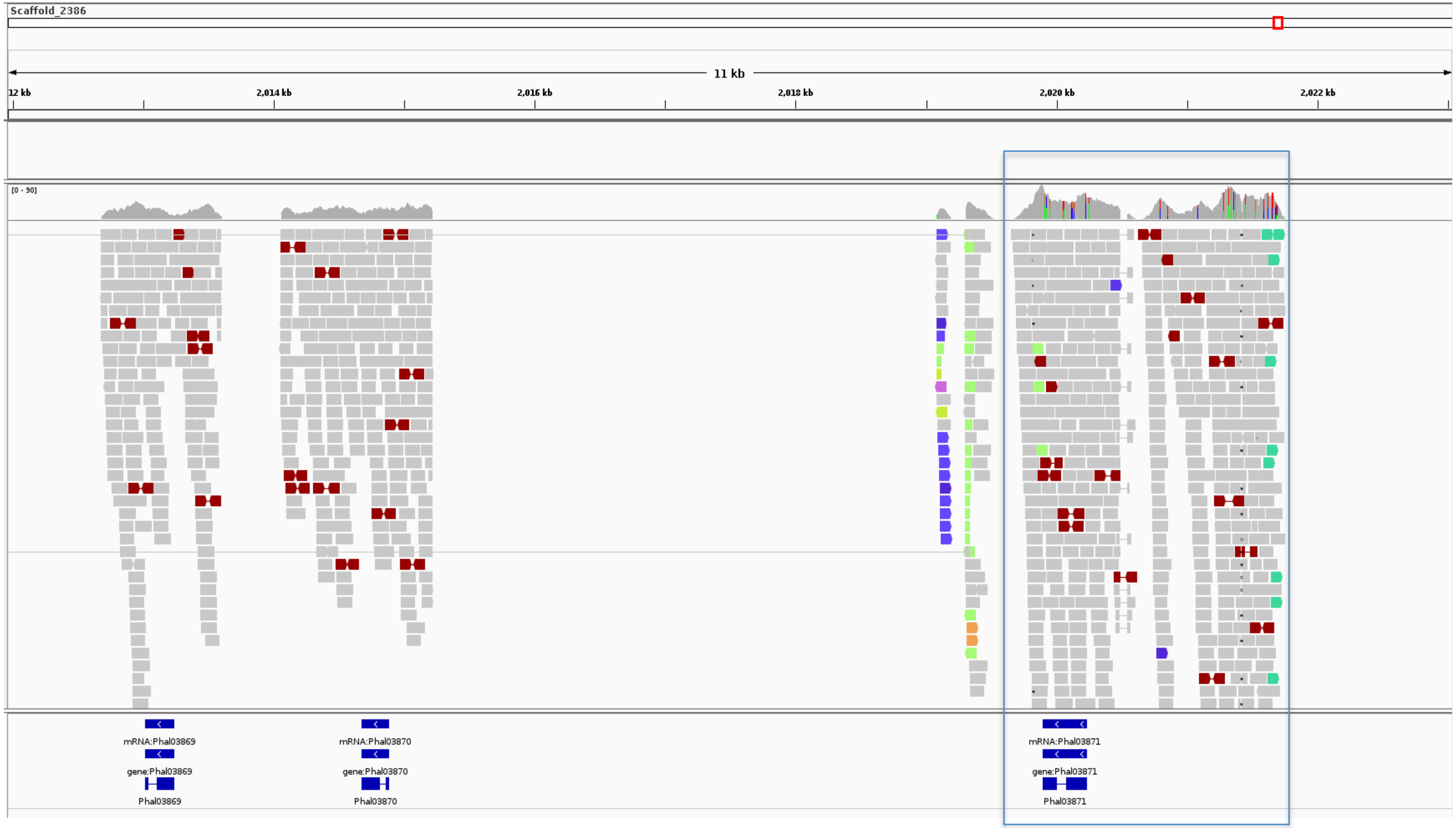


**Supplementary Figure 3:** A region of apparent heterozygosity on scaffold 2386. We aligned sequence reads from the 300-bp genomic library against the the *P. halstedii* genome assembly using BWA-mem and visualized this 11-kb region of the alignment using IGV. Sequence reads that match multiple sites on the genome are excluded. The region of apparent heterozygosity is indicated in the blue rectangle.

# Supplementary Figure 4

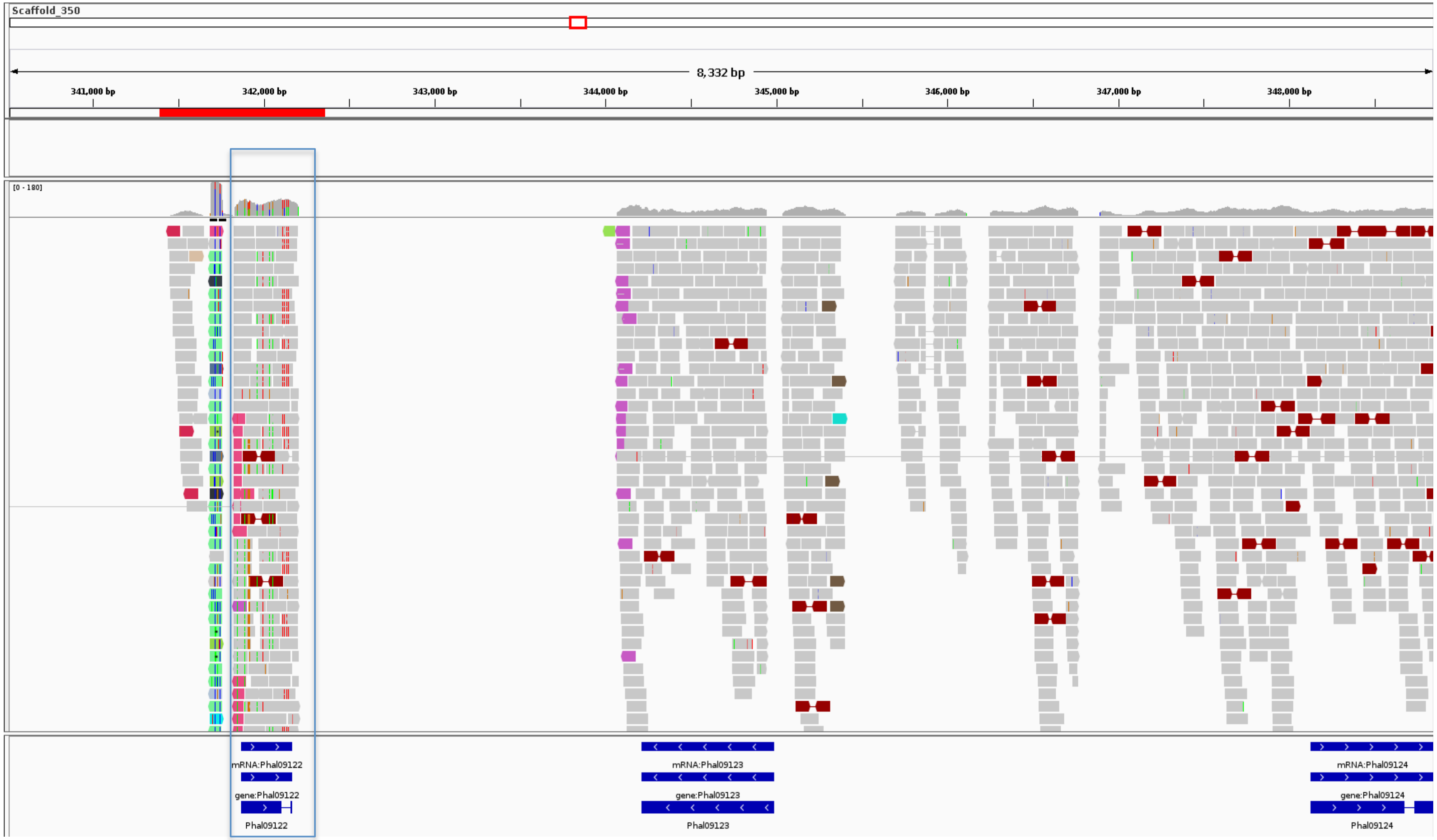


**Supplementary Figure 4:** A region of apparent heterozygosity on scaffold 350. We aligned sequence reads from the 300-bp genomic library against the the *P. halstedii* genome assembly using BWA-mem and visualized this 8.3-kb region of the alignment using IGV. Sequence reads that match multiple sites on the genome are excluded.  The region of apparent heterozygosity is indicated in the blue rectangle.

# Supplementary Figure 5

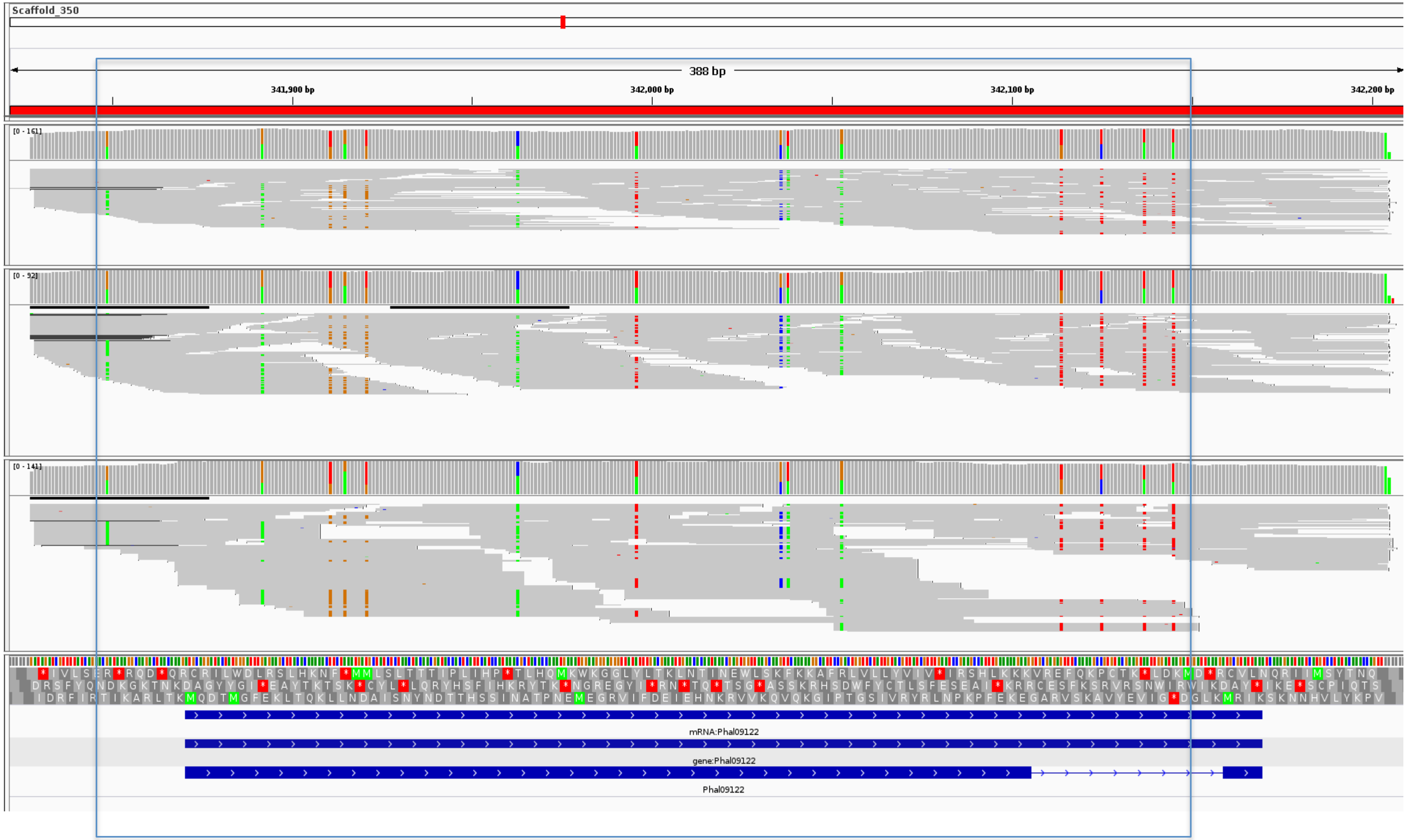


**Supplementary Figure 5:** A region of apparent heterozygosity on scaffold 350. We aligned sequence reads from the 300-bp, 1-kb and 8-kb genomic libraries against the the *P. halstedii* genome assembly using BWA-mem and visualized this 300-bp region of the alignment using IGV. Sequence reads that match multiple sites on the genome are excluded.  The region of apparent heterozygosity is indicated in the blue rectangle.

# Supplementary Figure 6

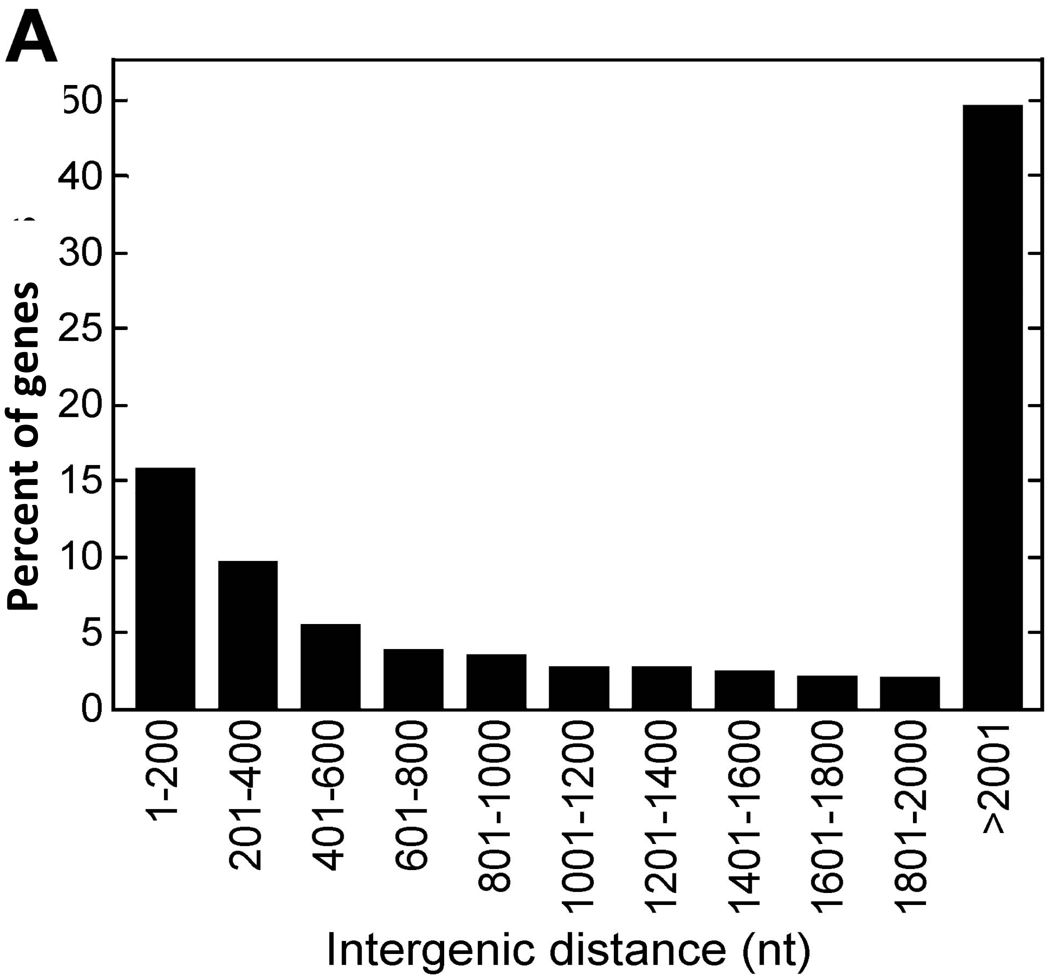


**Supplementary Figure 6:** Representation of the genome architecture of *Pl. halstedii*.

# Supplementary Figure 7

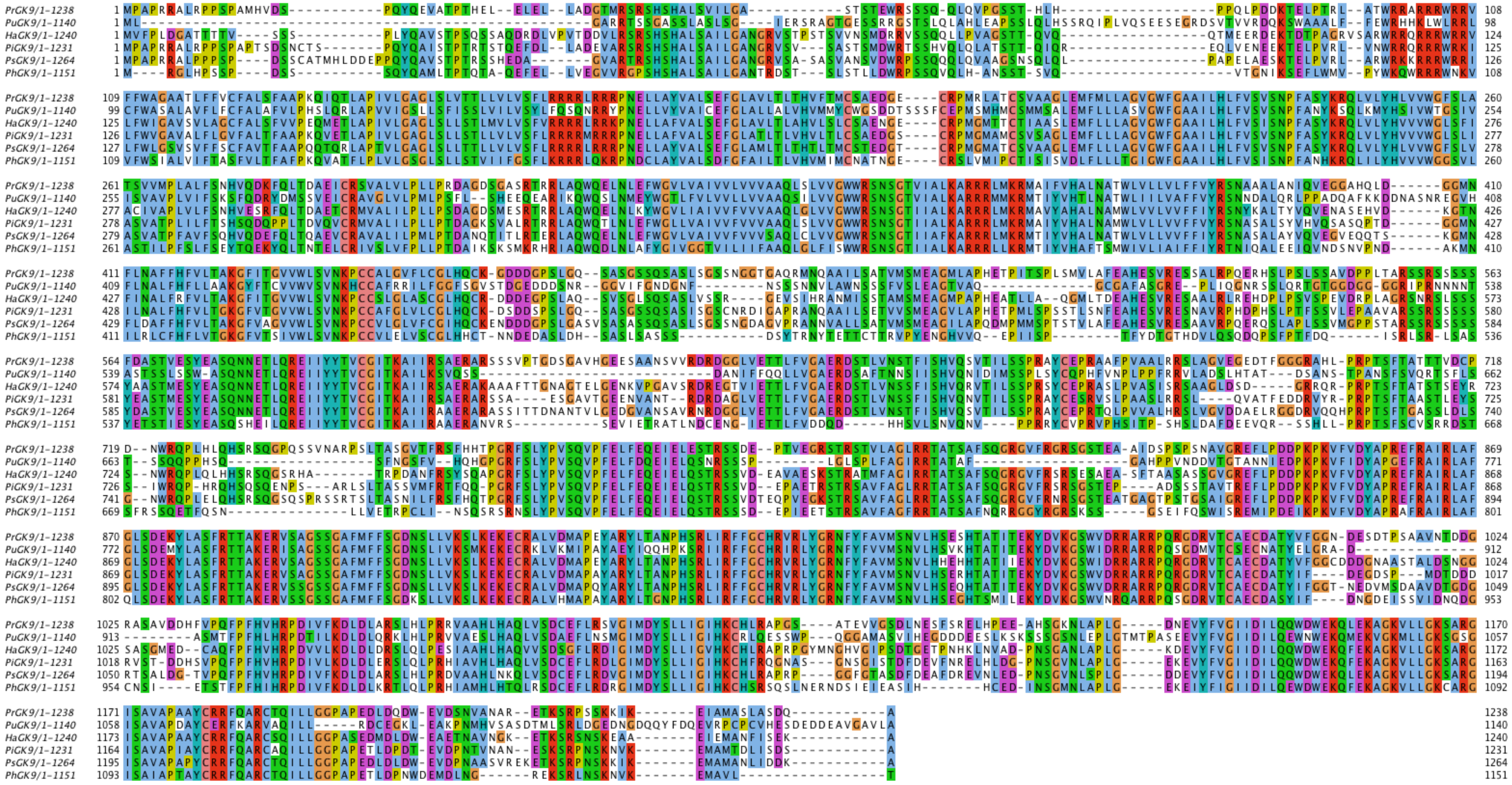

**Supplementary Figure 7**: Conservation of GK9 in the oomycete genomes.

## Supplementary Figure 8

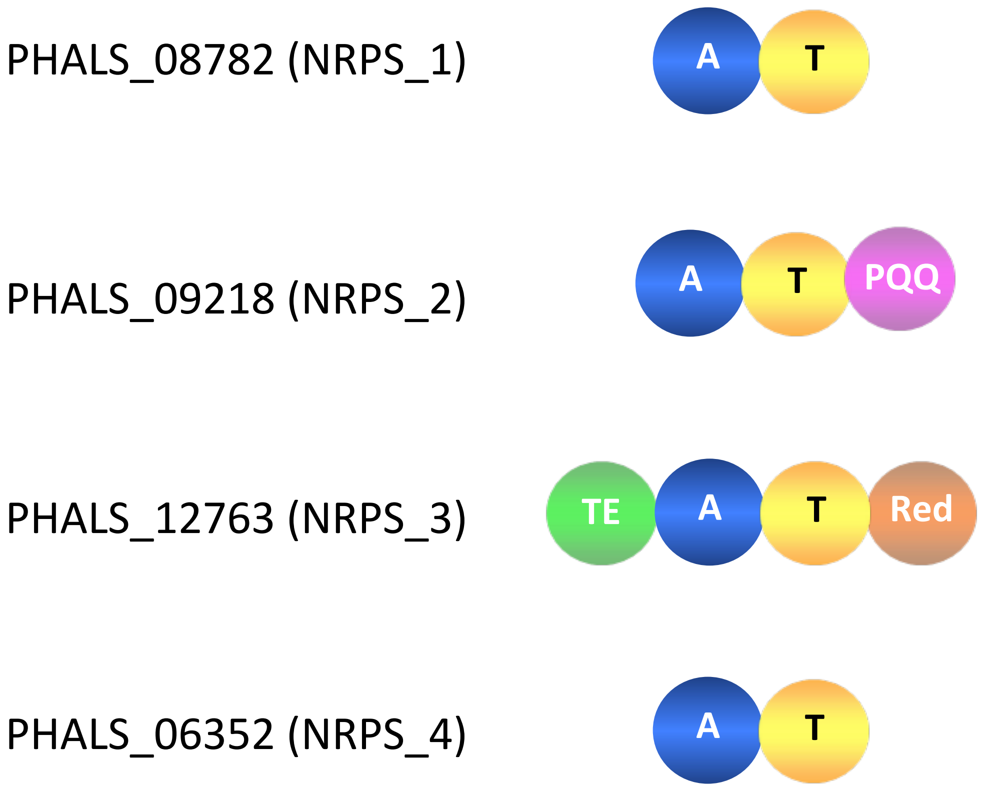


**Supplementary Figure 8**: Domain organisation of the NRPS encoded in the *P. halstedii* genome. Adelylation (A), thiolation (T), PQQ binding (PQQ), thioesterase (TE), and reductase (Red) domains are shown, which were identified by BLAST-P searches.

# Supplementary Figure 9

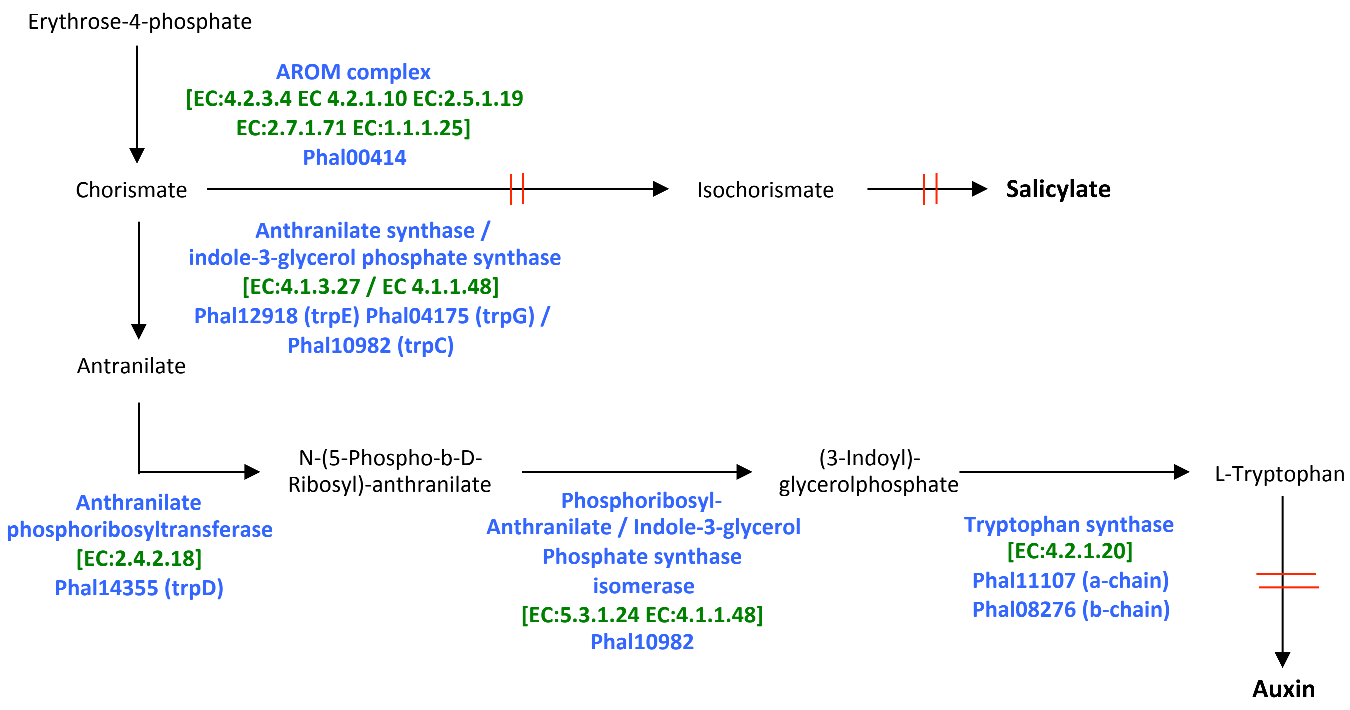


**Supplementary Figure 9**: Genes involved in the auxin production .

## Supplementary Figure 10

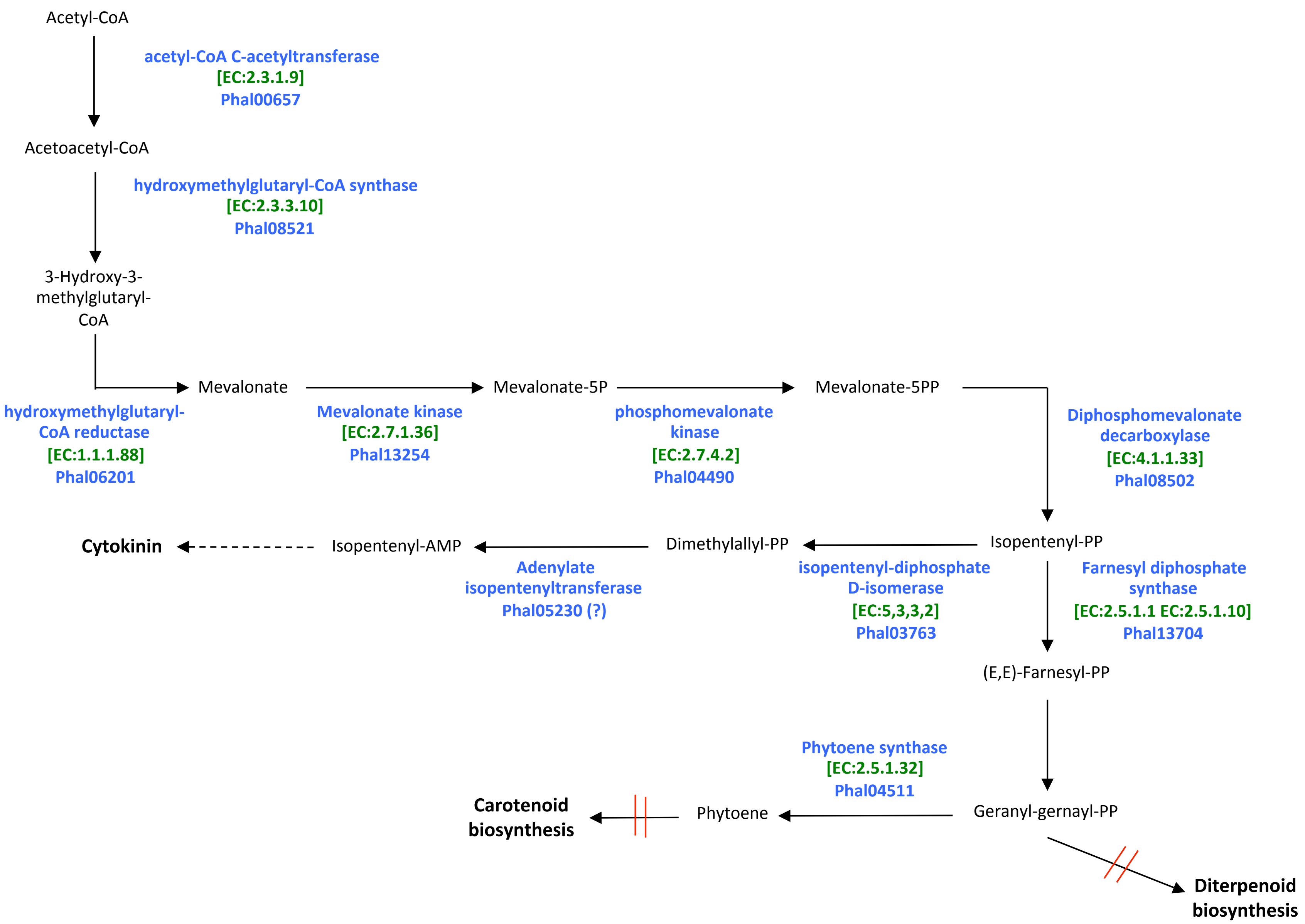


**Supplementary Figure 10**: Non-functional diterpenoid biosynthesis pathway.

# Supplementary Figure 11

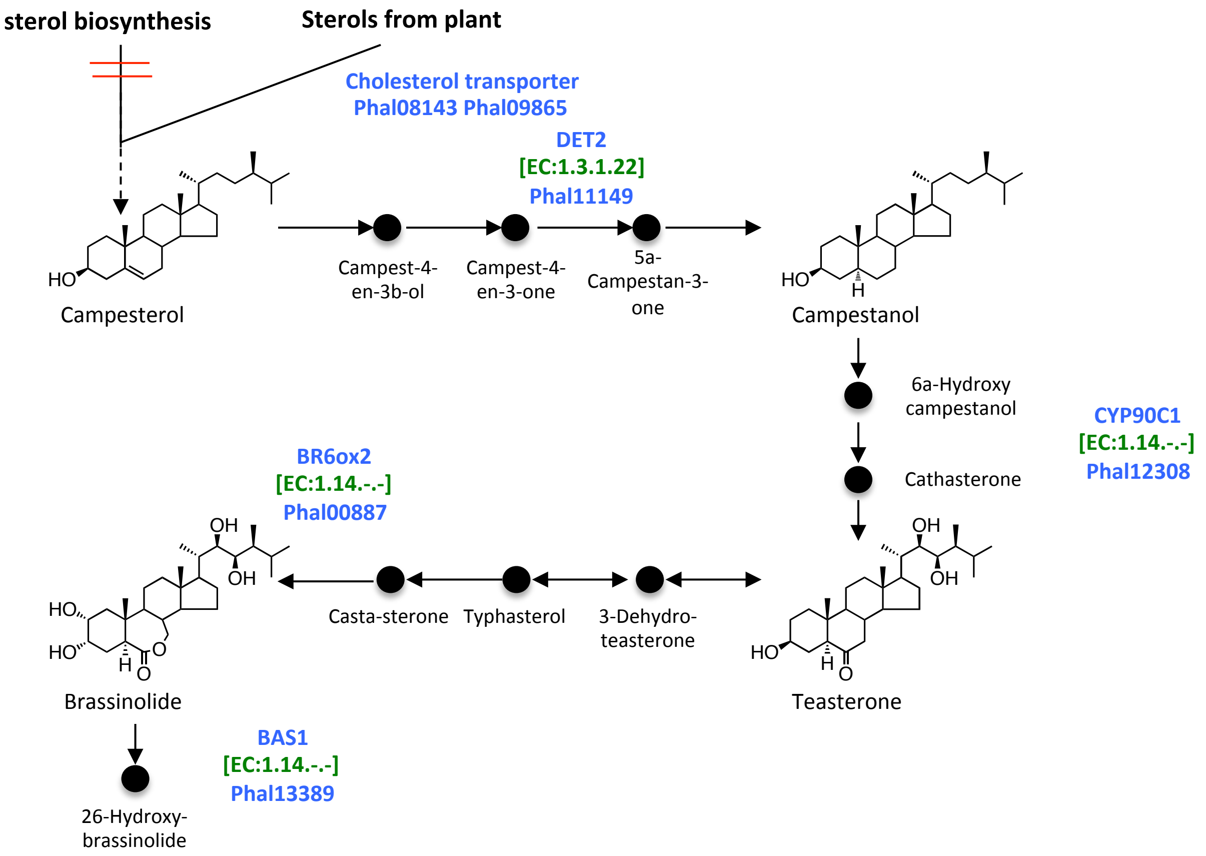


**Supplementary Figure 11**: Genes involved in the brassinolide biosynthesis pathway.

# Supplementary Figure 12

***Phal06084 aligned with HaNLP3 and NLPPya.***

```
Signal peptide
Glutamines in Q-rich region
Conserved cysteines
Heptapeptide motif
Residues making up a cation binding pocket
Residues next to cation binding pocket

NLPPya          --MVRFVSALLLAAAGVLASTN-------------------AAVINHDAVPVWPQPEPAD 39
HaNLP3          MKLDGFITTAILAHIPVYARNDYVQEEKQQQLQEPLDGQWKPTTTGHDAIVPFSEPKPVT 60
Phal06084       MSIVSFFSFAVLMVSMIQAQTQNQENLKVTDQQEQLTADWQPKTIPYDQVRGFRQPKPTT 60
                  :  *.:  :*    : * .:                   . .  :* :  : :*:*.

NLPPya          ATQALAVRFKPQLDVVNGCQPYPAVDPQGNTSGGLKPSGSQAAACR-DMSKAQVYSRSGT 98
HaNLP3          ISEKAGVKFKPLLDVNTGCAPYAAVNAEGETSGGLQTSGDPESGCRGSKYGSQVYGRSTW 120
Phal06084       ISQQVALTFKPQLDIVTGCLPYPAVNRLGETSGGLPKDADTETKCHGSQFNSQVYGRSTW 120
                 ::  .: *** **: .** **.**:  *:*****  ...  : *: .   :***.**

NLPPya          YNGYYAIMYSWYMPKDSPSTGIGHRHDWENVVVWLDN--AASANIVALSASAHSGYKKSF 156
HaNLP3          YNDVWAIMYAWYFPKDSPMLLMGHRHDWENVVVFINDPDEVEPTILGCSTSWHSGYIKYA 180
Phal06084       FNDVWVMMFSWYFPKDSPFPLLGHRHDWENVVIFIDSPKVPLPKILGCSMSYHSEYIKFA 180
                :*. :.:*::**:*****   :**********::::.     ..*:. * * ** * *

NLPPya          PADKSYLDGITAKISYKSTWPLDHELGFTTSAGKQQPLIQWEQMTQAARDALESTDFGNA 216
HaNLP3          PCPTDSINGSSVMIKYEHSFPLNHALNITKDAGAYQDLIMWHQMPDLARRALNDTDFGKA 240
Phal06084       PCPTFATNGTSVKVKYEDSFPLNHALNVTEKEGTFQDLIMWNQLPPIVRKSLNDTDFGSA 240
                *. .   :* :. :.*: ::**:* *..* . *  * ** *.*:.  .* :*:.****.*

NLPPya          NVPFKS-NFQDKLVKAF-FQ------ 234
HaNLP3          ITPMNDLNFMEKIEAAWPFKTKKDGA 266
Phal06084       NVPMNDWNFLKKVEEAWPFT------ 260
                 .*::. ** .*:  *: *
```

**Supplementary Figure 12**: Multiple alignment of the *Pythium aphanidermatum* NLPPya, the *Hyaloperonospora arabidopsidis* HaNLP3, and the *Plasmopara halstedii* Phal06084. Phal06084 and HaNLP3 both have a Q-rich region and all residues making up the cation-binding pocket. Phal06084 and HaNLP3 also both have an additional disulfide bridge, only found in one branch of a phylogenetic tree of oomycete NLPs.

# Supplementary Figure 13

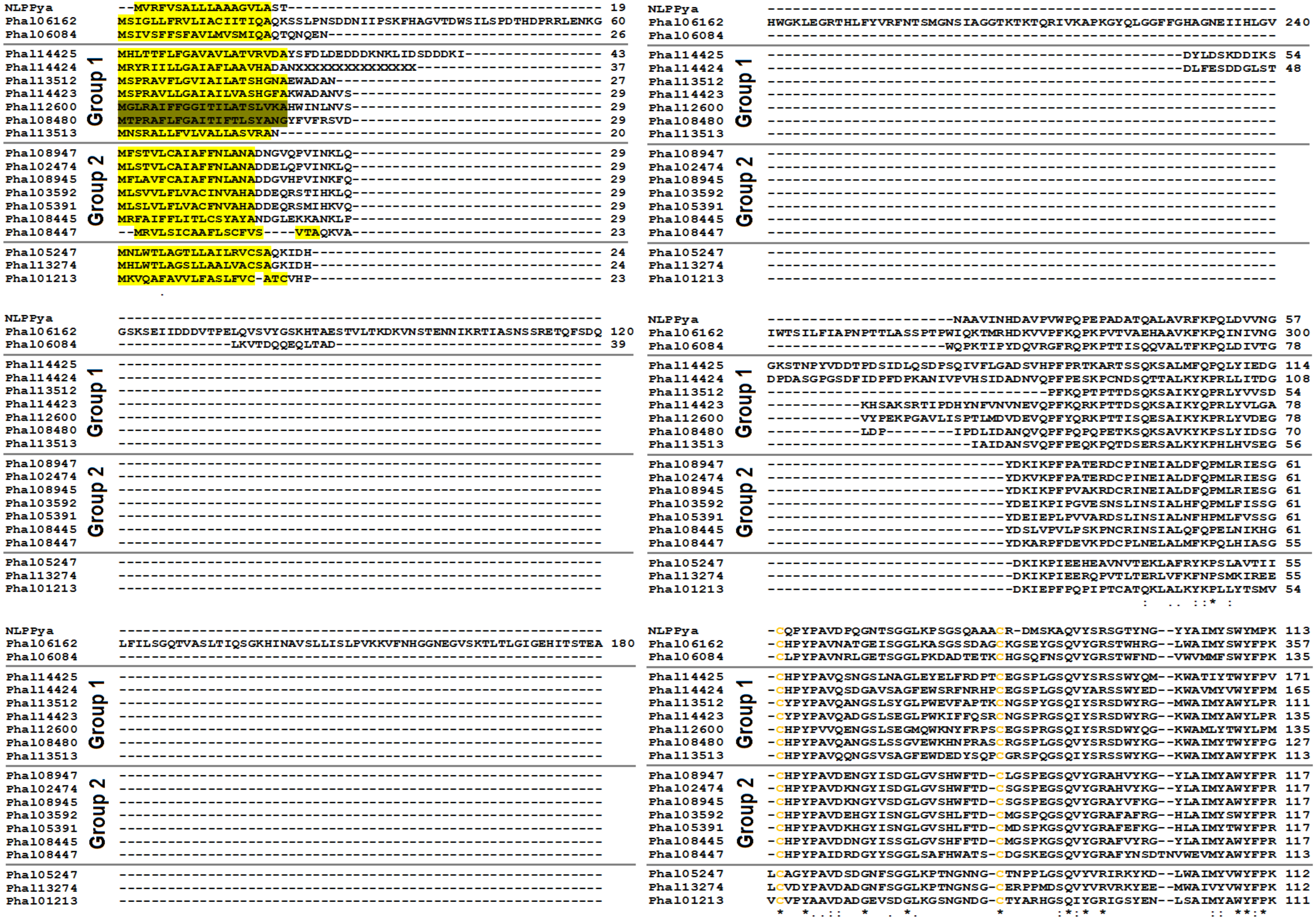

# Supplementary Figure 13

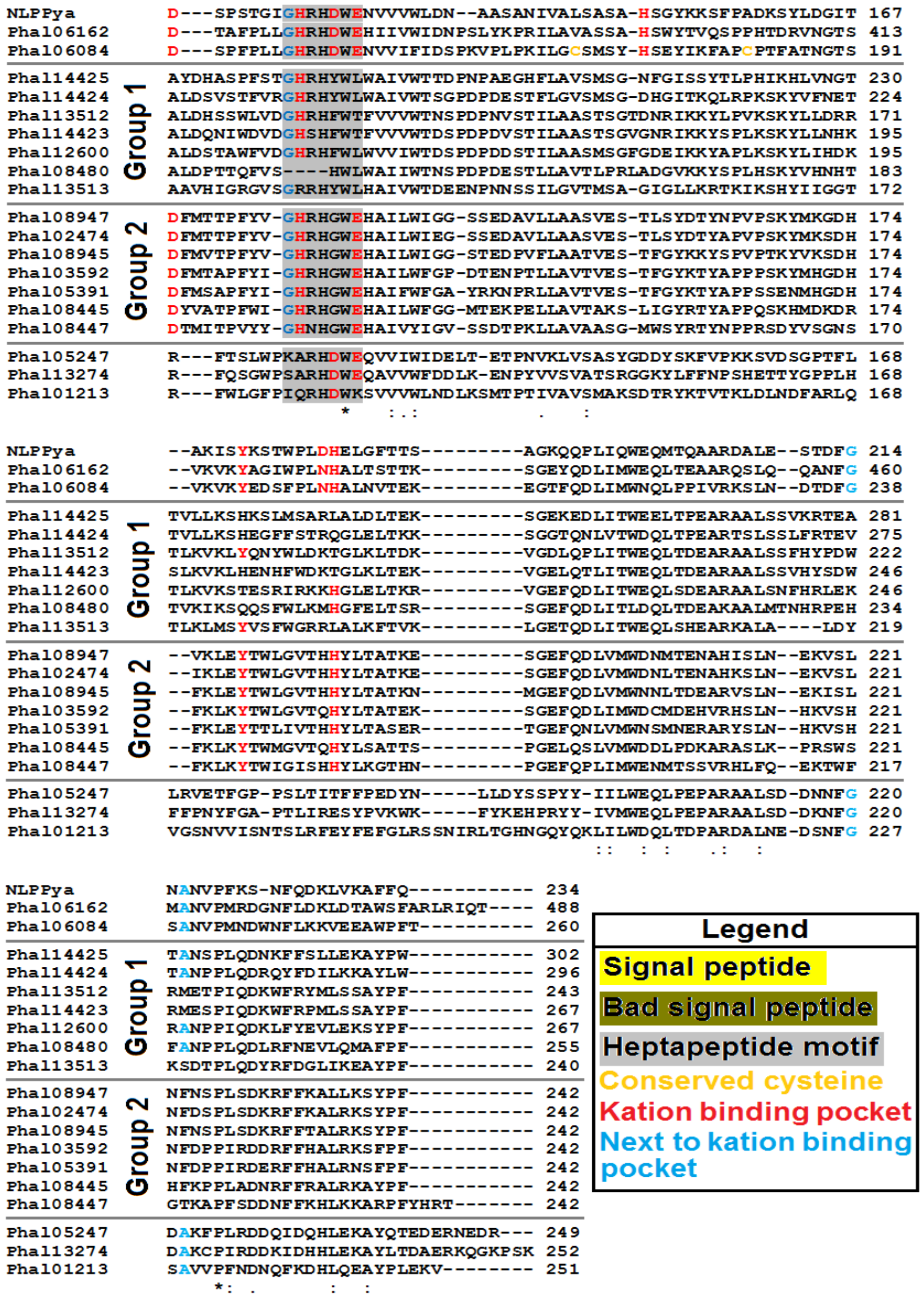


**Supplementary Figure 13**: Multiple alignment of the *Pythium aphanidermatum* NLPPya and all *Plasmopara halstedii* NLPs. The *Pl. halstedii* genome only encodes two NLPs that contain a complete cation-binding pocket. 17 out of the 19 PhalNLPs contain a functional (D-score>0.45) signal peptide, suggesting they are secreted. Phal06162 contains a 240 aa domain between the signal peptide and the start of the NEP1 domain, a feature not uncommon in this group of NLPs.

## Supplementary Figure 14

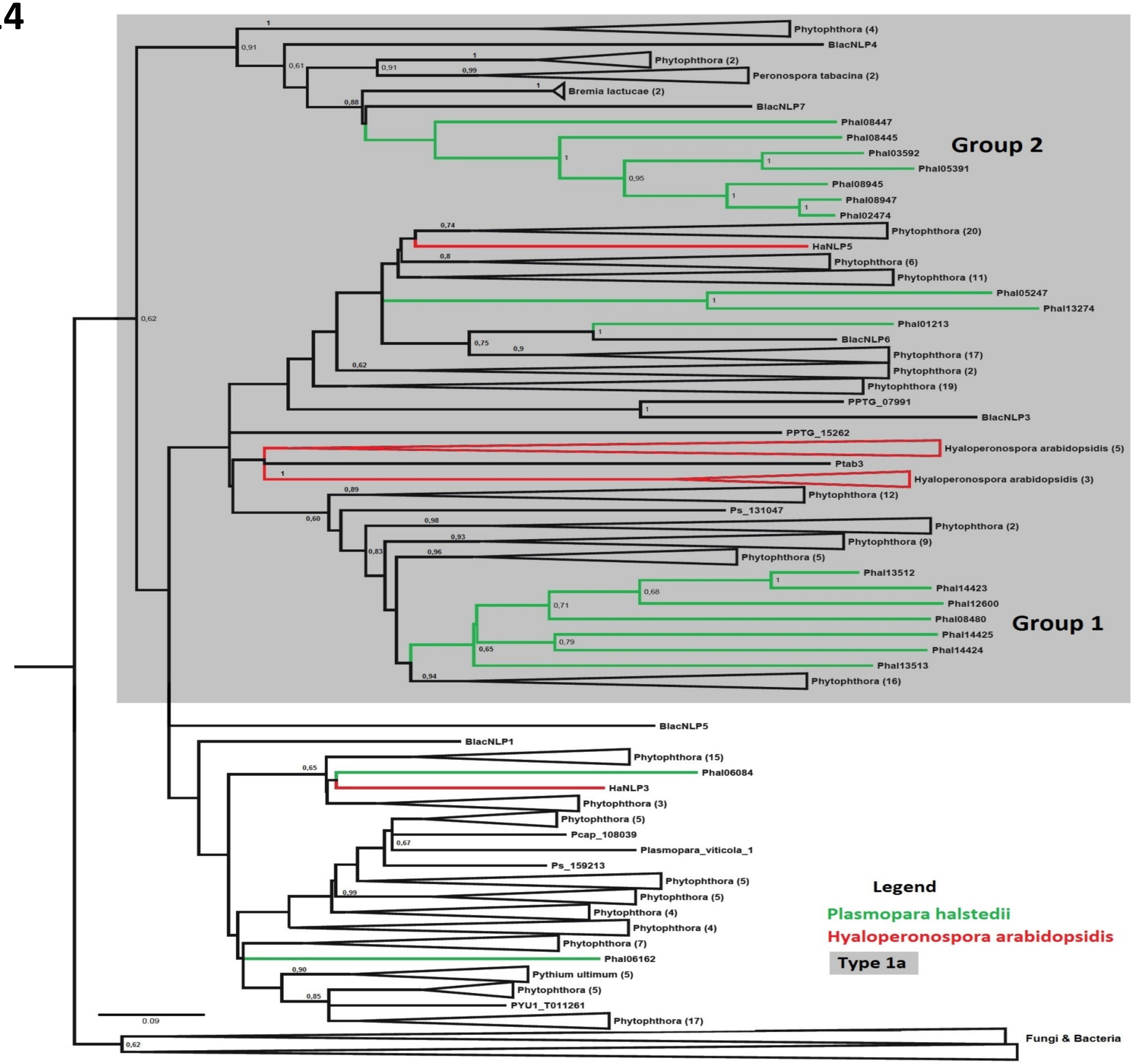


**Supplementary Figure 14:** Phylogeny of the 19 PhalNLPs compared with 378 type 1 NLPs of 91 different species (53). Neighbor Joining tree (Mega5, 100 Bootstraps). All signal peptides were removed before alignment. Only bootstrap values >0.6 are shown. 17 of the 19 PhalNLPs are of Type 1a, lacking the acidic cation binding pocket required for induction of necrosis. Two species-specific expansions can be observed, which were named group 1 and group 2. The type 1 NLP Phal06084 clusters together with the well-studied HaNLP3 (54).

# Supplementary Figure 15

***PhalPseudoNLP1* aligned with its closed relative (*Phal14423*)**

```
Phal14423        ATGAGCCCACGTGCCGTTCTTCTCGGAGCAATTGCTATCCTTGTTGCATCCCATGGCTTT 60
PhalPseudoNLP1   ATGAGCTCTCGCGCCATGCTTCTCGGAATTATTGCCATCTTTGCTGCTTCCTATGGTATA 60
                 ****** *:** ***.* *********. :***** *** *** ***:*** **** :*:

Phal14423        GCGAAATGGGCTGACGCTAACGTCTCGAAACACTCAGCAAAGTCACGAACCATTCCCGAC 120
PhalPseudoNLP1   GTGGAAAGGGCTGACGCTGATGTATCTGTACACCCAGAAG---------CTATCTCCTAC 111
                 * *.**:***********.* **.** .:**** ***.*.         * **  ** **

Phal14423        CATTACAACTTTGTAAATGTCAACGAAGTGCAGCCATTTAAACAGCGTAAACCAACTACG 180
PhalPseudoNLP1   CATTACAACTTTATAAATGTCAACGCAGTGCAGCCCTTTAAACAGCCTAAACCGACGACA 171
                 ************.************.*********.********** ******.** **.

Phal14423        GATTCTCAGAAATCTGCGATCAAGTACCAACCACGGCTTTACGTATTGGGTGCATGTTAT 240
PhalPseudoNLP1   GATTCTCAAAGATCTGCCATTAAATACCAACCACGGCTTTCCATTTCGAGCCGATGTCAT 231
                 ********.*.****** ** **.****************.*.*:* *.*   **** **

Phal14423        CCATATCCTGCAGTGCAGGCGGATGGATCACTAAGTGAGGGACTTCCCTGGAAAATCTTC 300
PhalPseudoNLP1   CCATATCCTGCAGTGCAGGCGAATGGGTCACTAAATGAGGGACTTCCCTGGAAAGTCTTC 291
                 *********************.****.*******.*******************.*****

Phal14423        TTTCAGTCTCGATGTAATGGATCTCCCCGCGGTTCCCAAATTTACTCGCGATCCGATTGG 360
PhalPseudoNLP1   ---CAGTATCGATGTCATGGATCTCCCCACGGTTCTCAAATTTACTCGTGATCCGATTGG 348
                    ****.*******.************.****** ************ **********

Phal14423        TACCGAGGCAAGTGGGCCATTATGTACGCGTGGTACCTTCCGAGAGCCCTGGACCAAAAC 420
PhalPseudoNLP1   TACCGAGGCAAGTGGGCCATTATGTACGCGTGGTACCTTCCGAAGGCCTTGGACCAAACT 408
                 *******************************************..*** *********.

Phal14423        ATCTGGGATGTTGACGGCCATAGTCATTTTTGGACCTTTGTCGTTGTCTGGACAGACAGC 480
PhalPseudoNLP1   AACTGGTTGGTTGACGGCCATTGTCACTTTTGGACCTGGGTCGTTGTCTGGACAGACAGC 468
                 *:**** : ************:**** **********  *********************

Phal14423        CCGGATCCGGATGTCTCGACTATTCTCGCTGCATCAACCTCTGGTGTCGGCAACAGAATT 540
PhalPseudoNLP1   CCTGATCCGAATGTCTCGACCATTCTCGCTGGATCAACAGCTGGCATCGGCGACAAAATC 528
                 ** ******.********** ********** ******. **** .*****.***.***

Phal14423        AAAAAATATTCACCACTCAAGTCAAAGTATCTTCTTAACCACAAGTCTTTGAAGGTCAAG 600
PhalPseudoNLP1   AAGAAATACCTACCACTAGAGTCAAAGTATCTTCTTGACAACAAGTCTTTGAAGGTCAAA 588
                 **.*****   ******..*****************.**.*******************.

Phal14423        CTACACGAAAATCATTTTTGGGATAAGACTGGACTCAAGCTGACAGAAAAAGTTGGTGAG 660
PhalPseudoNLP1   ACACATGAAAGTTATTTATGGAAGAGGACTGGAGTCAAGGTTACCGAAAAAGTCGGT--A 646
                 . *** ****.* ****:***.* *.******* ***** * **.******** ***  .

Phal14423        TTGCAGACTCTCATAACTTGGGAGCAGCTTACAGATGAGGCTCGAGCAGCGCTATCTTCT 720
PhalPseudoNLP1   CTGCAGACTCTCATTACTTGGGAGCAGCTTACAGATGAGGCTCGAACAGCGCTATCATCT 706
                  *************:******************************.**********:***

Phal14423        GTTCATTACTCTGATTGGCGTATGGAGTCGCCAATTCAAGACAAGTGGTTTCGTCCAATG 780
PhalPseudoNLP1   TAGCATTACCCGTATTGGCGTATGGAGTCGCCGATTCAAAACAAGCGTTTTAATCGGACG 766
                  : ****** *  *******************.******.***** * ***..** .* *

Phal14423        CTAAGTAGTGCTTATCCATTTTAG 804
PhalPseudoNLP1   CTGAAAGCTGCTTATCCATTCTAG 790
                 **.*.:. ************ ***
```

**Supplementary Figure 15:** Multiple alignment of *PhalPseudoNLP1* and its closest relative *Phal14423*. *PhalPseudoNLP1* encodes a premature stop codon at position 337-339 (indicated in Bold).

# Supplementary Figure 16

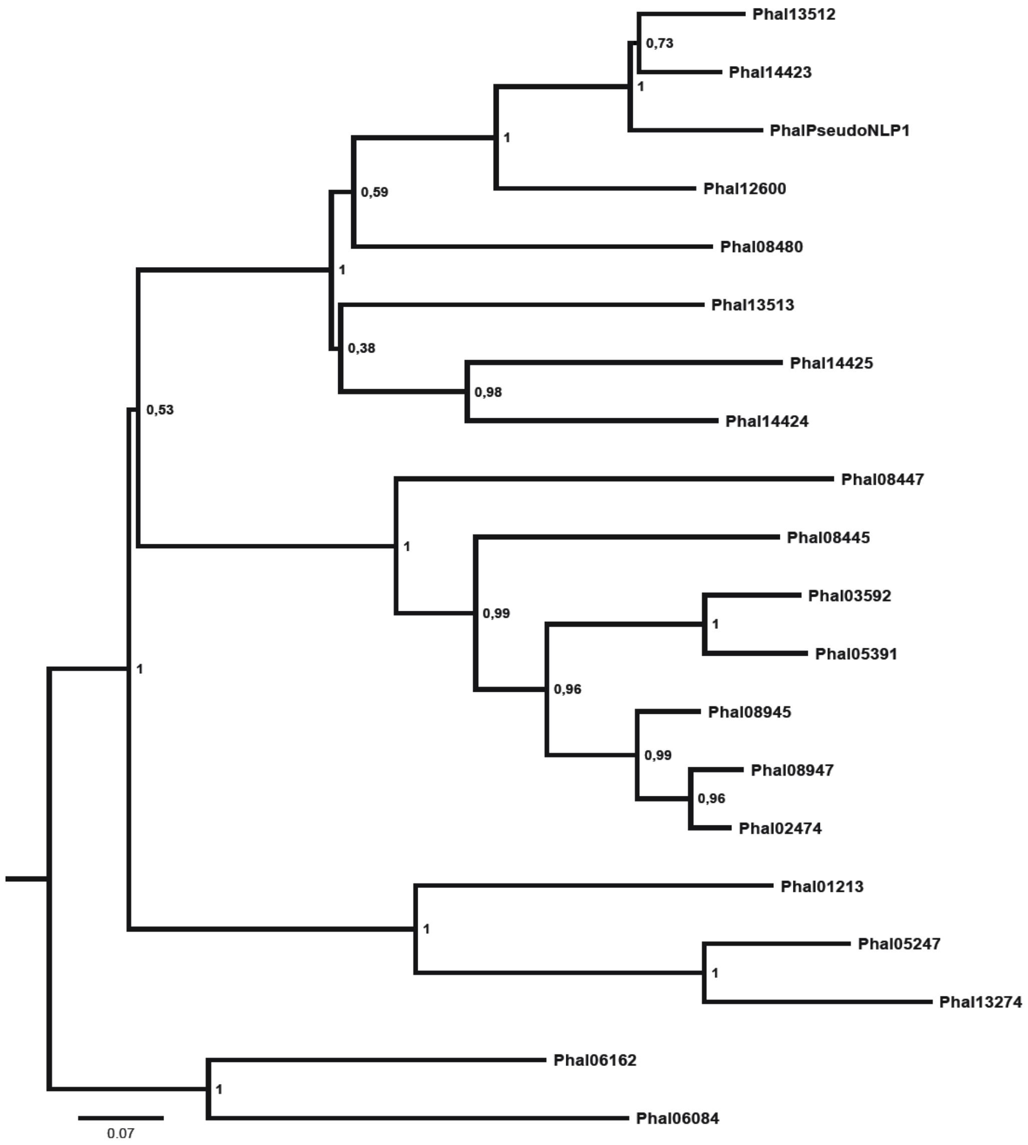


**Supplementary Figure 16:** DNA-based Maximum Likelyhood tree of all PhalNLP genes, including the pseudogene. The tree is made using Mega5 (100 bootstraps).

## Supplementary Figure 17

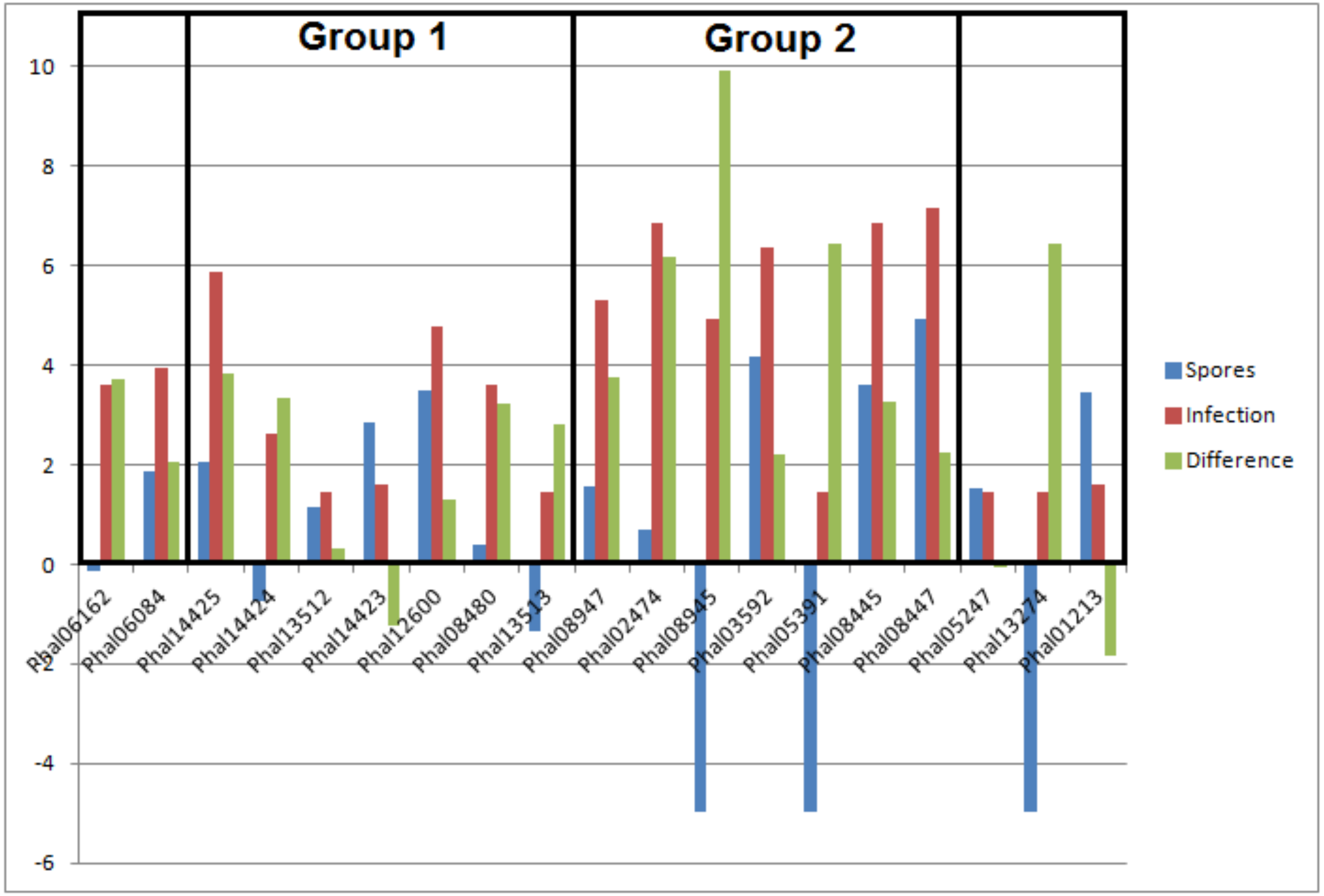


**Supplementary Figure 17:** Expression of the PhalNLPs in two different developmental stages. For clarity, the difference, and thus the total up- or down-regulation is included. Values have been derived from the RNA-seq expression profiling (See methods).

# Supplementary Figure 18

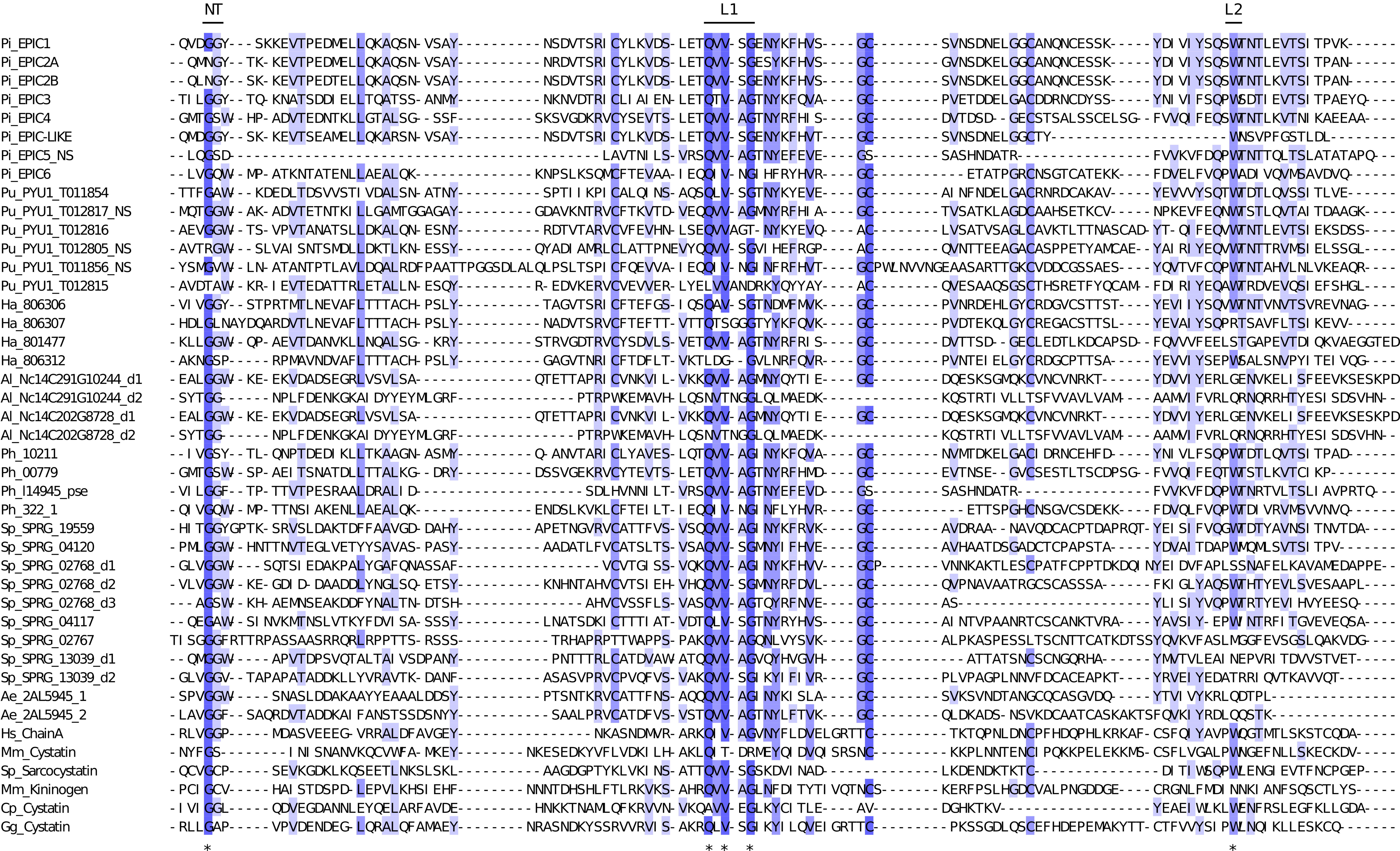

**Supplementary Figure 18:** Sequence alignment of Kazal-like domains from seven pathogenic oomycetes. Multiple sequence alignment of 169 Kazal-like domains of seven pathogenic oomycetes. Out of the 169 oomycete Kazal-domains of oomycetes, 60 are from *Phytophthora infestans* (Pi), 37 are from *Pythium ultimum* (Pu), 4 are from *Hyaloperonospora arabidopsidis* (Ha), 8 are from *Albugo laibachii* (Al), 25 are from *Saprolegnia parasitica* (Sp), 30 are from *Plasmopara halstedii* (Ph) and 5 are from *Aphanomyces euteiches* (Ae). The alignment also includes 8 additional known Kazal-like domains present in 2 serine protease inhibitors from crayfish and a protozoan parasite species, respectively. Out of the 8 additional Kazal-like inhibitors, 4 are from the crayfish *Pacifastus leniusculus* (PAPI-1_d1-d2, CAA56043) and 4 from the apicomplexan protozoan parasite *Toxoplasma gondii* (PI-1_d1-d2, AF121778). The amino acid residues that defined the Kazal-like family protease inhibitor domain are marked with asterisks (bottom). The conserved cysteines and their position are numbered in the alignment (top). Cysteine positions three and six shown in grey are missing in some protease inhibitors domains of *P. infestans*. The first suffix indicates the number of the Kazal-like domain from left to right of the C-terminal effector domain in multidomain proteins. The second suffix indicates the P1 amino acid active residue, which is the central to the specificity of Kazal-like inhibitors. The third suffix “NS” when present indicate protease inhibitor domains from proteins that are not predicted to be secreted.

Supplementary Figure 19

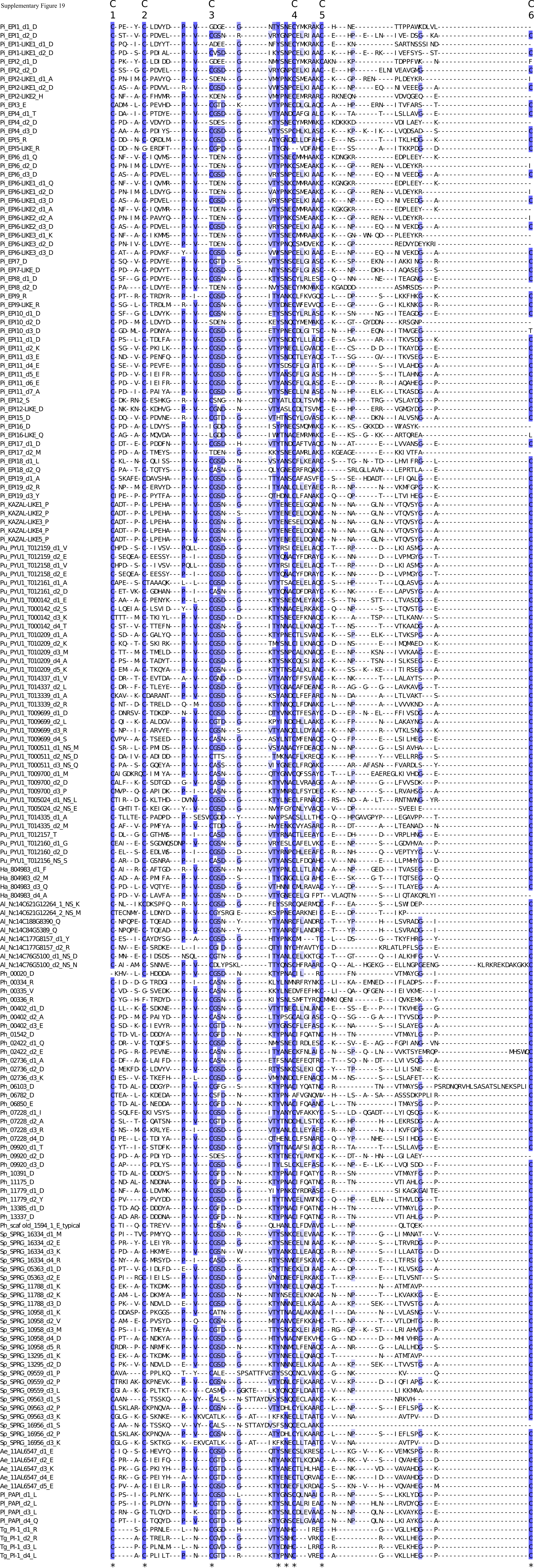

**Supplementary Figure 19. Sequence alignment of cystatin-like domains of seven pathogenic oomycetes.**

Multiple sequence alignment of 38 cystatin-like domains present in 30 cysteine protease inhibitors (EPICs) of seven pathogenic oomycetes. Out of the 38 oomycete cystatin-like domains, 8 are from *Phytophthora infestans* (Pi), 6 are from *Pythium ultimum* (Pu), 4 are from *Hyaloperonospora arabidopsidis* (Ha), 4 are from *Albugo laibachii* (Al), 9 are from *Saprolegnia parasitica* (Sp), 1 is from *Plasmopara halstedii* (Ph) and 6 are from *Aphanomyces euteiches* (Ae). The alignment also includes 6 cystatin-like domains present in 6 cysteine protease inhibitors from plants (*Carica papaya* Cp Cystatin gi|311505), from animals (insect *Sarcophaga peregrina* Sp_ Sarcocystatin gi|399335, chicken Gg_Cystatin P01038, mouse Mm_Cystatin gi|6226846 Mm_Kininogen gi|12643495, human Hs_ Chain A gi14278690). The proposed active-site residues in cystatins, including the N-terminal trunk (NT), first binding loop (L1) and second binding loop (L2) are indicated in the sequence with a bar (top). The amino acids that define cystatins are marked with asterisks (bottom). The first suffix indicates the number of the cystatin-like domain from left to right of the C-terminal effector domain in multidomain proteins. The second suffix “NS” was added to protease inhibitor domains from proteins that were not predicted to be secreted.

# Supplementary Figure 20

| | | |
|---|---|---|
| CRN predictions on the ORFs of *Pl. halstedii* genome | BlastP | 476 |
| | BlastP+HMM (full length/Haas) | 200/139 |
| | BlastP + Both HMM | 125 |
| | BlastP + Both HMM + SignalP v4 (Probability: 0.6) | 33 |
| | BlastP + Both HMM + Manual | 55 |
| | BlastP + Both HMM + Manual + SignalP v4 (Probability: 0.6) | 16 |
| CRN predictions on the protein sequences of *Pl. halstedii* genome | Constructing *Pl. halstedii* CRNs HMM | 49 |
| | Total non-overlapping CRNs in *Pl. halstedii* genome | 77 |

**Supplementary Figure 20:** Table is representing the number of candidate CRNs predicted in the current study.

## Supplementary Figure 21

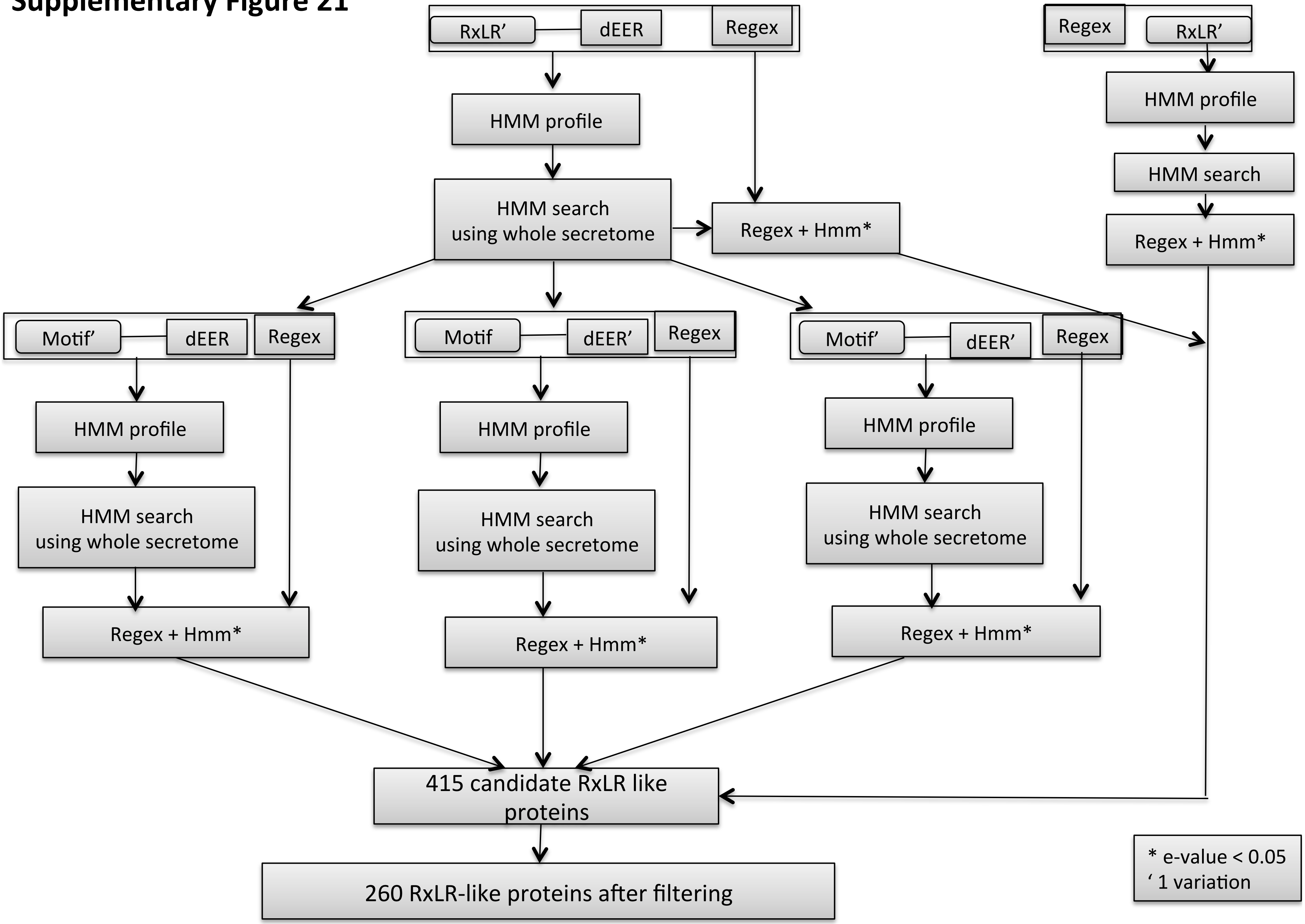


**Supplementary Figure 21.** Pipeline used for predicting RxLR-like proteins form the *P. halstedii* protein sequences.

## Supplementary Figure 22

A

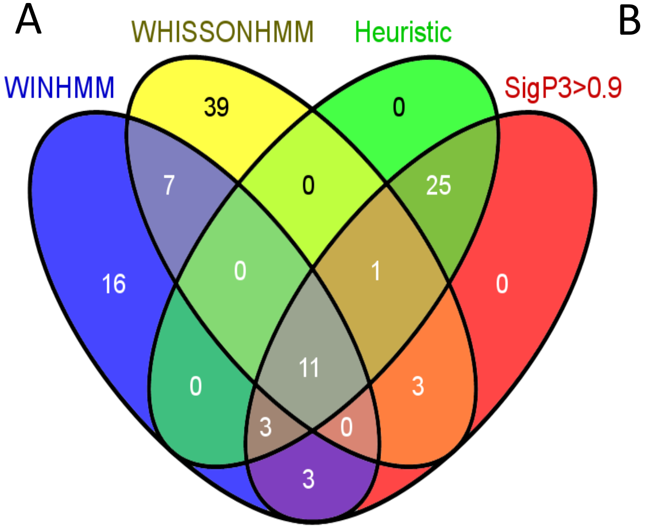


B

| | Method | Number (SignalP3 secreted) |
|---|---|---|
| RxLR predictions on ORFs of *Pl. halstedii* genome | Heuristic (SigP + RxLR-dEER regex) | 40 (40) |
| | HMM (from Whisson *et al.* 2007) | 61 (15) |
| | HMM (from Win *et al.* 2007) | 40 (17) |
| | Heuristic only | 25 (25) |
| | HMM (Whisson only) | 42 (3) |
| | Hmm (Win only) | 19 (3) |
| | Agreed by heuristic and both HMM | 11 (11) |
| | Agreed by heuristic and >= 1 HMM | 15 (15) |
| | Total by any method | 108 (46) |
| predictions on protein sequences of *Pl. halstedii* genome | Using *Pl. halstedii* HMM using 4 iterations | 260 |
| | Total non-overlapping RxLR-like proteins in *Pl. halstedii* genome | 274 |

Whisson SC, et al. 2007. A translocation signal for delivery of oomycete effector proteins into host plant cells. Nature 450: 115-118. doi: 10.1038, nature06203
Win J, et al. 2007. Adaptive evolution has targeted the C-terminal domain of the RXLR effectors of plant pathogenic oomycetes. Plant Cell 19: 2349-2369. doi: 10.1105/tpc.107.051037

C

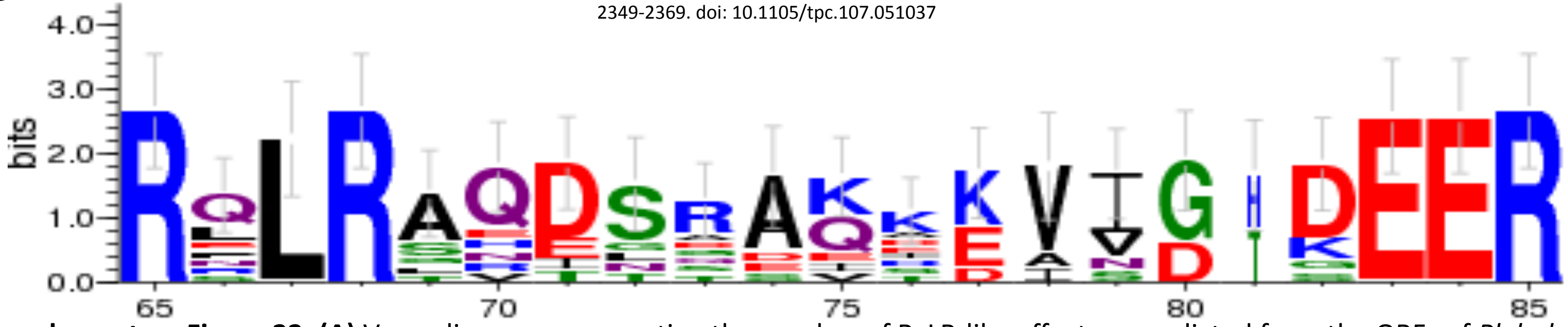


**Supplementary Figure 22: (A)** Venn-diagram representing the number of RxLR-like effectors predicted from the ORFs of *Pl. halstedii*. **(B)** RxLR-like effector predictions using different methods **(C)** Weblogo visualization of the RxLR-dEER motif of the RxLR-like effectors.

Ortholog 1 Supplementary Figure 23

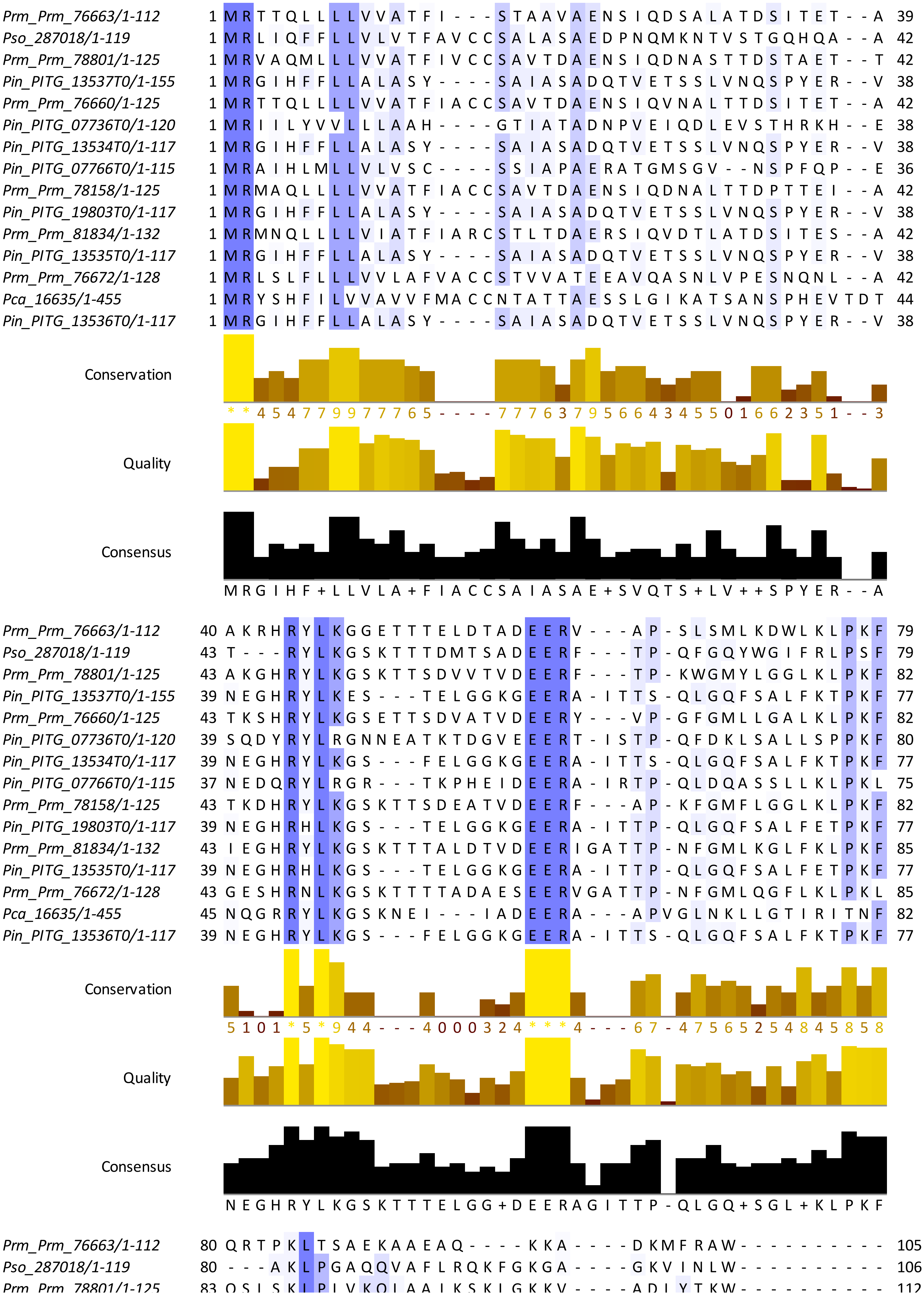

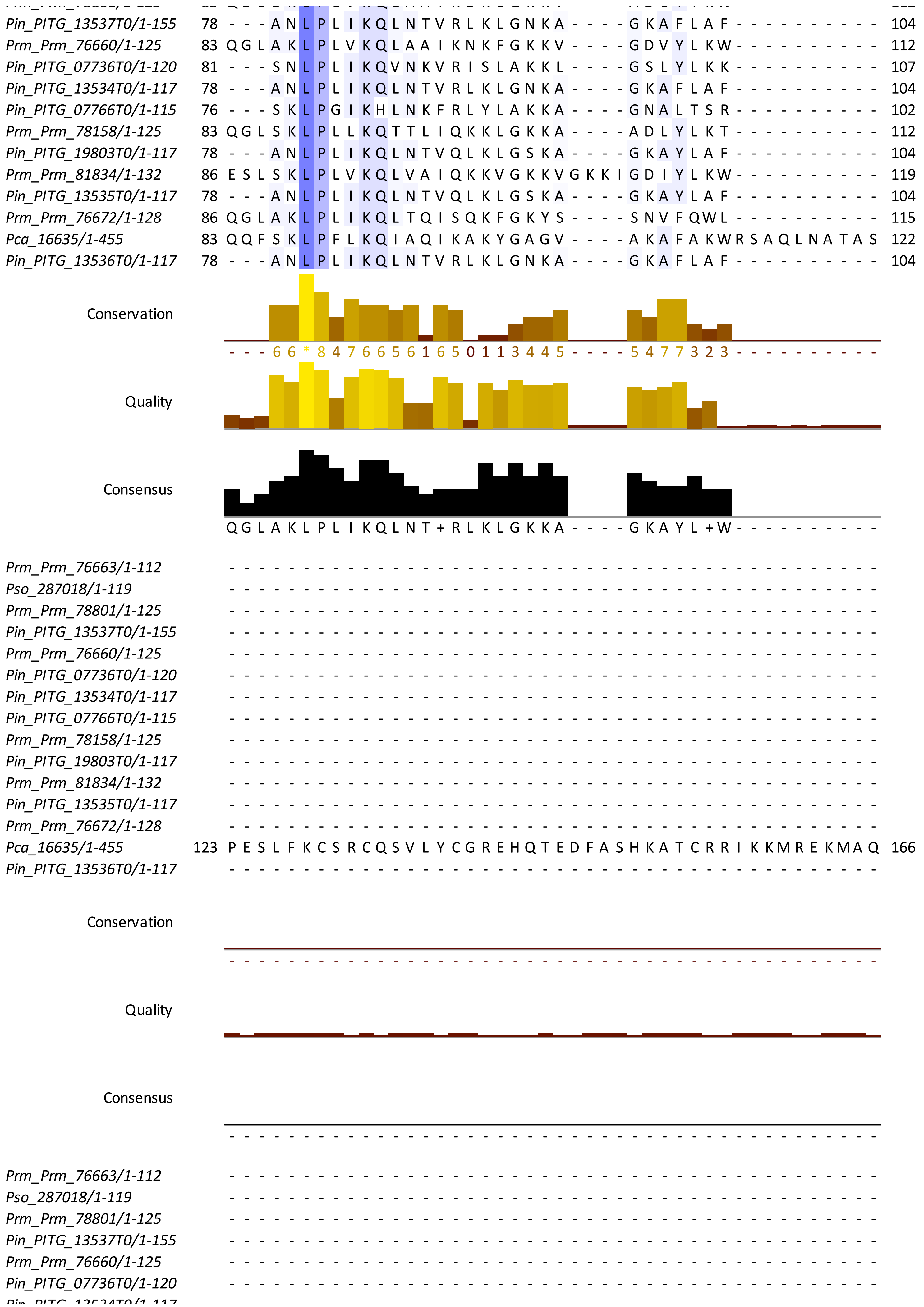
Pin_PITG_13537T0/1-155 78 ---ANLPLIKQLNTVRLKLGNKA----GKAFLAF---------- 104
Prm_Prm_76660/1-125 83 QGLAKLPLVKQLAAIKNKFGKKV----GDVYLKW---------- 112
Pin_PITG_07736T0/1-120 81 ---SNLPLIKQVNKVRISLAKKL----GSLYLKK---------- 107
Pin_PITG_13534T0/1-117 78 ---ANLPLIKQLNTVRLKLGNKA----GKAFLAF---------- 104
Pin_PITG_07766T0/1-115 76 ---SKLPGIKHLNKFRLYLAKKA----GNALTSR---------- 102
Prm_Prm_78158/1-125 83 QGLSKLPLLKQTTLIQKKLGKKA----ADLYLKT---------- 112
Pin_PITG_19803T0/1-117 78 ---ANLPLIKQLNTVQLKLGSKA----GKAYLAF---------- 104
Prm_Prm_81834/1-132 86 ESLSKLPLVKQLVAIQKKVGKKVGKKIGDIYLKW---------- 119
Pin_PITG_13535T0/1-117 78 ---ANLPLIKQLNTVQLKLGSKA----GKAYLAF---------- 104
Prm_Prm_76672/1-128 86 QGLAKLPLIKQLTQISQKFGKYS----SNVFQWL---------- 115
Pca_16635/1-455 83 QQFSKLPFLKQIAQIKAKYGAGV----AKAFAKWRSAQLNATAS 122
Pin_PITG_13536T0/1-117 78 ---ANLPLIKQLNTVRLKLGNKA----GKAFLAF---------- 104
Conservation
---66*8476656165011344 5----5477323----------
Quality
Consensus
QGLAKLPLIKQLNT+RLKLGKKA----GKAYL+W----------
Prm_Prm_76663/1-112
Pso_287018/1-119
Prm_Prm_78801/1-125
Pin_PITG_13537T0/1-155
Prm_Prm_76660/1-125
Pin_PITG_07736T0/1-120
Pin_PITG_13534T0/1-117
Pin_PITG_07766T0/1-115
Prm_Prm_78158/1-125
Pin_PITG_19803T0/1-117
Prm_Prm_81834/1-132
Pin_PITG_13535T0/1-117
Prm_Prm_76672/1-128
Pca_16635/1-455 123 PESLFKCSRCQSVLYCGREHQTEDFASHKATCRRIKKMREKMAQ 166
Pin_PITG_13536T0/1-117
Conservation
Quality
Consensus
Prm_Prm_76663/1-112
Pso_287018/1-119
Prm_Prm_78801/1-125
Pin_PITG_13537T0/1-155
Prm_Prm_76660/1-125
Pin_PITG_07736T0/1-120

| | | | |
|---|---|---|---|
| Pin_PITG_13534T0/1-117 | | - - - - - - - - - - - - - - - - - - - - - - - - - - - - - - - - - - - - - - - - - - - - | |
| Pin_PITG_07766T0/1-115 | | - - - - - - - - - - - - - - - - - - - - - - - - - - - - - - - - - - - - - - - - - - - - | |
| Prm_Prm_78158/1-125 | | - - - - - - - - - - - - - - - - - - - - - - - - - - - - - - - - - - - - - - - - - - - - | |
| Pin_PITG_19803T0/1-117 | | - - - - - - - - - - - - - - - - - - - - - - - - - - - - - - - - - - - - - - - - - - - - | |
| Prm_Prm_81834/1-132 | | - - - - - - - - - - - - - - - - - - - - - - - - - - - - - - - - - - - - - - - - - - - - | |
| Pin_PITG_13535T0/1-117 | | - - - - - - - - - - - - - - - - - - - - - - - - - - - - - - - - - - - - - - - - - - - - | |
| Prm_Prm_76672/1-128 | | - - - - - - - - - - - - - - - - - - - - - - - - - - - - - - - - - - - - - - - - - - - - | |
| Pca_16635/1-455 | 167 | E E E I V R N S T E D F W T P A N A F E T H V G H F W G L R G T R P Y M T A K L E V I R | 210 |
| Pin_PITG_13536T0/1-117 | | - - - - - - - - - - - - - - - - - - - - - - - - - - - - - - - - - - - - - - - - - - - - | |
| Conservation | | - - - - - - - - - - - - - - - - - - - - - - - - - - - - - - - - - - - - - - - - - - - - | |

Quality

| | | | |
|---|---|---|---|
| Consensus | | - - - - - - - - - - - - - - - - - - - - - - - - - - - - - - - - - - - - - - - - - - - - | |
| Prm_Prm_76663/1-112 | | - - - - - - - - - - - - - - - - - - - - - - - - - - - - - - - - - - - - - - - - - - - - | |
| Pso_287018/1-119 | | - - - - - - - - - - - - - - - - - - - - - - - - - - - - - - - - - - - - - - - - - - - - | |
| Prm_Prm_78801/1-125 | | - - - - - - - - - - - - - - - - - - - - - - - - - - - - - - - - - - - - - - - - - - - - | |
| Pin_PITG_13537T0/1-155 | | - - - - - - - - - - - - - - - - - - - - - - - - - - - - - - - - - - - - - - - - - - - - | |
| Prm_Prm_76660/1-125 | | - - - - - - - - - - - - - - - - - - - - - - - - - - - - - - - - - - - - - - - - - - - - | |
| Pin_PITG_07736T0/1-120 | | - - - - - - - - - - - - - - - - - - - - - - - - - - - - - - - - - - - - - - - - - - - - | |
| Pin_PITG_13534T0/1-117 | | - - - - - - - - - - - - - - - - - - - - - - - - - - - - - - - - - - - - - - - - - - - - | |
| Pin_PITG_07766T0/1-115 | | - - - - - - - - - - - - - - - - - - - - - - - - - - - - - - - - - - - - - - - - - - - - | |
| Prm_Prm_78158/1-125 | | - - - - - - - - - - - - - - - - - - - - - - - - - - - - - - - - - - - - - - - - - - - - | |
| Pin_PITG_19803T0/1-117 | | - - - - - - - - - - - - - - - - - - - - - - - - - - - - - - - - - - - - - - - - - - - - | |
| Prm_Prm_81834/1-132 | | - - - - - - - - - - - - - - - - - - - - - - - - - - - - - - - - - - - - - - - - - - - - | |
| Pin_PITG_13535T0/1-117 | | - - - - - - - - - - - - - - - - - - - - - - - - - - - - - - - - - - - - - - - - - - - - | |
| Prm_Prm_76672/1-128 | | - - - - - - - - - - - - - - - - - - - - - - - - - - - - - - - - - - - - - - - - - - - - | |
| Pca_16635/1-455 | 211 | A L S T I P S R P A I E A A L A E A M D S M R L C R V D N L G I R E V I P T L M L I L D | 254 |
| Pin_PITG_13536T0/1-117 | | - - - - - - - - - - - - - - - - - - - - - - - - - - - - - - - - - - - - - - - - - - - - | |
| Conservation | | - - - - - - - - - - - - - - - - - - - - - - - - - - - - - - - - - - - - - - - - - - - - | |

Quality

| | | | |
|---|---|---|---|
| Consensus | | - - - - - - - - - - - - - - - - - - - - - - - - - - - - - - - - - - - - - - - - - - - - | |
| Prm_Prm_76663/1-112 | | - - - - - - - - - - - - - - - - - - - - - - - - - - - - - - - - - - - - - - - - - - - - | |
| Pso_287018/1-119 | | - - - - - - - - - - - - - - - - - - - - - - - - - - - - - - - - - - - - - - - - - - - - | |
| Prm_Prm_78801/1-125 | | - - - - - - - - - - - - - - - - - - - - - - - - - - - - - - - - - - - - - - - - - - - - | |
| Pin_PITG_13537T0/1-155 | | - - - - - - - - - - - - - - - - - - - - - - - - - - - - - - - - - - - - - - - - - - - - | |
| Prm_Prm_76660/1-125 | | - - - - - - - - - - - - - - - - - - - - - - - - - - - - - - - - - - - - - - - - - - - - | |
| Pin_PITG_07736T0/1-120 | | - - - - - - - - - - - - - - - - - - - - - - - - - - - - - - - - - - - - - - - - - - - - | |
| Pin_PITG_13534T0/1-117 | | - - - - - - - - - - - - - - - - - - - - - - - - - - - - - - - - - - - - - - - - - - - - | |
| Pin_PITG_07766T0/1-115 | | - - - - - - - - - - - - - - - - - - - - - - - - - - - - - - - - - - - - - - - - - - - - | |
| Prm_Prm_78158/1-125 | | - - - - - - - - - - - - - - - - - - - - - - - - - - - - - - - - - - - - - - - - - - - - | |
| Pin_PITG_19803T0/1-117 | | - - - - - - - - - - - - - - - - - - - - - - - - - - - - - - - - - - - - - - - - - - - - | |

```
Prm_Prm_81834/1-132          - - - - - - - - - - - - - - - - - - - - - - - - - - - - - - - - - - - - - - - - - - - -
Pin_PITG_13535T0/1-117       - - - - - - - - - - - - - - - - - - - - - - - - - - - - - - - - - - - - - - - - - - - -
Prm_Prm_76672/1-128          - - - - - - - - - - - - - - - - - - - - - - - - - - - - - - - - - - - - - - - - - - - -
Pca_16635/1-455          255 Q Y Q E A Y D F I K W Y A T T G D D D H Y D W G N M D L P F L D V R N A D M T E E I P G 298
Pin_PITG_13536T0/1-117       - - - - - - - - - - - - - - - - - - - - - - - - - - - - - - - - - - - - - - - - - - - -

                Conservation
                             - - - - - - - - - - - - - - - - - - - - - - - - - - - - - - - - - - - - - - - - - - - -

                     Quality

                   Consensus
                             - - - - - - - - - - - - - - - - - - - - - - - - - - - - - - - - - - - - - - - - - - - -

Prm_Prm_76663/1-112          - - - - - - - - - - - - - - - - - - - - - - - - - - - - - - - - - - - - - - - - - - - -
Pso_287018/1-119             - - - - - - - - - - - - - - - - - - - - - - - - - - - - - - - - - - - - - - - - - - - -
Prm_Prm_78801/1-125          - - - - - - - - - - - - - - - - - - - - - - - - - - - - - - - - - - - - - - - - - - - -
Pin_PITG_13537T0/1-155       - - - - - - - - - - - - - - - - - - - - - - - - - - - - - - - - - - - - - - - - - - - -
Prm_Prm_76660/1-125          - - - - - - - - - - - - - - - - - - - - - - - - - - - - - - - - - - - - - - - - - - - -
Pin_PITG_07736T0/1-120       - - - - - - - - - - - - - - - - - - - - - - - - - - - - - - - - - - - - - - - - - - - -
Pin_PITG_13534T0/1-117       - - - - - - - - - - - - - - - - - - - - - - - - - - - - - - - - - - - - - - - - - - - -
Pin_PITG_07766T0/1-115       - - - - - - - - - - - - - - - - - - - - - - - - - - - - - - - - - - - - - - - - - - - -
Prm_Prm_78158/1-125          - - - - - - - - - - - - - - - - - - - - - - - - - - - - - - - - - - - - - - - - - - - -
Pin_PITG_19803T0/1-117       - - - - - - - - - - - - - - - - - - - - - - - - - - - - - - - - - - - - - - - - - - - -
Prm_Prm_81834/1-132          - - - - - - - - - - - - - - - - - - - - - - - - - - - - - - - - - - - - - - - - - - - -
Pin_PITG_13535T0/1-117       - - - - - - - - - - - - - - - - - - - - - - - - - - - - - - - - - - - - - - - - - - - -
Prm_Prm_76672/1-128          - - - - - - - - - - - - - - - - - - - - - - - - - - - - - - - - - - - - - - - - - - - -
Pca_16635/1-455          299 S M R N G R D V F F M S N L A Y I K L M L A K T V K D A I N A H N Q G R T S L P S I E S 342
Pin_PITG_13536T0/1-117       - - - - - - - - - - - - - - - - - - - - - - - - - - - - - - - - - - - - - - - - - - - -

                Conservation
                             - - - - - - - - - - - - - - - - - - - - - - - - - - - - - - - - - - - - - - - - - - - -

                     Quality

                   Consensus
                             - - - - - - - - - - - - - - - - - - - - - - - - - - - - - - - - - - - - - - - - - - - -

Prm_Prm_76663/1-112          - - - - - - - - - - - - - - - - - - - - - - - - - - - - - - - - - - - - - - - - - - - -
Pso_287018/1-119             - - - - - - - - - - - - - - - - - - - - - - - - - - - - - - - - - - - - - - - - - - - -
Prm_Prm_78801/1-125          - - - - - - - - - - - - - - - - - - - - - - - - - - - - - - - - - - - - - - - - - - - -
Pin_PITG_13537T0/1-155       - - - - - - - - - - - - - - - - - - - - - - - - - - - - - - - - - - - - - - - - - - - -
Prm_Prm_76660/1-125          - - - - - - - - - - - - - - - - - - - - - - - - - - - - - - - - - - - - - - - - - - - -
Pin_PITG_07736T0/1-120       - - - - - - - - - - - - - - - - - - - - - - - - - - - - - - - - - - - - - - - - - - - -
Pin_PITG_13534T0/1-117       - - - - - - - - - - - - - - - - - - - - - - - - - - - - - - - - - - - - - - - - - - - -
Pin_PITG_07766T0/1-115       - - - - - - - - - - - - - - - - - - - - - - - - - - - - - - - - - - - - - - - - - - - -
Prm_Prm_78158/1-125          - - - - - - - - - - - - - - - - - - - - - - - - - - - - - - - - - - - - - - - - - - - -
Pin_PITG_19803T0/1-117       - - - - - - - - - - - - - - - - - - - - - - - - - - - - - - - - - - - - - - - - - - - -
Prm_Prm_81834/1-132          - - - - - - - - - - - - - - - - - - - - - - - - - - - - - - - - - - - - - - - - - - - -
Pin_PITG_13535T0/1-117       - - - - - - - - - - - - - - - - - - - - - - - - - - - - - - - - - - - - - - - - - - - -
Prm_Prm_76672/1-128          - - - - - - - - - - - - - - - - - - - - - - - - - - - - - - - - - - - - - - - - - - - -
Pca_16635/1-455          343 L R A V V A P N G T M R S V T E L K R L H K K L T S Q M H A A S S L A H F Q N Q H F W N 386
```

```
Pin_PITG_13536T0/1-117        --------------------------------------------

Conservation

                              --------------------------------------------

Quality

Consensus
                              --------------------------------------------

Prm_Prm_76663/1-112           --------------------------------------------
Pso_287018/1-119              --------------------------------------------
Prm_Prm_78801/1-125           --------------------------------------------
Pin_PITG_13537T0/1-155        --------------------------------------------
Prm_Prm_76660/1-125           --------------------------------------------
Pin_PITG_07736T0/1-120        --------------------------------------------
Pin_PITG_13534T0/1-117        --------------------------------------------
Pin_PITG_07766T0/1-115        --------------------------------------------
Prm_Prm_78158/1-125           --------------------------------------------
Pin_PITG_19803T0/1-117        --------------------------------------------
Prm_Prm_81834/1-132           --------------------------------------------
Pin_PITG_13535T0/1-117        --------------------------------------------
Prm_Prm_76672/1-128           --------------------------------------------
Pca_16635/1-455           387 GVLDPTPIVQAPKPPHYAPGDINQVKLWVLQSAMLWRDHHDFIR 430
Pin_PITG_13536T0/1-117        --------------------------------------------

Conservation

                              --------------------------------------------

Quality

Consensus
                              --------------------------------------------

Prm_Prm_76663/1-112       106 ------------------MNLRNNP------------------- 112
Pso_287018/1-119          107 ------------FRLVRRSSKNSGI------------------- 119
Prm_Prm_78801/1-125       113 ------------VKKRYESNPQNFM------------------- 125
Pin_PITG_13537T0/1-155    105 ------------VKKRSNGNMNNFYVDSRVCEGNKTRRKRNIRF 136
Prm_Prm_76660/1-125       113 ------------VKKRYNSNPQNFM------------------- 125
Pin_PITG_07736T0/1-120    108 ------------VRKAYERNPNNFV------------------- 120
Pin_PITG_13534T0/1-117    105 ------------VKKRNNGNMNNFM------------------- 117
Pin_PITG_07766T0/1-115    103 ------------MKKRYDSNPNNFI------------------- 115
Prm_Prm_78158/1-125       113 ------------PKKRYEVNLQNFI------------------- 125
Pin_PITG_19803T0/1-117    105 ------------VKKRNNGNMNNFM------------------- 117
Prm_Prm_81834/1-132       120 ------------AKKRYESNPQNFM------------------- 132
Pin_PITG_13535T0/1-117    105 ------------VKKRNNGNMNNFM------------------- 117
Prm_Prm_76672/1-128       116 ------------MKKFRIYNTNANI------------------- 128
Pca_16635/1-455           431 EKRSAYPDYLPEMPQRGIQQLVTFP------------------- 455
Pin_PITG_13536T0/1-117    105 ------------VKKRNNGNMNNFM------------------- 117

Conservation
```

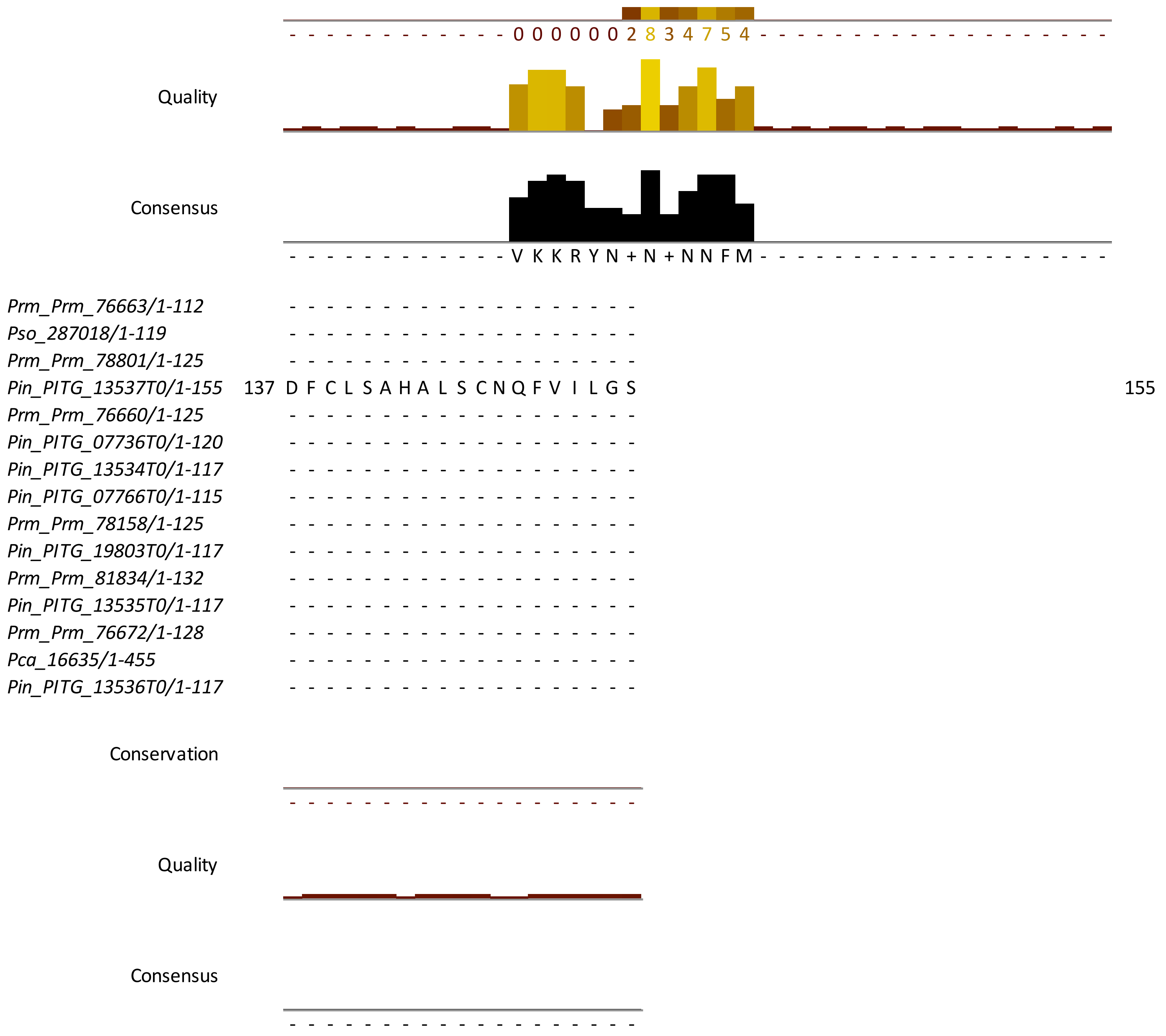
- - - - - - - - - - - - 0 0 0 0 0 0 2 8 3 4 7 5 4 - - - - - - - - - - - - - - - - - - -
Quality
Consensus
- - - - - - - - - - - - V K K R Y N + N + N N F M - - - - - - - - - - - - - - - - - - -
Prm_Prm_76663/1-112 - - - - - - - - - - - - - - - - - - -
Pso_287018/1-119 - - - - - - - - - - - - - - - - - - -
Prm_Prm_78801/1-125 - - - - - - - - - - - - - - - - - - -
Pin_PITG_13537T0/1-155 137 D F C L S A H A L S C N Q F V I L G S 155
Prm_Prm_76660/1-125 - - - - - - - - - - - - - - - - - - -
Pin_PITG_07736T0/1-120 - - - - - - - - - - - - - - - - - - -
Pin_PITG_13534T0/1-117 - - - - - - - - - - - - - - - - - - -
Pin_PITG_07766T0/1-115 - - - - - - - - - - - - - - - - - - -
Prm_Prm_78158/1-125 - - - - - - - - - - - - - - - - - - -
Pin_PITG_19803T0/1-117 - - - - - - - - - - - - - - - - - - -
Prm_Prm_81834/1-132 - - - - - - - - - - - - - - - - - - -
Pin_PITG_13535T0/1-117 - - - - - - - - - - - - - - - - - - -
Prm_Prm_76672/1-128 - - - - - - - - - - - - - - - - - - -
Pca_16635/1-455 - - - - - - - - - - - - - - - - - - -
Pin_PITG_13536T0/1-117 - - - - - - - - - - - - - - - - - - -
Conservation
- - - - - - - - - - - - - - - - - - -
Quality
Consensus
- - - - - - - - - - - - - - - - - - -

Ortholog 2

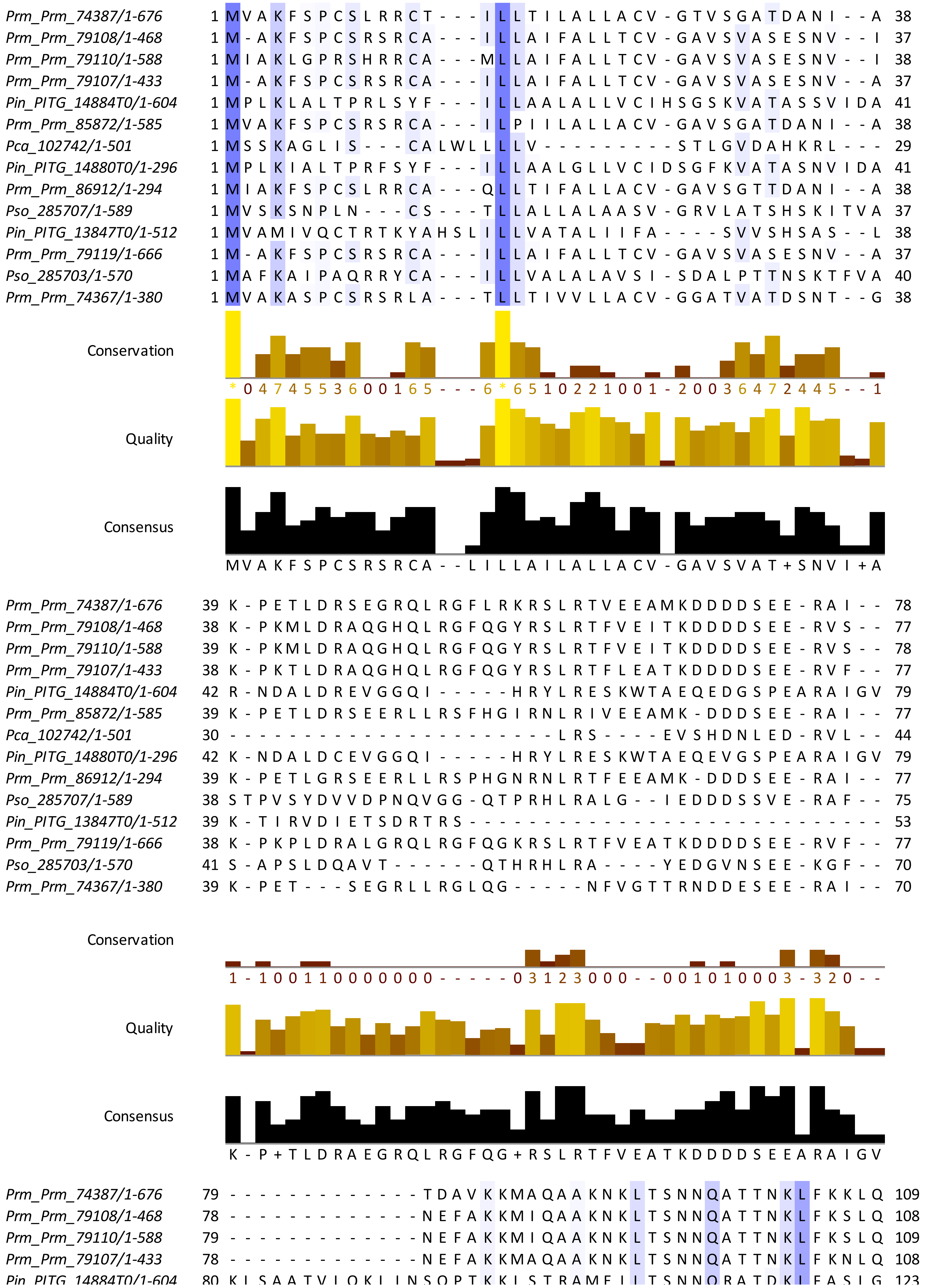
Prm_Prm_74387/1-676 1 MVAKFSPCSLRRCT---ILLTILALLACV-GTVSGATDANI--A 38
Prm_Prm_79108/1-468 1 M-AKFSPCSRSRCA---ILLAIFALLTCV-GAVSVASESNV--I 37
Prm_Prm_79110/1-588 1 MIAKLGPRSHRRCA---MLLAIFALLTCV-GAVSVASESNV--I 38
Prm_Prm_79107/1-433 1 M-AKFSPCSRSRCA---ILLAIFALLTCV-GAVSVASESNV--A 37
Pin_PITG_14884T0/1-604 1 MPLKLALTPRLSYF---ILLAALALLVCIHSGSKVATASSVIDA 41
Prm_Prm_85872/1-585 1 MVAKFSPCSRSRCA---ILPIILALLACV-GAVSGATDANI--A 38
Pca_102742/1-501 1 MSSKAGLIS---CALWLLLLV---------STLGVDAHKRL--- 29
Pin_PITG_14880T0/1-296 1 MPLKIALTPRFSYF---ILLAALGLLVCIDSGFKVATASNVIDA 41
Prm_Prm_86912/1-294 1 MIAKFSPCSLRRCA---QLLTIFALLACV-GAVSGTTDANI--A 38
Pso_285707/1-589 1 MVSKSNPLN---CS---TLLALLALAASV-GRVLATSHSKITVA 37
Pin_PITG_13847T0/1-512 1 MVAMIVQCTRTKYAHSLILLVATALIIFA----SVVSHSAS--L 38
Prm_Prm_79119/1-666 1 M-AKFSPCSRSRCA---ILLAIFALLTCV-GAVSVASESNV--A 37
Pso_285703/1-570 1 MAFKAIPAQRRYCA---ILLVALALAVSI-SDALPTTNSKTFVA 40
Prm_Prm_74367/1-380 1 MVAKASPCSRSRLA---TLLTIVVLLACV-GGATVATDSNT--G 38
Conservation
* 0 4 7 4 5 5 3 6 0 0 1 6 5 - - - 6 * 6 5 1 0 2 2 1 0 0 1 - 2 0 0 3 6 4 7 2 4 4 5 - - 1
Quality
Consensus
MVAKFSPCSRSRCA--LILLAILALLACV-GAVSVAT+SNVI+A
Prm_Prm_74387/1-676 39 K-PETLDRSEGRQLRGFLRKRSLRTVEEAMKDDDDSEE-RAI-- 78
Prm_Prm_79108/1-468 38 K-PKMLDRAQGHQLRGFQGYRSLRTFVEITKDDDDSEE-RVS-- 77
Prm_Prm_79110/1-588 39 K-PKMLDRAQGHQLRGFQGYRSLRTFVEITKDDDDSEE-RVS-- 78
Prm_Prm_79107/1-433 38 K-PKTLDRAQGHQLRGFQGYRSLRTFLEATKDDDDSEE-RVF-- 77
Pin_PITG_14884T0/1-604 42 R-NDALDREVGGQI-----HRYLRESKWTAEQEDGSPEARAIGV 79
Prm_Prm_85872/1-585 39 K-PETLDRSEERLLRSFHGIRNLRIVEEAMK-DDDSEE-RAI-- 77
Pca_102742/1-501 30 ----------------------LRS----EVSHDNLED-RVL-- 44
Pin_PITG_14880T0/1-296 42 K-NDALDCEVGGQI-----HRYLRESKWTAEQEVGSPEARAIGV 79
Prm_Prm_86912/1-294 39 K-PETLGRSEERLLRSPHGNRNLRTFEEAMK-DDDSEE-RAI-- 77
Pso_285707/1-589 38 STPVSYDVVDPNQVGG-QTPRHLRALG--IEDDDSSVE-RAF-- 75
Pin_PITG_13847T0/1-512 39 K-TIRVDIETSDRTRS---------------------------- 53
Prm_Prm_79119/1-666 38 K-PKPLDRALGRQLRGFQGKRSLRTFVEATKDDDDSEE-RVF-- 77
Pso_285703/1-570 41 S-APSLDQAVT------QTHRHLRA----YEDGVNSEE-KGF-- 70
Prm_Prm_74367/1-380 39 K-PET---SEGRLLRGLQG-----NFVGTTRNDDESEE-RAI-- 70
Conservation
1 - 1 0 0 1 1 0 0 0 0 0 0 0 - - - - - 0 3 1 2 3 0 0 0 - - 0 0 1 0 1 0 0 0 3 - 3 2 0 - -
Quality
Consensus
K-P+TLDRAEGRQLRGFQG+RSLRTFVEATKDDDDSEEARAIGV
Prm_Prm_74387/1-676 79 -------------TDAVKKMAQAAKNKLTSNNQATTNKLFKKLQ 109
Prm_Prm_79108/1-468 78 -------------NEFAKKMIQAAKNKLTSNNQATTNKLFKSLQ 108
Prm_Prm_79110/1-588 79 -------------NEFAKKMIQAAKNKLTSNNQATTNKLFKSLQ 109
Prm_Prm_79107/1-433 78 -------------NEFAKKMAQAAKNKLTSNNQATTNKLFKNLQ 108
Pin_PITG_14884T0/1-604 80 KISAATVIQKIINSQPTKKISTRAMFIITSNNQRATDKIFASIG 123

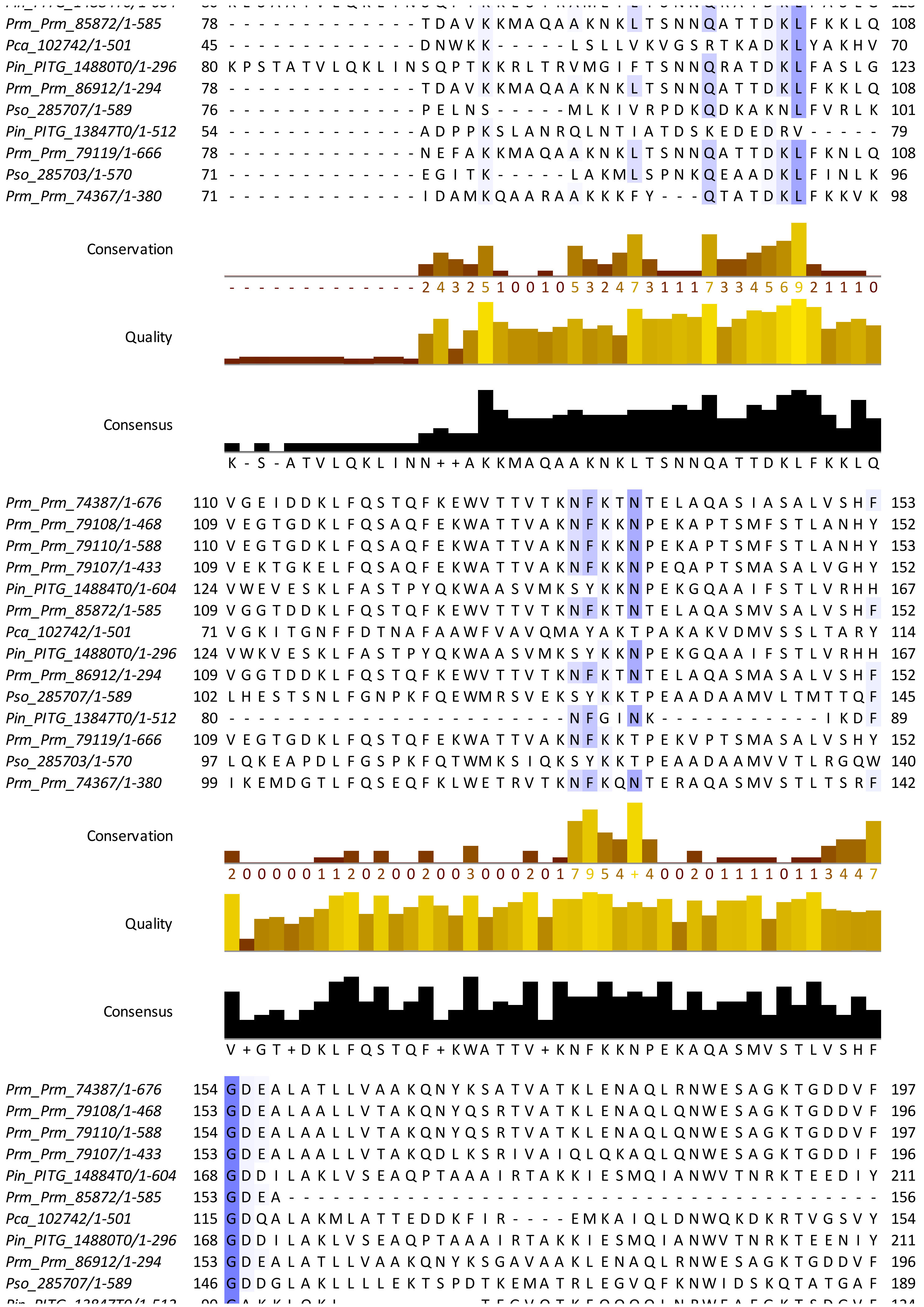
Prm_Prm_85872/1-585
Pca_102742/1-501
Pin_PITG_14880T0/1-296
Prm_Prm_86912/1-294
Pso_285707/1-589
Pin_PITG_13847T0/1-512
Prm_Prm_79119/1-666
Pso_285703/1-570
Prm_Prm_74367/1-380
Conservation
Quality
Consensus
Prm_Prm_74387/1-676
Prm_Prm_79108/1-468
Prm_Prm_79110/1-588
Prm_Prm_79107/1-433
Pin_PITG_14884T0/1-604
Prm_Prm_85872/1-585
Pca_102742/1-501
Pin_PITG_14880T0/1-296
Prm_Prm_86912/1-294
Pso_285707/1-589
Pin_PITG_13847T0/1-512
Prm_Prm_79119/1-666
Pso_285703/1-570
Prm_Prm_74367/1-380
Conservation
Quality
Consensus
Prm_Prm_74387/1-676
Prm_Prm_79108/1-468
Prm_Prm_79110/1-588
Prm_Prm_79107/1-433
Pin_PITG_14884T0/1-604
Prm_Prm_85872/1-585
Pca_102742/1-501
Pin_PITG_14880T0/1-296
Prm_Prm_86912/1-294
Pso_285707/1-589

```
Pin_PITG_13847T0/1-512   90  GAKKLQKL---------IEGVQIKFQQQQLNKWEAEGKISDGVF  124
Prm_Prm_79119/1-666     153  GDEALAALLVTAKQNYQSRTVATQLENGLLQNWKSAGKTGDDVF  196
Pso_285703/1-570        141  GDEKLAKVLQSAMGSSSTKAIAARLEDVQFKKWIGEGKTADDAF  184
Prm_Prm_74367/1-380     143  GDEALAVSLVTAKQNSKSRPIATDLENAQLQNWESAGKTGDDVF  186

Conservation                 *55420002000002000000001110001013000012001 12
Quality
Consensus                    GDEALAKLLVTAKQNYKSRTVATKLENAQLQNWESAGKTGDDVF

Prm_Prm_74387/1-676     198  DLLKLNDKGEKLFESPVAATWISYVTKLNTQNSDEVIFSSLKKL  241
Prm_Prm_79108/1-468     197  DLLKLNDKGEKLFESPVAATWISYMTKLNAQNSDEVIFSSLKKL  240
Prm_Prm_79110/1-588     198  DLLKLNDKGEKLFESPVAATWISYMTKLNAQNSDEVIFSSLKKL  241
Prm_Prm_79107/1-433     197  KLLKLNGKGENLFETPLAPVWMSYVTQLNKENPGEVIFSSLKKL  240
Pin_PITG_14884T0/1-604  212  KLLKLDADKEGLLRNPLLNTWVSFVKKLDNEDPYNLLLLKLTKT  255
Prm_Prm_85872/1-585          --------------------------------------------
Pca_102742/1-501        155  KLLKLDKEQDELLQSPLIATWIAYATKLDNEDPLGAVFSTLKTQ  198
Pin_PITG_14880T0/1-296  212  KLLKLDADEKGLLRNPLLNMWISFVKKLGNEDPYNLLLLKLRKP  255
Prm_Prm_86912/1-294     197  DLLKLNDKGEKLFESPVAATWISYVTKLNTQNSDEVIFSSLKKL  240
Pso_285707/1-589        190  KLLKLDQDAGDILKNPLVSTWMSYV-KLRKESPINEMLLQLKTR  232
Pin_PITG_13847T0/1-512  125  KLLKLDAEGTNILRSPELSTWMAFVSK-QGKDPYDFLLFKLRRR  167
Prm_Prm_79119/1-666     197  ELLKLSDKGEKLFETPLAPIWMSYVTWLNKENPGEVIFSSLKKL  240
Pso_285703/1-570        185  NLLKLDEKANDLLKNPVVKTWVSYVEKLD-VNPYELLLAKLETH  227
Prm_Prm_74367/1-380     187  KLLKLNDKGEKLFESPMAATWLAYLTKLNKKNPEEDMISSLKRV  230

Conservation                 12242202000211220100312211120002100011012110
Quality
Consensus                    KLLKL+DKGEKLFESPLAATWISYVTKLNKENP+EVIFSSLKKL

Prm_Prm_74387/1-676     242  FDDEALAKMIAGAKDSASTAATATKLEQVQLNKWMVEGKTVDDI  285
Prm_Prm_79108/1-468     241  YSDDVLAKMIGGAKNSANVGATAKKLEQVLLSKWMSEGKTIDDI  284
Prm_Prm_79110/1-588     242  YSDDVLAKMIGGAKNSANVGATAKKLEQVLLSKWMSEGKTIDDI  285
Prm_Prm_79107/1-433     241  YSNEVLAKMIVGAKNSASTIVMADKLELLLLNRWLREGKTVDDI  284
Pin_PITG_14884T0/1-604  256  NDEEALAHMLIAAKGDSLTSSVVRNLENALLTAWLRDGKTTGDV  299
Prm_Prm_85872/1-585     157  -----LAKMIAGAKTSASTAATAKKLEQVQLNKWISEGKTADDI  195
Pca_102742/1-501        199  YNGKDFATMLLNVKDTDDSFVVAEKLETLLMKSWQREDKSVVDV  242
Pin_PITG_14880T0/1-296  256  NDEEALAHMLIAAKDNSLTSGVARNLESAMLTIWLRD-------  292
Prm_Prm_86912/1-294     241  FDDEALAKMIAGAKNSASTTATATKLEQVQLNKWMTEGKTVDDI  284
Pso_285707/1-589        233  YGDGALARMLGLAKQDSSTSSIAGQLEEALVKGWLNKRKTDDYV  276
Pin_PITG_13847T0/1-512  168  YDDVDLAKMLVLSKNDANSKAIAEKLETLQLEKWMTDKKSIIDV  211
Prm_Prm_79119/1-666     241  YSDEVLAKMIVGAKNSANVGATANKLEYLQLNKWLSEGKTIDDI  284
Pso_285703/1-570        228  HTAAGLARTLLVARQDYTTRSIAGKLQEAQLERWSRKKRSADYI  271
Prm_Prm_74367/1-380     231  YDDEALAKVITGAQNSASTVVLANTLERALMGKWLDEGKTLDEI  274

Conservation
```

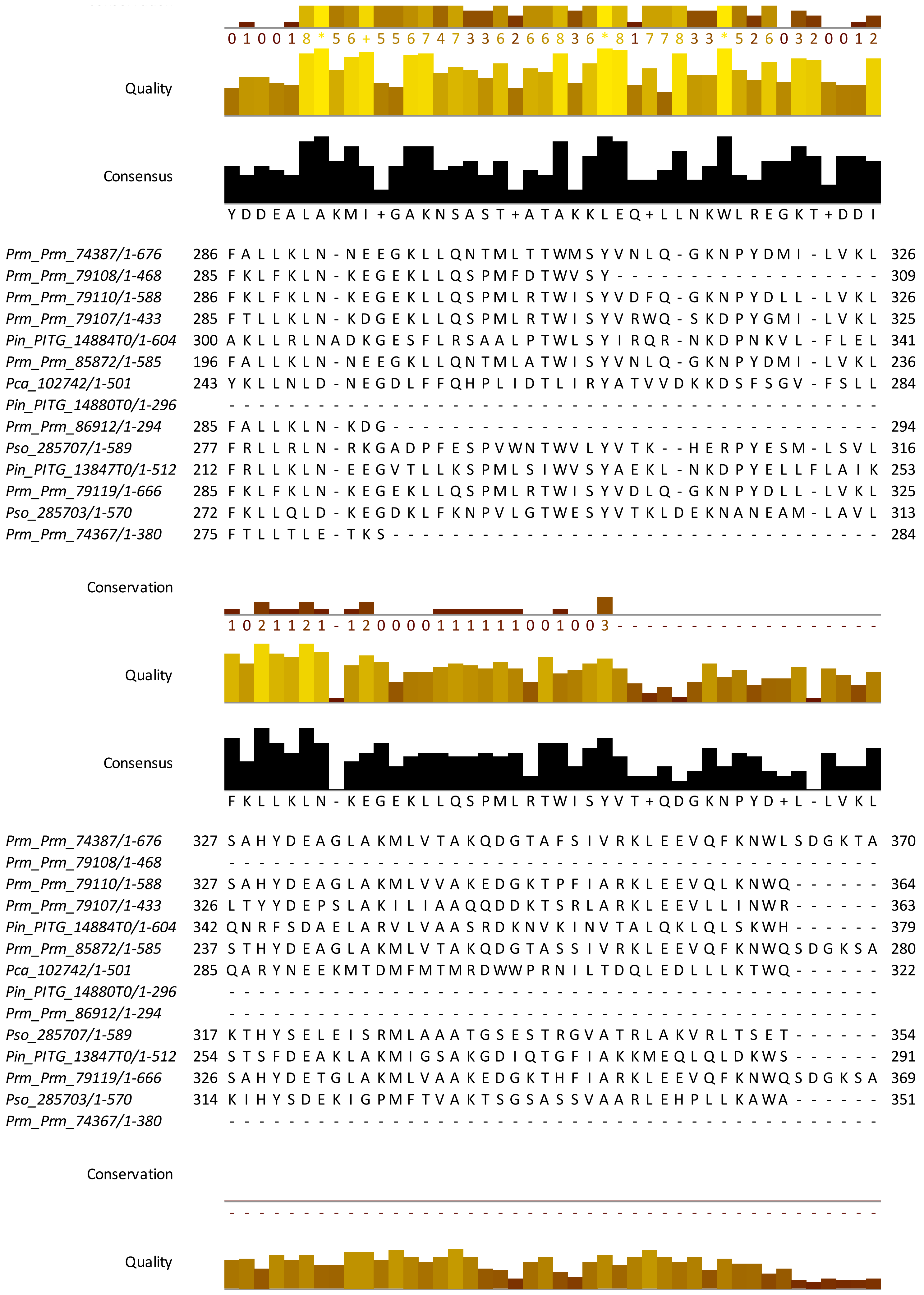
0 1 0 0 1 8 * 5 6 + 5 5 6 7 4 7 3 3 6 2 6 6 8 3 6 * 8 1 7 7 8 3 3 * 5 2 6 0 3 2 0 0 1 2
Quality
Consensus
Y D D E A L A K M I + G A K N S A S T + A T A K K L E Q + L L N K W L R E G K T + D D I
Prm_Prm_74387/1-676 286 F A L L K L N - N E E G K L L Q N T M L T T W M S Y V N L Q - G K N P Y D M I - L V K L 326
Prm_Prm_79108/1-468 285 F K L F K L N - K E G E K L L Q S P M F D T W V S Y - - - - - - - - - - - - - - - - - - 309
Prm_Prm_79110/1-588 286 F K L F K L N - K E G E K L L Q S P M L R T W I S Y V D F Q - G K N P Y D L L - L V K L 326
Prm_Prm_79107/1-433 285 F T L L K L N - K D G E K L L Q S P M L R T W I S Y V R W Q - S K D P Y G M I - L V K L 325
Pin_PITG_14884T0/1-604 300 A K L L R L N A D K G E S F L R S A A L P T W L S Y I R Q R - N K D P N K V L - F L E L 341
Prm_Prm_85872/1-585 196 F A L L K L N - N E E G K L L Q N T M L A T W I S Y V N L Q - G K N P Y D M I - L V K L 236
Pca_102742/1-501 243 Y K L L N L D - N E G D L F F Q H P L I D T L I R Y A T V V D K K D S F S G V - F S L L 284
Pin_PITG_14880T0/1-296 - - - - - - - - - - - - - - - - - - - - - - - - - - - - - - - - - - - - - - - - - - - -
Prm_Prm_86912/1-294 285 F A L L K L N - K D G - - - - - - - - - - - - - - - - - - - - - - - - - - - - - - - - 294
Pso_285707/1-589 277 F R L L R L N - R K G A D P F E S P V W N T W V L Y V T K - - H E R P Y E S M - L S V L 316
Pin_PITG_13847T0/1-512 212 F R L L K L N - E E G V T L L K S P M L S I W V S Y A E K L - N K D P Y E L L F L A I K 253
Prm_Prm_79119/1-666 285 F K L F K L N - K E G E K L L Q S P M L R T W I S Y V D L Q - G K N P Y D L L - L V K L 325
Pso_285703/1-570 272 F K L L Q L D - K E G D K L F K N P V L G T W E S Y V T K L D E K N A N E A M - L A V L 313
Prm_Prm_74367/1-380 275 F T L L T L E - T K S - - - - - - - - - - - - - - - - - - - - - - - - - - - - - - - - 284
Conservation
1 0 2 1 1 2 1 - 1 2 0 0 0 0 1 1 1 1 1 1 0 0 1 0 0 3 - - - - - - - - - - - - - - - - - -
Quality
Consensus
F K L L K L N - K E G E K L L Q S P M L R T W I S Y V T + Q D G K N P Y D + L - L V K L
Prm_Prm_74387/1-676 327 S A H Y D E A G L A K M L V T A K Q D G T A F S I V R K L E E V Q F K N W L S D G K T A 370
Prm_Prm_79108/1-468 - - - - - - - - - - - - - - - - - - - - - - - - - - - - - - - - - - - - - - - - - - - -
Prm_Prm_79110/1-588 327 S A H Y D E A G L A K M L V V A K E D G K T P F I A R K L E E V Q L K N W Q - - - - - - 364
Prm_Prm_79107/1-433 326 L T Y Y D E P S L A K I L I A A Q Q D D K T S R L A R K L E E V L L I N W R - - - - - - 363
Pin_PITG_14884T0/1-604 342 Q N R F S D A E L A R V L V A A S R D K N V K I N V T A L Q K L Q L S K W H - - - - - - 379
Prm_Prm_85872/1-585 237 S T H Y D E A G L A K M L V T A K Q D G T A S S I V R K L E E V Q F K N W Q S D G K S A 280
Pca_102742/1-501 285 Q A R Y N E E K M T D M F M T M R D W W P R N I L T D Q L E D L L L K T W Q - - - - - - 322
Pin_PITG_14880T0/1-296 - - - - - - - - - - - - - - - - - - - - - - - - - - - - - - - - - - - - - - - - - - - -
Prm_Prm_86912/1-294 - - - - - - - - - - - - - - - - - - - - - - - - - - - - - - - - - - - - - - - - - - - -
Pso_285707/1-589 317 K T H Y S E L E I S R M L A A A T G S E S T R G V A T R L A K V R L T S E T - - - - - - 354
Pin_PITG_13847T0/1-512 254 S T S F D E A K L A K M I G S A K G D I Q T G F I A K K M E Q L Q L D K W S - - - - - - 291
Prm_Prm_79119/1-666 326 S A H Y D E T G L A K M L V A A K E D G K T H F I A R K L E E V Q F K N W Q S D G K S A 369
Pso_285703/1-570 314 K I H Y S D E K I G P M F T V A K T S G S A S S V A A R L E H P L L K A W A - - - - - - 351
Prm_Prm_74367/1-380 - - - - - - - - - - - - - - - - - - - - - - - - - - - - - - - - - - - - - - - - - - - -
Conservation
- - - - - - - - - - - - - - - - - - - - - - - - - - - - - - - - - - - - - - - - - - - -
Quality

```
Consensus
                            S + H Y D E A G L A K M L V A A K Q D G K T S + I A R K L E E V Q L K N W Q S D G K S A

Prm_Prm_74387/1-676     371 D D V Y N L L K L N L E R G D I L K S P M L T T W I S Y T A K L D E N P Y Q L L L A L L 414
Prm_Prm_79108/1-468         - - - - - - - - - - - - - - - - - - - - - - - - - - - - - - - - - - - - - - - - - - - -
Prm_Prm_79110/1-588         - - - - - - - - - - - - - - - - - - - - - - - - - - - - - - - - - - - - - - - - - - - -
Prm_Prm_79107/1-433         - - - - - - - - - - - - - - - - - - - - - - - - - - - - - - - - - - - - - - - - - - - -
Pin_PITG_14884T0/1-604      - - - - - - - - - - - - - - - - - - - - - - - - - - - - - - - - - - - - - - - - - - - -
Prm_Prm_85872/1-585     281 D D V Y N L L K L N L E R G D I L K S P M L T T W I S Y T A K L D E N P Y Q L L L V K L 324
Pca_102742/1-501            - - - - - - - - - - - - - - - - - - - - - - - - - - - - - - - - - - - - - - - - - - - -
Pin_PITG_14880T0/1-296      - - - - - - - - - - - - - - - - - - - - - - - - - - - - - - - - - - - - - - - - - - - -
Prm_Prm_86912/1-294         - - - - - - - - - - - - - - - - - - - - - - - - - - - - - - - - - - - - - - - - - - - -
Pso_285707/1-589            - - - - - - - - - - - - - - - - - - - - - - - - - - - - - - - - - - - - - - - - - - - -
Pin_PITG_13847T0/1-512      - - - - - - - - - - - - - - - - - - - - - - - - - - - - - - - - - - - - - - - - - - - -
Prm_Prm_79119/1-666     370 D N V Y N L L K L N L D G D D I L K N P M L T T W I S Y T T K L D E N P Y E L L L A N L 413
Pso_285703/1-570            - - - - - - - - - - - - - - - - - - - - - - - - - - - - - - - - - - - - - - - - - - - -
Prm_Prm_74367/1-380         - - - - - - - - - - - - - - - - - - - - - - - - - - - - - - - - - - - - - - - - - - - -

Conservation
                            - - - - - - - - - - - - - - - - - - - - - - - - - - - - - - - - - - - - - - - - - - - -

Quality

Consensus
                            D D V Y N L L K L N L E R G D I L K S P M L T T W I S Y T A K L D E N P Y Q L L L A - L

Prm_Prm_74387/1-676     415 M K Q Y D D A T L T K M F V A A K R G R A T E S I G T K L E E V Q L K S W L S D G K T A 458
Prm_Prm_79108/1-468         - - - - - - - - - - - - - - - - - - - - - - - - - - - - - - - - - - - - - - - - - - - -
Prm_Prm_79110/1-588     365 - - - - - - - - - - - - - - - - - - - - - - - - - - - - - - - - - - - - - - S G G K T P 370
Prm_Prm_79107/1-433     364 - - - - - - - - - - - - - - - - - - - - - - - - - - - - - - - - - - - - - - S D G K T A 369
Pin_PITG_14884T0/1-604  380 - - - - - - - - - - - - - - - - - - - - - - - - - - - - - - - - - - - - - - G R G D T A 385
Prm_Prm_85872/1-585     325 M K R Y D D A T L T K M F V A A K R G R A T K S I S T K L E E V Q L K S W L S D G K T A 368
Pca_102742/1-501        323 - - - - - - - - - - - - - - - - - - - - - - - - - - - - - - - - - - - - - - R Q E K T M 328
Pin_PITG_14880T0/1-296      - - - - - - - - - - - - - - - - - - - - - - - - - - - - - - - - - - - - - - - - - - - -
Prm_Prm_86912/1-294         - - - - - - - - - - - - - - - - - - - - - - - - - - - - - - - - - - - - - - - - - - - -
Pso_285707/1-589        355 - - - - - - - - - - - - - - - - - - - - - - - - - - - - - - - - - - - - - V N G G E S A 361
Pin_PITG_13847T0/1-512  292 - - - - - - - - - - - - - - - - - - - - - - - - - - - - - - - - - - - - - - A A G K S G 297
Prm_Prm_79119/1-666     414 L K H Y D D A T L T K M F V A A K R S S A T G F T G T K L G E V Q L K N W L S S G K T A 457
Pso_285703/1-570        352 - - - - - - - - - - - - - - - - - - - - - - - - - - - - - - - - - - - - - - N D G K D A 357
Prm_Prm_74367/1-380         - - - - - - - - - - - - - - - - - - - - - - - - - - - - - - - - - - - - - - - - - - - -

Conservation
                            - - - - - - - - - - - - - - - - - - - - - - - - - - - - - - - - - - - - - - - - - - - -

Quality

Consensus
                            M K - Y D D A T L T K M F V A A K R G R A T - S I G T K L E E V Q L K S W L S D G K T A

Prm_Prm_74387/1-676     459 [illegible] 502
```

```
Prm_Prm_74387/1-676      459 DDVFKLLQLNKDGDTIFASSVWSVWSSYLTKLDKENVDELMFVV 502
Prm_Prm_79108/1-468      310 -----------------------------------------FM- 311
Prm_Prm_79110/1-588      371 GGIFKLLKLDKEGDDILASPVLNTWSSYLASLNKGNPDELMFMK 414
Prm_Prm_79107/1-433      370 DDVFKLLLSNKDGGNVLENPMLRVWMAYLTKLDKENADKLMFVV 413
Pin_PITG_14884T0/1-604   386 DDIFRHLGLSKEGEELLESSVFNTWVSYVKLVDYTNTDALVFSV 429
Prm_Prm_85872/1-585      369 DDVFKLLQLNKDGDTIFASSVWSVWSSYLTKLDKENADELMFVV 412
Pca_102742/1-501         329 DDVFKLLKLEQQGDSLFSSKLLSTWVSYVAKVE-TNPYNVVFSK 371
Pin_PITG_14880T0/1-296       --------------------------------------------
Prm_Prm_86912/1-294          --------------------------------------------
Pso_285707/1-589         362 AAAFQRIKFDENGEKLFHN------AEFVAKAE-RYGTKDTLQV 398
Pin_PITG_13847T0/1-512   298 DDVFKSIGLDKEGGKLFESPAWNTWVAYLNMQG-NNADEIIFSV 340
Prm_Prm_79119/1-666      458 DDVFKLLQLNKDGDTILASSIWSVWSSYLTKLDKENADELMFVV 501
Pso_285703/1-570         358 ADVFTLFRLDKEGDKLFENPQFATWAYDISRQGRENPNKVMLSV 401
Prm_Prm_74367/1-380          --------------------------------------------
```

Conservation

-----------------------------------------10-

Quality

Consensus

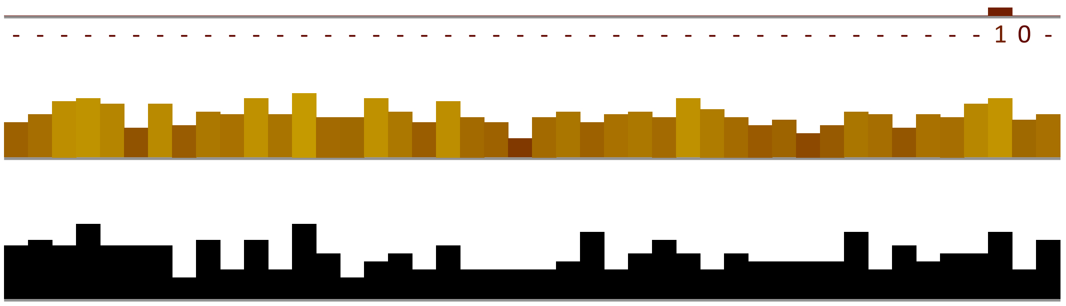

DDVFKLL+L+K+GD+LF+S+VWSTWSSYLTKLDKENADELMF+V

```
Prm_Prm_74387/1-676      503 LKKHYGGDEGVAKMVTIANKEAS--TKDIGRKLKEELWRSQGKD 544
Prm_Prm_79108/1-468      312 -----------------LKSSAS--TPS---KLQEEIWRNQGKT 333
Prm_Prm_79110/1-588      415 LQEQY-GEAGLTSMIAKAKTSAS--TQSIAVKLQEEIWRSQGKT 455
Prm_Prm_79107/1-433      414 LKKHYGGDEGVAKMVTAANK------------------------ 433
Pin_PITG_14884T0/1-604   430 MKKHY-SDEILAKMIAQAKT--SVLTRALASKLAAEMWRSPGKS 470
Prm_Prm_85872/1-585      413 LKKHYGGDEGVAKMVGVANKETS--TKDIGRKLEEELWRSQGKD 454
Pca_102742/1-501         372 LKSTY-GEETLTSMIIQARDMPT-ADYVGTEKVLFKDWVKAKYT 413
Pin_PITG_14880T0/1-296       --------------------------------------------
Prm_Prm_86912/1-294          --------------------------------------------
Pso_285707/1-589         399 LRAHY-TDERLAAILNVASQAPSKRTKVVAISLQLQLWQDQRRS 441
Pin_PITG_13847T0/1-512   341 LRKQL-GDVGLTNAFARANGANSRLTKDVATRLEMDIWRMNGHT 383
Prm_Prm_79119/1-666      502 LKKHY-GDEGVAKIVTVANKGVS--TKDVGRKLEEELWRSQGKD 542
Pso_285703/1-570         402 MKAHY-SDESLEKMMLKATHLSN---SAIVSQLRSELWLSQGKT 441
Prm_Prm_74367/1-380          --------------------------------------------
```

Conservation

-----------------110------------------------

Quality

Consensus

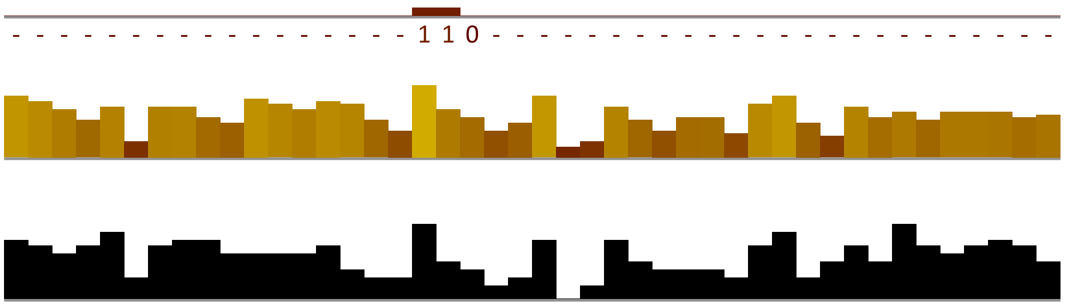

LKKHYGGDEGLAKMV+VANK+AS-LTKDIARKL+EELWRSQGKT

```
Prm_Prm_74387/1-676      545 GDDVFKLLKLDEEGQKFFESPALSTWVSYVS---KLNKQNPG-- 583
Prm_Prm_79108/1-468      334 ADDIFKLLKLRNSGKKMFSTVDLRTWISYVTRLNQFSK-KPDEF 376
Prm_Prm_79110/1-588      456 AEDVFMLLKLRKSGVNIVDAAGLSTWVSYVTKLNKLSK-NPDEF 498
Prm_Prm_79107/1-433          --------------------------------------------
Pin_PITG_14884T0/1-604   471 ADDIFKFFQLDKTGDDLFEAPMFDAWILYVERLNKYEK-HPDKF 513
Prm_Prm_85872/1-585      455 GDDIFKLLKLDEEGQTFFESPALSTWVSYVTKLNKLNKQNPG-- 496
[illegible]
```

```
Pca_102742/1-501        414 VRDAFVHLKLKAEGEEVFESTAFSTWMAYAKELERTNA----DE 453
Pin_PITG_14880T0/1-296  293 -----------------------DIWA----------------- 296
Prm_Prm_86912/1-294         --------------------------------------------
Pso_285707/1-589        442 ADEVFKLLKLEQAGGSVLRSPFLETWVLYAEKLGRVDK-KVGEF 484
Pin_PITG_13847T0/1-512  384 SDDMFKILKLNEKGDKVFESPGFTTWVAYVLKLDGYKK-TPDEF 426
Prm_Prm_79119/1-666     543 GNDIFKLLKLDEEGQKFFESPALRTWISYVTRLNKFNKKSPD-- 584
Pso_285703/1-570        442 VDKAFQLFKLDEAGKNFLEDPGLGSWVSYVTTLSKAKK--SDEF 483
Prm_Prm_74367/1-380     285 ----------------------------------KVNTNTPD-- 292
```

Conservation

Quality

Consensus

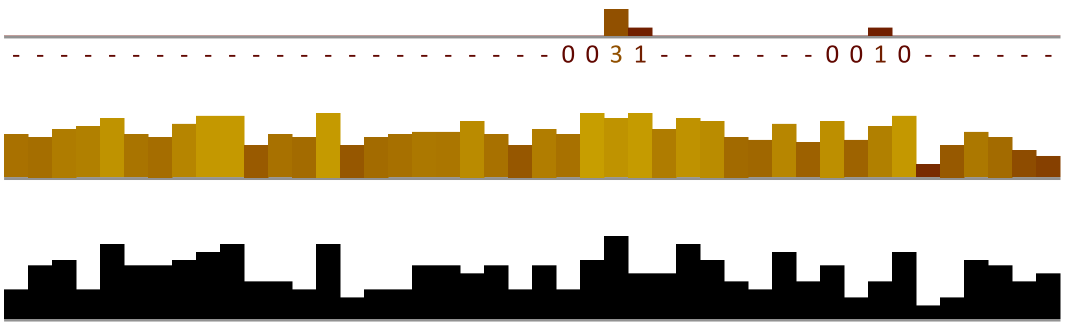

```
Conservation                -----------------------0031-------0010------
Consensus                   ADDIFKLLKLDEEGQKFFESPALSTWVSYVTKLNKLNKQNPDEF
```

```
Prm_Prm_74387/1-676     584 -AISQLEKRFGGDIELARMIGFAKQQAANAVTE----KVITQLQ 622
Prm_Prm_79108/1-468     377 IVILELEKQF-TGAGLARMLADAKYQASLIKAEGAE-TLAGKLQ 418
Prm_Prm_79110/1-588     499 VVISELEKQL-TSMTLARMLAEKINKISLTNAEGAAGSVAGKLE 541
Prm_Prm_79107/1-433         --------------------------------------------
Pin_PITG_14884T0/1-604  514 ALFTELEKRF-DYVDLARMLSHAKIQAEMKGHSV---ERLFSLR 553
Prm_Prm_85872/1-585     497 -AISQLEKRFGGDIEVARMIGFAKLKASNAVTE----KVVTQLQ 535
Pca_102742/1-501        454 AVLTVLKERF-GDAELPRIIAKAKAPIAWDPED----DIALVLE 492
Pin_PITG_14880T0/1-296      --------------------------------------------
Prm_Prm_86912/1-294         --------------------------------------------
Pso_285707/1-589        485 AAIAVLERRF-GGVEFAKMLSAAKSDGALKAS-----SVIDQLQ 522
Pin_PITG_13847T0/1-512  427 APIRQLERHF-GDATLARMLANSKRRASSAKTK----EYIKDLQ 465
Prm_Prm_79119/1-666     585 -AISQLEKRF-GEMELARMIGVAKEKVTTEATE----KVIAQLQ 622
Pso_285703/1-570        484 AAISFLADRF-RDFELAKMLSAAKIKNSGGNN-----RVIEELQ 521
Prm_Prm_74367/1-380     293 -AISQLEKRFGSSIKLAKVIAYAKKRAITEATK----ADATRLQ 331
```

Conservation

Quality

Consensus

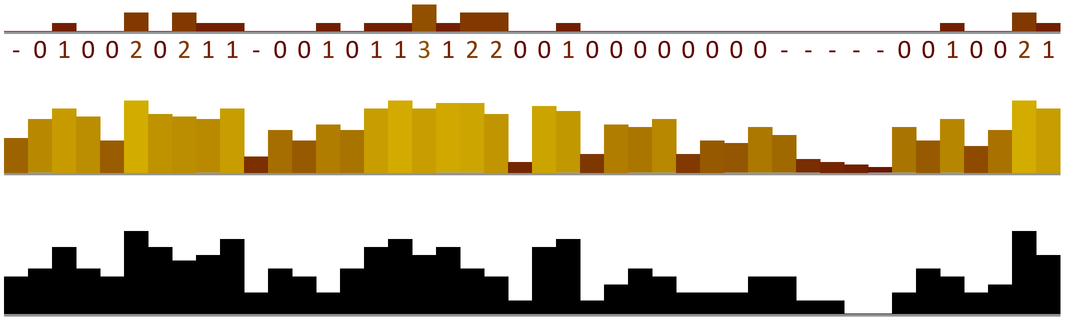

```
Conservation                -010020211-001011312200100000000-----0010021
Consensus                   AAISQLEKRFGGD+ELARMLA+AKIKASLAATEGA--KVITQLQ
```

```
Prm_Prm_74387/1-676     623 NLQFKQWLGDG-RTLNTISDKMVSDTMVSALFDTRNTKVILDYS 665
Prm_Prm_79108/1-468     419 ALQFKKWLKQG-VTPDSITKEFTF-----VLIDSTDVKILLAYS 456
Prm_Prm_79110/1-588     542 ALQFKKWLKQG-ATPNSIAATFTF-----A---RTDLRISVAYN 576
Prm_Prm_79107/1-433         --------------------------------------------
Pin_PITG_14884T0/1-604  554 NQQFDQWMNQKRLDPGRVAALVAE-----QPRDIRNNGVVLGFY 592
Prm_Prm_85872/1-585     536 NLQFKQWLAAG-ITPERVSTKMLL-----TPFDTRNTKVVLDFY 573
Pca_102742/1-501        493 RLQ----------------------------------------- 495
Pin_PITG_14880T0/1-296      --------------------------------------------
Prm_Prm_86912/1-294         --------------------------------------------
Pso_285707/1-589        523 KLQFKKWFKAG-EAPKSVSEKVINRQDP-RGLDLRGSDVAYGYQ 564
Pin_PITG_13847T0/1-512  466 DWQFEKWVVEE-TSPNVLSQLLRK-----KLLGV--DRVRSDFR 501
Prm_Prm_79119/1-666     623 DLQFKQWLAAR-MTPRGLSDKMEL-----TGFDERNAKVVLDYV 660
```

```
Pso_285703/1-570          522 TLQFKKWWEDG-VTPRDISDMVT------KHFDTRGTGVVLGYH 558
Prm_Prm_74367/1-380       332 DLQFKQWLAAG-VTPNNIGTRVMG-----DVFDIRRTQVSIDYG 369
```

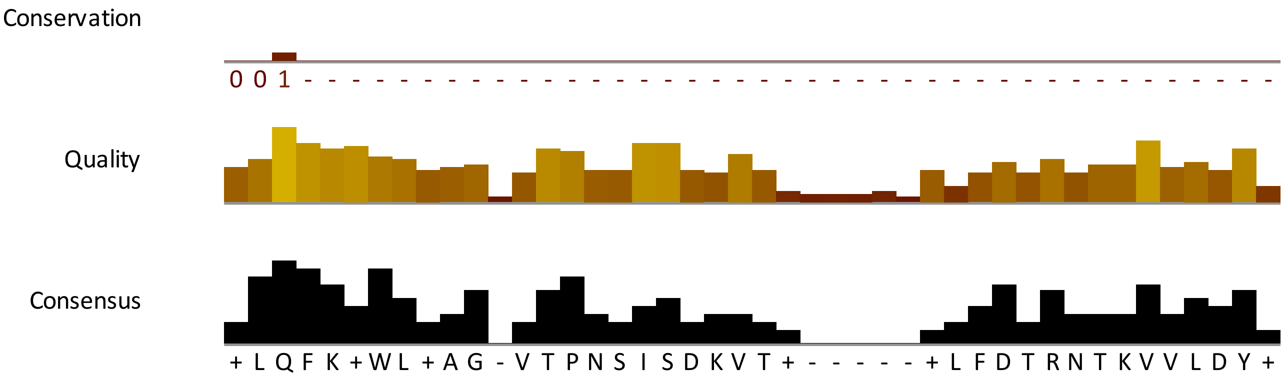


```
Prm_Prm_74387/1-676       666 DFLKA--------------SAARTG 676
Prm_Prm_79108/1-468       457 EYYTL-------------NVGKVNA 468
Prm_Prm_79110/1-588       577 DYYKL-------------NFGKVTA 588
Prm_Prm_79107/1-433           -------------------------
Pin_PITG_14884T0/1-604    593 DFYKA-------------NGGSPLY 604
Prm_Prm_85872/1-585       574 DFYKT-------------IRLSRVI 585
Pca_102742/1-501          496 -------------------KLLSEN 501
Pin_PITG_14880T0/1-296        -------------------------
Prm_Prm_86912/1-294           -------------------------
Pso_285707/1-589          565 DFYKAIVARGGLKDKTRLDSIIERV 589
Pin_PITG_13847T0/1-512    502 NFLKH--------------NPELSG 512
Prm_Prm_79119/1-666       661 DFVRL-------------------H 666
Pso_285703/1-570          559 DFYKA-------------KPVVDPL 570
Prm_Prm_74367/1-380       370 TFYRN--------------KLAAAT 380
```

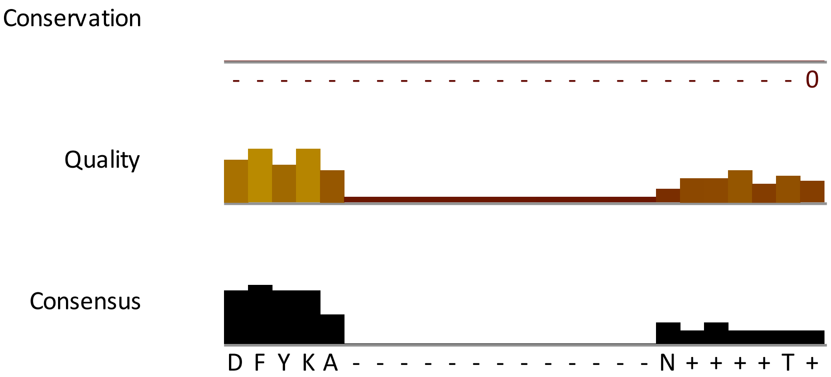

Ortholog 3

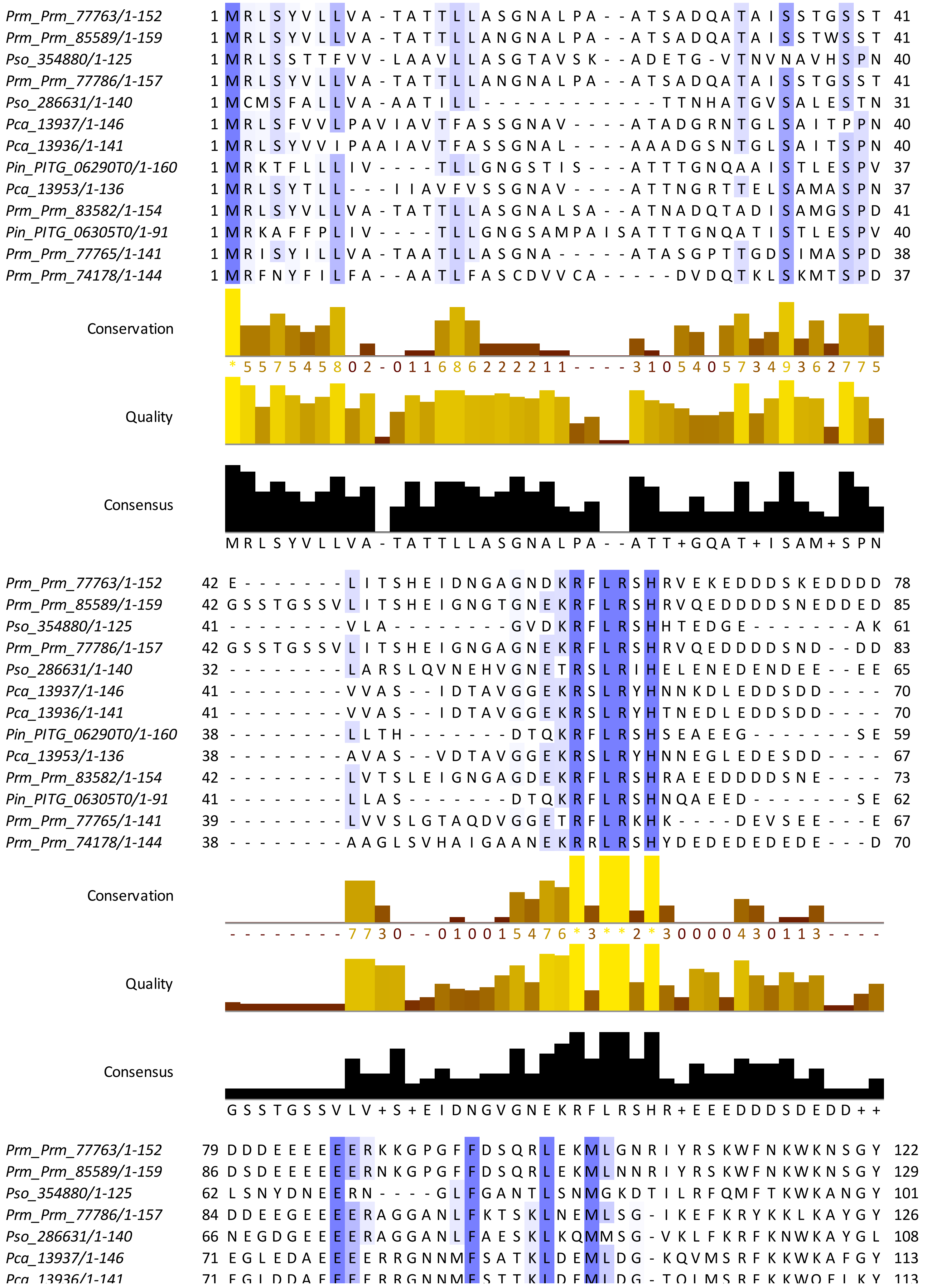
Prm_Prm_77763/1-152 1 M R L S Y V L L V A - T A T T L L A S G N A L P A - - A T S A D Q A T A I S S T G S S T 41
Prm_Prm_85589/1-159 1 M R L S Y V L L V A - T A T T L L A N G N A L P A - - A T S A D Q A T A I S S T W S S T 41
Pso_354880/1-125 1 M R L S S T T F V V - L A A V L L A S G T A V S K - - A D E T G - V T N V N A V H S P N 40
Prm_Prm_77786/1-157 1 M R L S Y V L L V A - T A T T L L A N G N A L P A - - A T S A D Q A T A I S S T G S S T 41
Pso_286631/1-140 1 M C M S F A L L V A - A A T I L L - - - - - - - - - - - - - T T N H A T G V S A L E S T N 31
Pca_13937/1-146 1 M R L S F V V L P A V I A V T F A S S G N A V - - - - A T A D G R N T G L S A I T P P N 40
Pca_13936/1-141 1 M R L S Y V V I P A A I A V T F A S S G N A L - - - - A A A D G S N T G L S A I T S P N 40
Pin_PITG_06290T0/1-160 1 M R K T F L L L I V - - - - T L L G N G S T I S - - - A T T T G N Q A A I S T L E S P V 37
Pca_13953/1-136 1 M R L S Y T L L - - - I I A V F V S S G N A V - - - - A T T N G R T T E L S A M A S P N 37
Prm_Prm_83582/1-154 1 M R L S Y V L L V A - T A T T L L A S G N A L S A - - A T N A D Q T A D I S A M G S P D 41
Pin_PITG_06305T0/1-91 1 M R K A F F P L I V - - - - T L L G N G S A M P A I S A T T T G N Q A T I S T L E S P V 40
Prm_Prm_77765/1-141 1 M R I S Y I L L V A - T A A T L L A S G N A - - - - - A T A S G P T T G D S I M A S P D 38
Prm_Prm_74178/1-144 1 M R F N Y F I L F A - - A A T L F A S C D V V C A - - - - - D V D Q T K L S K M T S P D 37
Conservation
* 5 5 7 5 4 5 8 0 2 - 0 1 1 6 8 6 2 2 2 2 1 1 - - - - 3 1 0 5 4 0 5 7 3 4 9 3 6 2 7 7 5
Quality
Consensus
M R L S Y V L L V A - T A T T L L A S G N A L P A - - A T T + G Q A T + I S A M + S P N
Prm_Prm_77763/1-152 42 E - - - - - - - L I T S H E I D N G A G N D K R F L R S H R V E K E D D D S K E D D D D 78
Prm_Prm_85589/1-159 42 G S S T G S S V L I T S H E I G N G T G N E K R F L R S H R V Q E D D D D S N E D D E D 85
Pso_354880/1-125 41 - - - - - - - - V L A - - - - - - - - G V D K R F L R S H H T E D G E - - - - - - - A K 61
Prm_Prm_77786/1-157 42 G S S T G S S V L I T S H E I G N G A G N E K R F L R S H R V Q E D D D D S N D - - D D 83
Pso_286631/1-140 32 - - - - - - - - L A R S L Q V N E H V G N E T R S L R I H E L E N E D E N D E E - - E E 65
Pca_13937/1-146 41 - - - - - - - - V V A S - - I D T A V G G E K R S L R Y H N N K D L E D D S D D - - - - 70
Pca_13936/1-141 41 - - - - - - - - V V A S - - I D T A V G G E K R S L R Y H T N E D L E D D S D D - - - - 70
Pin_PITG_06290T0/1-160 38 - - - - - - - - L L T H - - - - - - - D T Q K R F L R S H S E A E E G - - - - - - - S E 59
Pca_13953/1-136 38 - - - - - - - - A V A S - - V D T A V G G E K R S L R Y H N N E G L E D E S D D - - - - 67
Prm_Prm_83582/1-154 42 - - - - - - - - L V T S L E I G N G A G D E K R F L R S H R A E E D D D D S N E - - - - 73
Pin_PITG_06305T0/1-91 41 - - - - - - - - L L A S - - - - - - - D T Q K R F L R S H N Q A E E D - - - - - - - S E 62
Prm_Prm_77765/1-141 39 - - - - - - - - L V V S L G T A Q D V G G E T R F L R K H K - - - - D E V S E E - - - E 67
Prm_Prm_74178/1-144 38 - - - - - - - - A A G L S V H A I G A A N E K R R L R S H Y D E D E D E D E D E - - - D 70
Conservation
- - - - - - - - 7 7 3 0 - - 0 1 0 0 1 5 4 7 6 * 3 * * 2 * 3 0 0 0 0 4 3 0 1 1 3 - - - -
Quality
Consensus
G S S T G S S V L V + S + E I D N G V G N E K R F L R S H R + E E E D D D S D E D D + +
Prm_Prm_77763/1-152 79 D D D E E E E E E R K K G P G F F D S Q R L E K M L G N R I Y R S K W F N K W K N S G Y 122
Prm_Prm_85589/1-159 86 D S D E E E E E E R N K G P G F F D S Q R L E K M L N N R I Y R S K W F N K W K N S G Y 129
Pso_354880/1-125 62 L S N Y D N E E R N - - - - G L F G A N T L S N M G K D T I L R F Q M F T K W K A N G Y 101
Prm_Prm_77786/1-157 84 D D E E G E E E E R A G G A N L F K T S K L N E M L S G - I K E F K R Y K K L K A Y G Y 126
Pso_286631/1-140 66 N E G D G E E E E R A G G A N L F A E S K L K Q M M S G - V K L F K R F K N W K A Y G L 108
Pca_13937/1-146 71 E G L E D A E E E E R R G N N M F S A T K L D E M L D G - K Q V M S R F K K W K A F G Y 113
Pca_13936/1-141 71 E G L D D A E E E E R R G N N M F S T T K L D E M L D G - T Q I M S R F K K W Q E L K Y 113

```
Pin_PITG_06290T0/1-160   60 E D E F G N A E E R K V G A N L F A D S K L A Q M L N S - R K T F P R F A R W K R E G F 102
Pca_13953/1-136          68 E A L - - L E E E E R K Y T N M F S T T K L D E M L N G - T K M M S R F R K W K A R G Y 108
Prm_Prm_83582/1-154      74 N E N E N E E E E R A G G A N L F T T S K L N E M L S G - V K T F D R F A R W K R D G W 116
Pin_PITG_06305T0/1-91    63 E D E F G N G E E R K V G D R L F A D W K L E Q M L N S - - - - - - - - - - - - - - - - 90
Prm_Prm_77765/1-141      68 D D D D E E E E E R K K G P G F F D S Q R L E K M L G N R I Y R S K W F N R W K D G G Y 111
Prm_Prm_74178/1-144      71 E D E D E D E E E R I K G V D L F K E W K L D Q M L S G - I K E F K R Y K N L K A Y G Y 113
```

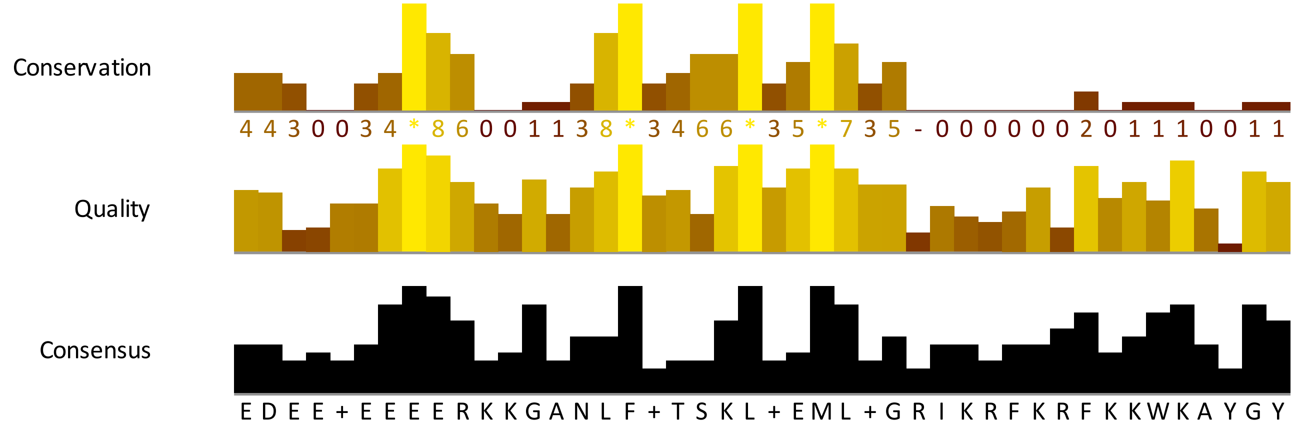


```
Prm_Prm_77763/1-152     123 T P G N V E S E M G N K - L Y Q K Y R - I W Y Y Y G P G A R R - - - - - - - - - - - - - 151
Prm_Prm_85589/1-159     130 T P G N V E S E M G N K - L Y Q K Y R - T W Y Y Y G P G A R R - - - - - - - - - - - - - 158
Pso_354880/1-125        102 L P K K I K D D I P R S - - - - - - - - L Y K A Y K I H Y R M - - - - - - - - - - - - - 124
Prm_Prm_77786/1-157     127 T P I K F Q T K L E K M - Y G N K Y K D L W E R Y S A N F Y S - - - - - - - - - - - - - 156
Pso_286631/1-140        109 S P G D V S I R L E N A G K W D D Y K E L Y F T Y Q K Y Y S T - - - - - - - - - - - - - 139
Pca_13937/1-146         114 N T Y N L P D A I Q A - - - - R K Y D E L R K M Y R K F L Y Y - - - - - - - - - - - - - 140
Pca_13936/1-141         114 N M Y N L P D T I L A - - - - S K Y D E L R K M Y R R F L Y Y - - - - - - - - - - - - - 140
Pin_PITG_06290T0/1-160  103 I P S T L R T K L E K L G L W T K Y S G L H T M Y S N N Y Y V H H R E G Q T V K V R V D 146
Pca_13953/1-136         109 N T Y N L P A V T Q K - - - - D K Y T W I R Q K Y R D F L Y H - - - - - - - - - - - - - 135
Prm_Prm_83582/1-154     117 I P T A I F N K F G T K - - - S K Y K E L A S M Y N R N Y D T I R - - - - - - - - - - - 146
Pin_PITG_06305T0/1-91       - - - - - - - - - - - - - - - - - - - - - - - - - - - - - - - - - - - - - - - - - - - -
Prm_Prm_77765/1-141     112 T P I N V Q N N M G N E - L Y Q K Y K - H W Y Y H G P G A L R - - - - - - - - - - - - - 140
Prm_Prm_74178/1-144     114 T P S S L Q T K L T R L - Y G K K Y K D L W E R Y S A N Y Y T - - - - - - - - - - - - - 143
```

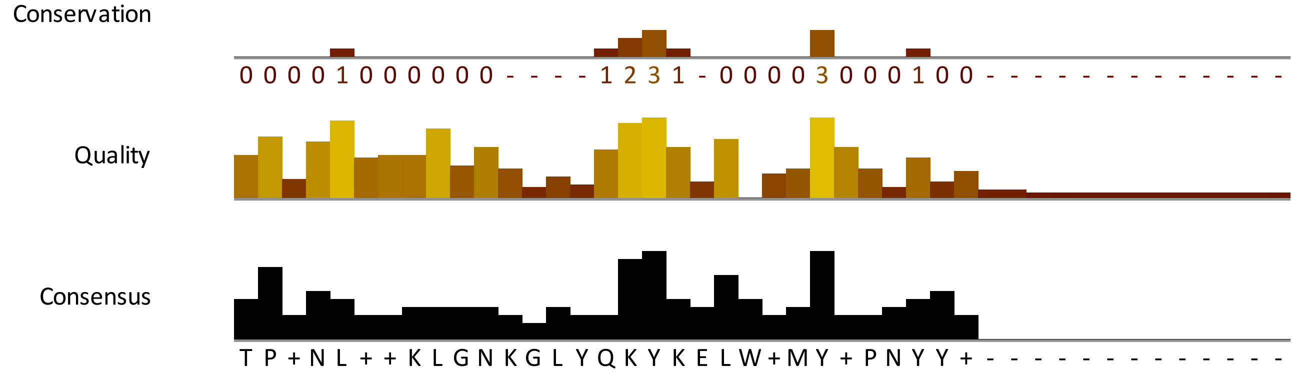


```
Prm_Prm_77763/1-152     152 - - - - - - - - - - - - - N - - - - - 152
Prm_Prm_85589/1-159     159 - - - - - - - - - - - - - N - - - - - 159
Pso_354880/1-125        125 - - - - - - - - - - - - - N - - - - - 125
Prm_Prm_77786/1-157     157 - - - - - - - - - - - - - I - - - - - 157
Pso_286631/1-140        140 - - - - - - - - - - - - - I - - - - - 140
Pca_13937/1-146         141 - - - - - - - - - - - - - N Y R K K K 146
Pca_13936/1-141         141 - - - - - - - - - - - - - N - - - - - 141
Pin_PITG_06290T0/1-160  147 L A I I H S R R N T N G G S - - - - - 160
Pca_13953/1-136         136 - - - - - - - - - - - - - N - - - - - 136
Prm_Prm_83582/1-154     147 - - - - - - V K P V N A G R - - - - - 154
Pin_PITG_06305T0/1-91    91 - - - - - - - - - - - - - H - - - - - 91
Prm_Prm_77765/1-141     141 - - - - - - - - - - - - - N - - - - - 141
Prm_Prm_74178/1-144     144 - - - - - - - - - - - - - I - - - - - 144
```

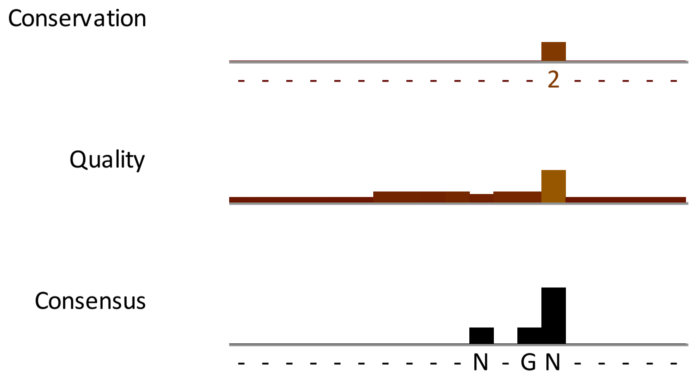
Conservation
- - - - - - - - - - - - - 2 - - - - -
Quality
Consensus
- - - - - - - - - - N - G N - - - - -

Ortholog 4

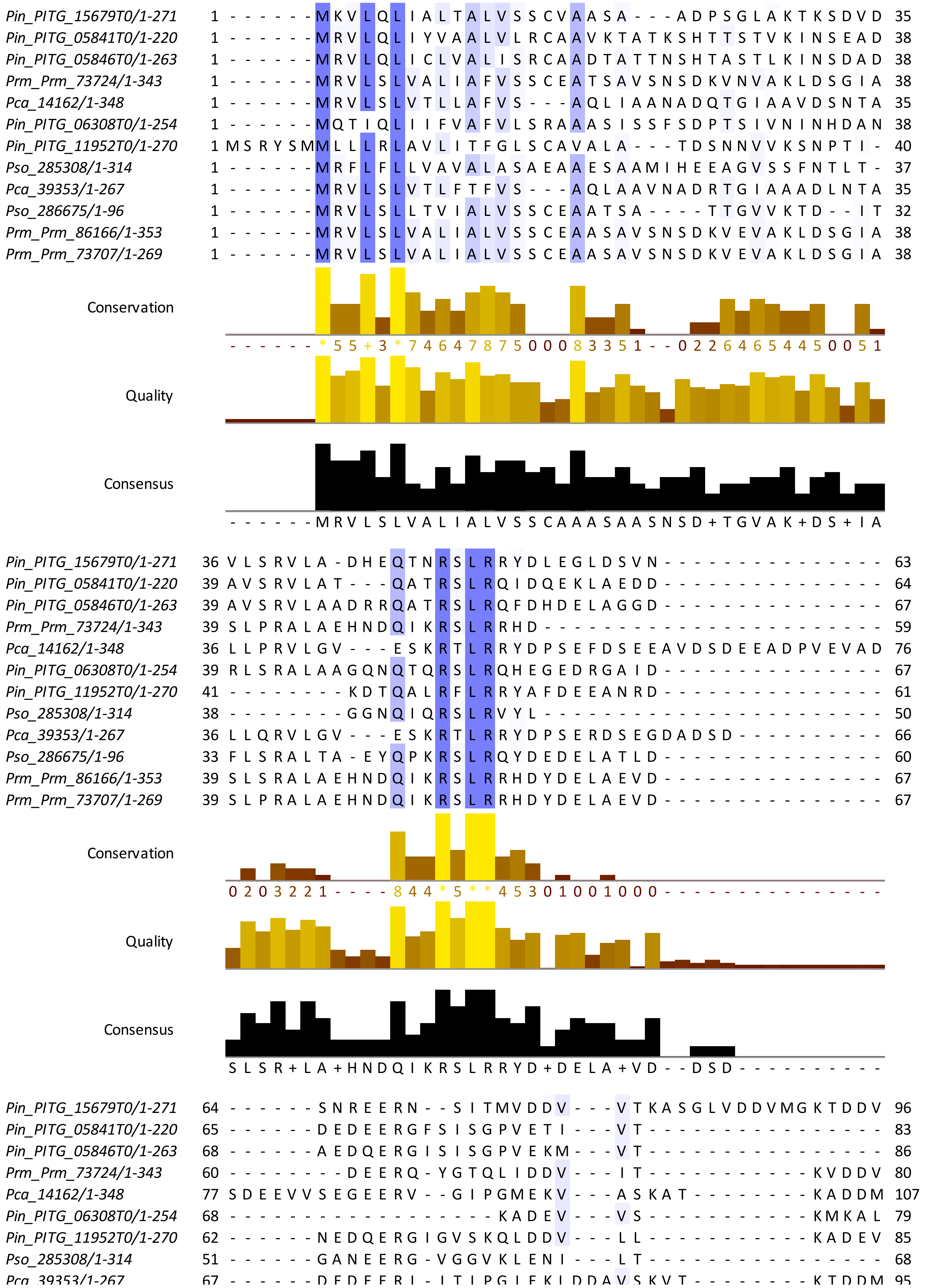
Pin_PITG_15679T0/1-271 1 ------MKVLQLIALTALVSSCVAASA---ADPSGLAKTKSDVD 35
Pin_PITG_05841T0/1-220 1 ------MRVLQLIYVAALVLRCAAVKTATKSHTTSTVKINSEAD 38
Pin_PITG_05846T0/1-263 1 ------MRVLQLICLVALISRCAADTATTNSHTASTLKINSDAD 38
Prm_Prm_73724/1-343 1 ------MRVLSLVALIAFVSSCEATSAVSNSDKVNVAKLDSGIA 38
Pca_14162/1-348 1 ------MRVLSLVTLLAFVS---AQLIAANADQTGIAAVDSNTA 35
Pin_PITG_06308T0/1-254 1 ------MQTIQLIIFVAFVLSRAAASISSFSDPTSIVNINHDAN 38
Pin_PITG_11952T0/1-270 1 MSRYSMMLLLRLAVLITFGLSCAVALA---TDSNNVVKSNPTI- 40
Pso_285308/1-314 1 ------MRFLFLLVAVALASAEAAESAAMIHEEAGVSSFNTLT- 37
Pca_39353/1-267 1 ------MRVLSLVTLFTFVS---AQLAAVNADRTGIAAADLNTA 35
Pso_286675/1-96 1 ------MRVLSLLTVIALVSSCEAATSA----TTGVVKTD--IT 32
Prm_Prm_86166/1-353 1 ------MRVLSLVALIALVSSCEAASAVSNSDKVEVAKLDSGIA 38
Prm_Prm_73707/1-269 1 ------MRVLSLVALIALVSSCEAASAVSNSDKVEVAKLDSGIA 38
Conservation
------*55+3*7464787500083351--02264654450051
Quality
Consensus
------MRVLSLVALIALVSSCAAASAASNSD+TGVAK+DS+IA
Pin_PITG_15679T0/1-271 36 VLSRVLA-DHEQTNRSLRRYDLEGLDSVN--------------- 63
Pin_PITG_05841T0/1-220 39 AVSRVLAT---QATRSLRQIDQEKLAEDD--------------- 64
Pin_PITG_05846T0/1-263 39 AVSRVLAADRRQATRSLRQFDHDELAGGD--------------- 67
Prm_Prm_73724/1-343 39 SLPRALAEHNDQIKRSLRRHD----------------------- 59
Pca_14162/1-348 36 LLPRVLGV---ESKRTLRRYDPSEFDSEEAVDSDEEADPVEVAD 76
Pin_PITG_06308T0/1-254 39 RLSRALAAGQNQTQRSLRQHEGEDRGAID--------------- 67
Pin_PITG_11952T0/1-270 41 --------KDTQALRFLRRYAFDEEANRD--------------- 61
Pso_285308/1-314 38 --------GGNQIQRSLRVYL----------------------- 50
Pca_39353/1-267 36 LLQRVLGV---ESKRTLRRYDPSERDSEGDADSD---------- 66
Pso_286675/1-96 33 FLSRALTA-EYQPKRSLRQYDEDELATLD--------------- 60
Prm_Prm_86166/1-353 39 SLSRALAEHNDQIKRSLRRHDYDELAEVD--------------- 67
Prm_Prm_73707/1-269 39 SLPRALAEHNDQIKRSLRRHDYDELAEVD--------------- 67
Conservation
0203221----844*5**45301001000---------------
Quality
Consensus
SLSR+LA+HNDQIKRSLRRYD+DELA+VD--DSD----------
Pin_PITG_15679T0/1-271 64 ------SNREERN--SITMVDDV---VTKASGLVDDVMGKTDDV 96
Pin_PITG_05841T0/1-220 65 ------DEDEERGFSISGPVETI---VT---------------- 83
Pin_PITG_05846T0/1-263 68 ------AEDQERGISISGPVEKM---VT---------------- 86
Prm_Prm_73724/1-343 60 --------DEERQ-YGTQLIDDV---IT----------KVDDV 80
Pca_14162/1-348 77 SDEEVVSEGEERV--GIPGMEKV---ASKAT--------KADDM 107
Pin_PITG_06308T0/1-254 68 -----------------KADEV---VS----------KMKAL 79
Pin_PITG_11952T0/1-270 62 ------NEDQERGIGVSKQLDDV---LL----------KADEV 85
Pso_285308/1-314 51 ------GANEERG-VGGVKLENI---LT---------------- 68
Pca_39353/1-267 67 ------DEDEERL-ITIPGIEKIDDAVSKVT--------KTDDM 95

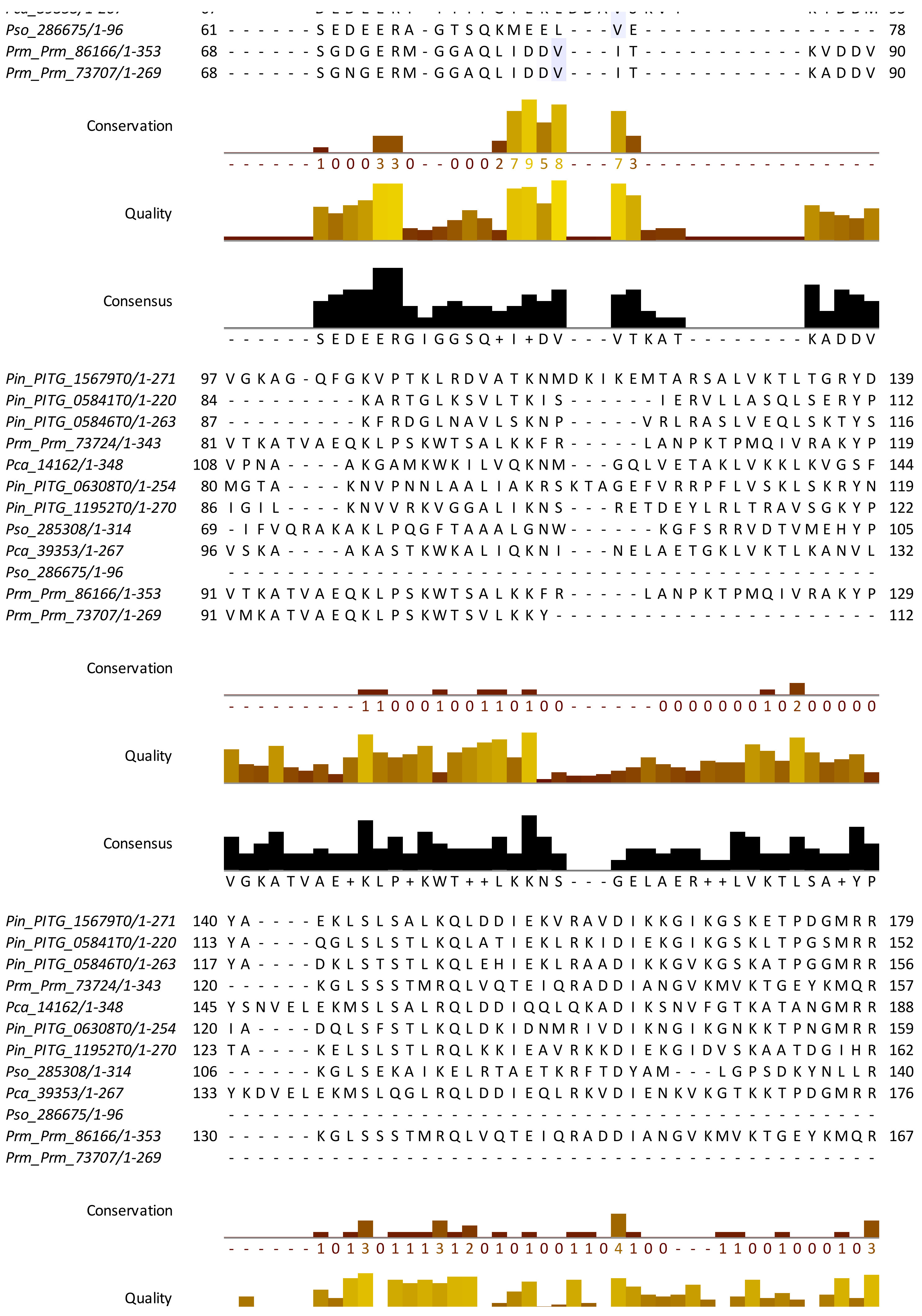
Pso_286675/1-96 61 ------SEDEERA-GTSQKMEEL---VE------------------ 78
Prm_Prm_86166/1-353 68 ------SGDGERM-GGAQLIDDV---IT------------KVDDV 90
Prm_Prm_73707/1-269 68 ------SGNGERM-GGAQLIDDV---IT------------KADDV 90
Conservation
------1000330--00027958---73------------------
Quality
Consensus
------SEDEERGIGGSQ+I+DV---VTKAT--------KADDV
Pin_PITG_15679T0/1-271 97 VGKAG-QFGKVPTKLRDVATKNMDKIKEMTARSALVKTLTGRYD 139
Pin_PITG_05841T0/1-220 84 ---------KARTGLKSVLTKIS------IERVLLASQLSERYP 112
Pin_PITG_05846T0/1-263 87 ---------KFRDGLNAVLSKNP-----VRLRASLVEQLSKTYS 116
Prm_Prm_73724/1-343 81 VTKATVAEQKLPSKWTSALKKFR-----LANPKTPMQIVRAKYP 119
Pca_14162/1-348 108 VPNA----AKGAMKWKILVQKNM---GQLVETAKLVKKLKVGSF 144
Pin_PITG_06308T0/1-254 80 MGTA----KNVPNNLAALIAKRSKTAGEFVRRPFLVSKLSKRYN 119
Pin_PITG_11952T0/1-270 86 IGIL----KNVVRKVGGALIKNS---RETDEYLRLTRAVSGKYP 122
Pso_285308/1-314 69 -IFVQRAKAKLPQGFTAAALGNW------KGFSRRVDTVMEHYP 105
Pca_39353/1-267 96 VSKA----AKASTKWKALIQKNI---NELAETGKLVKTLKANVL 132
Pso_286675/1-96 --------------------------------------------
Prm_Prm_86166/1-353 91 VTKATVAEQKLPSKWTSALKKFR-----LANPKTPMQIVRAKYP 129
Prm_Prm_73707/1-269 91 VMKATVAEQKLPSKWTSVLKKY---------------------- 112
Conservation
---------1100010011010 0------0000000102 00000
Quality
Consensus
VGKATVAE+KLP+KWT++LKKNS---GELAER++LVKTLSA+YP
Pin_PITG_15679T0/1-271 140 YA----EKLSLSALKQLDDIEKVRAVDIKKGIKGSKETPDGMRR 179
Pin_PITG_05841T0/1-220 113 YA----QGLSLSTLKQLATIEKLRKIDIEKGIKGSKLTPGSMRR 152
Pin_PITG_05846T0/1-263 117 YA----DKLSTSTLKQLEHIEKLRAADIKKGVKGSKATPGGMRR 156
Prm_Prm_73724/1-343 120 ------KGLSSSTMRQLVQTEIQRADDIANGVKMVKTGEYKMQR 157
Pca_14162/1-348 145 YSNVELEKMSLSALRQLDDIQQLQKADIKSNVFGTKATANGMRR 188
Pin_PITG_06308T0/1-254 120 IA----DQLSFSTLKQLDKIDNMRIVDIKNGIKGNKKTPNGMRR 159
Pin_PITG_11952T0/1-270 123 TA----KELSLSTLRQLKKIEAVRKKDIEKGIDVSKAATDGIHR 162
Pso_285308/1-314 106 ------KGLSEKAIKELRTAETKRFTDYAM---LGPSDKYNLLR 140
Pca_39353/1-267 133 YKDVELEKMSLQGLRQLDDIEQLRKVDIENKVKGTKKTPDGMRR 176
Pso_286675/1-96 --------------------------------------------
Prm_Prm_86166/1-353 130 ------KGLSSSTMRQLVQTEIQRADDIANGVKMVKTGEYKMQR 167
Prm_Prm_73707/1-269 --------------------------------------------
Conservation
------101301113120101001104100---1100100010 3
Quality

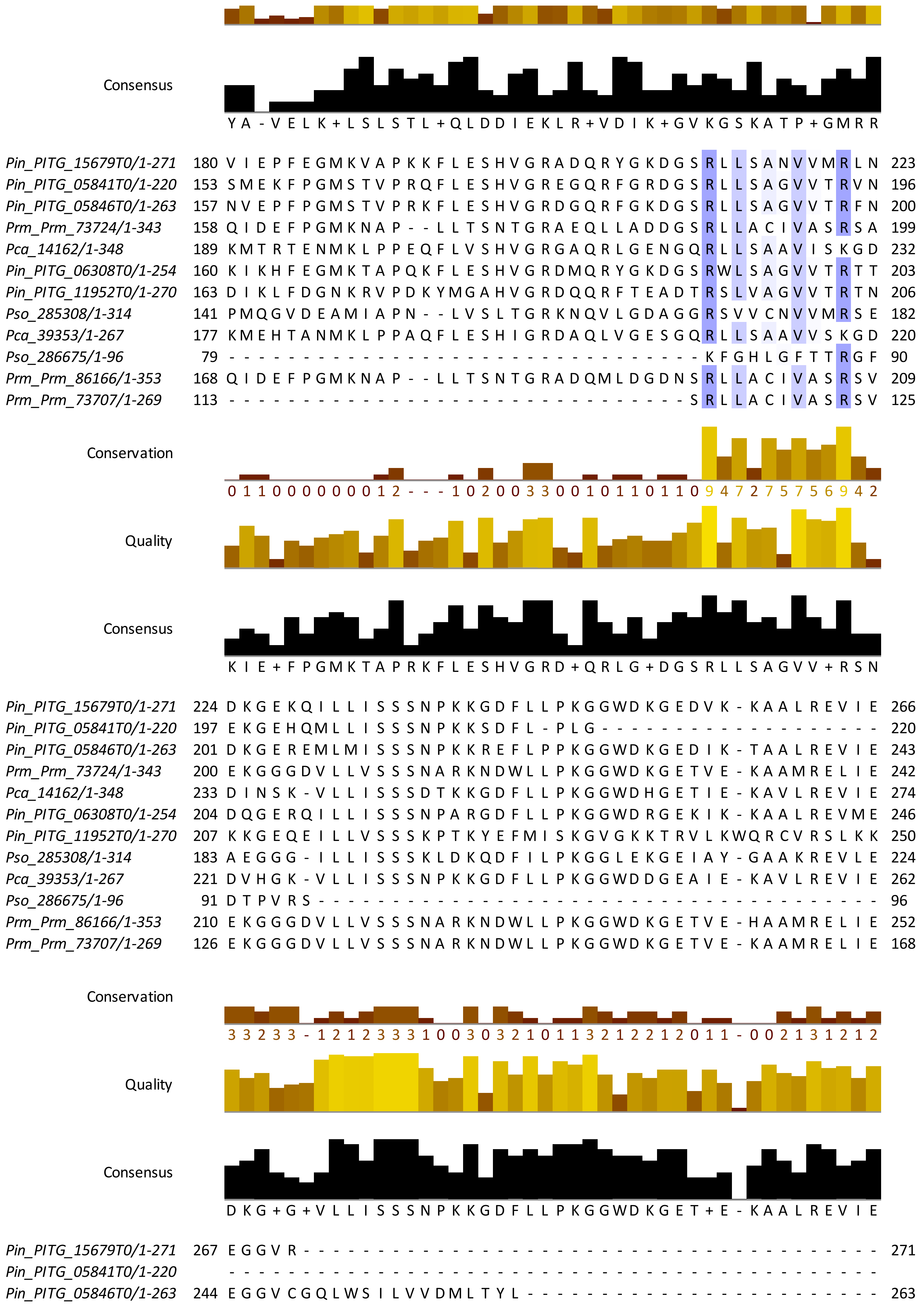
Consensus
Y A - V E L K + L S L S T L + Q L D D I E K L R + V D I K + G V K G S K A T P + G M R R
Pin_PITG_15679T0/1-271 180 V I E P F E G M K V A P K K F L E S H V G R A D Q R Y G K D G S R L L S A N V V M R L N 223
Pin_PITG_05841T0/1-220 153 S M E K F P G M S T V P R Q F L E S H V G R E G Q R F G R D G S R L L S A G V V T R V N 196
Pin_PITG_05846T0/1-263 157 N V E P F P G M S T V P R K F L E S H V G R D G Q R F G K D G S R L L S A G V V T R F N 200
Prm_Prm_73724/1-343 158 Q I D E F P G M K N A P - - L L T S N T G R A E Q L L A D D G S R L L A C I V A S R S A 199
Pca_14162/1-348 189 K M T R T E N M K L P P E Q F L V S H V G R G A Q R L G E N G Q R L L S A A V I S K G D 232
Pin_PITG_06308T0/1-254 160 K I K H F E G M K T A P Q K F L E S H V G R D M Q R Y G K D G S R W L S A G V V T R T T 203
Pin_PITG_11952T0/1-270 163 D I K L F D G N K R V P D K Y M G A H V G R D Q Q R F T E A D T R S L V A G V V T R T N 206
Pso_285308/1-314 141 P M Q G V D E A M I A P N - - L V S L T G R K N Q V L G D A G G R S V V C N V V M R S E 182
Pca_39353/1-267 177 K M E H T A N M K L P P A Q F L E S H I G R D A Q L V G E S G Q R L L S A A V V S K G D 220
Pso_286675/1-96 79 - - - - - - - - - - - - - - - - - - - - - - - - - - - - - - - - K F G H L G F T T R G F 90
Prm_Prm_86166/1-353 168 Q I D E F P G M K N A P - - L L T S N T G R A D Q M L D G D N S R L L A C I V A S R S V 209
Prm_Prm_73707/1-269 113 - - - - - - - - - - - - - - - - - - - - - - - - - - - - - - - S R L L A C I V A S R S V 125
Conservation
0 1 1 0 0 0 0 0 0 0 1 2 - - - 1 0 2 0 0 3 3 0 0 1 0 1 1 0 1 1 0 9 4 7 2 7 5 7 5 6 9 4 2
Quality
Consensus
K I E + F P G M K T A P R K F L E S H V G R D + Q R L G + D G S R L L S A G V V + R S N
Pin_PITG_15679T0/1-271 224 D K G E K Q I L L I S S S N P K K G D F L L P K G G W D K G E D V K - K A A L R E V I E 266
Pin_PITG_05841T0/1-220 197 E K G E H Q M L L I S S S N P K K S D F L - P L G - - - - - - - - - - - - - - - - - - - 220
Pin_PITG_05846T0/1-263 201 D K G E R E M L M I S S S N P K K R E F L P P K G G W D K G E D I K - T A A L R E V I E 243
Prm_Prm_73724/1-343 200 E K G G G D V L L V S S S N A R K N D W L L P K G G W D K G E T V E - K A A M R E L I E 242
Pca_14162/1-348 233 D I N S K - V L L I S S S D T K K G D F L L P K G G W D H G E T I E - K A V L R E V I E 274
Pin_PITG_06308T0/1-254 204 D Q G E R Q I L L I S S S N P A R G D F L L P K G G W D R G E K I K - K A A L R E V M E 246
Pin_PITG_11952T0/1-270 207 K K G E Q E I L L V S S S K P T K Y E F M I S K G V G K K T R V L K W Q R C V R S L K K 250
Pso_285308/1-314 183 A E G G G - I L L I S S S K L D K Q D F I L P K G G L E K G E I A Y - G A A K R E V L E 224
Pca_39353/1-267 221 D V H G K - V L L I S S S N P K K G D F L L P K G G W D D G E A I E - K A V L R E V I E 262
Pso_286675/1-96 91 D T P V R S - - - - - - - - - - - - - - - - - - - - - - - - - - - - - - - - - - - - - - 96
Prm_Prm_86166/1-353 210 E K G G G D V L L V S S S N A R K N D W L L P K G G W D K G E T V E - H A A M R E L I E 252
Prm_Prm_73707/1-269 126 E K G G G D V L L V S S S N A R K N D W L L P K G G W D K G E T V E - K A A M R E L I E 168
Conservation
3 3 2 3 3 - 1 2 1 2 3 3 3 1 0 0 3 0 3 2 1 0 1 1 3 2 1 2 2 1 2 0 1 1 - 0 0 2 1 3 1 2 1 2
Quality
Consensus
D K G + G + V L L I S S S N P K K G D F L L P K G G W D K G E T + E - K A A L R E V I E
Pin_PITG_15679T0/1-271 267 E G G V R - - - - - - - - - - - - - - - - - - - - - - - - - - - - - - - - - - - - - - - 271
Pin_PITG_05841T0/1-220 - - - - - - - - - - - - - - - - - - - - - - - - - - - - - - - - - - - - - - - - - - - -
Pin_PITG_05846T0/1-263 244 E G G V C G Q L W S I L V V D M L T Y L - - - - - - - - - - - - - - - - - - - - - - - - 263

```
Prm_Prm_73724/1-343     243 EGGVGGT-------------IMHSLGETAFK---NALEPSKGYV 270
Pca_14162/1-348         275 EGGVNGQ-------------LLHKLGEYPFK---------KGAT 296
Pin_PITG_06308T0/1-254  247 EGGVCRAL------------------------------------ 254
Pin_PITG_11952T0/1-270  251 EGYVD--------------------------------------- 255
Pso_285308/1-314        225 EGGVRYRSDIFIFYRWSVLTLVMALQVKVKKLKELGVTLVGDKT 268
Pca_39353/1-267         263 EGGVK--------------------------------------- 267
Pso_286675/1-96             --------------------------------------------
Prm_Prm_86166/1-353     253 EGGVGGT-------------IMHSLGETAFK---NALEPSKGYV 280
Prm_Prm_73707/1-269     169 EGGVGGT-------------IMHSLGETAFK---NALEPSKGYV 196
```

Conservation

Quality

Consensus

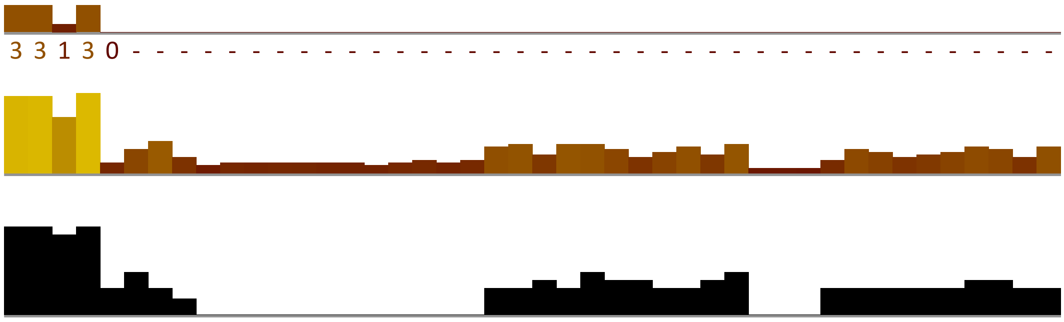

```
Conservation                33130---------------------------------------
Consensus                   EGGVGGTL------------IMHSLGETAFK---NALEPSKGYV
```

```
Pin_PITG_15679T0/1-271      --------------------------------------------
Pin_PITG_05841T0/1-220      --------------------------------------------
Pin_PITG_05846T0/1-263      --------------------------------------------
Prm_Prm_73724/1-343     271 YNSFWMKADDIFDQWPESIRYRIWVSFDDAEKMLKKRPQMAEIV 314
Pca_14162/1-348         297 AYAYMMKASTVYDDWAESIRYRIWLSYDDAITLLGKRDYMAKVV 340
Pin_PITG_06308T0/1-254      --------------------------------------------
Pin_PITG_11952T0/1-270  256 -RSWILGAVTIYTNSR---------------------------- 270
Pso_285308/1-314        269 YESFLMRSKKVYEQWSESRRLRVWVRECQSIEIPGETQLTVRLL 312
Pca_39353/1-267             --------------------------------------------
Pso_286675/1-96             --------------------------------------------
Prm_Prm_86166/1-353     281 YNSFWMKADDIFDQWPESIRYRIWVSFDDAEKMLKKRPQMAEIV 324
Prm_Prm_73707/1-269     197 YNSFWMKADDIFDQWPESIRYRIWVSFDDAEKMLKKRPQMAEIV 240
```

Conservation

Quality

Consensus

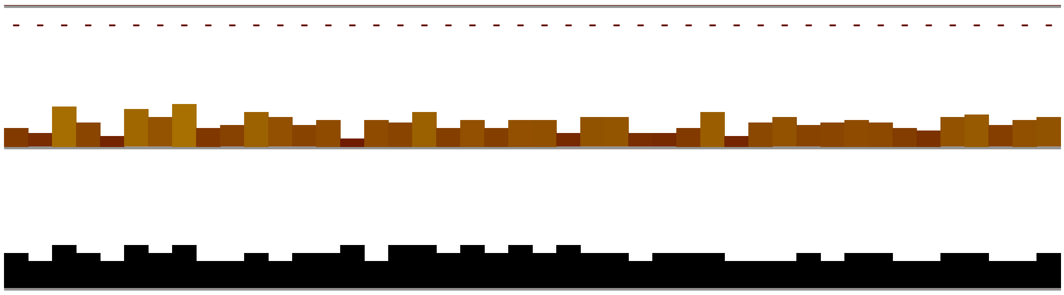

```
Conservation                --------------------------------------------
Consensus                   YNSFWMKADDI+DQWPESIRYRIWVSFDDAEKMLKKRPQMAEIV
```

```
Pin_PITG_15679T0/1-271      -----------------------------
Pin_PITG_05841T0/1-220      -----------------------------
Pin_PITG_05846T0/1-263      -----------------------------
Prm_Prm_73724/1-343     315 KATKEKAALVKTGDLPAMDDTLSKLKFEP 343
Pca_14162/1-348         341 STGSNAWW--------------------- 348
Pin_PITG_06308T0/1-254      -----------------------------
Pin_PITG_11952T0/1-270      -----------------------------
Pso_285308/1-314        313 FN--------------------------- 314
Pca_39353/1-267             -----------------------------
Pso_286675/1-96             -----------------------------
Prm_Prm_86166/1-353     325 KATKEKAALVKTGDLPAMDDTLSKLKFEP 353
Prm_Prm_73707/1-269     241 KATKEKAALVKTGDLPAMDDTLSKLKFEP 269
```

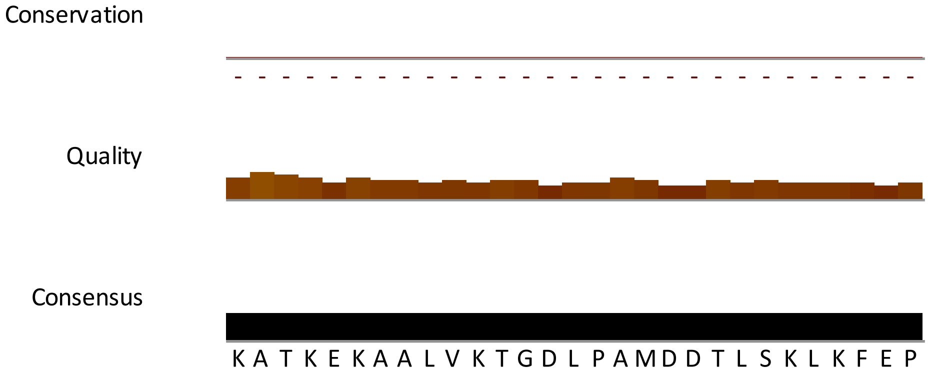
Conservation
Quality
Consensus
K A T K E K A A L V K T G D L P A M D D T L S K L K F E P

Ortholog 5

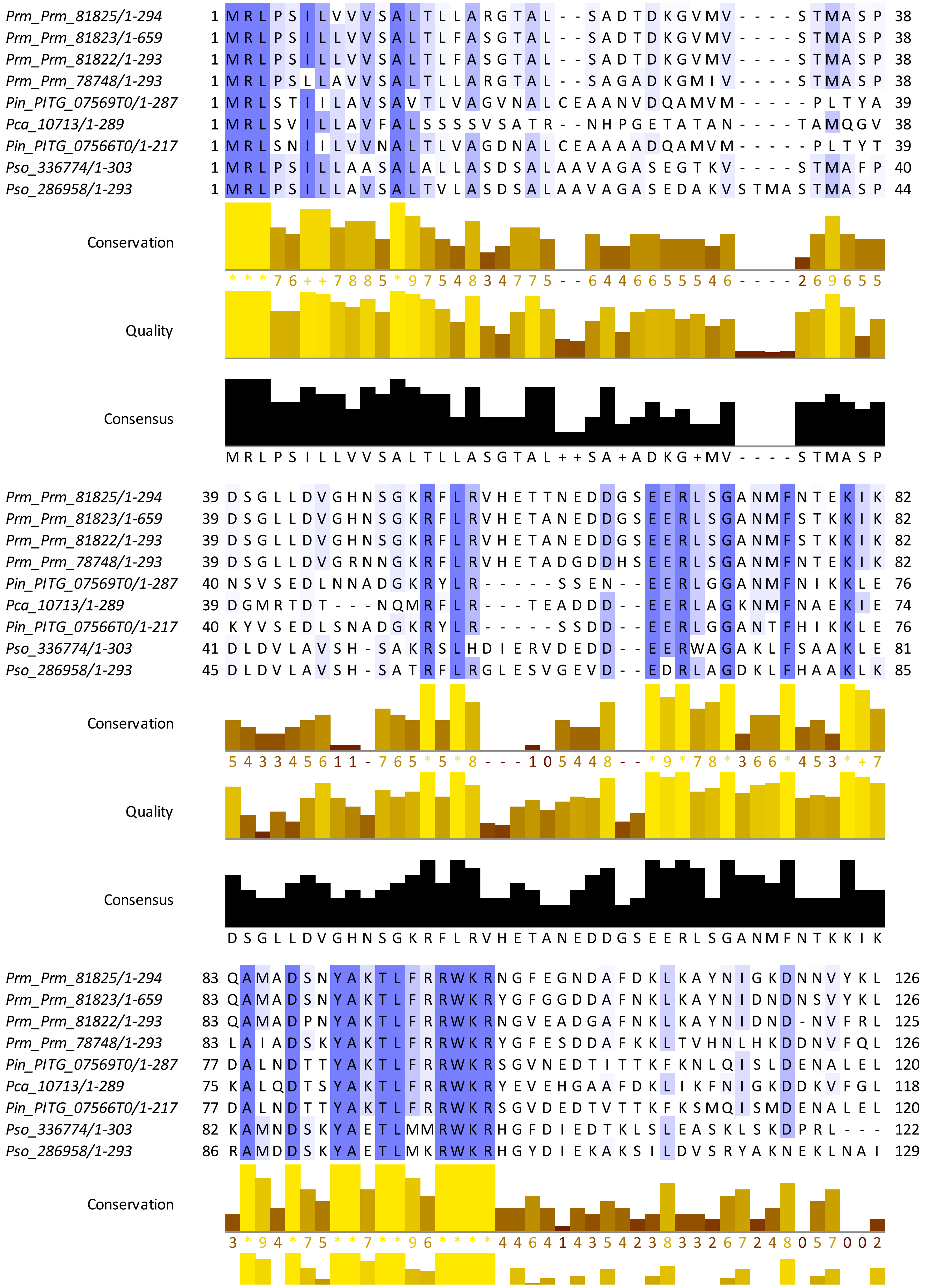
Prm_Prm_81825/1-294 1 MRLPSILVVVSALTLLARGTAL--SADTDKGVMV----STMASP 38
Prm_Prm_81823/1-659 1 MRLPSILLVVSALTLFASGTAL--SADTDKGVMV----STMASP 38
Prm_Prm_81822/1-293 1 MRLPSILLVVSALTLFASGTAL--SADTDKGVMV----STMASP 38
Prm_Prm_78748/1-293 1 MRLPSLLAVVSALTLLARGTAL--SAGADKGMIV----STMASP 38
Pin_PITG_07569T0/1-287 1 MRLSTIILAVSAVTLVAGVNALCEAANVDQAMVM-----PLTYA 39
Pca_10713/1-289 1 MRLSVILLAVFALSSSSVSATR--NHPGETATAN----TAMQGV 38
Pin_PITG_07566T0/1-217 1 MRLSNIILVVNALTLVAGDNALCEAAAADQAMVM-----PLTYT 39
Pso_336774/1-303 1 MRLPSILLAASALALLASDSALAAVAGASEGTKV----STMAFP 40
Pso_286958/1-293 1 MRLPSILLAVSALTVLASDSALAAVAGASEDAKVSTMASTMASP 44
Conservation
***76++7885*975483477 5--6446655546----269655
Quality
Consensus
MRLPSILLVVSALTLLASGTAL++SA+ADKG+MV----STMASP
Prm_Prm_81825/1-294 39 DSGLLDVGHNSGKRFLRVHETTNEDDGSEERLSGANMFNTEKIK 82
Prm_Prm_81823/1-659 39 DSGLLDVGHNSGKRFLRVHETANEDDGSEERLSGANMFSTKKIK 82
Prm_Prm_81822/1-293 39 DSGLLDVGHNSGKRFLRVHETANEDDGSEERLSGANMFSTKKIK 82
Prm_Prm_78748/1-293 39 DSGLLDVGRNNGKRFLRVHETADGDDHSEERLSGANMFNTEKIK 82
Pin_PITG_07569T0/1-287 40 NSVSEDLNNADGKRYLR-----SSEN--EERLGGANMFNIKKLE 76
Pca_10713/1-289 39 DGMRTDT---NQMRFLR---TEADDD--EERLAGKNMFNAEKIE 74
Pin_PITG_07566T0/1-217 40 KYVSEDLSNADGKRYLR-----SSDD--EERLGGANTFHIKKLE 76
Pso_336774/1-303 41 DLDVLAVSH-SAKRSLHDIERVDEDD--EERWAGAKLFSAAKLE 81
Pso_286958/1-293 45 DLDVLAVSH-SATRFLRGLESVGEVD--EDRLAGDKLFHAAKLK 85
Conservation
54334561 1-765*5*8---105448--*9*78*366*453*+7
Quality
Consensus
DSGLLDVGHNSGKRFLRVHETANEDDGSEERLSGANMFNTKKIK
Prm_Prm_81825/1-294 83 QAMADSNYAKTLFRRWKRNGFEGNDAFDKLKAYNIGKDNNVYKL 126
Prm_Prm_81823/1-659 83 QAMADSNYAKTLFRRWKRYGFGGDDAFNKLKAYNIDNDNSVYKL 126
Prm_Prm_81822/1-293 83 QAMADPNYAKTLFRRWKRNGVEADGAFNKLKAYNIDND-NVFRL 125
Prm_Prm_78748/1-293 83 LAIADSKYAKTLFRRWKRYGFESDDAFKKLTVHNLHKDDNVFQL 126
Pin_PITG_07569T0/1-287 77 DALNDTTYAKTLFRRWKRSGVNEDTITTKFKNLQISLDENALEL 120
Pca_10713/1-289 75 KALQDTSYAKTLFRRWKRYEVEHGAAFDKLIKFNIGKDDKVFGL 118
Pin_PITG_07566T0/1-217 77 DALNDTTYAKTLFRRWKRSGVDEDTVTTKFKSMQISMDENALEL 120
Pso_336774/1-303 82 KAMNDSKYAETLMMRWKRHGFDIEDTKLSLEASKLSKDPRL--- 122
Pso_286958/1-293 86 RAMDDSKYAETLMKRWKRHGYDIEKAKSILDVSRYAKNEKLNAI 129
Conservation
3*94*75**7**96****44641435423833267248057002

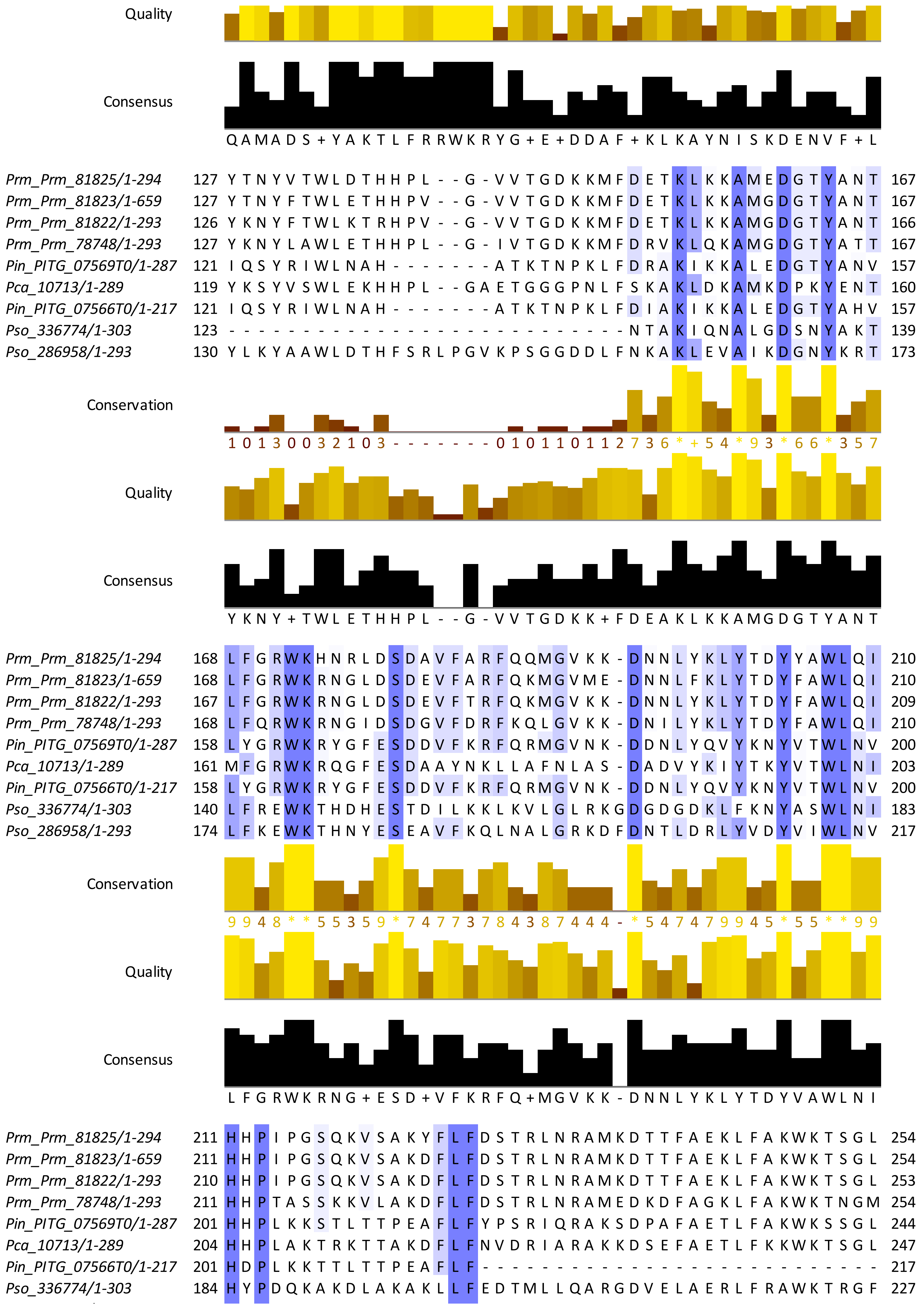
Quality
Consensus
QAMADS+YAKTLFRRWKRYG+E+DDAF+KLKAYNISKDENVF+L
Prm_Prm_81825/1-294 127 YTNYVTWLDTHHPL--G-VVTGDKKMFDETKLKKAMEDGTYANT 167
Prm_Prm_81823/1-659 127 YTNYFTWLETHHPV--G-VVTGDKKMFDETKLKKAMGDGTYANT 167
Prm_Prm_81822/1-293 126 YKNYFTWLKTRHPV--G-VVTGDKKMFDETKLKKAMGDGTYANT 166
Prm_Prm_78748/1-293 127 YKNYLAWLETHHPL--G-IVTGDKKMFDRVKLQKAMGDGTYATT 167
Pin_PITG_07569T0/1-287 121 IQSYRIWLNAH-------ATKTNPKLFDRAKIKKALEDGTYANV 157
Pca_10713/1-289 119 YKSYVSWLEKHHPL--GAETGGGPNLFSKAKLDKAMKDPKYENT 160
Pin_PITG_07566T0/1-217 121 IQSYRIWLNAH-------ATKTNPKLFDIAKIKKALEDGTYAHV 157
Pso_336774/1-303 123 --------------------------NTAKIQNALGDSNYAKT 139
Pso_286958/1-293 130 YLKYAAWLDTHFSRLPGVKPSGGDDLFNKAKLEVAIKDGNYKRT 173
Conservation
10130032103-------0101101127 36*+54*93*66*357
Quality
Consensus
YKNY+TWLETHHPL--G-VVTGDKK+FDEAKLKKAMGDGTYANT
Prm_Prm_81825/1-294 168 LFGRWKHNRLDSDAVFARFQQMGVKK-DNNLYKLYTDYYAWLQI 210
Prm_Prm_81823/1-659 168 LFGRWKRNGLDSDEVFARFQKMGVME-DNNLFKLYTDYFAWLQI 210
Prm_Prm_81822/1-293 167 LFGRWKRNGLDSDEVFTRFQKMGVKK-DNNLYKLYTDYFAWLQI 209
Prm_Prm_78748/1-293 168 LFQRWKRNGIDSDGVFDRFKQLGVKK-DNILYKLYTDYFAWLQI 210
Pin_PITG_07569T0/1-287 158 LYGRWKRYGFESDDVFKRFQRMGVNK-DDNLYQVYKNYVTWLNV 200
Pca_10713/1-289 161 MFGRWKRQGFESDAAYNKLLAFNLAS-DADVYKIYTKYVTWLNI 203
Pin_PITG_07566T0/1-217 158 LYGRWKRYGFESDDVFKRFQRMGVNK-DDNLYQVYKNYVTWLNV 200
Pso_336774/1-303 140 LFREWKTHDHESTDILKKLKVLGLRKGDGDGDKLFKNYASWLNI 183
Pso_286958/1-293 174 LFKEWKTHNYESEAVFKQLNALGRKDFDNTLDRLYVDYVIWLNV 217
Conservation
9948**55359*7477378438744 4-*547479945*55**99
Quality
Consensus
LFGRWKRNG+ESD+VFKRFQ+MGVKK-DNNLYKLYTDYVAWLNI
Prm_Prm_81825/1-294 211 HHPIPGSQKVSAKYFLFDSTRLNRAMKDTTFAEKLFAKWKTSGL 254
Prm_Prm_81823/1-659 211 HHPIPGSQKVSAKDFLFDSTRLNRAMKDTTFAEKLFAKWKTSGL 254
Prm_Prm_81822/1-293 210 HHPIPGSQKVSAKDFLFDSTRLNRAMKDTTFAEKLFAKWKTSGL 253
Prm_Prm_78748/1-293 211 HHPTASSKKVLAKDFLFDSTRLNRAMEDKDFAGKLFAKWKTNGM 254
Pin_PITG_07569T0/1-287 201 HHPLKKSTLTTPEAFLFYPSRIQRAKSDPAFAETLFAKWKSSGL 244
Pca_10713/1-289 204 HHPLAKTRKTTAKDFLFNVDRIARAKKDSEFAETLFKKWKTSGL 247
Pin_PITG_07566T0/1-217 201 HDPLKKTTLTTPEAFLF--------------------------- 217
Pso_336774/1-303 184 HYPDQKAKDLAKAKLLFEDTMLLQARGDVELAERLFRAWKTRGF 227

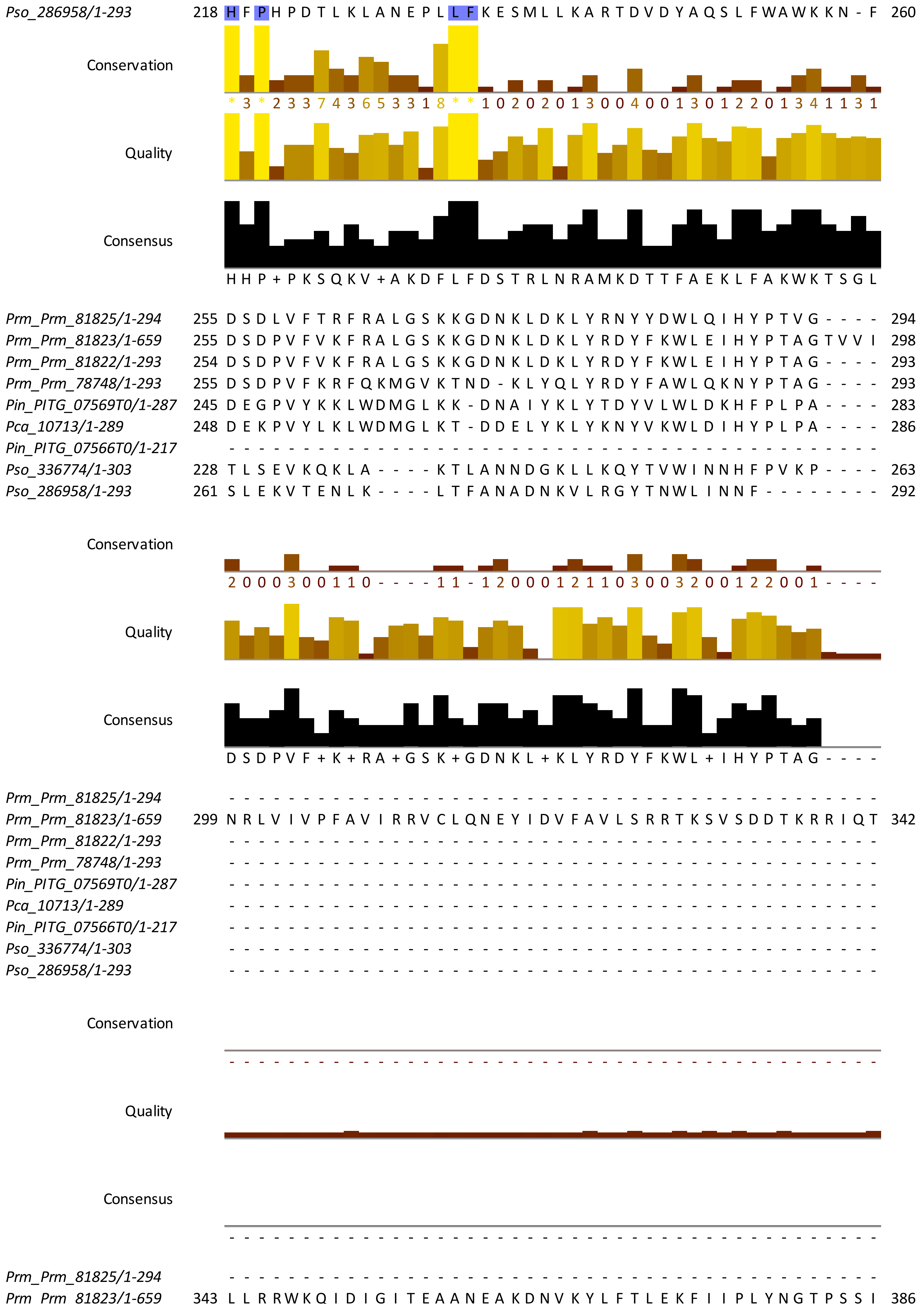

Pso_286958/1-293 218 HFPHPDTLKLANEPLLFKESMLLKARTDVDYAQSLFWAWKKN-F 260
Conservation
* 3 * 2 3 3 7 4 3 6 5 3 3 1 8 * * 1 0 2 0 2 0 1 3 0 0 4 0 0 1 3 0 1 2 2 0 1 3 4 1 1 3 1
Quality
Consensus
HHP+PKSQKV+AKDFLFDSTRLNRAMKDTTFAEKLFAKWKTSGL
Prm_Prm_81825/1-294 255 DSDLVFTRFRALGSKKGDNKLDKLYRNYYDWLQIHYPTVG---- 294
Prm_Prm_81823/1-659 255 DSDPVFVKFRALGSKKGDNKLDKLYRDYFKWLEIHYPTAGTVVI 298
Prm_Prm_81822/1-293 254 DSDPVFVKFRALGSKKGDNKLDKLYRDYFKWLEIHYPTAG---- 293
Prm_Prm_78748/1-293 255 DSDPVFKRFQKMGVKTND-KLYQLYRDYFAWLQKNYPTAG---- 293
Pin_PITG_07569T0/1-287 245 DEGPVYKKLWDMGLKK-DNAIYKLYTDYVLWLDKHFPLPA---- 283
Pca_10713/1-289 248 DEKPVYLKLWDMGLKT-DDELYKLYKNYVKWLDIHYPLPA---- 286
Pin_PITG_07566T0/1-217 --------------------------------------------
Pso_336774/1-303 228 TLSEVKQKLA----KTLANNDGKLLKQYTVWINNHFPVKP---- 263
Pso_286958/1-293 261 SLEKVTENLK----LTFANADNKVLRGYTNWLINNF-------- 292
Conservation
2 0 0 0 3 0 0 1 1 0 - - - - 1 1 - 1 2 0 0 0 1 2 1 1 0 3 0 0 3 2 0 0 1 2 2 0 0 1 - - - -
Quality
Consensus
DSDPVF+K+RA+GSK+GDNKL+KLYRDYFKWL+IHYPTAG----
Prm_Prm_81825/1-294 --------------------------------------------
Prm_Prm_81823/1-659 299 NRLVIVPFAVIRRVCLQNEYIDVFAVLSRRTKSVSDDTKRRIQT 342
Prm_Prm_81822/1-293 --------------------------------------------
Prm_Prm_78748/1-293 --------------------------------------------
Pin_PITG_07569T0/1-287 --------------------------------------------
Pca_10713/1-289 --------------------------------------------
Pin_PITG_07566T0/1-217 --------------------------------------------
Pso_336774/1-303 --------------------------------------------
Pso_286958/1-293 --------------------------------------------
Conservation
--------------------------------------------
Quality
Consensus
--------------------------------------------
Prm_Prm_81825/1-294 --------------------------------------------
Prm_Prm_81823/1-659 343 LLRRWKQIDIGITEAANEAKDNVKYLFTLEKFIIPLYNGTPSSI 386

[illegible]

Prm_Prm_81822/1-293 - - - - - - - - - - - - - - - - - - - - - - - - - - - - - - - - - - - - - - - - - - - -
Prm_Prm_78748/1-293 - - - - - - - - - - - - - - - - - - - - - - - - - - - - - - - - - - - - - - - - - - - -
Pin_PITG_07569T0/1-287 - - - - - - - - - - - - - - - - - - - - - - - - - - - - - - - - - - - - - - - - - - - -
Pca_10713/1-289 - - - - - - - - - - - - - - - - - - - - - - - - - - - - - - - - - - - - - - - - - - - -
Pin_PITG_07566T0/1-217 - - - - - - - - - - - - - - - - - - - - - - - - - - - - - - - - - - - - - - - - - - - -
Pso_336774/1-303 - - - - - - - - - - - - - - - - - - - - - - - - - - - - - - - - - - - - - - - - - - - -
Pso_286958/1-293 - - - - - - - - - - - - - - - - - - - - - - - - - - - - - - - - - - - - - - - - - - - -

Conservation

- - - - - - - - - - - - - - - - - - - - - - - - - - - - - - - - - - - - - - - - - - - -

Quality

Consensus

- - - - - - - - - - - - - - - - - - - - - - - - - - - - - - - - - - - - - - - - - - - -

Prm_Prm_81825/1-294 - - - - - - - - - - - - - - - - - - - - - - - - - - - - - - - - - - - - - - - - - - - -
Prm_Prm_81823/1-659 387 I D T L P A L M N S I K M I H S I A R Y Y N T T E R M A N L F T K I T N Q M I T N C K H 430
Prm_Prm_81822/1-293 - - - - - - - - - - - - - - - - - - - - - - - - - - - - - - - - - - - - - - - - - - - -
Prm_Prm_78748/1-293 - - - - - - - - - - - - - - - - - - - - - - - - - - - - - - - - - - - - - - - - - - - -
Pin_PITG_07569T0/1-287 - - - - - - - - - - - - - - - - - - - - - - - - - - - - - - - - - - - - - - - - - - - -
Pca_10713/1-289 - - - - - - - - - - - - - - - - - - - - - - - - - - - - - - - - - - - - - - - - - - - -
Pin_PITG_07566T0/1-217 - - - - - - - - - - - - - - - - - - - - - - - - - - - - - - - - - - - - - - - - - - - -
Pso_336774/1-303 - - - - - - - - - - - - - - - - - - - - - - - - - - - - - - - - - - - - - - - - - - - -
Pso_286958/1-293 - - - - - - - - - - - - - - - - - - - - - - - - - - - - - - - - - - - - - - - - - - - -

Conservation

- - - - - - - - - - - - - - - - - - - - - - - - - - - - - - - - - - - - - - - - - - - -

Quality

Consensus

- - - - - - - - - - - - - - - - - - - - - - - - - - - - - - - - - - - - - - - - - - - -

Prm_Prm_81825/1-294 - - - - - - - - - - - - - - - - - - - - - - - - - - - - - - - - - - - - - - - - - - - -
Prm_Prm_81823/1-659 431 F M W D K D P E E L V R H L D S C L K L N E A Y Q Q Q Y R V T K D K L F A T P K G K Q F 474
Prm_Prm_81822/1-293 - - - - - - - - - - - - - - - - - - - - - - - - - - - - - - - - - - - - - - - - - - - -
Prm_Prm_78748/1-293 - - - - - - - - - - - - - - - - - - - - - - - - - - - - - - - - - - - - - - - - - - - -
Pin_PITG_07569T0/1-287 - - - - - - - - - - - - - - - - - - - - - - - - - - - - - - - - - - - - - - - - - - - -
Pca_10713/1-289 - - - - - - - - - - - - - - - - - - - - - - - - - - - - - - - - - - - - - - - - - - - -
Pin_PITG_07566T0/1-217 - - - - - - - - - - - - - - - - - - - - - - - - - - - - - - - - - - - - - - - - - - - -
Pso_336774/1-303 - - - - - - - - - - - - - - - - - - - - - - - - - - - - - - - - - - - - - - - - - - - -
Pso_286958/1-293 - - - - - - - - - - - - - - - - - - - - - - - - - - - - - - - - - - - - - - - - - - - -

Conservation

- - - - - - - - - - - - - - - - - - - - - - - - - - - - - - - - - - - - - - - - - - - -

Quality

```
Consensus
                              - - - - - - - - - - - - - - - - - - - - - - - - - - - - - - - - - - - - - - - - - - - -

Prm_Prm_81825/1-294           - - - - - - - - - - - - - - - - - - - - - - - - - - - - - - - - - - - - - - - - - - - -
Prm_Prm_81823/1-659       475 E F N E M K I F G K F N L F C R R V M K L I D M F T T I P P V L V A A H G R H G S A V G 518
Prm_Prm_81822/1-293           - - - - - - - - - - - - - - - - - - - - - - - - - - - - - - - - - - - - - - - - - - - -
Prm_Prm_78748/1-293           - - - - - - - - - - - - - - - - - - - - - - - - - - - - - - - - - - - - - - - - - - - -
Pin_PITG_07569T0/1-287        - - - - - - - - - - - - - - - - - - - - - - - - - - - - - - - - - - - - - - - - - - - -
Pca_10713/1-289               - - - - - - - - - - - - - - - - - - - - - - - - - - - - - - - - - - - - - - - - - - - -
Pin_PITG_07566T0/1-217        - - - - - - - - - - - - - - - - - - - - - - - - - - - - - - - - - - - - - - - - - - - -
Pso_336774/1-303              - - - - - - - - - - - - - - - - - - - - - - - - - - - - - - - - - - - - - - - - - - - -
Pso_286958/1-293              - - - - - - - - - - - - - - - - - - - - - - - - - - - - - - - - - - - - - - - - - - - -

Conservation
                              - - - - - - - - - - - - - - - - - - - - - - - - - - - - - - - - - - - - - - - - - - - -

Quality

Consensus
                              - - - - - - - - - - - - - - - - - - - - - - - - - - - - - - - - - - - - - - - - - - - -

Prm_Prm_81825/1-294           - - - - - - - - - - - - - - - - - - - - - - - - - - - - - - - - - - - - - - - - - - - -
Prm_Prm_81823/1-659       519 G T V S I N S Y G R H L S D S Y R D L N P A Y F H I A A V I L L G S L N S I F I V D V T 562
Prm_Prm_81822/1-293           - - - - - - - - - - - - - - - - - - - - - - - - - - - - - - - - - - - - - - - - - - - -
Prm_Prm_78748/1-293           - - - - - - - - - - - - - - - - - - - - - - - - - - - - - - - - - - - - - - - - - - - -
Pin_PITG_07569T0/1-287        - - - - - - - - - - - - - - - - - - - - - - - - - - - - - - - - - - - - - - - - - - - -
Pca_10713/1-289               - - - - - - - - - - - - - - - - - - - - - - - - - - - - - - - - - - - - - - - - - - - -
Pin_PITG_07566T0/1-217        - - - - - - - - - - - - - - - - - - - - - - - - - - - - - - - - - - - - - - - - - - - -
Pso_336774/1-303              - - - - - - - - - - - - - - - - - - - - - - - - - - - - - - - - - - - - - - - - - - - -
Pso_286958/1-293              - - - - - - - - - - - - - - - - - - - - - - - - - - - - - - - - - - - - - - - - - - - -

Conservation
                              - - - - - - - - - - - - - - - - - - - - - - - - - - - - - - - - - - - - - - - - - - - -

Quality

Consensus
                              - - - - - - - - - - - - - - - - - - - - - - - - - - - - - - - - - - - - - - - - - - - -

Prm_Prm_81825/1-294           - - - - - - - - - - - - - - - - - - - - - - - - - - - - - - - - - - - - - - - - - - - -
Prm_Prm_81823/1-659       563 A G S E S S A A K S T M Y A K T R R L S W I F E T Y T D G P L V P S G S Y T T G A Y Y Y 606
Prm_Prm_81822/1-293           - - - - - - - - - - - - - - - - - - - - - - - - - - - - - - - - - - - - - - - - - - - -
Prm_Prm_78748/1-293           - - - - - - - - - - - - - - - - - - - - - - - - - - - - - - - - - - - - - - - - - - - -
Pin_PITG_07569T0/1-287        - - - - - - - - - - - - - - - - - - - - - - - - - - - - - - - - - - - - - - - - - - - -
Pca_10713/1-289               - - - - - - - - - - - - - - - - - - - - - - - - - - - - - - - - - - - - - - - - - - - -
Pin_PITG_07566T0/1-217        - - - - - - - - - - - - - - - - - - - - - - - - - - - - - - - - - - - - - - - - - - - -
Pso_336774/1-303              - - - - - - - - - - - - - - - - - - - - - - - - - - - - - - - - - - - - - - - - - - - -
Pso_286958/1-293              - - - - - - - - - - - - - - - - - - - - - - - - - - - - - - - - - - - - - - - - - - - -

Conservation
```

```
Conservation
                          - - - - - - - - - - - - - - - - - - - - - - - - - - - - - - - - - - - - - - - - - - - -

Quality

Consensus
                          - - - - - - - - - - - - - - - - - - - - - - - - - - - - - - - - - - - - - - - - - - - -

Prm_Prm_81825/1-294           - - - - - - - - - - - - - - - - - - - - - - - - - - - - - - - - - - - - - - - - - - - -
Prm_Prm_81823/1-659       607 Y L L E M D D A G A I I D G E W H Q S S Y A D V S M L A C S G S S S G Y E A V G S S S T 650
Prm_Prm_81822/1-293           - - - - - - - - - - - - - - - - - - - - - - - - - - - - - - - - - - - - - - - - - - - -
Prm_Prm_78748/1-293           - - - - - - - - - - - - - - - - - - - - - - - - - - - - - - - - - - - - - - - - - - - -
Pin_PITG_07569T0/1-287        - - - - - - - - - - - - - - - - - - - - - - - - - - - - - - - - - - - - - - - - - - - -
Pca_10713/1-289               - - - - - - - - - - - - - - - - - - - - - - - - - - - - - - - - - - - - - - - - - - - -
Pin_PITG_07566T0/1-217        - - - - - - - - - - - - - - - - - - - - - - - - - - - - - - - - - - - - - - - - - - - -
Pso_336774/1-303          264 - - - - - - - - - - - - - T Q A S P I V A I L D R V E V V S G A S D G S N S I G T G E E 294
Pso_286958/1-293              - - - - - - - - - - - - - - - - - - - - - - - - - - - - - - - - - - - - - - - - - - - -

Conservation
                              - - - - - - - - - - - - - - - - - - - - - - - - - - - - - - - - - - - - - - - - - - - -

Quality

Consensus
                              - - - - - - - - - - - - - - - - - - - - - - - - - - - - - S G - S - G - - - - G - - - -

Prm_Prm_81825/1-294           - - - - - - - - -
Prm_Prm_81823/1-659       651 S D R C S R E P L                                                                     659
Prm_Prm_81822/1-293           - - - - - - - - -
Prm_Prm_78748/1-293           - - - - - - - - -
Pin_PITG_07569T0/1-287    284 - - - - - K A T N                                                                     287
Pca_10713/1-289           287 - - - - - - K A T                                                                     289
Pin_PITG_07566T0/1-217        - - - - - - - - -
Pso_336774/1-303          295 T H G S A K K P Q                                                                     303
Pso_286958/1-293              - - - - - - - - H                                                                     293

Conservation
                              - - - - - - - - -

Quality

Consensus
                              - - - - - K K P -
```

# Ortholog 6

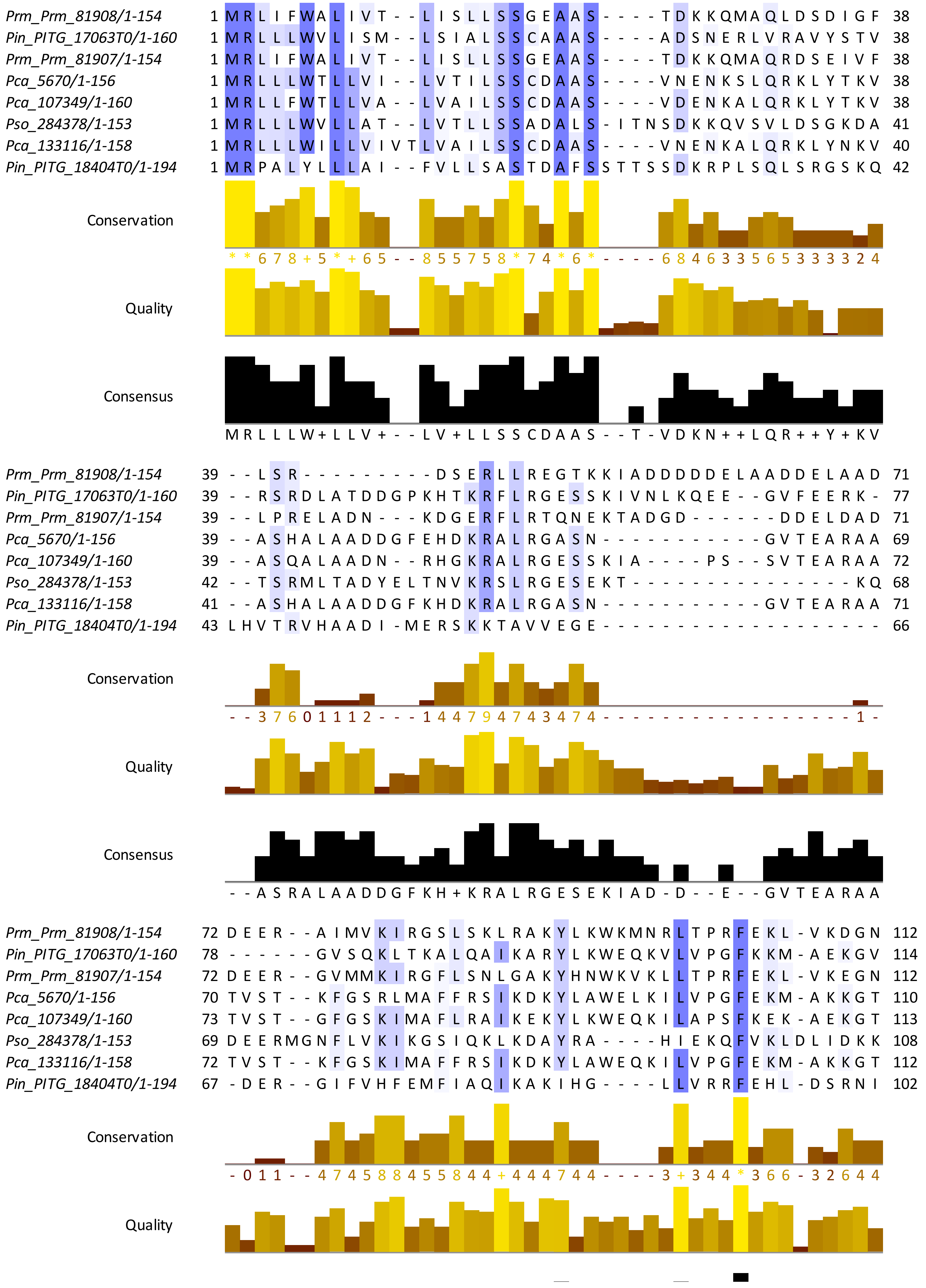
Prm_Prm_81908/1-154 1 MRLIFWALIVT--LISLLSSGEAAS----TDKKQMAQLDSDIGF 38
Pin_PITG_17063T0/1-160 1 MRLLLWVLISM--LSIALSSCAAAS----ADSNERLVRAVYSTV 38
Prm_Prm_81907/1-154 1 MRLIFWALIVT--LISLLSSGEAAS----TDKKQMAQRDSEIVF 38
Pca_5670/1-156 1 MRLLLWTLLVI--LVTILSSCDAAS----VNENKSLQRKLYTKV 38
Pca_107349/1-160 1 MRLLFWTLLVA--LVAILSSCDAAS----VDENKALQRKLYTKV 38
Pso_284378/1-153 1 MRLLLWVLLAT--LVTLLSSADALS-ITNSDKKQVSVLDSGKDA 41
Pca_133116/1-158 1 MRLLLWILLVIVTLVAILSSCDAAS----VNENKALQRKLYNKV 40
Pin_PITG_18404T0/1-194 1 MRPALYLLLAI--FVLLSASTDAFSSTTSSDKRPLSQLSRGSKQ 42
Conservation
**678+5*+65--855758*74*6*----6846335653333324
Quality
Consensus
MRLLLW+LLV+--LV+LLSSCDAAS--T-VDKN++LQR++Y+KV
Prm_Prm_81908/1-154 39 --LSR----------DSERLLREGTKKIADDDDDELAADDELAAD 71
Pin_PITG_17063T0/1-160 39 --RSRDLATDDGPKHTKRFLRGESSKIVNLKQEE--GVFEERK- 77
Prm_Prm_81907/1-154 39 --LPRELADN---KDGERFLRTQNEKTADGD------DDELDAD 71
Pca_5670/1-156 39 --ASHALAADDGFEHDKRALRGASN-----------GVTEARAA 69
Pca_107349/1-160 39 --ASQALAADN--RHGKRALRGESSKIA----PS--SVTEARAA 72
Pso_284378/1-153 42 --TSRMLTADYELTNVKRSLRGESEKT---------------KQ 68
Pca_133116/1-158 41 --ASHALAADDGFKHDKRALRGASN-----------GVTEARAA 71
Pin_PITG_18404T0/1-194 43 LHVTRVHAADI-MERSKKTAVVEGE------------------- 66
Conservation
--3760112---14479474347 4-------------------1-
Quality
Consensus
--ASRALAADDGFKH+KRALRGESEKIAD-D--E--GVTEARAA
Prm_Prm_81908/1-154 72 DEER--AIMVKIRGSLSKLRAKYLKWKMNRLTPRFEKL-VKDGN 112
Pin_PITG_17063T0/1-160 78 ------GVSQKLTKALQAIKARYLKWEQKVLVPGFKKM-AEKGV 114
Prm_Prm_81907/1-154 72 DEER--GVMMKIRGFLSNLGAKYHNWKVKLLTPRFEKL-VKEGN 112
Pca_5670/1-156 70 TVST--KFGSRLMAFFRSIKDKYLAWELKILVPGFEKM-AKKGT 110
Pca_107349/1-160 73 TVST--GFGSKIMAFLRAIKEKYLKWEQKILAPSFKEK-AEKGT 113
Pso_284378/1-153 69 DEERMGNFLVKIKGSIQKLKDAYRA----HIEKQFVKLDLIDKK 108
Pca_133116/1-158 72 TVST--KFGSKIMAFFRSIKDKYLAWEQKILVPGFEKM-AKKGT 112
Pin_PITG_18404T0/1-194 67 -DER--GIFVHFEMFIAQIKAKIHG----LLVRRFEHL-DSRNI 102
Conservation
-011--4745884558 44+444744----3+344*366-32644
Quality

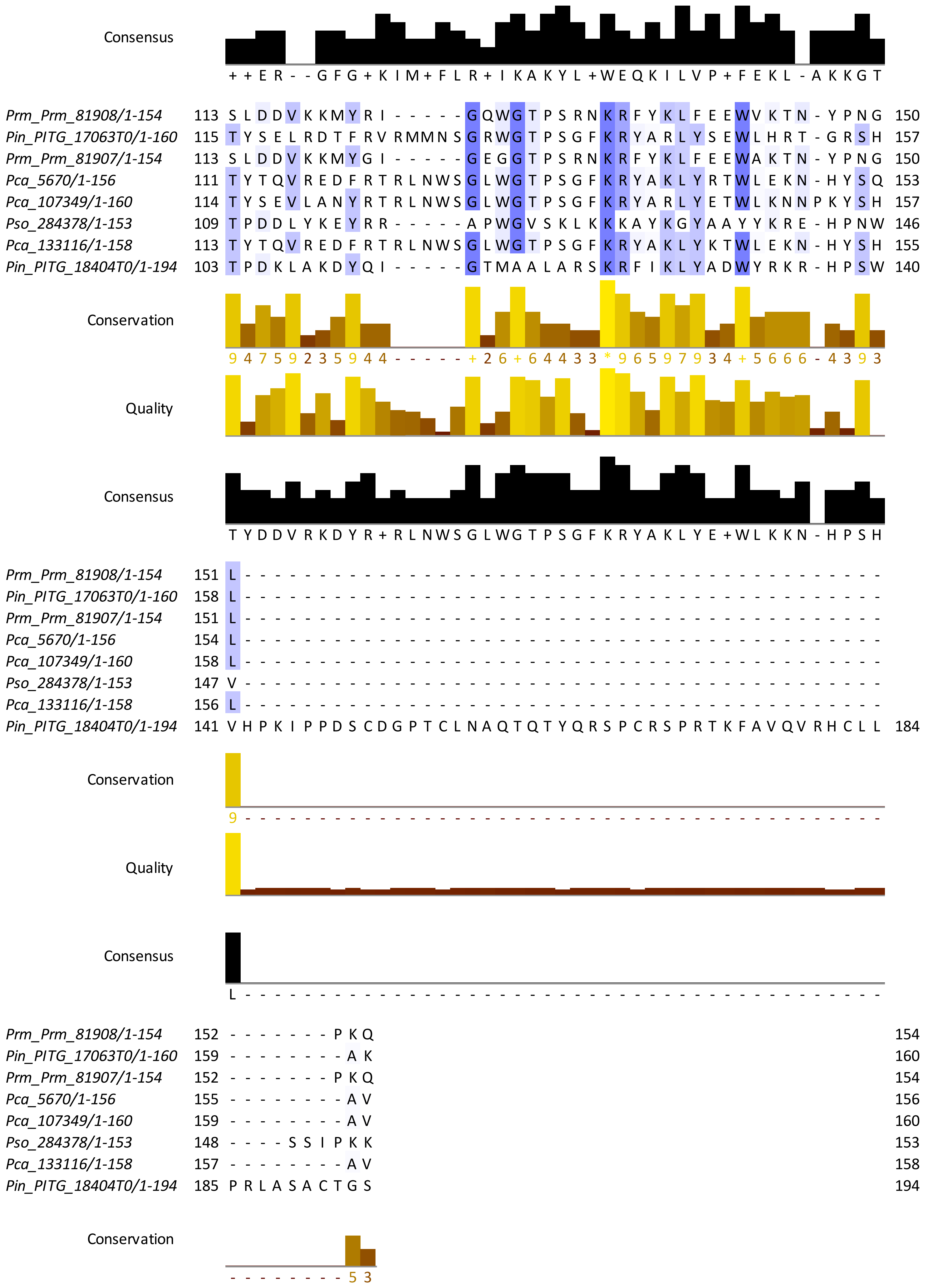
Consensus
++ER--GFG+KIM+FLR+IKAKYL+WEQKILVP+FEKL-AKKGT
Prm_Prm_81908/1-154 113 SLDDVKKMYRI-----GQWGTPSRNKRFYKLFEEWVKTN-YPNG 150
Pin_PITG_17063T0/1-160 115 TYSELRDTFRVRMMNSGRWGTPSGFKRYARLYSEWLHRT-GRSH 157
Prm_Prm_81907/1-154 113 SLDDVKKMYGI-----GEGGTPSRNKRFYKLFEEWAKTN-YPNG 150
Pca_5670/1-156 111 TYTQVREDFRTRLNWSGLWGTPSGFKRYAKLYRTWLEKN-HYSQ 153
Pca_107349/1-160 114 TYSEVLANYRTRLNWSGLWGTPSGFKRYARLYETWLKNNPKYSH 157
Pso_284378/1-153 109 TPDDLYKEYRR-----APWGVSKLKKKAYKGYAAYYKRE-HPNW 146
Pca_133116/1-158 113 TYTQVREDFRTRLNWSGLWGTPSGFKRYAKLYKTWLEKN-HYSH 155
Pin_PITG_18404T0/1-194 103 TPDKLAKDYQI-----GTMAALARSKRFIKLYADWYRKR-HPSW 140
Conservation
94759235944-----+26+64433*965979334+5666-4393
Quality
Consensus
TYDDVRKDYR+RLNWSGLWGTPSGFKRYAKLYE+WLKKN-HPSH
Prm_Prm_81908/1-154 151 L-------------------------------------------
Pin_PITG_17063T0/1-160 158 L-------------------------------------------
Prm_Prm_81907/1-154 151 L-------------------------------------------
Pca_5670/1-156 154 L-------------------------------------------
Pca_107349/1-160 158 L-------------------------------------------
Pso_284378/1-153 147 V-------------------------------------------
Pca_133116/1-158 156 L-------------------------------------------
Pin_PITG_18404T0/1-194 141 VHPKIPPDSCDGPTCLNAQTQTYQRSPCRSPRTKFAVQVRHCLL 184
Conservation
9-------------------------------------------
Quality
Consensus
L-------------------------------------------
Prm_Prm_81908/1-154 152 -------PKQ 154
Pin_PITG_17063T0/1-160 159 --------AK 160
Prm_Prm_81907/1-154 152 -------PKQ 154
Pca_5670/1-156 155 --------AV 156
Pca_107349/1-160 159 --------AV 160
Pso_284378/1-153 148 ----SSIPKK 153
Pca_133116/1-158 157 --------AV 158
Pin_PITG_18404T0/1-194 185 PRLASACTGS 194
Conservation
--------53

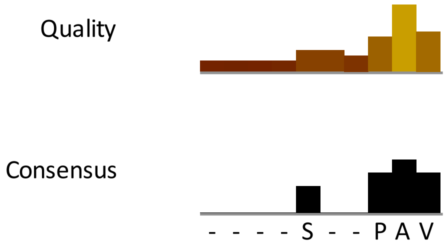
Quality
Consensus
- - - - S - - P A V

# Ortholog 7

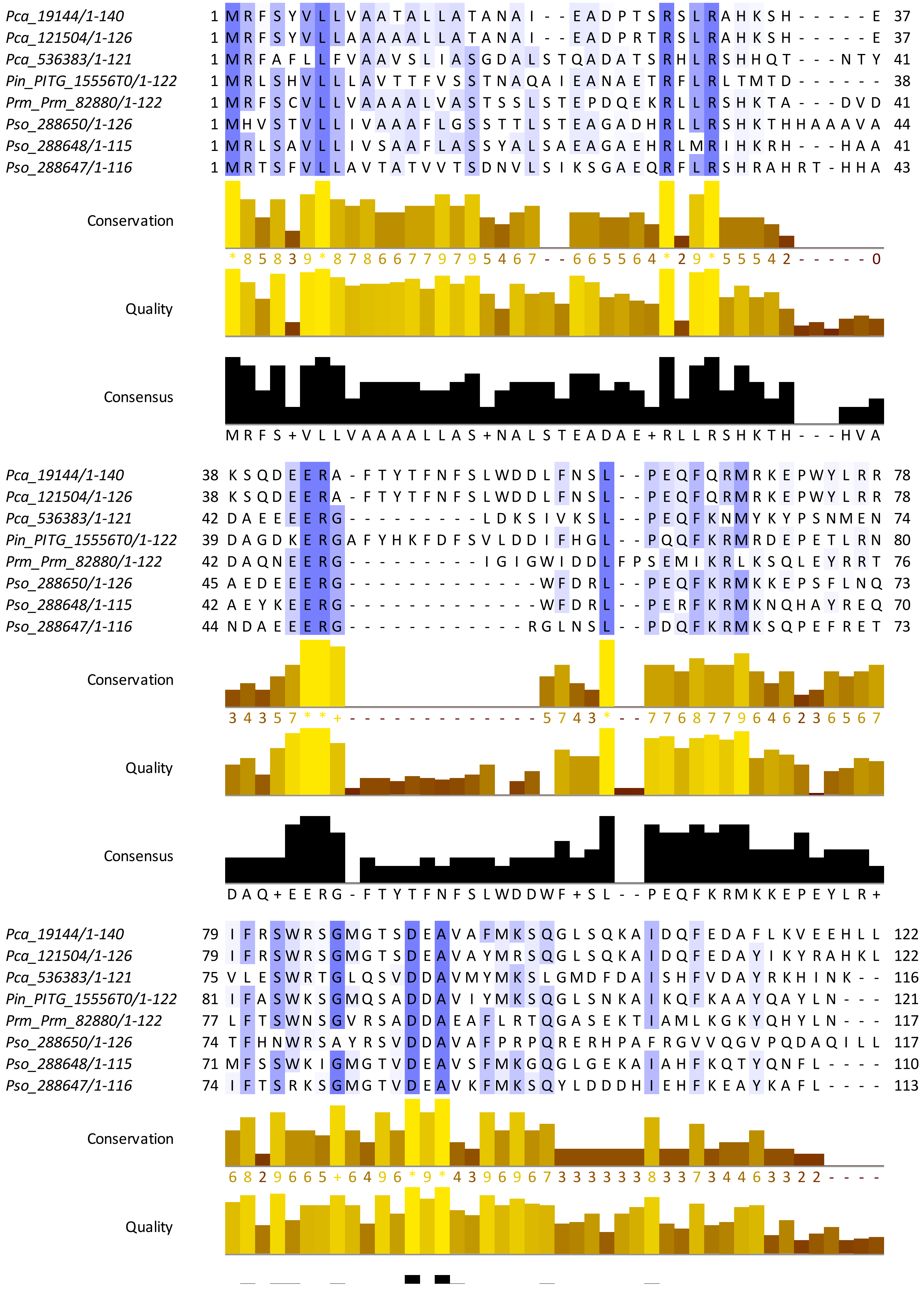

Pca_19144/1-140 1 MRFSYVLLVAATALLATANAI--EADPTSRSLRAHKSH-----E 37
Pca_121504/1-126 1 MRFSYVLLAAAAALLATANAI--EADPRTRSLRAHKSH-----E 37
Pca_536383/1-121 1 MRFAFLLFVAAVSLIASGDALSTQADATSRHLRSHHQT---NTY 41
Pin_PITG_15556T0/1-122 1 MRLSHVLLLAVTTFVSSTNAQAIEANAETRFLRLTMTD------ 38
Prm_Prm_82880/1-122 1 MRFSCVLLVAAAALVASTSSLSTEPDQEKRLLRSHKTA---DVD 41
Pso_288650/1-126 1 MHVSTVLLIVAAAFLGSSTTLSTEAGADHRLLRSHKTHHAAAVA 44
Pso_288648/1-115 1 MRLSAVLLIVSAAFLASSYALSAEAGAEHRLMRIHKRH---HAA 41
Pso_288647/1-116 1 MRTSFVLLAVTATVVTSDNVLSIKSGAEQRFLRSHRAHRT-HHA 43
Conservation
*8583 9*878667797954 67--665564*29*55542-----0
Quality
Consensus
MRFS+VLLVAAAALLAS+NALSTEADAE+RLLRSHKTH---HVA
Pca_19144/1-140 38 KSQDEERA-FTYTFNFSLWDDLFNSL--PEQFQRMRKEPWYLRR 78
Pca_121504/1-126 38 KSQDEERA-FTYTFNFSLWDDLFNSL--PEQFQRMRKEPWYLRR 78
Pca_536383/1-121 42 DAEEEERG---------LDKSIVKSL--PEQFKNMYKYPSNMEN 74
Pin_PITG_15556T0/1-122 39 DAGDKERGAFYHKFDFSVLDDIFHGL--PQQFKRMRDEPETLRN 80
Prm_Prm_82880/1-122 42 DAQNEERG---------IGIGWIDDLFPSEMIKRLKSQLEYRRT 76
Pso_288650/1-126 45 AEDEEERG-------------WFDRL--PEQFKRMKKEPSFLNQ 73
Pso_288648/1-115 42 AEYKEERG-------------WFDRL--PERFKRMKNQHAYREQ 70
Pso_288647/1-116 44 NDAEEERG------------RGLNSL--PDQFKRMKSQPEFRET 73
Conservation
34357**+-------------5743*--7768779646236567
Quality
Consensus
DAQ+EERG-FTYTFNFSLWDDWF+SL--PEQFKRMKKEPEYLR+
Pca_19144/1-140 79 IFRSWRSGMGTSDEAVAFMKSQGLSQKAIDQFEDAFLKVEEHLL 122
Pca_121504/1-126 79 IFRSWRSGMGTSDEAVAYMRSQGLSQKAIDQFEDAYIKYRAHKL 122
Pca_536383/1-121 75 VLESWRTGLQSVDDAVMYMKSLGMDFDAISHFVDAYRKHINK-- 116
Pin_PITG_15556T0/1-122 81 IFASWKSGMQSADDAVIYMKSQGLSNKAIKQFKAAYQAYLN--- 121
Prm_Prm_82880/1-122 77 LFTSWNSGVRSADDAEAFLRTQGASEKTIAMLKGKYQHYLN--- 117
Pso_288650/1-126 74 TFHNWRSAYRSVDDAVAFPRPQRERHPAFRGVVQGVPQDAQILL 117
Pso_288648/1-115 71 MFSSWKIGMGTVDEAVSFMKGQGLGEKAIAHFKQTYQNFL---- 110
Pso_288647/1-116 74 IFTSRKSGMGTVDEAVKFMKSQYLDDDHIEHFKEAYKAFL---- 113
Conservation
6829665+6496*9*4396967333333383373446332 2----
Quality

Consensus

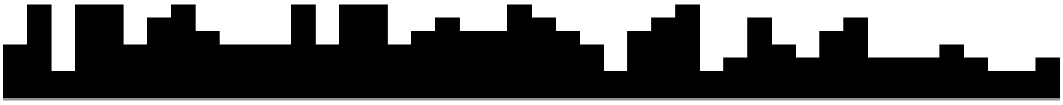

I F + S W R S G M G + V D + A V A F M K S Q G L S + K A I + + F K D A Y Q K Y L N H L L

| | | | |
|---|---|---|---|
| *Pca_19144/1-140* | 123 | N L L G I R Y S G E S L N P S G A G | 140 |
| *Pca_121504/1-126* | 123 | - - - - - - - - - - - - - - A K G K | 126 |
| *Pca_536383/1-121* | 117 | - - - - - - - - - - - - - K G L P Y | 121 |
| *Pin_PITG_15556T0/1-122* | | - - - - - - - - - - - - - - - - - H | 122 |
| *Prm_Prm_82880/1-122* | 118 | - - - - - - - - - - - - - T G T V T | 122 |
| *Pso_288650/1-126* | 118 | D A V E - - - - - - - - - M A S F F | 126 |
| *Pso_288648/1-115* | 111 | - - - - - - - - - - - - - A G R S H | 115 |
| *Pso_288647/1-116* | 114 | - - - - - - - - - - - - - - - R N N | 116 |

Conservation

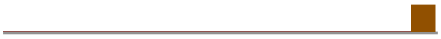

- - - - - - - - - - - - - - - 0 0 3

Quality

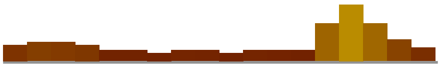

Consensus

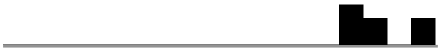

- - - - - - - - - - - - - - G R - H

## Ortholog 8

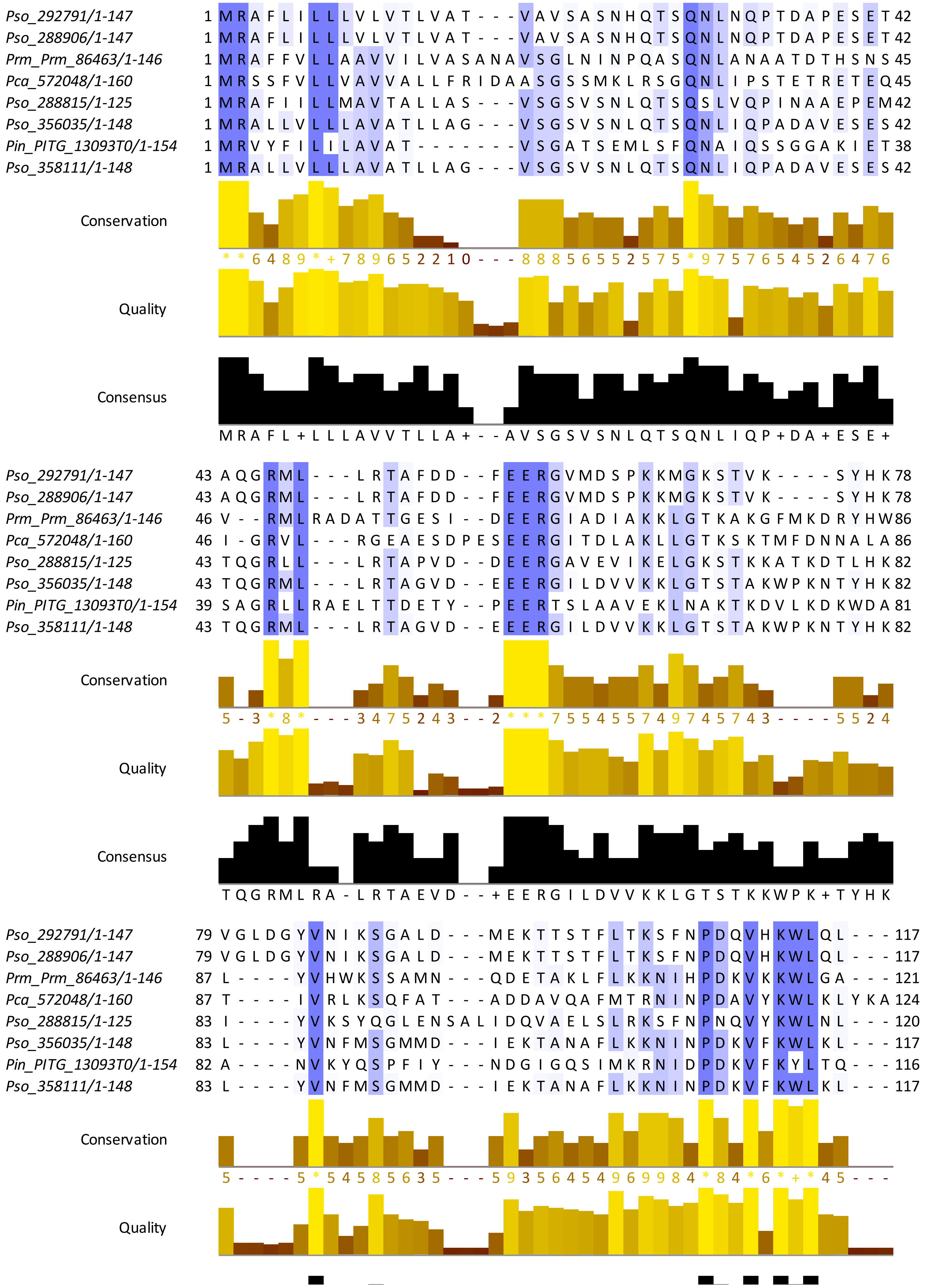
Pso_292791/1-147 1 MRAFLILLLVLVTLVAT---VAVSASNHQTSQNLNQPTDAPESET 42
Pso_288906/1-147 1 MRAFLILLLVLVTLVAT---VAVSASNHQTSQNLNQPTDAPESET 42
Prm_Prm_86463/1-146 1 MRAFFVLLAAVVILVASANAVSGLNINPQASQNLANAATDTHSNS 45
Pca_572048/1-160 1 MRSSFVLLVAVVALLFRIDAASGSSMKLRSGQNLIPSTETRETEQ 45
Pso_288815/1-125 1 MRAFIILLMAVTALLAS---VSGSVSNLQTSQSLVQPINAAEPEM 42
Pso_356035/1-148 1 MRALLVLLLAVATLLAG---VSGSVSNLQTSQNLIQPADAVESES 42
Pin_PITG_13093T0/1-154 1 MRVYFILILAVAT-------VSGATSEMLSFQNAIQSSGGAKIET 38
Pso_358111/1-148 1 MRALLVLLLAVATLLAG---VSGSVSNLQTSQNLIQPADAVESES 42
Conservation
* * 6 4 8 9 * + 7 8 9 6 5 2 2 1 0 - - - 8 8 8 5 6 5 5 2 5 7 5 * 9 7 5 7 6 5 4 5 2 6 4 7 6
Quality
Consensus
MRAFL+LLLAVVTLLA+--AVSGSVSNLQTSQNLIQP+DA+ESE+
Pso_292791/1-147 43 AQGRML---LRTAFDD--FEERGVMDSPKKMGKSTVK----SYHK 78
Pso_288906/1-147 43 AQGRML---LRTAFDD--FEERGVMDSPKKMGKSTVK----SYHK 78
Prm_Prm_86463/1-146 46 V--RMLRADATTGESI--DEERGIADIAKKLGTKAKGFMKDRYHW 86
Pca_572048/1-160 46 I-GRVL---RGEAESDPESEERGITDLAKLLGTKSKTMFDNNALA 86
Pso_288815/1-125 43 TQGRLL---LRTAPVD--DEERGAVEVIKELGKSTKKATKDTLHK 82
Pso_356035/1-148 43 TQGRML---LRTAGVD--EEERGILDVVKKLGTSTAKWPKNTYHK 82
Pin_PITG_13093T0/1-154 39 SAGRLLRAELTTDETY--PEERTSLAAVEKLNAKTKDVLKDKWDA 81
Pso_358111/1-148 43 TQGRML---LRTAGVD--EEERGILDVVKKLGTSTAKWPKNTYHK 82
Conservation
5 - 3 * 8 * - - - 3 4 7 5 2 4 3 - - 2 * * * 7 5 5 4 5 5 7 4 9 7 4 5 7 4 3 - - - - 5 5 2 4
Quality
Consensus
TQGRMLRA-LRTAEVD--+EERGILDVVKKLGTSTKKWPK+TYHK
Pso_292791/1-147 79 VGLDGYVNIKSGALD---MEKTTSTFLTKSFNPDQVHKWLQL--- 117
Pso_288906/1-147 79 VGLDGYVNIKSGALD---MEKTTSTFLTKSFNPDQVHKWLQL--- 117
Prm_Prm_86463/1-146 87 L----YVHWKSSAMN---QDETAKLFLKKNIHPDKVKKWLGA--- 121
Pca_572048/1-160 87 T----IVRLKSQFAT---ADDAVQAFMTRNINPDAVYKWLKLYKA 124
Pso_288815/1-125 83 I----YVKSYQGLENSALIDQVAELSLRKSFNPNQVYKWLNL--- 120
Pso_356035/1-148 83 L----YVNFMSGMMD---IEKTANAFLKKNINPDKVFKWLKL--- 117
Pin_PITG_13093T0/1-154 82 A----NVKYQSPFIY---NDGIGQSIMKRNIDPDKVFKYLTQ--- 116
Pso_358111/1-148 83 L----YVNFMSGMMD---IEKTANAFLKKNINPDKVFKWLKL--- 117
Conservation
5 - - - - 5 * 5 4 5 8 5 6 3 5 - - - 5 9 3 5 6 4 5 4 9 6 9 9 8 4 * 8 4 * 6 * + * 4 5 - - -
Quality

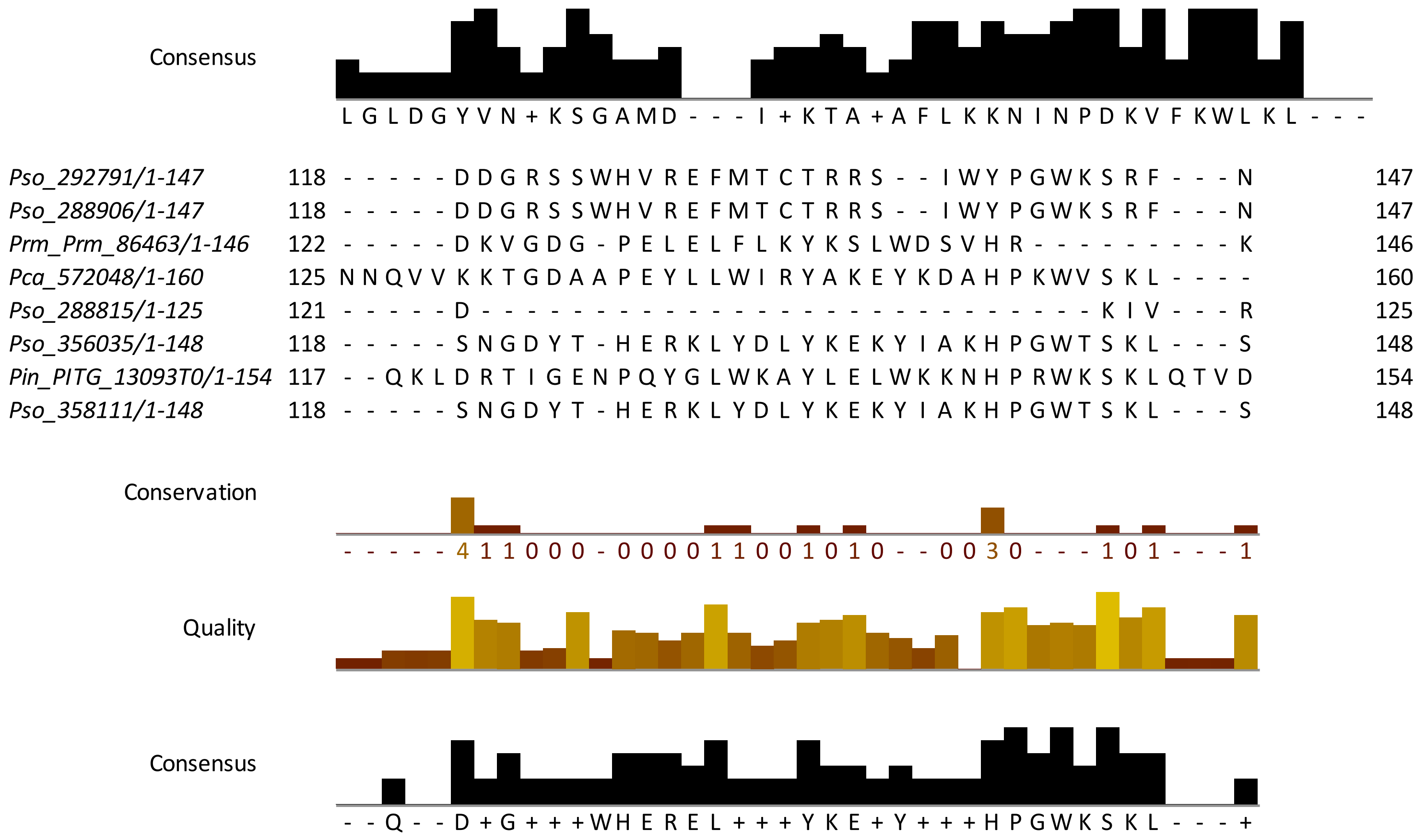

Consensus
L G L D G Y V N + K S G A M D - - - I + K T A + A F L K K N I N P D K V F K W L K L - - -
Pso_292791/1-147 118 - - - - - D D G R S S W H V R E F M T C T R R S - - I W Y P G W K S R F - - - N 147
Pso_288906/1-147 118 - - - - - D D G R S S W H V R E F M T C T R R S - - I W Y P G W K S R F - - - N 147
Prm_Prm_86463/1-146 122 - - - - - D K V G D G - P E L E L F L K Y K S L W D S V H R - - - - - - - - - K 146
Pca_572048/1-160 125 N N Q V V K K T G D A A P E Y L L W I R Y A K E Y K D A H P K W V S K L - - - - 160
Pso_288815/1-125 121 - - - - - D - - - - - - - - - - - - - - - - - - - - - - - - - - - K I V - - - R 125
Pso_356035/1-148 118 - - - - - S N G D Y T - H E R K L Y D L Y K E K Y I A K H P G W T S K L - - - S 148
Pin_PITG_13093T0/1-154 117 - - Q K L D R T I G E N P Q Y G L W K A Y L E L W K K N H P R W K S K L Q T V D 154
Pso_358111/1-148 118 - - - - - S N G D Y T - H E R K L Y D L Y K E K Y I A K H P G W T S K L - - - S 148
Conservation
- - - - - 4 1 1 0 0 0 - 0 0 0 0 1 1 0 0 1 0 1 0 - - 0 0 3 0 - - - 1 0 1 - - - 1
Quality
Consensus
- - Q - - D + G + + + W H E R E L + + + Y K E + Y + + + H P G W K S K L - - - +

Ortholog 9

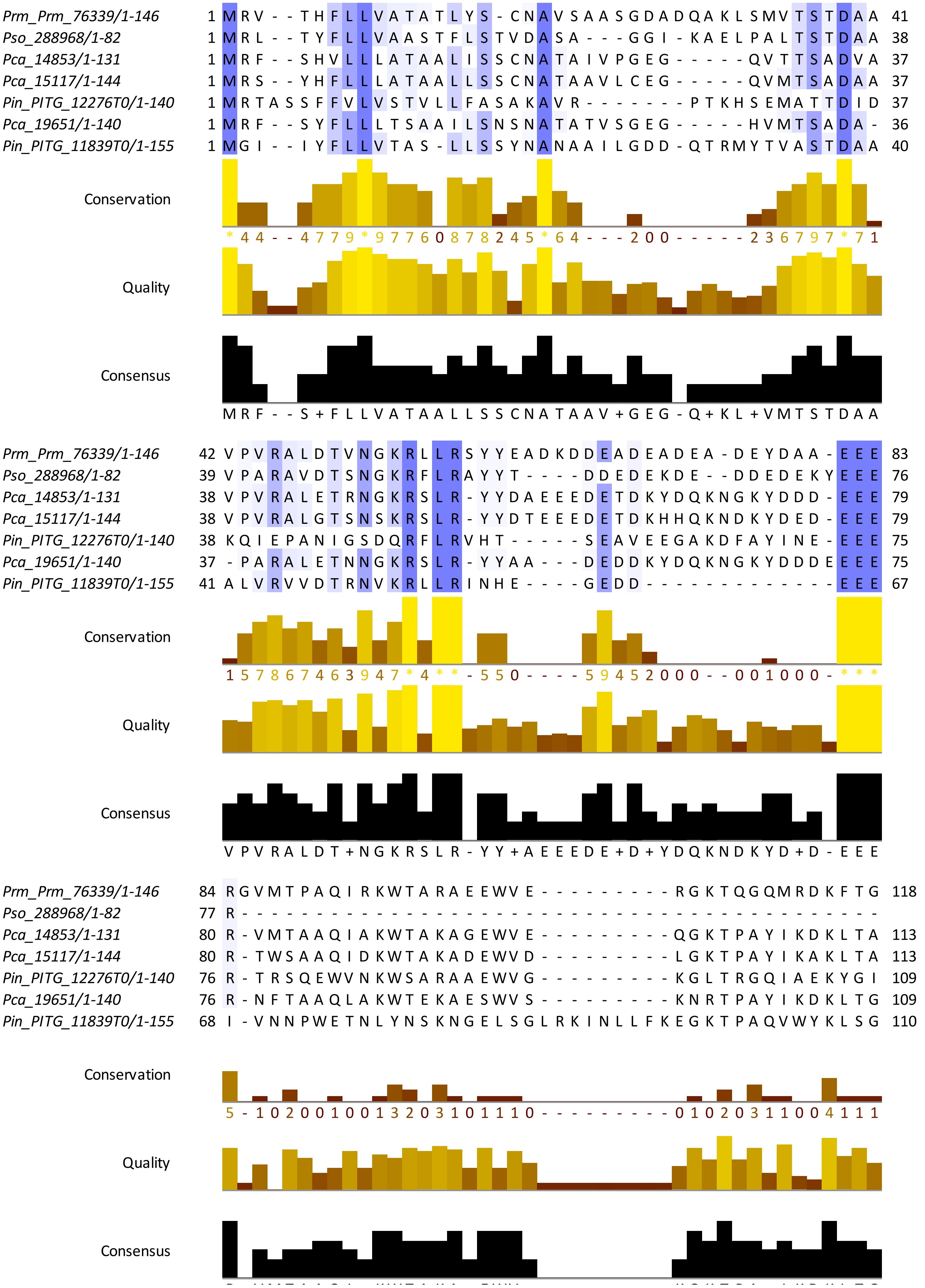
Prm_Prm_76339/1-146 1 M R V - - T H F L L V A T A T L Y S - C N A V S A A S G D A D Q A K L S M V T S T D A A 41
Pso_288968/1-82 1 M R L - - T Y F L L V A A S T F L S T V D A S A - - - G G I - K A E L P A L T S T D A A 38
Pca_14853/1-131 1 M R F - - S H V L L L A T A A L I S S C N A T A I V P G E G - - - - - Q V T T S A D V A 37
Pca_15117/1-144 1 M R S - - Y H F L L L A T A A L L S S C N A T A A V L C E G - - - - - Q V M T S A D A A 37
Pin_PITG_12276T0/1-140 1 M R T A S S F F V L V S T V L L F A S A K A V R - - - - - - - - P T K H S E M A T T D I D 37
Pca_19651/1-140 1 M R F - - S Y F L L L T S A A I L S N S N A T A T V S G E G - - - - - H V M T S A D A - 36
Pin_PITG_11839T0/1-155 1 M G I - - I Y F L L V T A S - L L S S Y N A N A A I L G D D - Q T R M Y T V A S T D A A 40
Conservation
* 4 4 - - 4 7 7 9 * 9 7 7 6 0 8 7 8 2 4 5 * 6 4 - - - 2 0 0 - - - - - 2 3 6 7 9 7 * 7 1
Quality
Consensus
M R F - - S + F L L V A T A A L L S S C N A T A A V + G E G - Q + K L + V M T S T D A A
Prm_Prm_76339/1-146 42 V P V R A L D T V N G K R L L R S Y Y E A D K D D E A D E A D E A - D E Y D A A - E E E 83
Pso_288968/1-82 39 V P A R A V D T S N G K R F L R A Y Y T - - - - D D E D E K D E - - D D E D E K Y E E E 76
Pca_14853/1-131 38 V P V R A L E T R N G K R S L R - Y Y D A E E E D E T D K Y D Q K N G K Y D D D - E E E 79
Pca_15117/1-144 38 V P V R A L G T S N S K R S L R - Y Y D T E E E D E T D K H H Q K N D K Y D E D - E E E 79
Pin_PITG_12276T0/1-140 38 K Q I E P A N I G S D Q R F L R V H T - - - - - S E A V E E G A K D F A Y I N E - E E E 75
Pca_19651/1-140 37 - P A R A L E T N N G K R S L R - Y Y A A - - - D E D D K Y D Q K N G K Y D D D E E E E 75
Pin_PITG_11839T0/1-155 41 A L V R V V D T R N V K R L L R I N H E - - - - G E D D - - - - - - - - - - - - - - E E E 67
Conservation
1 5 7 8 6 7 4 6 3 9 4 7 * 4 * * - 5 5 0 - - - - 5 9 4 5 2 0 0 0 - - 0 0 1 0 0 0 - * * *
Quality
Consensus
V P V R A L D T + N G K R S L R - Y Y + A E E E D E + D + Y D Q K N D K Y D + D - E E E
Prm_Prm_76339/1-146 84 R G V M T P A Q I R K W T A R A E E W V E - - - - - - - - - R G K T Q G Q M R D K F T G 118
Pso_288968/1-82 77 R - - - - - - - - - - - - - - - - - - - - - - - - - - - - - - - - - - - - - - - - - -
Pca_14853/1-131 80 R - V M T A A Q I A K W T A K A G E W V E - - - - - - - - - Q G K T P A Y I K D K L T A 113
Pca_15117/1-144 80 R - T W S A A Q I D K W T A K A D E W V D - - - - - - - - - L G K T P A Y I K A K L T A 113
Pin_PITG_12276T0/1-140 76 R - T R S Q E W V N K W S A R A A E W V G - - - - - - - - - K G L T R G Q I A E K Y G I 109
Pca_19651/1-140 76 R - N F T A A Q L A K W T E K A E S W V S - - - - - - - - - K N R T P A Y I K D K L T G 109
Pin_PITG_11839T0/1-155 68 I - V N N P W E T N L Y N S K N G E L S G L R K I N L L F K E G K T P A Q V W Y K L S G 110
Conservation
5 - 1 0 2 0 0 1 0 0 1 3 2 0 3 1 0 1 1 1 0 - - - - - - - - - 0 1 0 2 0 3 1 1 0 0 4 1 1 1
Quality
Consensus

```
                              R - V M T A A Q I + K W T A K A + E W V + - - - - - - - - - K G K T P A + I K D K L T G

Prm_Prm_76339/1-146      119  L R G V M N - - - A K D K E K Y N L F L A A Y L R - - - - - - - - - - - - - - A H P - -  143
Pso_288968/1-82           78  - - - - - - - - - - - - - - - - - - - - - - - - - - - - - - - - - - - - - - A N P - -   80
Pca_14853/1-131          114  F N G V M S - - - D K N K E K I L S V - - - - - - - - - - - - - - - - - - - - - - - - -  129
Pca_15117/1-144          114  F N G V M S - - - D K N R K K Y E L F L A K W G R - - - - - - - - - - - - - - A N P D E  140
Pin_PITG_12276T0/1-140   110  K K G R R T D P R S K N K Q K Y N L F L A A S L K - - - - - - - - - - - - - - K H P - -  137
Pca_19651/1-140          110  M N G L M T - - - A E N R K K Y E L F T A A Y G R - - - - - - - - - - - - - - A N P H A  136
Pin_PITG_11839T0/1-155   111  L G A G M I - - - Q K N G M K F N L F F L Q S I T L C I P A T L A S Q D E V L A I S H S  151
```

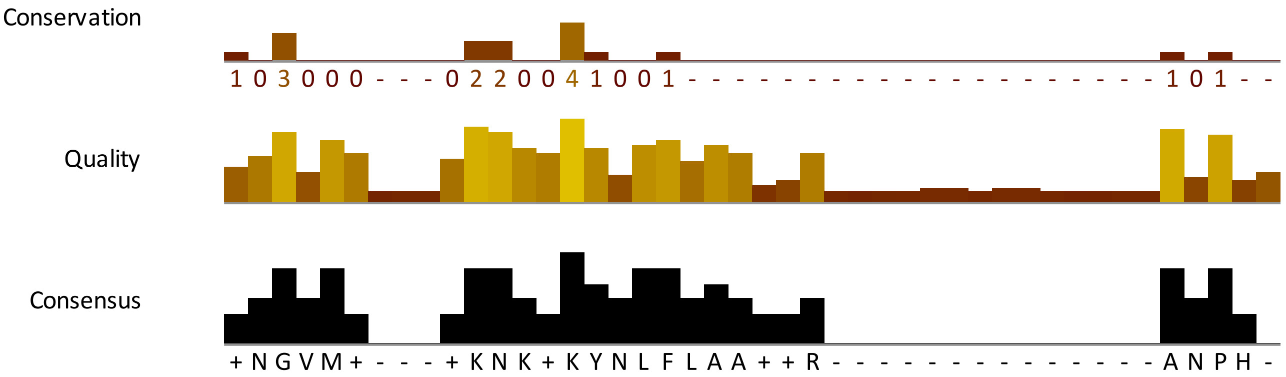


```
Prm_Prm_76339/1-146      144  - G G I    146
Pso_288968/1-82           81  - - D L     82
Pca_14853/1-131          130  - - R G    131
Pca_15117/1-144          141  F G R R    144
Pin_PITG_12276T0/1-140   138  - G G I    140
Pca_19651/1-140          137  L D R L    140
Pin_PITG_11839T0/1-155   152  L N I T    155
```

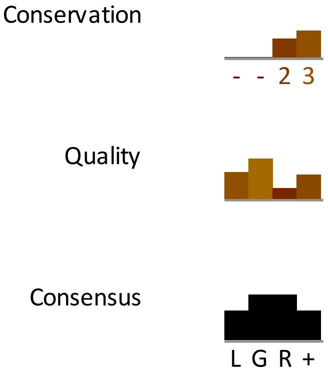

## Ortholog 10

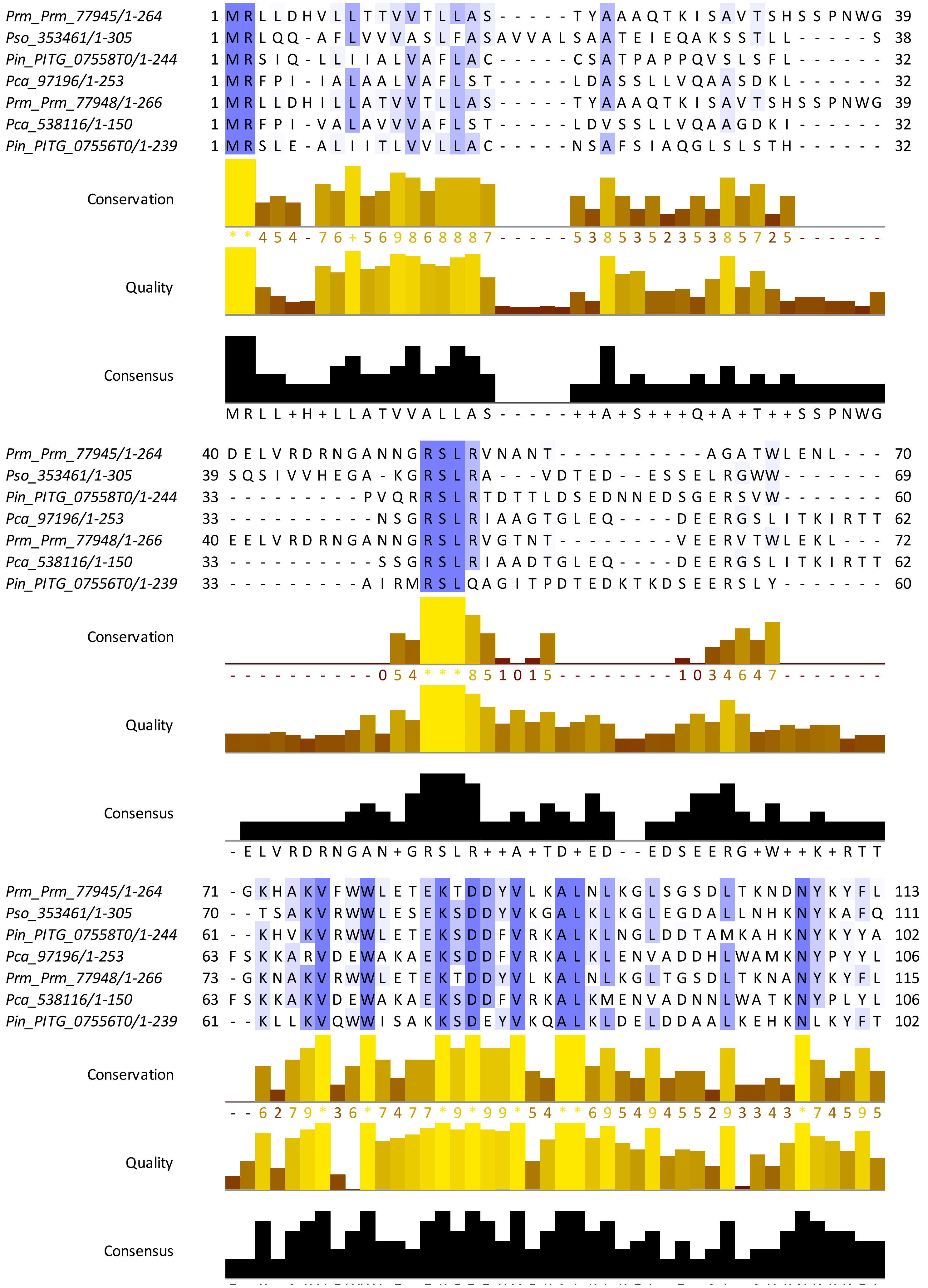
Prm_Prm_77945/1-264 1 MRLLDHVLLTTVVTLLAS-----TYAAAQTKISAVTSHSSPNWG 39
Pso_353461/1-305 1 MRLQQ-AFLVVVASLFASAVVALSAATEIEQAKSSTLL-----S 38
Pin_PITG_07558T0/1-244 1 MRSIQ-LLIIALVAFLAC-----CSATPAPPQVSLSFL------ 32
Pca_97196/1-253 1 MRFPI-IALAALVAFLST-----LDASSLLVQAASDKL------ 32
Prm_Prm_77948/1-266 1 MRLLDHILLATVVTLLAS-----TYAAAQTKISAVTSHSSPNWG 39
Pca_538116/1-150 1 MRFPI-VALAVVVAFLST-----LDVSSLLVQAAGDKI------ 32
Pin_PITG_07556T0/1-239 1 MRSLE-ALIITLVVLLAC-----NSAFSIAQGLSLSTH------ 32
Conservation
**454-76+5698688 87-----538535235385725------
Quality
Consensus
MRLL+H+LLATVVALLAS-----++A+S+++Q+A+T++SSPNWG
Prm_Prm_77945/1-264 40 DELVRDRNGANNGRSLRVNANT----------AGATWLENL--- 70
Pso_353461/1-305 39 SQSIVVHEGA-KGRSLRA---VDTED--ESSELRGWW------- 69
Pin_PITG_07558T0/1-244 33 ----------PVQRRSLRTDTTLDSEDNNEDSGERSVW------- 60
Pca_97196/1-253 33 -----------NSGRSLRIAAGTGLEQ---DEERGSLITKIRTT 62
Prm_Prm_77948/1-266 40 EELVRDRNGANNGRSLRVGTNT--------VEERVTWLEKL--- 72
Pca_538116/1-150 33 ------------SSGRSLRIAADTGLEQ---DEERGSLITKIRTT 62
Pin_PITG_07556T0/1-239 33 ----------AIRMRSLQAGITPDTEDKTKDSEERSLY------- 60
Conservation
-----------054***851015--------1034647-------
Quality
Consensus
-ELVRDRNGAN+GRSLR++A+TD+ED--EDSEERG+W++K+RTT
Prm_Prm_77945/1-264 71 -GKHAKVFWWLETEKTDDYVLKALNLKGLSGSDLTKNDNYKYFL 113
Pso_353461/1-305 70 --TSAKVRWWLESEKSDDYVKGALKLKGLEGDALLNHHKNYKAFQ 111
Pin_PITG_07558T0/1-244 61 --KHVKVRWWLETEKSDDFVRKALKLNGLDDTAMKAHKNYKYYA 102
Pca_97196/1-253 63 FSKKARVDEWAKAEKSDDFVRKALKLENVADDHLWAMKNYPYYL 106
Prm_Prm_77948/1-266 73 -GKNAKVRWWLETEKTDDYVLKALNLKGLTGSDLTKNANYKYFL 115
Pca_538116/1-150 63 FSKKAKVDEWAKAEKSDDFVRKALKMENVADNNLWATKNYPLYL 106
Pin_PITG_07556T0/1-239 61 --KLLKVQWWISAKKSDEYVKQALKLDELDDAALKEHKNLKYFT 102
Conservation
--6279*36*7477*9*99*54**69549455293343*74595
Quality
Consensus

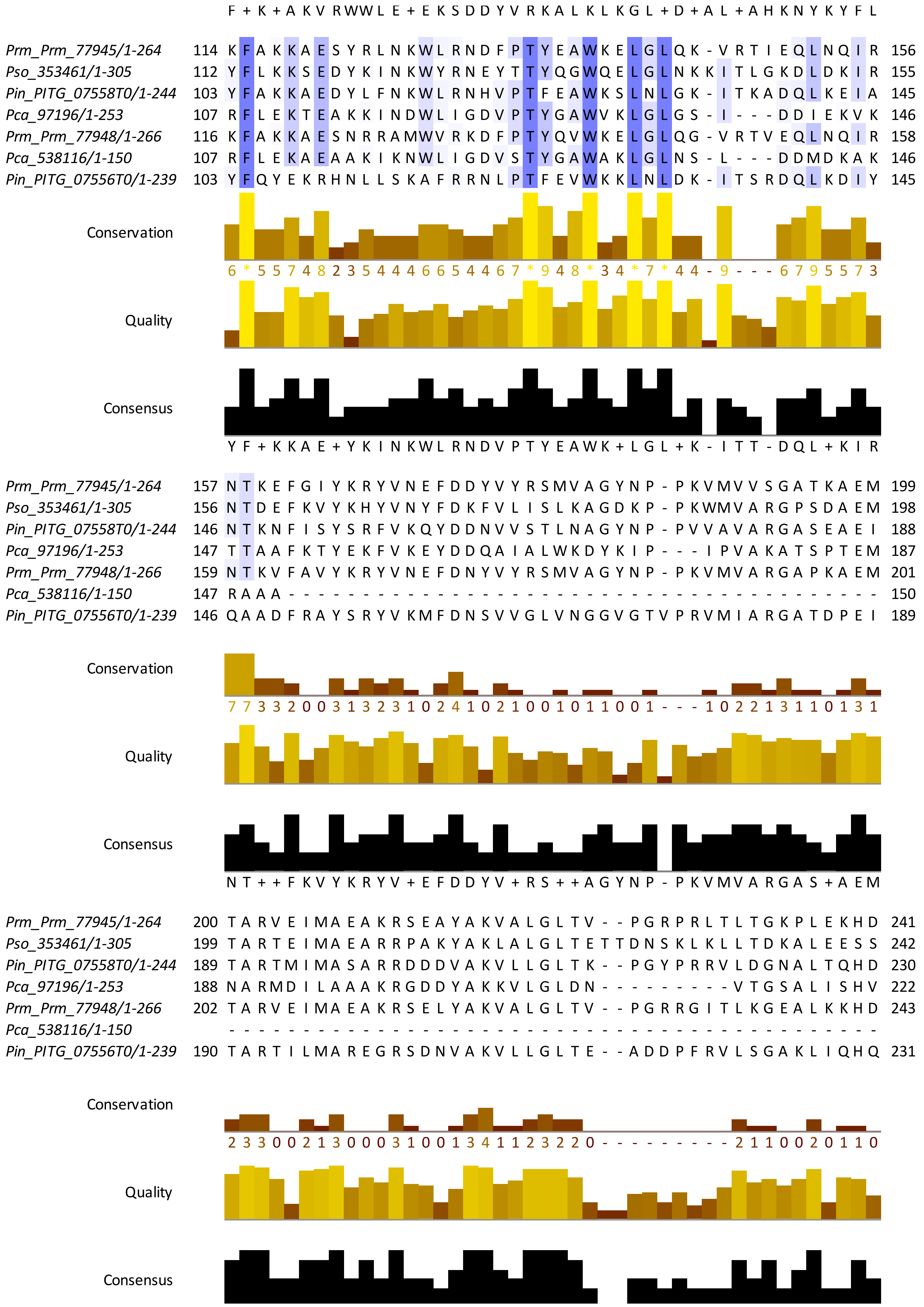
F + K + A K V R W W L E + E K S D D Y V R K A L K L K G L + D + A L + A H K N Y K Y F L
Prm_Prm_77945/1-264 114 K F A K K A E S Y R L N K W L R N D F P T Y E A W K E L G L Q K - V R T I E Q L N Q I R 156
Pso_353461/1-305 112 Y F L K K S E D Y K I N K W Y R N E Y T T Y Q G W Q E L G L N K K I T L G K D L D K I R 155
Pin_PITG_07558T0/1-244 103 Y F A K K A E D Y L F N K W L R N H V P T F E A W K S L N L G K - I T K A D Q L K E I A 145
Pca_97196/1-253 107 R F L E K T E A K K I N D W L I G D V P T Y G A W V K L G L G S - I - - - D D I E K V K 146
Prm_Prm_77948/1-266 116 K F A K K A E S N R R A M W V R K D F P T Y Q V W K E L G L Q G - V R T V E Q L N Q I R 158
Pca_538116/1-150 107 R F L E K A E A A K I K N W L I G D V S T Y G A W A K L G L N S - L - - - D D M D K A K 146
Pin_PITG_07556T0/1-239 103 Y F Q Y E K R H N L L S K A F R R N L P T F E V W K K L N L D K - I T S R D Q L K D I Y 145
Conservation
6 * 5 5 7 4 8 2 3 5 4 4 4 6 6 5 4 4 6 7 * 9 4 8 * 3 4 * 7 * 4 4 - 9 - - - 6 7 9 5 5 7 3
Quality
Consensus
Y F + K K A E + Y K I N K W L R N D V P T Y E A W K + L G L + K - I T T - D Q L + K I R
Prm_Prm_77945/1-264 157 N T K E F G I Y K R Y V N E F D D Y V Y R S M V A G Y N P - P K V M V V S G A T K A E M 199
Pso_353461/1-305 156 N T D E F K V Y K H Y V N Y F D K F V L I S L K A G D K P - P K W M V A R G P S D A E M 198
Pin_PITG_07558T0/1-244 146 N T K N F I S Y S R F V K Q Y D D N V V S T L N A G Y N P - P V V A V A R G A S E A E I 188
Pca_97196/1-253 147 T T A A F K T Y E K F V K E Y D D Q A I A L W K D Y K I P - - - I P V A K A T S P T E M 187
Prm_Prm_77948/1-266 159 N T K V F A V Y K R Y V N E F D N Y V Y R S M V A G Y N P - P K V M V A R G A P K A E M 201
Pca_538116/1-150 147 R A A A - - - - - - - - - - - - - - - - - - - - - - - - - - - - - - - - - - - - - - - - 150
Pin_PITG_07556T0/1-239 146 Q A A D F R A Y S R Y V K M F D N S V V G L V N G G V G T V P R V M I A R G A T D P E I 189
Conservation
7 7 3 3 2 0 0 3 1 3 2 3 1 0 2 4 1 0 2 1 0 0 1 0 1 1 0 0 1 - - - 1 0 2 2 1 3 1 1 0 1 3 1
Quality
Consensus
N T + + F K V Y K R Y V + E F D D Y V + R S + + A G Y N P - P K V M V A R G A S + A E M
Prm_Prm_77945/1-264 200 T A R V E I M A E A K R S E A Y A K V A L G L T V - - P G R P R L T L T G K P L E K H D 241
Pso_353461/1-305 199 T A R T E I M A E A R R P A K Y A K L A L G L T E T T D N S K L K L L T D K A L E E S S 242
Pin_PITG_07558T0/1-244 189 T A R T M I M A S A R R D D D V A K V L L G L T K - - P G Y P R R V L D G N A L T Q H D 230
Pca_97196/1-253 188 N A R M D I L A A A K R G D D Y A K K V L G L D N - - - - - - - - - V T G S A L I S H V 222
Prm_Prm_77948/1-266 202 T A R V E I M A E A K R S E L Y A K V A L G L T V - - P G R R G I T L K G E A L K K H D 243
Pca_538116/1-150 - - - - - - - - - - - - - - - - - - - - - - - - - - - - - - - - - - - - - - - - - - -
Pin_PITG_07556T0/1-239 190 T A R T I L M A R E G R S D N V A K V L L G L T E - - A D D P F R V L S G A K L I Q H Q 231
Conservation
2 3 3 0 0 2 1 3 0 0 0 3 1 0 0 1 3 4 1 1 2 3 2 2 0 - - - - - - - - - 2 1 1 0 0 2 0 1 1 0
Quality
Consensus

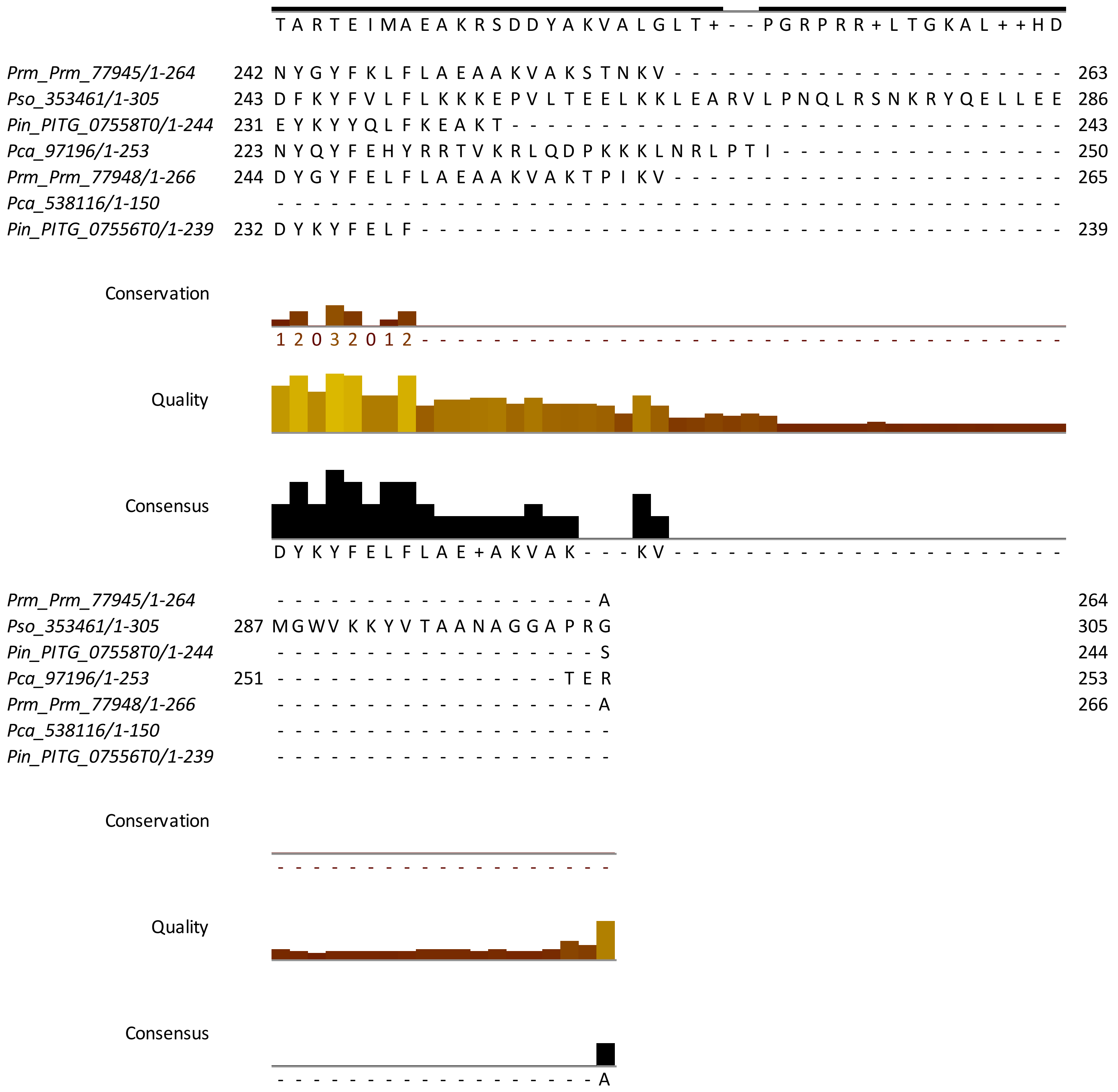

T A R T E I M A E A K R S D D Y A K V A L G L T + - - P G R P R R + L T G K A L + + H D
Prm_Prm_77945/1-264 242 N Y G Y F K L F L A E A A K V A K S T N K V - - - - - - - - - - - - - - - - - - - - - - 263
Pso_353461/1-305 243 D F K Y F V L F L K K K E P V L T E E L K K L E A R V L P N Q L R S N K R Y Q E L L E E 286
Pin_PITG_07558T0/1-244 231 E Y K Y Y Q L F K E A K T - - - - - - - - - - - - - - - - - - - - - - - - - - - - - 243
Pca_97196/1-253 223 N Y Q Y F E H Y R R T V K R L Q D P K K K L N R L P T I - - - - - - - - - - - - - - - - 250
Prm_Prm_77948/1-266 244 D Y G Y F E L F L A E A A K V A K T P I K V - - - - - - - - - - - - - - - - - - - - - - 265
Pca_538116/1-150 - - - - - - - - - - - - - - - - - - - - - - - - - - - - - - - - - - - - - - - - - - - -
Pin_PITG_07556T0/1-239 232 D Y K Y F E L F - - - - - - - - - - - - - - - - - - - - - - - - - - - - - - - - - - - - 239
Conservation
1 2 0 3 2 0 1 2 - - - - - - - - - - - - - - - - - - - - - - - - - - - - - - - - - - - -
Quality
Consensus
D Y K Y F E L F L A E + A K V A K - - - K V - - - - - - - - - - - - - - - - - - - - - -
Prm_Prm_77945/1-264 - - - - - - - - - - - - - - - - - - A 264
Pso_353461/1-305 287 M G W V K K Y V T A A N A G G A P R G 305
Pin_PITG_07558T0/1-244 - - - - - - - - - - - - - - - - - - S 244
Pca_97196/1-253 251 - - - - - - - - - - - - - - - - T E R 253
Prm_Prm_77948/1-266 - - - - - - - - - - - - - - - - - - A 266
Pca_538116/1-150 - - - - - - - - - - - - - - - - - - -
Pin_PITG_07556T0/1-239 - - - - - - - - - - - - - - - - - - -
Conservation
- - - - - - - - - - - - - - - - - - -
Quality
Consensus
- - - - - - - - - - - - - - - - - - A

Ortholog 11

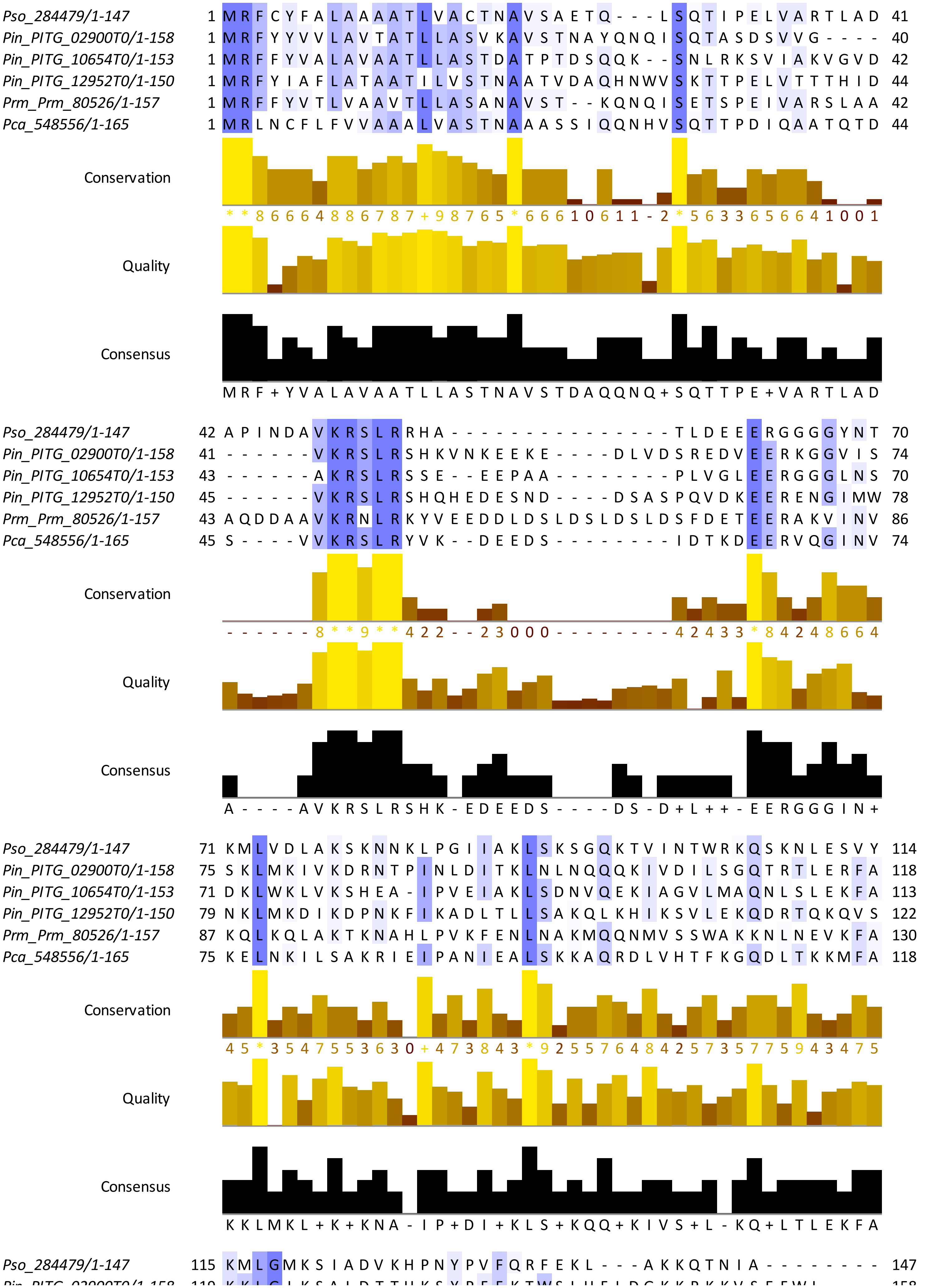
Pso_284479/1-147 1 MRFCYFALAAAATLVACTNAVSAETQ---LSQTIPELVARTLAD 41
Pin_PITG_02900T0/1-158 1 MRFYYVVLAVTATLLASVKAVSTNAYQNQISQTASDSVVG---- 40
Pin_PITG_10654T0/1-153 1 MRFFYVALAVAATLLASTDATPTDSQQK--SNLRKSVIAKVGVD 42
Pin_PITG_12952T0/1-150 1 MRFYIAFLATAATILVSTNAATVDAQHNWVSKTTPELVTTTHID 44
Prm_Prm_80526/1-157 1 MRFFYVTLVAAVTLLASANAVST--KQNQISETSPEIVARSLAA 42
Pca_548556/1-165 1 MRLNCFLFVVAAALVASTNAAASSIQQNHVSQTTPDIQAATQTD 44
Conservation
Quality
Consensus
MRF+YVALAVAATLLASTNAVSTDAQQNQ+SQTTPE+VARTLAD
Pso_284479/1-147 42 APINDAVKRSLRRHA----------------TLDEEERGGGGYNT 70
Pin_PITG_02900T0/1-158 41 ------VKRSLRSHKVNKEEKE----DLVDSREDVEERKGGVIS 74
Pin_PITG_10654T0/1-153 43 ------AKRSLRSSE--EEPAA--------PLVGLEERGGGLNS 70
Pin_PITG_12952T0/1-150 45 ------VKRSLRSHQHEDESND----DSASPQVDKEERENGIMW 78
Prm_Prm_80526/1-157 43 AQDDAAVKRNLRKYVEEDDLDSLDSLDSLDSFDETEERAKVINV 86
Pca_548556/1-165 45 S----VVKRSLRYVK--DEEDS---------IDTKDEERVQGINV 74
Conservation
Quality
Consensus
A----AVKRSLRSHK-EDEEDS----DS-D+L++-EERGGGIN+
Pso_284479/1-147 71 KMLVDLAKSKNNKLPGIIAKLSKSGQKTVINTWRKQSKNLESVY 114
Pin_PITG_02900T0/1-158 75 SKLMKIVKDRNTPINLDITKLNLNQQQKIVDILSGQTRTLERFA 118
Pin_PITG_10654T0/1-153 71 DKLWKLVKSHEA-IPVEIAKLSDNVQEKIAGVLMAQNLSLEKFA 113
Pin_PITG_12952T0/1-150 79 NKLMKDIKDPNKFIKADLTLLSAKQLKHIKSVLEKQDRTQKQVS 122
Prm_Prm_80526/1-157 87 KQLKQLAKTKNAHLPVKFENLNAKMQQNMVSSWAKKNLNEVKFA 130
Pca_548556/1-165 75 KELNKILSAKRIEIPANIEALSKKAQRDLVHTFKGQDLTKKMFA 118
Conservation
Quality
Consensus
KKLMKL+K+KNA-IP+DI+KLS+KQQ+KIVS+L-KQ+LTLEKFA
Pso_284479/1-147 115 KMLGMKSIADVKHPNYPVFQRFEKL---AKKQTNIA-------- 147

```
Pin_PITG_02900T0/1-158   119 KKLGTKSALDTTHKSYPFFKTWSLHFLDGKKPKKVSEFWI----  158
Pin_PITG_10654T0/1-153   114 KKVGMDGAHDTAHRNNPFFQKWKANFLDGKKPKKIPEEWI----  153
Pin_PITG_12952T0/1-150   123 KKLG--------NQNSKFVEEWKESFRVRKKPKPQW--------  150
Prm_Prm_80526/1-157      131 KKLGLKSVNDVGNKNYPAFQKFREMFT-----------------  157
Pca_548556/1-165         119 TKLGMRDVDDVANRHYPFFEKWEHLFRPGKKEKKVPEMLIGNHE 162
```

Conservation

Quality

Consensus

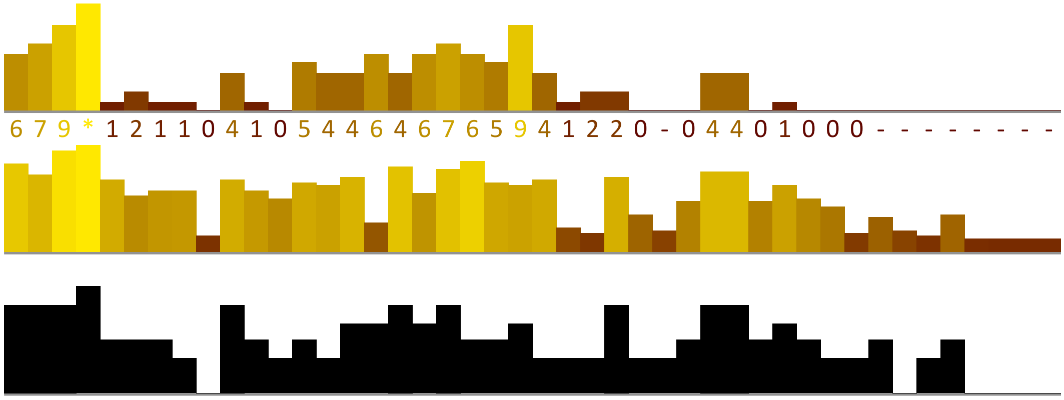

```
Conservation: 679*1211041054464676594122 0-0440 1000--------
Consensus:    KKLGMKS+-DVA++NYPFFQKW+ELF+DGKKPKK+PE-WI----
```

```
Pso_284479/1-147             ---
Pin_PITG_02900T0/1-158       ---
Pin_PITG_10654T0/1-153       ---
Pin_PITG_12952T0/1-150       ---
Prm_Prm_80526/1-157          ---
Pca_548556/1-165         163 YYY  165
```

Conservation

Quality

Consensus

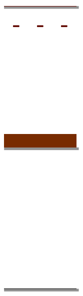

```
Conservation: ---
Consensus:    ---
```

Ortholog 12

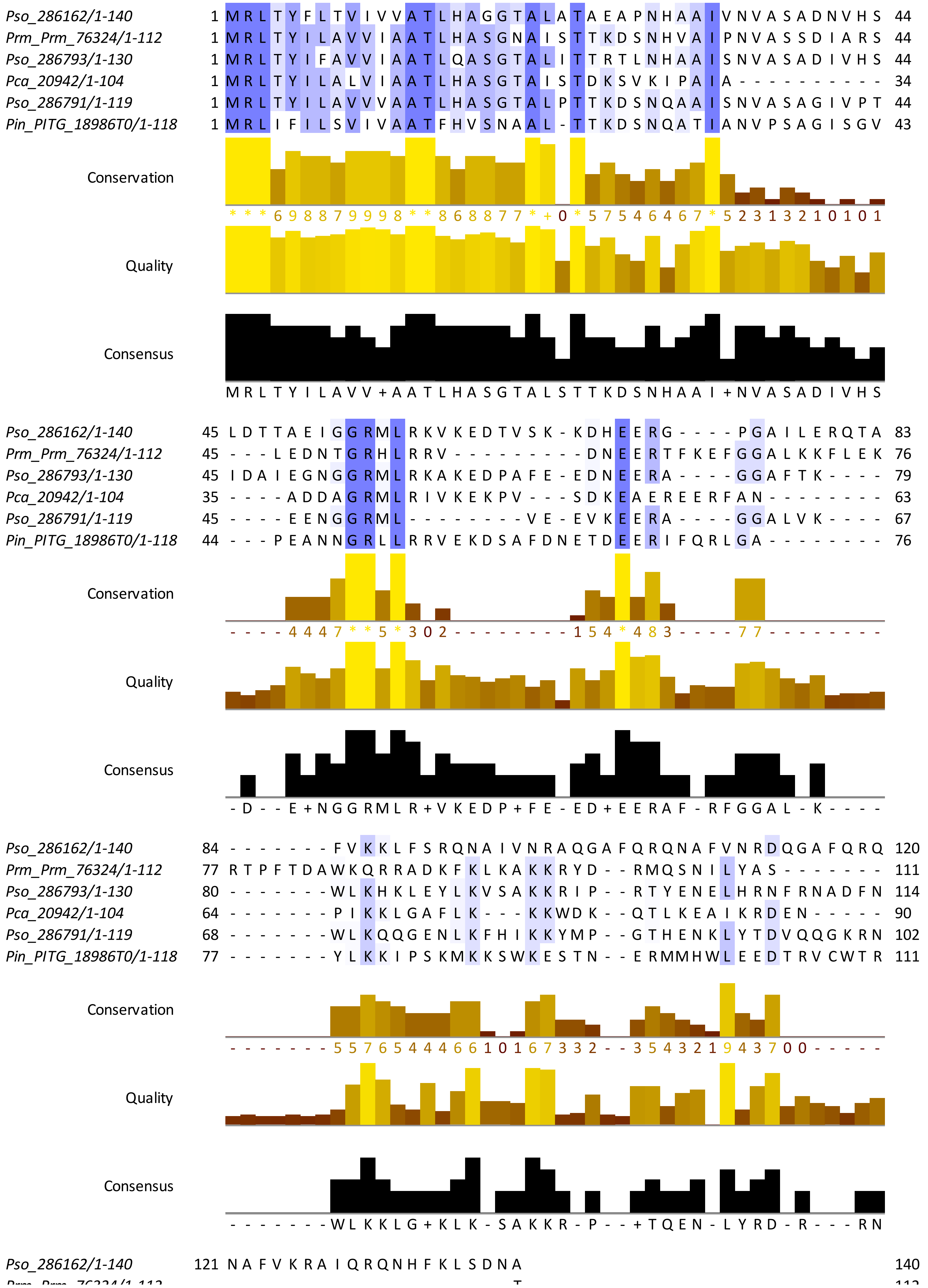
Pso_286162/1-140 1 MRLTYFLTVIVVATLHAGGTALATAEAPNHAAIVNVASADNVHS 44
Prm_Prm_76324/1-112 1 MRLTYILAVVIAATLHASGNAISTTKDSNHVAIPNVASSDIARS 44
Pso_286793/1-130 1 MRLTYIFAVVIAATLQASGTALITTRTLNHAAISNVASADIVHS 44
Pca_20942/1-104 1 MRLTYILALVIAATLHASGTAISTDKSVKIPAIA---------- 34
Pso_286791/1-119 1 MRLTYILAVVVAATLHASGTALPTTKDSNQAAISNVASAGIVPT 44
Pin_PITG_18986T0/1-118 1 MRLIFILSVIVAATFHVSNAAL-TTKDSNQATIANVPSAGISGV 43
Conservation
***69887999 8**868877*+0*57546467*52313210101
Quality
Consensus
MRLTYILAVV+AATLHASGTALSTTKDSNHAAI+NVASADIVHS
Pso_286162/1-140 45 LDTTAEIGGRMLRKVKEDTVSK-KDHEERG----PGAILERQTA 83
Prm_Prm_76324/1-112 45 ---LEDNTGRHLRRV---------DNEERTFKEFGGALKKFLEK 76
Pso_286793/1-130 45 IDAIEGNGGRMLRKAKEDPAFE-EDNEERA----GGAFTK---- 79
Pca_20942/1-104 35 ----ADDAGRMLRIVKEKPV---SDKEAEREERFAN-------- 63
Pso_286791/1-119 45 ----EENGGRML--------VE-EVKEERA----GGALVK---- 67
Pin_PITG_18986T0/1-118 44 ---PEANNGRLLRRVEKDSAFDNETDEERIFQRLGA-------- 76
Conservation
----4447**5*302---------154*483----77--------
Quality
Consensus
-D--E+NGGRMLR+VKEDP+FE-ED+EERAF-RFGGAL-K----
Pso_286162/1-140 84 -------FVKKLFSRQNAIVNRAQGAFQRQNAFVNRDQGAFQRQ 120
Prm_Prm_76324/1-112 77 RTPFTDAWKQRRADKFKLKAKKRYD--RMQSNILYAS------- 111
Pso_286793/1-130 80 -------WLKHKLEYLKVSAKKRIP--RTYENELHRNFRNADFN 114
Pca_20942/1-104 64 -------PIKKLGAFLK---KKWDK--QTLKEAIKRDEN----- 90
Pso_286791/1-119 68 -------WLKQQGENLKFHIKKYMP--GTHENKLYTDVQQGKRN 102
Pin_PITG_18986T0/1-118 77 -------YLKKIPSKMKKSWKESTN--ERMMHWLEEDTRVCWTR 111
Conservation
-------55765444661016733 2--354321943700-----
Quality
Consensus
-------WLKKLG+KLK-SAKKR-P--+TQEN-LYRD-R---RN
Pso_286162/1-140 121 NAFVKRAIQRQNHFKLSDNA 140

```
Prm_Prm_76324/1-112           - - - - - - - - - - - - - - - - - - - T    112
Pso_286793/1-130         115  G R F K R N - - - Q A A N I R A S I - N    130
Pca_20942/1-104           91  - - - - R R K W I R E Q G F E P R - - D    104
Pso_286791/1-119         103  A E M L K R - - - R D S G I R A A M G N    119
Pin_PITG_18986T0/1-118   112  G L I L D T - - - - - - - - - - - - - L    118
```

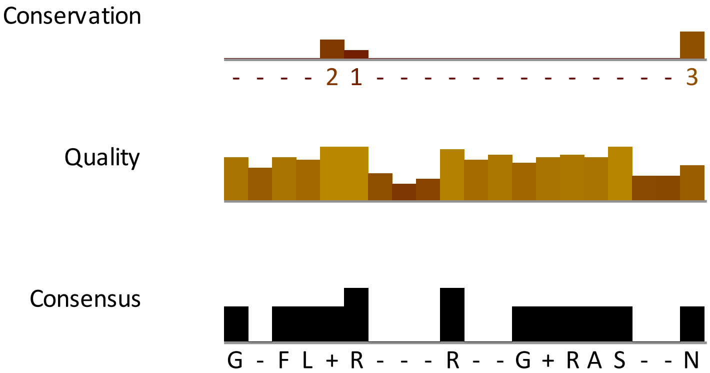

Ortholog 13

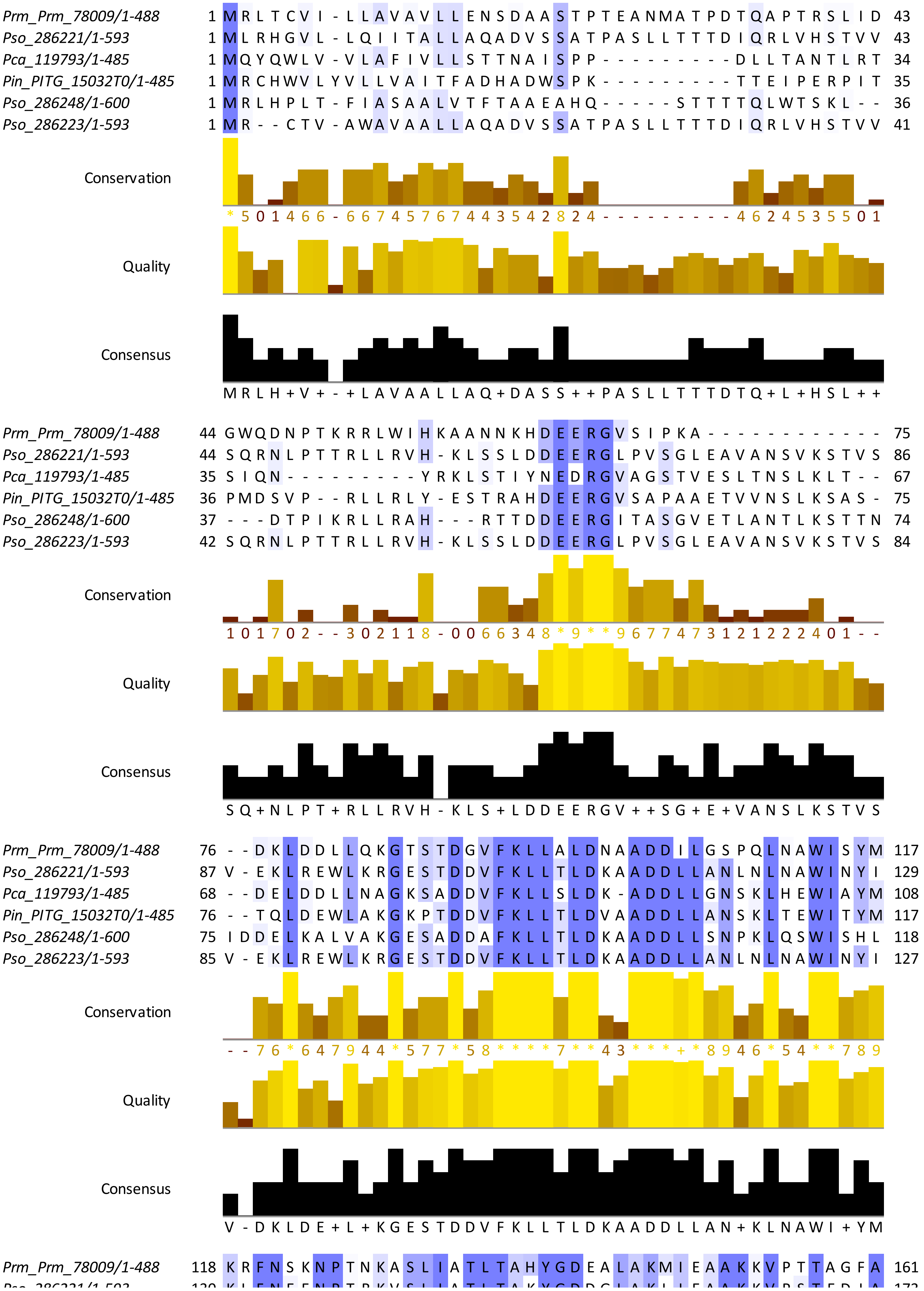
Prm_Prm_78009/1-488 1 MRLTCVI-LLAVAVLLENSDAASTPTEANMATPDTQAPTRSLID 43
Pso_286221/1-593 1 MLRHGVL-LQIITALLAQADVSSATPASLLTTTDIQRLVHSTVV 43
Pca_119793/1-485 1 MQYQWLV-VLAFIVLLSTTNAISPP---------DLLTANTLRT 34
Pin_PITG_15032T0/1-485 1 MRCHWVLYVLLVAITFADHADWSPK---------TTEIPERPIT 35
Pso_286248/1-600 1 MRLHPLT-FIASAALVTFTAAEAHQ----STTTTQLWTSKL-- 36
Pso_286223/1-593 1 MR--CTV-AWAVAALLAQADVSSATPASLLTTTDIQRLVHSTVV 41
Conservation
* 5 0 1 4 6 6 - 6 6 7 4 5 7 6 7 4 4 3 5 4 2 8 2 4 - - - - - - - - - 4 6 2 4 5 3 5 5 0 1
Quality
Consensus
MRLH+V+-+LAVAALLAQ+DASS++PASLLTTTDTQ+L+HSL++
Prm_Prm_78009/1-488 44 GWQDNPTKRRLWIHKAANNKHDEERGVSIPKA------------ 75
Pso_286221/1-593 44 SQRNLPTTRLLRVH-KLSSLDDEERGLPVSGLEAVANSVKSTVS 86
Pca_119793/1-485 35 SIQN---------YRKLSTIYNEDRGVAGSTVESLTNSLKLT-- 67
Pin_PITG_15032T0/1-485 36 PMDSVP--RLLRLY-ESTRAHDEERGVSAPAAETVVNSLKSAS- 75
Pso_286248/1-600 37 ---DTPIKRLLRAH---RTTDDEERGITASGVETLANTLKSTTN 74
Pso_286223/1-593 42 SQRNLPTTRLLRVH-KLSSLDDEERGLPVSGLEAVANSVKSTVS 84
Conservation
1 0 1 7 0 2 - - 3 0 2 1 1 8 - 0 0 6 6 3 4 8 * 9 * * 9 6 7 7 4 7 3 1 2 1 2 2 2 4 0 1 - -
Quality
Consensus
SQ+NLPT+RLLRVH-KLS+LDDEERGV++SG+E+VANSLKSTVS
Prm_Prm_78009/1-488 76 --DKLDDLLQKGTSTDGVFKLLALDNAADDILGSPQLNAWISYM 117
Pso_286221/1-593 87 V-EKLREWLKRGESTDDVFKLLTLDKAADDLLANLNLNAWINYI 129
Pca_119793/1-485 68 --DELDDLLNAGKSADDVFKLLSLDK-ADDLLGNSKLHEWIAYM 108
Pin_PITG_15032T0/1-485 76 --TQLDEWLAKGKPTDDVFKLLTLDVAADDLLANSKLTEWITYM 117
Pso_286248/1-600 75 IDDELKALVAKGESADDAFKLLTLDKAADDLLSNPKLQSWISHL 118
Pso_286223/1-593 85 V-EKLREWLKRGESTDDVFKLLTLDKAADDLLANLNLNAWINYI 127
Conservation
- - 7 6 * 6 4 7 9 4 4 * 5 7 7 * 5 8 * * * * 7 * * 4 3 * * * + * 8 9 4 6 * 5 4 * * 7 8 9
Quality
Consensus
V-DKLDE+L+KGESTDDVFKLLTLDKAADDLLAN+KLNAWI+YM
Prm_Prm_78009/1-488 118 KRFNSKNPTNKASLIATLTAHYGDEALAKMIEAAKKVPTTAGFA 161

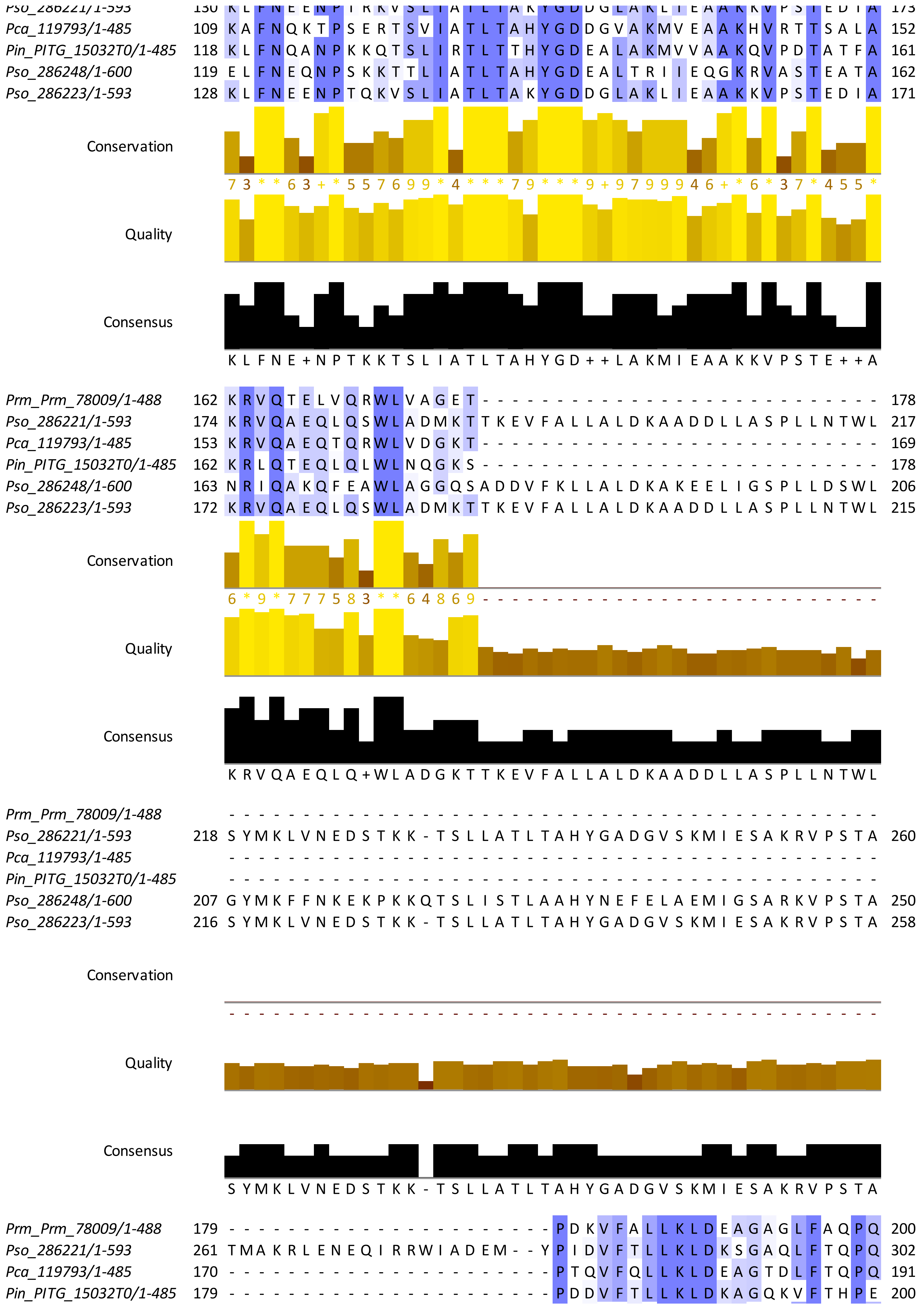

Pso_286221/1-593 130 KLFNEENPTRKVSLIATLTAKYGDDGLAKLIEAAKKVPSTEDIA 173
Pca_119793/1-485 109 KAFNQKTPSERTSVIATLTAHYGDDGVAKMVEAAKHVRTTSALA 152
Pin_PITG_15032T0/1-485 118 KLFNQANPKKQTSLIRTLTTHYGDEALAKMVVAAKQVPDTATFA 161
Pso_286248/1-600 119 ELFNEQNPSKKTTLIATLTAHYGDEALTRIIEQGKRVASTEATA 162
Pso_286223/1-593 128 KLFNEENPTQKVSLIATLTAKYGDDGLAKLIEAAKKVPSTEDIA 171
Conservation
7 3 * * 6 3 + * 5 5 7 6 9 9 * 4 * * * 7 9 * * * 9 + 9 7 9 9 9 4 6 + * 6 * 3 7 * 4 5 5 *
Quality
Consensus
KLFNE+NPTKKTSLIATLTAHYGD++LAKMIEAAKKVPSTE++A
Prm_Prm_78009/1-488 162 KRVQTELVQRWLVAGET--------------------------- 178
Pso_286221/1-593 174 KRVQAEQLQSWLADMKTTKEVFALLALDKAADDLLASPLLNTWL 217
Pca_119793/1-485 153 KRVQAEQTQRWLVDGKT--------------------------- 169
Pin_PITG_15032T0/1-485 162 KRLQTEQLQLWLNQGKS--------------------------- 178
Pso_286248/1-600 163 NRIQAKQFEAWLAGGQSADDVFKLLALDKAKEELIGSPLLDSWL 206
Pso_286223/1-593 172 KRVQAEQLQSWLADMKTTKEVFALLALDKAADDLLASPLLNTWL 215
Conservation
6 * 9 * 7 7 7 5 8 3 * * 6 4 8 6 9 - - - - - - - - - - - - - - - - - - - - - - - - - - -
Quality
Consensus
KRVQAEQLQ+WLADGKTTKEVFALLALDKAADDLLASPLLNTWL
Prm_Prm_78009/1-488 --------------------------------------------
Pso_286221/1-593 218 SYMKLVNEDSTKK-TSLLATLTAHYGADGVSKMIESAKRVPSTA 260
Pca_119793/1-485 --------------------------------------------
Pin_PITG_15032T0/1-485 --------------------------------------------
Pso_286248/1-600 207 GYMKFFNKEKPKKQTSLISTLAAHYNEFELAEMIGSARKVPSTA 250
Pso_286223/1-593 216 SYMKLVNEDSTKK-TSLLATLTAHYGADGVSKMIESAKRVPSTA 258
Conservation
Quality
Consensus
SYMKLVNEDSTKK-TSLLATLTAHYGADGVSKMIESAKRVPSTA
Prm_Prm_78009/1-488 179 ----------------------PDKVFALLKLDEAGAGLFAQPQ 200
Pso_286221/1-593 261 TMAKRLENEQIRRWIADEM--YPIDVFTLLKLDKSGAQLFTQPQ 302
Pca_119793/1-485 170 ----------------------PTQVFQLLKLDEAGTDLFTQPQ 191
Pin_PITG_15032T0/1-485 179 ----------------------PDDVFTLLKLDKAGQKVFTHPE 200

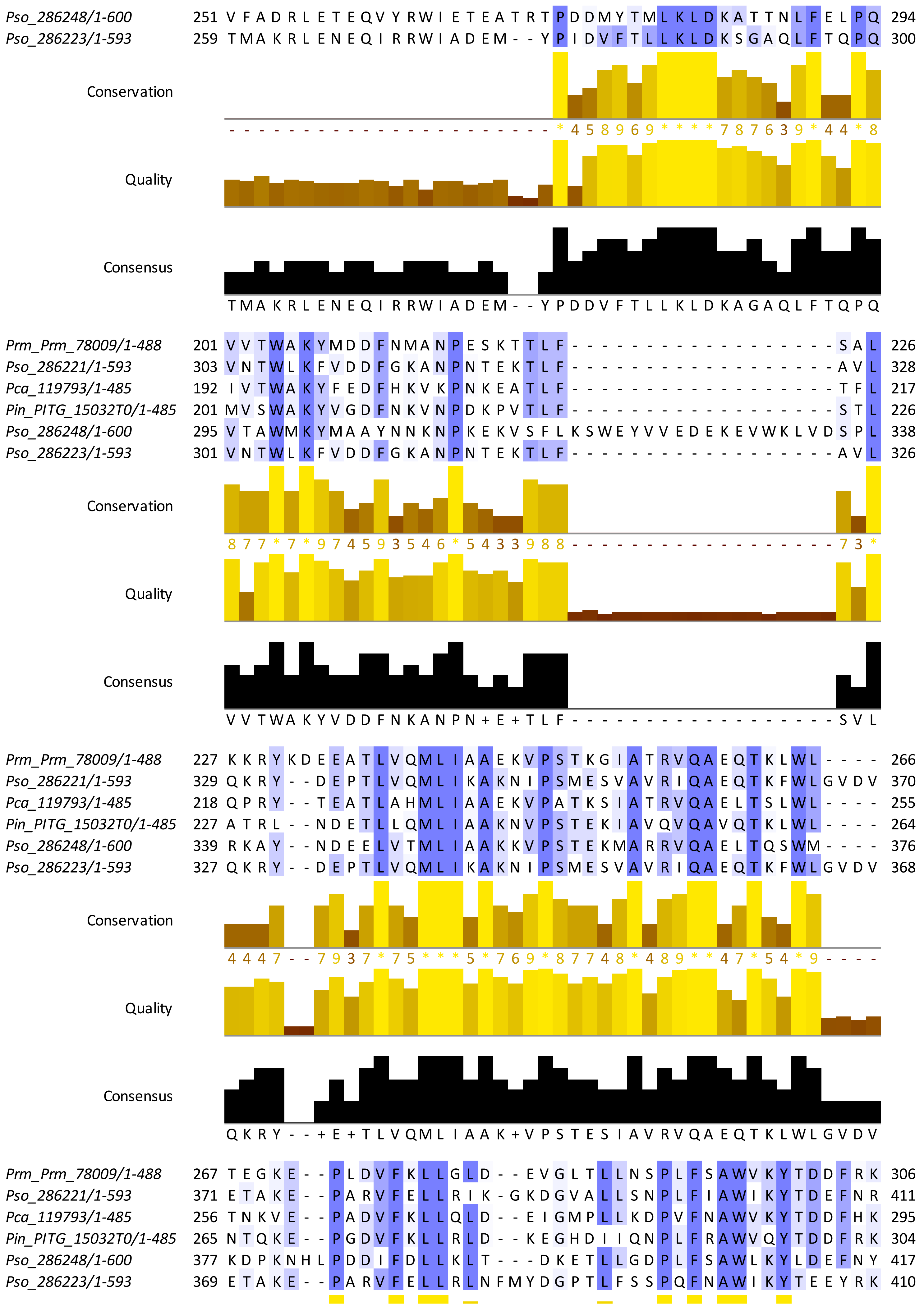
Pso_286248/1-600 251 VFADRLETEQVYRWIETEATRTPDDMYTMLKLDKATTNLFELPQ 294
Pso_286223/1-593 259 TMAKRLENEQIRRWIADEM--YPIDVFTLLKLDKSGAQLFTQPQ 300
Conservation
---------------------*458969****787639*44*8
Quality
Consensus
TMAKRLENEQIRRWIADEM--YPDDVFTLLKLDKAGAQLFTQPQ
Prm_Prm_78009/1-488 201 VVTWAKYMDDFNMANPESKTTLF------------------SAL 226
Pso_286221/1-593 303 VNTWLKFVDDFGKANPNTEKTLF------------------AVL 328
Pca_119793/1-485 192 IVTWAKYFEDFHKVKPNKEATLF------------------TFL 217
Pin_PITG_15032T0/1-485 201 MVSWAKYVGDFNKVNPDKPVTLF------------------STL 226
Pso_286248/1-600 295 VTAWMKYMAAYNNKNPKEKVSFLKSWEYVVEDEKEVWKLVDSPL 338
Pso_286223/1-593 301 VNTWLKFVDDFGKANPNTEKTLF------------------AVL 326
Conservation
877*7*974593546*5433988------------------73*
Quality
Consensus
VVTWAKYVDDFNKANPN+E+TLF------------------SVL
Prm_Prm_78009/1-488 227 KKRYKDEEATLVQMLIAAEKVPSTKGIATRVQAEQTKLWL---- 266
Pso_286221/1-593 329 QKRY--DEPTLVQMLIKAKNIPSMESVAVRIQAEQTKFWLGVDV 370
Pca_119793/1-485 218 QPRY--TEATLAHMLIAAEKVPATKSIATRVQAELTSLWL---- 255
Pin_PITG_15032T0/1-485 227 ATRL--NDETLLQMLIAAKNVPSTEKIAVQVQAVQTKLWL---- 264
Pso_286248/1-600 339 RKAY--NDEELVTMLIAAKKVPSTEKMARRVQAELTQSWM---- 376
Pso_286223/1-593 327 QKRY--DEPTLVQMLIKAKNIPSMESVAVRIQAEQTKFWLGVDV 368
Conservation
4447--7937*75***5*769*87748*489**47*54*9----
Quality
Consensus
QKRY--+E+TLVQMLIAAK+VPSTESIAVRVQAEQTKLWLGVDV
Prm_Prm_78009/1-488 267 TEGKE--PLDVFKLLGLD--EVGLTLLNSPLFSAWVKYTDDFRK 306
Pso_286221/1-593 371 ETAKE--PARVFELLRIK-GKDGVALLSNPLFIAWIKYTDEFNR 411
Pca_119793/1-485 256 TNKVE--PADVFKLLQLD--EIGMPLLKDPVFNAWVKYTDDFHK 295
Pin_PITG_15032T0/1-485 265 NTQKE--PGDVFKLLRLD--KEGHDIIQNPLFRAWVQYTDDFRK 304
Pso_286248/1-600 377 KDPKNHLPDDIFDLLKLT---DKETLLGDPLFSAWLKYLDEFNY 417
Pso_286223/1-593 369 ETAKE--PARVFELLRLNFMYDGPTLFSSPQFNAWIKYTEEYRK 410

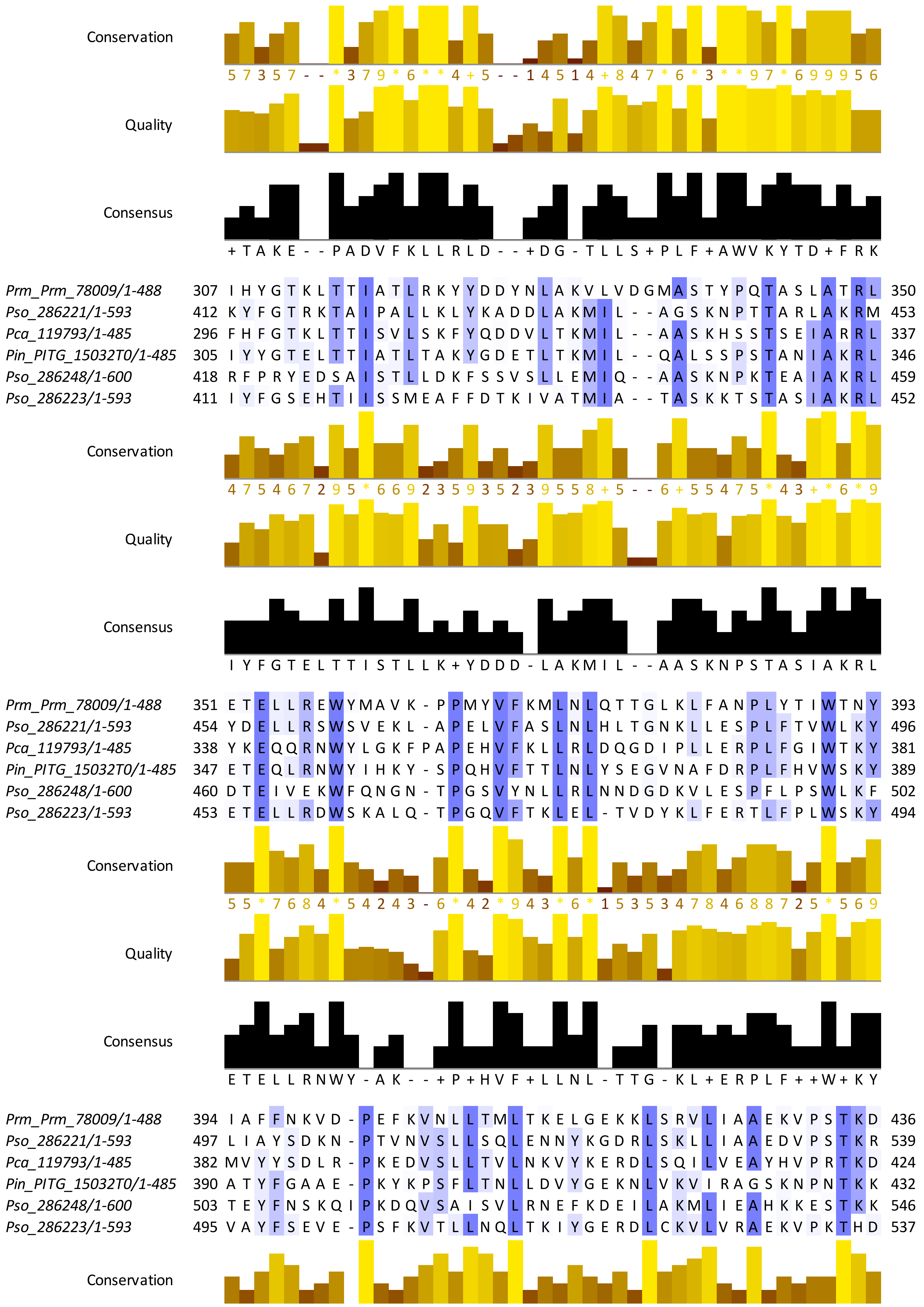
Conservation
5 7 3 5 7 - - * 3 7 9 * 6 * * 4 + 5 - - 1 4 5 1 4 + 8 4 7 * 6 * 3 * * 9 7 * 6 9 9 9 5 6
Quality
Consensus
+ T A K E - - P A D V F K L L R L D - - + D G - T L L S + P L F + A W V K Y T D + F R K
Prm_Prm_78009/1-488 307 I H Y G T K L T T I A T L R K Y Y D D Y N L A K V L V D G M A S T Y P Q T A S L A T R L 350
Pso_286221/1-593 412 K Y F G T R K T A I P A L L K L Y K A D D L A K M I L - - A G S K N P T T A R L A K R M 453
Pca_119793/1-485 296 F H F G T K L T T I S V L S K F Y Q D D V L T K M I L - - A A S K H S S T S E I A R R L 337
Pin_PITG_15032T0/1-485 305 I Y Y G T E L T T I A T L T A K Y G D E T L T K M I L - - Q A L S S P S T A N I A K R L 346
Pso_286248/1-600 418 R F P R Y E D S A I S T L L D K F S S V S L L E M I Q - - A A S K N P K T E A I A K R L 459
Pso_286223/1-593 411 I Y F G S E H T I I S S M E A F F D T K I V A T M I A - - T A S K K T S T A S I A K R L 452
Conservation
4 7 5 4 6 7 2 9 5 * 6 6 9 2 3 5 9 3 5 2 3 9 5 5 8 + 5 - - 6 + 5 5 4 7 5 * 4 3 + * 6 * 9
Quality
Consensus
I Y F G T E L T T I S T L L K + Y D D D - L A K M I L - - A A S K N P S T A S I A K R L
Prm_Prm_78009/1-488 351 E T E L L R E W Y M A V K - P P M Y V F K M L N L Q T T G L K L F A N P L Y T I W T N Y 393
Pso_286221/1-593 454 Y D E L L R S W S V E K L - A P E L V F A S L N L H L T G N K L L E S P L F T V W L K Y 496
Pca_119793/1-485 338 Y K E Q Q R N W Y L G K F P A P E H V F K L L R L D Q G D I P L L E R P L F G I W T K Y 381
Pin_PITG_15032T0/1-485 347 E T E Q L R N W Y I H K Y - S P Q H V F T T L N L Y S E G V N A F D R P L F H V W S K Y 389
Pso_286248/1-600 460 D T E I V E K W F Q N G N - T P G S V Y N L L R L N N D G D K V L E S P F L P S W L K F 502
Pso_286223/1-593 453 E T E L L R D W S K A L Q - T P G Q V F T K L E L - T V D Y K L F E R T L F P L W S K Y 494
Conservation
5 5 * 7 6 8 4 * 5 4 2 4 3 - 6 * 4 2 * 9 4 3 * 6 * 1 5 3 5 3 4 7 8 4 6 8 8 7 2 5 * 5 6 9
Quality
Consensus
E T E L L R N W Y - A K - - + P + H V F + L L N L - T T G - K L + E R P L F + + W + K Y
Prm_Prm_78009/1-488 394 I A F F N K V D - P E F K V N L L T M L T K E L G E K K L S R V L I A A E K V P S T K D 436
Pso_286221/1-593 497 L I A Y S D K N - P T V N V S L L S Q L E N N Y K G D R L S K L L I A A E D V P S T K R 539
Pca_119793/1-485 382 M V Y Y S D L R - P K E D V S L L T V L N K V Y K E R D L S Q I L V E A Y H V P R T K D 424
Pin_PITG_15032T0/1-485 390 A T Y F G A A E - P K Y K P S F L T N L L D V Y G E K N L V K V I R A G S K N P N T K K 432
Pso_286248/1-600 503 T E Y F N S K Q I P K D Q V S A I S V L R N E F K D E I L A K M L I E A H K K E S T K K 546
Pso_286223/1-593 495 V A Y F S E V E - P S F K V T L L N Q L T K I Y G E R D L C K V L V R A E K V P K T H D 537
Conservation

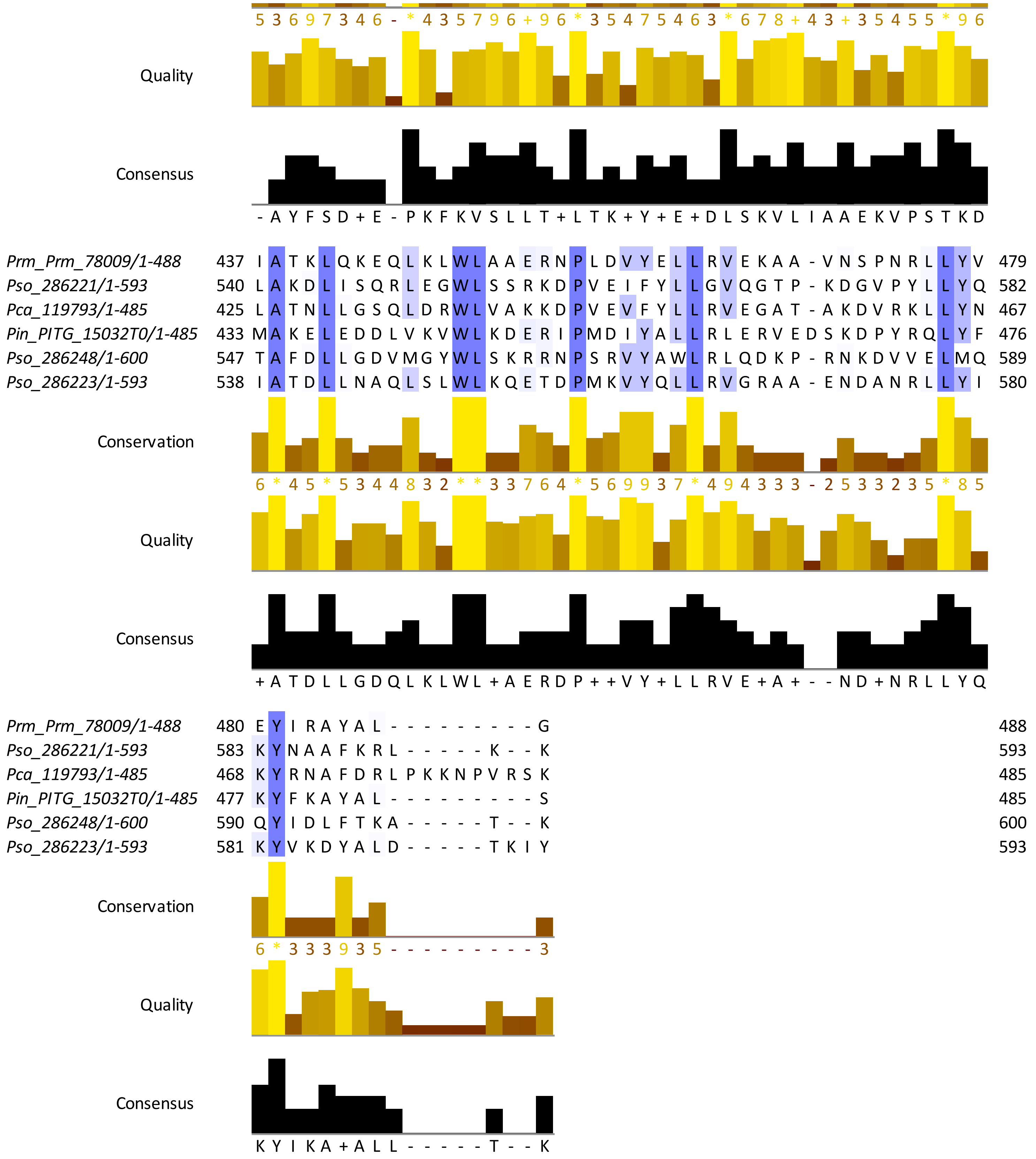

5 3 6 9 7 3 4 6 - * 4 3 5 7 9 6 + 9 6 * 3 5 4 7 5 4 6 3 * 6 7 8 + 4 3 + 3 5 4 5 5 * 9 6
Quality
Consensus
- A Y F S D + E - P K F K V S L L T + L T K + Y + E + D L S K V L I A A E K V P S T K D
Prm_Prm_78009/1-488 437 I A T K L Q K E Q L K L W L A A E R N P L D V Y E L L R V E K A A - V N S P N R L L Y V 479
Pso_286221/1-593 540 L A K D L I S Q R L E G W L S S R K D P V E I F Y L L G V Q G T P - K D G V P Y L L Y Q 582
Pca_119793/1-485 425 L A T N L L G S Q L D R W L V A K K D P V E V F Y L L R V E G A T - A K D V R K L L Y N 467
Pin_PITG_15032T0/1-485 433 M A K E L E D D L V K V W L K D E R I P M D I Y A L L R L E R V E D S K D P Y R Q L Y F 476
Pso_286248/1-600 547 T A F D L L G D V M G Y W L S K R R N P S R V Y A W L R L Q D K P - R N K D V V E L M Q 589
Pso_286223/1-593 538 I A T D L L N A Q L S L W L K Q E T D P M K V Y Q L L R V G R A A - E N D A N R L L Y I 580
Conservation
6 * 4 5 * 5 3 4 4 8 3 2 * * 3 3 7 6 4 * 5 6 9 9 3 7 * 4 9 4 3 3 3 - 2 5 3 3 2 3 5 * 8 5
Quality
Consensus
+ A T D L L G D Q L K L W L + A E R D P + + V Y + L L R V E + A + - - N D + N R L L Y Q
Prm_Prm_78009/1-488 480 E Y I R A Y A L - - - - - - - - - G 488
Pso_286221/1-593 583 K Y N A A F K R L - - - - - K - - K 593
Pca_119793/1-485 468 K Y R N A F D R L P K K N P V R S K 485
Pin_PITG_15032T0/1-485 477 K Y F K A Y A L - - - - - - - - - - S 485
Pso_286248/1-600 590 Q Y I D L F T K A - - - - - T - - K 600
Pso_286223/1-593 581 K Y V K D Y A L D - - - - - T K I Y 593
Conservation
6 * 3 3 3 9 3 5 - - - - - - - - - 3
Quality
Consensus
K Y I K A + A L L - - - - - T - - K

Ortholog 14

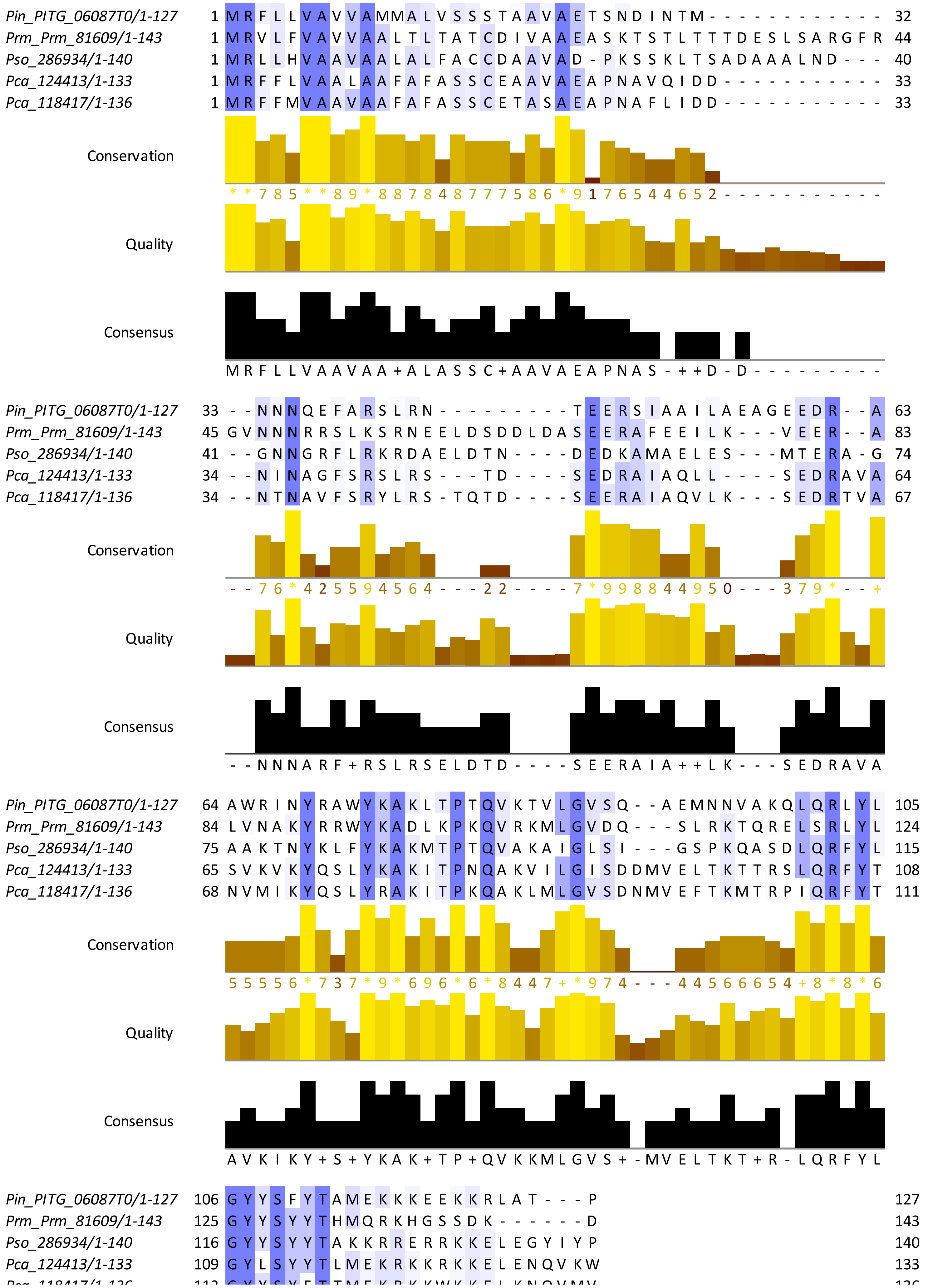
Pin_PITG_06087T0/1-127 1 MRFLLVAVVAMMALVSSSTAAVAETSNDINTM------------ 32
Prm_Prm_81609/1-143 1 MRVLFVAVVAALTLTATCDIVAAEASKTSTLTTTDESLSARGFR 44
Pso_286934/1-140 1 MRLLHVAAVAALALFACCDAAVAD-PKSSKLTSADAAALND--- 40
Pca_124413/1-133 1 MRFFLVAALAAFAFASSCEAAVAEAPNAVQIDD----------- 33
Pca_118417/1-136 1 MRFFMVAAVAAFAFASSCETASAEAPNAFLIDD----------- 33
Conservation
**785**89*887848777586*917654465 2------------
Quality
Consensus
MRFLLVAAVAA+ALASSC+AAVAEAPNAS-++D-D---------
Pin_PITG_06087T0/1-127 33 --NNNQEFARSLRN---------TEERSIAAILAEAGEEDR--A 63
Prm_Prm_81609/1-143 45 GVNNNRRSLKSRNEELDSDDLDASEERAFEEILK---VEER--A 83
Pso_286934/1-140 41 --GNNGRFLRKRDAELDTN----DEDKAMAELES---MTERA-G 74
Pca_124413/1-133 34 --NINAGFSRSLRS---TD----SEDRAIAQLL----SEDRAVA 64
Pca_118417/1-136 34 --NTNAVFSRYLRS-TQTD----SEERAIAQVLK---SEDRTVA 67
Conservation
--76*42559456 4---22----7*998844950---379*--+
Quality
Consensus
--NNNARF+RSLRSELDTD----SEERAIA++LK---SEDRAVA
Pin_PITG_06087T0/1-127 64 AWRINYRAWYKAKLTPTQVKTVLGVSQ--AEMNNVAKQLQRLYL 105
Prm_Prm_81609/1-143 84 LVNAKYRRWYKADLKPKQVRKMLGVDQ---SLRKTQRELSRLYL 124
Pso_286934/1-140 75 AAKTNYKLFYKAKMTPTQVAKAIGLSI---GSPKQASDLQRFYL 115
Pca_124413/1-133 65 SVKVKYQSLYKAKITPNQAKVILGISDDMVELTKTTRSLQRFYT 108
Pca_118417/1-136 68 NVMIKYQSLYRAKITPKQAKLMLGVSDNMVEFTKMTRPIQRFYT 111
Conservation
55556*737*9*696*6*8447+*974---44566654+8*8*6
Quality
Consensus
AVKIKY+S+YKAK+TP+QVKKMLGVS+-MVELTKT+R-LQRFYL
Pin_PITG_06087T0/1-127 106 GYYSFYTAMEKKKEEKKRLAT---P 127
Prm_Prm_81609/1-143 125 GYYSYYTHMQRKHGSSDK------D 143
Pso_286934/1-140 116 GYYSYYTAKKRRERRKKELEGYIYP 140
Pca_124413/1-133 109 GYLSYYTLMEKRKKRKKELENQVKW 133

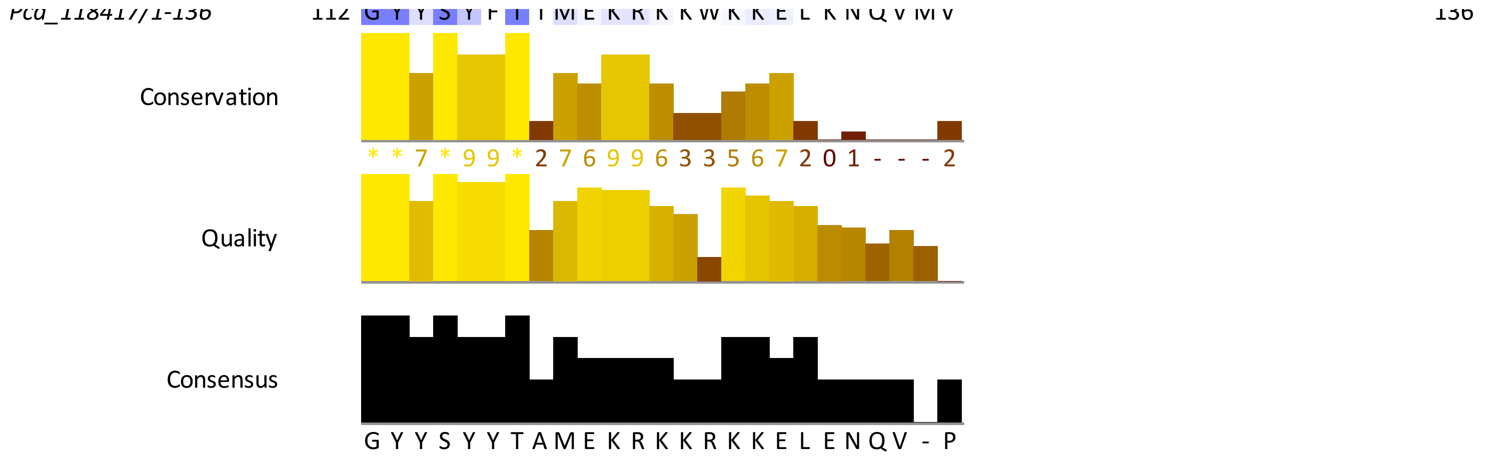
Pca_118417/1-136 112 G Y Y S Y F T I M E K R K K W K K E L K N Q V M V 136
Conservation
* * 7 * 9 9 * 2 7 6 9 9 6 3 3 5 6 7 2 0 1 - - - 2
Quality
Consensus
G Y Y S Y Y T A M E K R K K R K K E L E N Q V - P

Ortholog 15

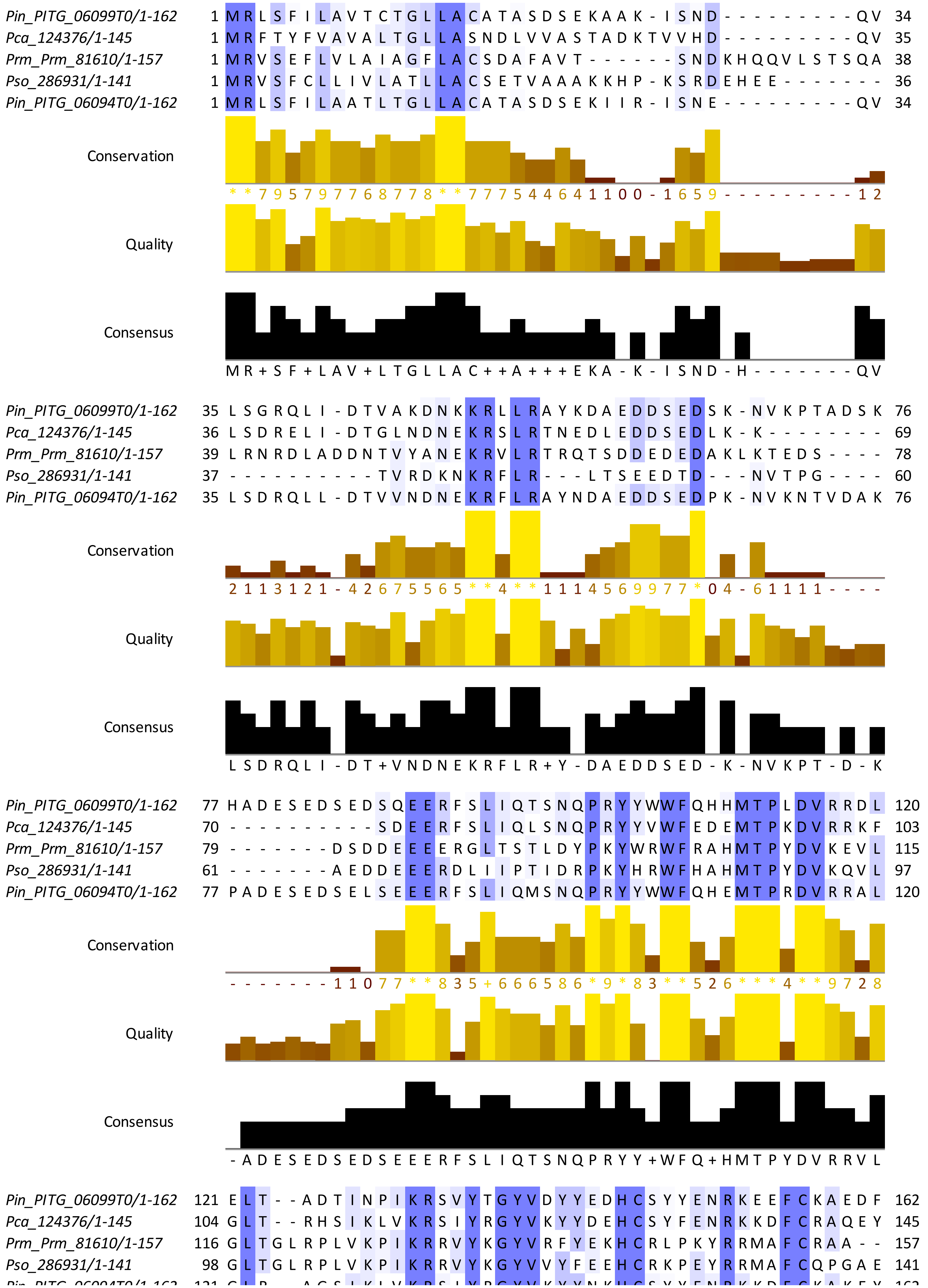
Pin_PITG_06099T0/1-162 1 M R L S F I L A V T C T G L L A C A T A S D S E K A A K - I S N D - - - - - - - - - Q V 34
Pca_124376/1-145 1 M R F T Y F V A V A L T G L L A S N D L V V A S T A D K T V V H D - - - - - - - - - Q V 35
Prm_Prm_81610/1-157 1 M R V S E F L V L A I A G F L A C S D A F A V T - - - - - - S N D K H Q Q V L S T S Q A 38
Pso_286931/1-141 1 M R V S F C L L I V L A T L L A C S E T V A A A K K H P - K S R D E H E E - - - - - - - 36
Pin_PITG_06094T0/1-162 1 M R L S F I L A A T L T G L L A C A T A S D S E K I I R - I S N E - - - - - - - - - Q V 34
Conservation
* * 7 9 5 7 9 7 7 6 8 7 7 8 * * 7 7 7 5 4 4 6 4 1 1 0 0 - 1 6 5 9 - - - - - - - - - 1 2
Quality
Consensus
M R + S F + L A V + L T G L L A C + + A + + + E K A - K - I S N D - H - - - - - - - Q V
Pin_PITG_06099T0/1-162 35 L S G R Q L I - D T V A K D N K K R L L R A Y K D A E D D S E D S K - N V K P T A D S K 76
Pca_124376/1-145 36 L S D R E L I - D T G L N D N E K R S L R T N E D L E D D S E D L K - K - - - - - - - - 69
Prm_Prm_81610/1-157 39 L R N R D L A D D N T V Y A N E K R V L R T R Q T S D D E D E D A K L K T E D S - - - - 78
Pso_286931/1-141 37 - - - - - - - - - - T V R D K N K R F L R - - - L T S E E D T D - - - N V T P G - - - - 60
Pin_PITG_06094T0/1-162 35 L S D R Q L L - D T V V N D N E K R F L R A Y N D A E D D S E D P K - N V K N T V D A K 76
Conservation
2 1 1 3 1 2 1 - 4 2 6 7 5 5 6 5 * * 4 * * 1 1 1 4 5 6 9 9 7 7 * 0 4 - 6 1 1 1 1 - - - -
Quality
Consensus
L S D R Q L I - D T + V N D N E K R F L R + Y - D A E D D S E D - K - N V K P T - D - K
Pin_PITG_06099T0/1-162 77 H A D E S E D S E D S Q E E R F S L I Q T S N Q P R Y Y W W F Q H H M T P L D V R R D L 120
Pca_124376/1-145 70 - - - - - - - - - - S D E E R F S L I Q L S N Q P R Y Y V W F E D E M T P K D V R R K F 103
Prm_Prm_81610/1-157 79 - - - - - - - D S D D E E E E R G L T S T L D Y P K Y W R W F R A H M T P Y D V K E V L 115
Pso_286931/1-141 61 - - - - - - - A E D D E E E R D L I I P T I D R P K Y H R W F H A H M T P Y D V K Q V L 97
Pin_PITG_06094T0/1-162 77 P A D E S E D S E L S E E E R F S L I Q M S N Q P R Y Y W W F Q H E M T P R D V R R A L 120
Conservation
- - - - - - - 1 1 0 7 7 * * 8 3 5 + 6 6 6 5 8 6 * 9 * 8 3 * * 5 2 6 * * * 4 * * 9 7 2 8
Quality
Consensus
- A D E S E D S E D S E E E R F S L I Q T S N Q P R Y Y + W F Q + H M T P Y D V R R V L
Pin_PITG_06099T0/1-162 121 E L T - - A D T I N P I K R S V Y T G Y V D Y Y E D H C S Y Y E N R K E E F C K A E D F 162
Pca_124376/1-145 104 G L T - - R H S I K L V K R S I Y R G Y V K Y Y D E H C S Y F E N R K K D F C R A Q E Y 145
Prm_Prm_81610/1-157 116 G L T G L R P L V K P I K R R V Y K G Y V R F Y E K H C R L P K Y R R M A F C R A A - - 157
Pso_286931/1-141 98 G L T G L R P L V K P I K R R V Y K G Y V V Y F E E H C R K P E Y R R M A F C Q P G A E 141

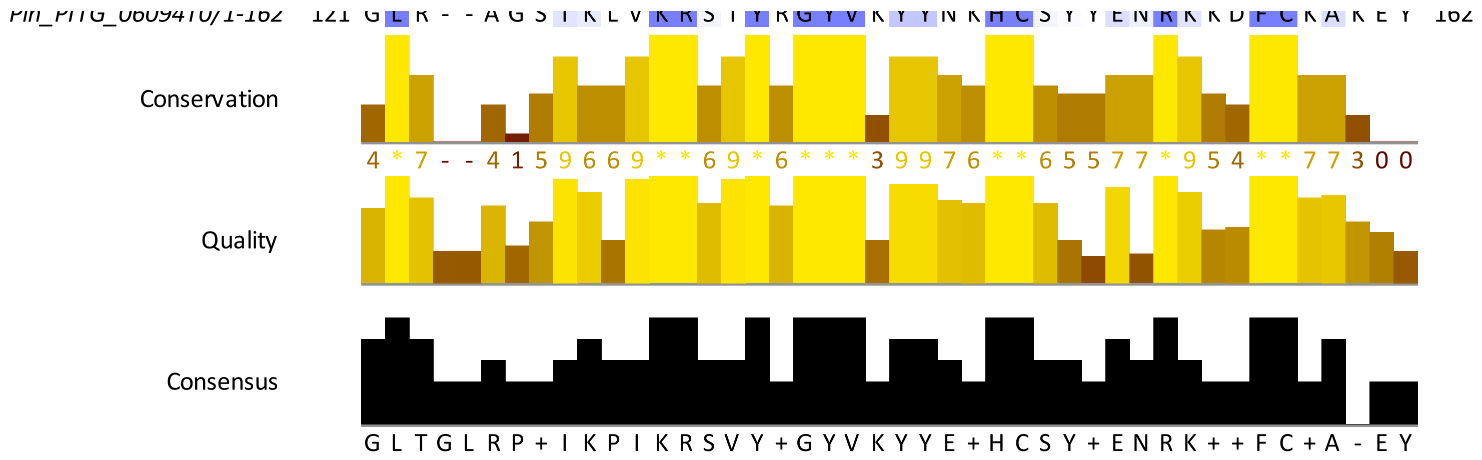


*Pin_PITG_06099T0/1-162*
*Pca_124376/1-145*
*Prm_Prm_81610/1-157*
*Pso_286931/1-141*
*Pin_PITG_06094T0/1-162*

Conservation

Quality

Consensus

Ortholog 16

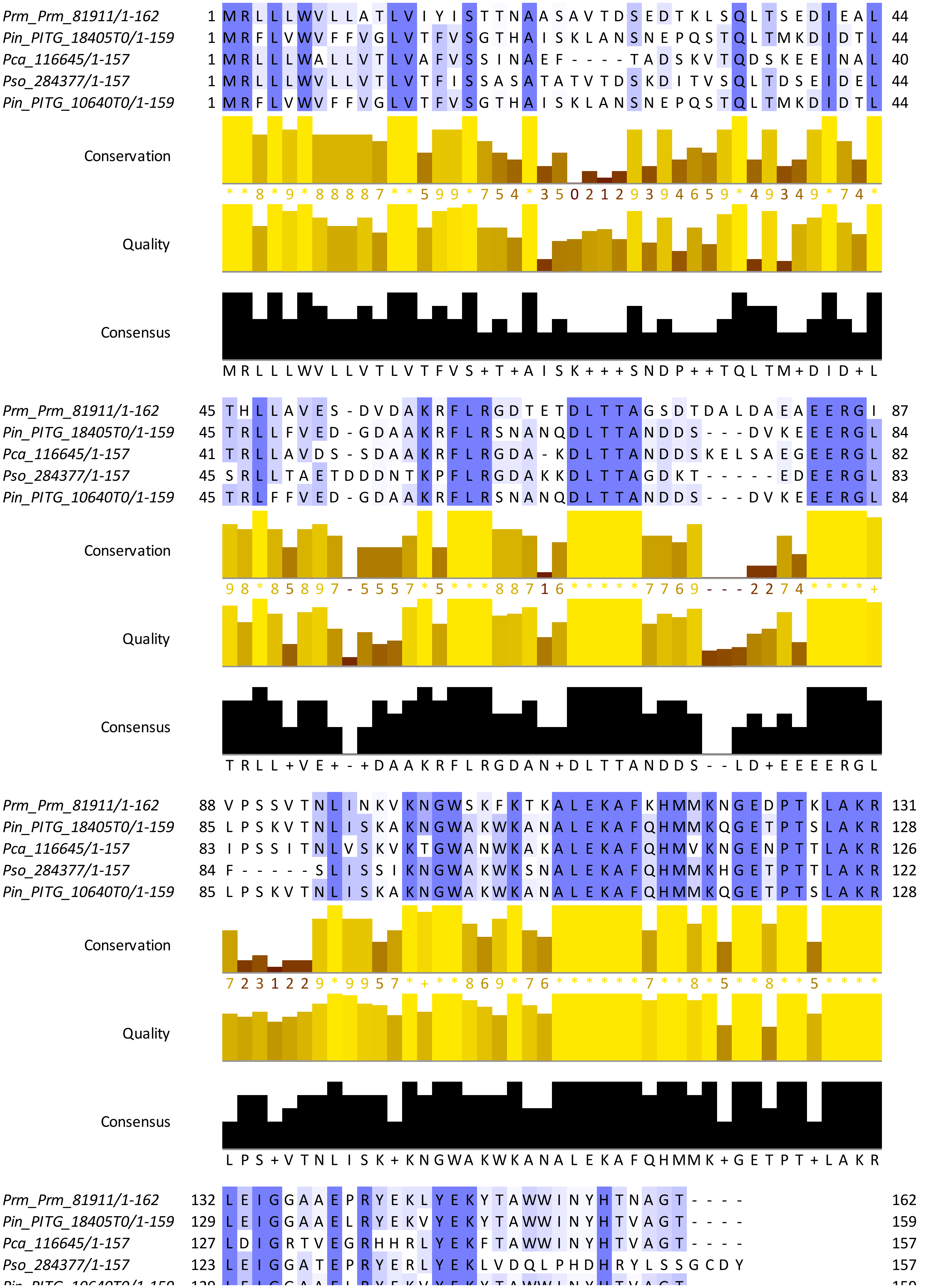
Prm_Prm_81911/1-162 1 MRLLLWVLLATLVIYISTTNAASAVTDSEDTKLSQLTSEDIEAL 44
Pin_PITG_18405T0/1-159 1 MRFLVWVFFVGLVTFVSGTHAISKLANSNEPQSTQLTMKDIDTL 44
Pca_116645/1-157 1 MRLLLWALLVTLVAFVSSINAEF----TADSKVTQDSKEEINAL 40
Pso_284377/1-157 1 MRLLLWVLLVTLVTFISSASATATVTDSKDITVSQLTDSEIDEL 44
Pin_PITG_10640T0/1-159 1 MRFLVWVFFVGLVTFVSGTHAISKLANSNEPQSTQLTMKDIDTL 44
Conservation
**8*9*88887**599*754*350212939465 9*49349*74*
Quality
Consensus
MRLLLWVLLVTLVTFVS+T+AISK+++SNDP++TQLTM+DID+L
Prm_Prm_81911/1-162 45 THLLAVES-DVDAKRFLRGDTETDLTTAGSDTDALDAEAEERGI 87
Pin_PITG_18405T0/1-159 45 TRLLFVED-GDAAKRFLRSNANQDLTTANDDS---DVKEEERGL 84
Pca_116645/1-157 41 TRLLAVDS-SDAAKRFLRGDA-KDLTTANDDSKELSAEGEERGL 82
Pso_284377/1-157 45 SRLLTAETDDDNTKPFLRGDAKKDLTTAGDKT-----EDEERGL 83
Pin_PITG_10640T0/1-159 45 TRLFFVED-GDAAKRFLRSNANQDLTTANDDS---DVKEEERGL 84
Conservation
98*85897-5557*5***88716*****7769---2274****+
Quality
Consensus
TRLL+VE+-+DAAKRFLRGDAN+DLTTANDDS--LD+EEEERGL
Prm_Prm_81911/1-162 88 VPSSVTNLINKVKNGWSKFKTKALEKAFKHMMKNGEDPTKLAKR 131
Pin_PITG_18405T0/1-159 85 LPSKVTNLISKAKNGWAKWKANALEKAFQHMMKQGETPTSLAKR 128
Pca_116645/1-157 83 IPSSITNLVSKVKTGWANWKAKALEKAFQHMVKNGENPTTLAKR 126
Pso_284377/1-157 84 F-----SLISSIKNGWAKWKSNALEKAFQHMMKHGETPTTLAKR 122
Pin_PITG_10640T0/1-159 85 LPSKVTNLISKAKNGWAKWKANALEKAFQHMMKQGETPTSLAKR 128
Conservation
7231229*9957*+**869*76******7**8*5**8**5****
Quality
Consensus
LPS+VTNLISK+KNGWAKWKANALEKAFQHMMK+GETPT+LAKR
Prm_Prm_81911/1-162 132 LEIGGAAEPRYEKLYEKYTAWWINYHTNAGT---- 162
Pin_PITG_18405T0/1-159 129 LEIGGAAELRYEKVYEKYTAWWINYHTVAGT---- 159
Pca_116645/1-157 127 LDIGRTVEGRHHRLYEKFTAWWINYHTVAGT---- 157
Pso_284377/1-157 123 LEIGGATEPRYERLYEKLVDQLPHDHRYLSSGCDY 157

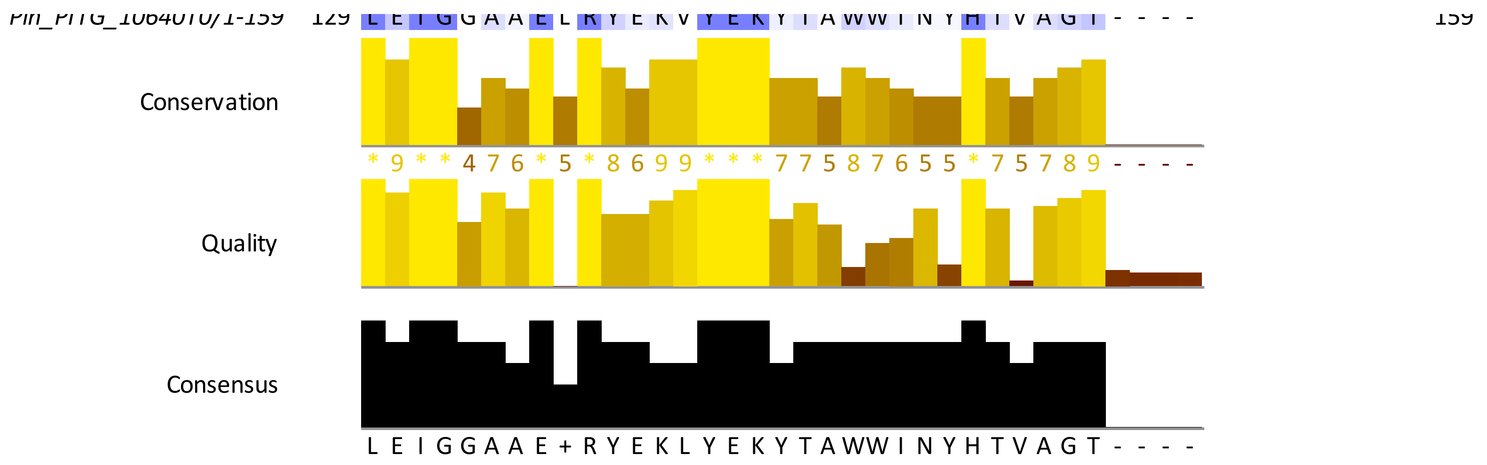
129 LEIGGAAELRYEKVYEKYTAWWINYHTVAGT---- 159
Conservation
* 9 * * 4 7 6 * 5 * 8 6 9 9 * * * 7 7 5 8 7 6 5 5 * 7 5 7 8 9 - - - -
Quality
Consensus
L E I G G A A E + R Y E K L Y E K Y T A W W I N Y H T V A G T - - - -

Ortholog 17

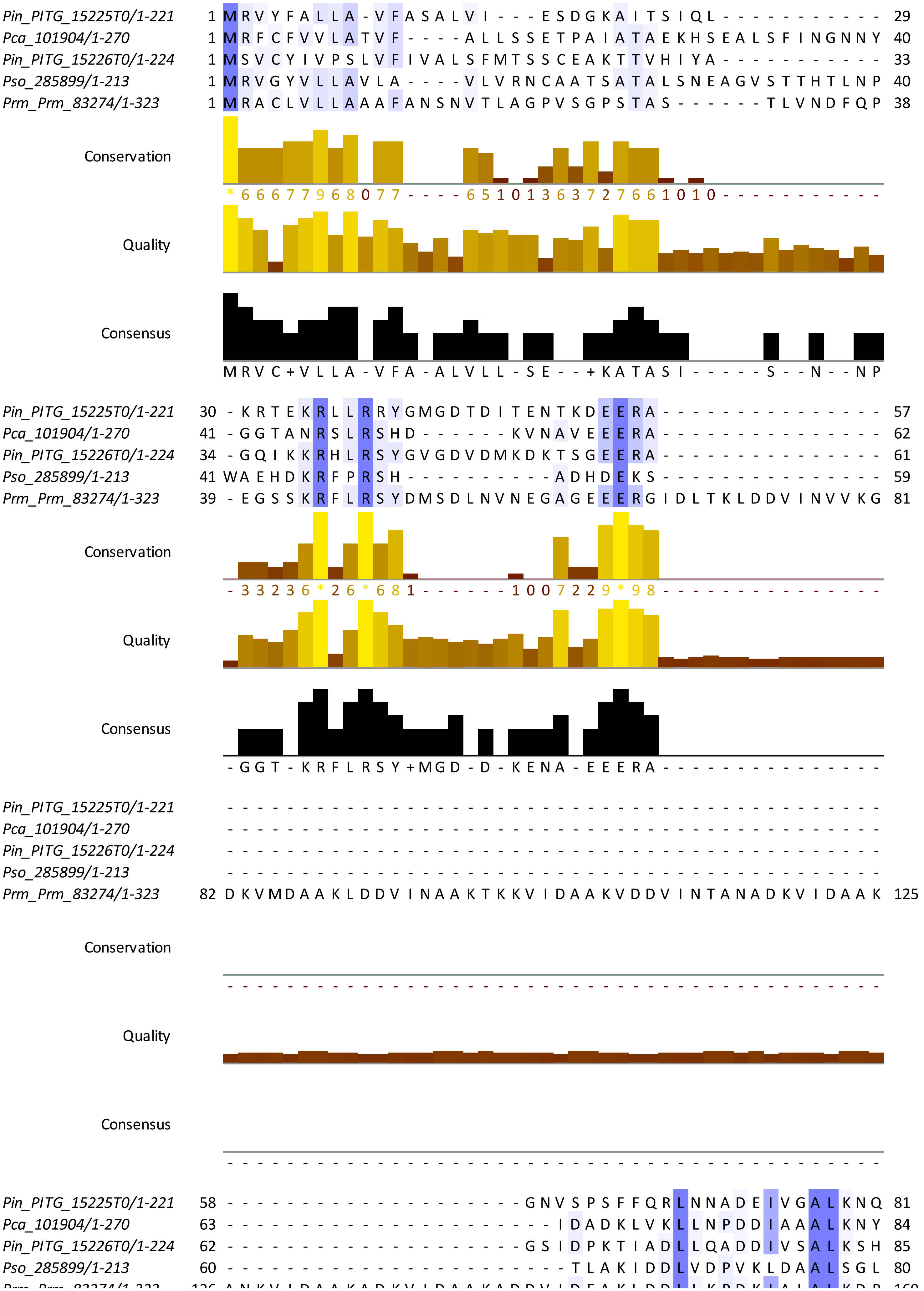

Pin_PITG_15225T0/1-221 1 MRVYFALLA-VFASALVI---ESDGKAITSIQL----------- 29
Pca_101904/1-270 1 MRFCFVVLATVF----ALLSSETPAIATAEKHSEALSFINGNNY 40
Pin_PITG_15226T0/1-224 1 MSVCYIVPSLVFIVALSFMTSSCEAKTTVHIYA----------- 33
Pso_285899/1-213 1 MRVGYVLLAVLA----VLVRNCAATSATALSNEAGVSTTHTLNP 40
Prm_Prm_83274/1-323 1 MRACLVLLAAAFANSNVTLAGPVSGPSTAS------TLVNDFQP 38
Conservation
*66677968077----65101363727661010-----------
Quality
Consensus
MRVC+VLLA-VFA-ALVLL-SE--+KATASI-----S--N--NP
Pin_PITG_15225T0/1-221 30 -KRTEKRLLRRYGMGDTDITENTKDEERA--------------- 57
Pca_101904/1-270 41 -GGTANRSLRSHD------KVNAVEEERA--------------- 62
Pin_PITG_15226T0/1-224 34 -GQIKKRHLRSYGVGDVDMKDKTSGEERA--------------- 61
Pso_285899/1-213 41 WAEHDKRFPRSH----------ADHDEKS--------------- 59
Prm_Prm_83274/1-323 39 -EGSSKRFLRSYDMSDLNVNEGAGEEERGIDLTKLDDVINVVKG 81
Conservation
-33236*26*681------1007229*98---------------
Quality
Consensus
-GGT-KRFLRSY+MGD-D-KENA-EEERA---------------
Pin_PITG_15225T0/1-221 --------------------------------------------
Pca_101904/1-270 --------------------------------------------
Pin_PITG_15226T0/1-224 --------------------------------------------
Pso_285899/1-213 --------------------------------------------
Prm_Prm_83274/1-323 82 DKVMDAAKLDDVINAAKTKKVIDAAKVDDVINTANADKVIDAAK 125
Conservation
--------------------------------------------
Quality
Consensus
--------------------------------------------
Pin_PITG_15225T0/1-221 58 --------------------GNVSPSFFQRLNNADEIVGALKNQ 81
Pca_101904/1-270 63 ----------------------IDADKLVKLLNPDDIAAALKNY 84
Pin_PITG_15226T0/1-224 62 --------------------GSIDPKTIADLLQADDIVSALKSH 85
Pso_285899/1-213 60 -----------------------TLAKIDDLVDPVKLDAALSGL 80
Prm_Prm_83274/1-323 [illegible]

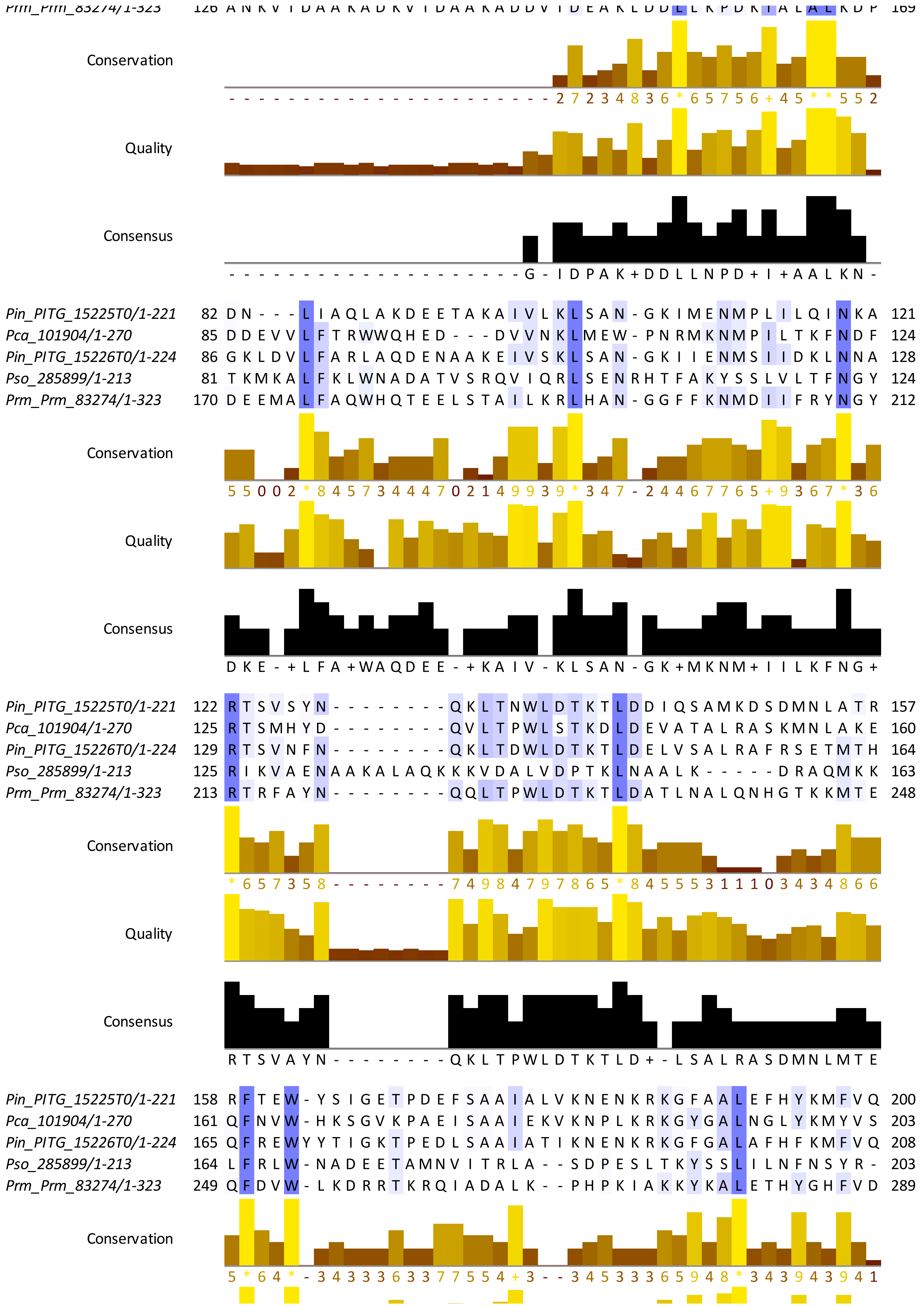

Prm_Prm_83274/1-323 126 ANKVIDAAKADKVIDAAKADDVIDEAKLDDLLKPDKIALALKDP 169
Conservation
----------------------272348 36*65756+45**552
Quality
Consensus
---------------------G-IDPAK+DDLLNPD+I+AALKN-
Pin_PITG_15225T0/1-221 82 DN---LIAQLAKDEETAKAIVLKLSAN-GKIMENMPLILQINKA 121
Pca_101904/1-270 85 DDEVVLFTRWWQHED---DVVNKLMEW-PNRMKNMPILTKFNDF 124
Pin_PITG_15226T0/1-224 86 GKLDVLFARLAQDENAAKEIVSKLSAN-GKIIENMSIIDKLNNA 128
Pso_285899/1-213 81 TKMKALFKLWNADATVSRQVIQRLSENRHTFAKYSSLVLTFNGY 124
Prm_Prm_83274/1-323 170 DEEMALFAQWHQTEELSTAILKRLHAN-GGFFKNMDIIFRYNGY 212
Conservation
55002*8457344470214993 9*347-24467765+9367*36
Quality
Consensus
DKE-+LFA+WAQDEE-+KAIV-KLSAN-GK+MKNM+IILKFNG+
Pin_PITG_15225T0/1-221 122 RTSVSYN--------QKLTNWLDTKTLDDIQSAMKDSDMNLATR 157
Pca_101904/1-270 125 RTSMHYD--------QVLTPWLSTKDLDEVATALRASKMNLAKE 160
Pin_PITG_15226T0/1-224 129 RTSVNFN--------QKLTDWLDTKTLDELVSALRAFRSETMTH 164
Pso_285899/1-213 125 RIKVAENAAKALAQKKKVDALVDPTKLNAALK-----DRAQMKK 163
Prm_Prm_83274/1-323 213 RTRFAYN--------QQLTPWLDTKTLDATLNALQNHGTKKMTE 248
Conservation
*657358--------74984797865*845553111034348 66
Quality
Consensus
RTSVAYN--------QKLTPWLDTKTLD+-LSALRASDMNLMTE
Pin_PITG_15225T0/1-221 158 RFTEW-YSIGETPDEFSAAIALVKNENKRKGFAALEFHYKMFVQ 200
Pca_101904/1-270 161 QFNVW-HKSGVKPAEISAAIEKVKNPLKRKGYGALNGLYKMYVS 203
Pin_PITG_15226T0/1-224 165 QFREWYYTIGKTPEDLSAAIATIKNENKRKGFGALAFHFKMFVQ 208
Pso_285899/1-213 164 LFRLW-NADEETAMNVITRLA--SDPESLTKYSSLILNFNSYR- 203
Prm_Prm_83274/1-323 249 QFDVW-LKDRRTKRQIADALK--PHPKIAKKYKALETHYGHFVD 289
Conservation
5*64*-3433363377554+3--34533365948*343943941

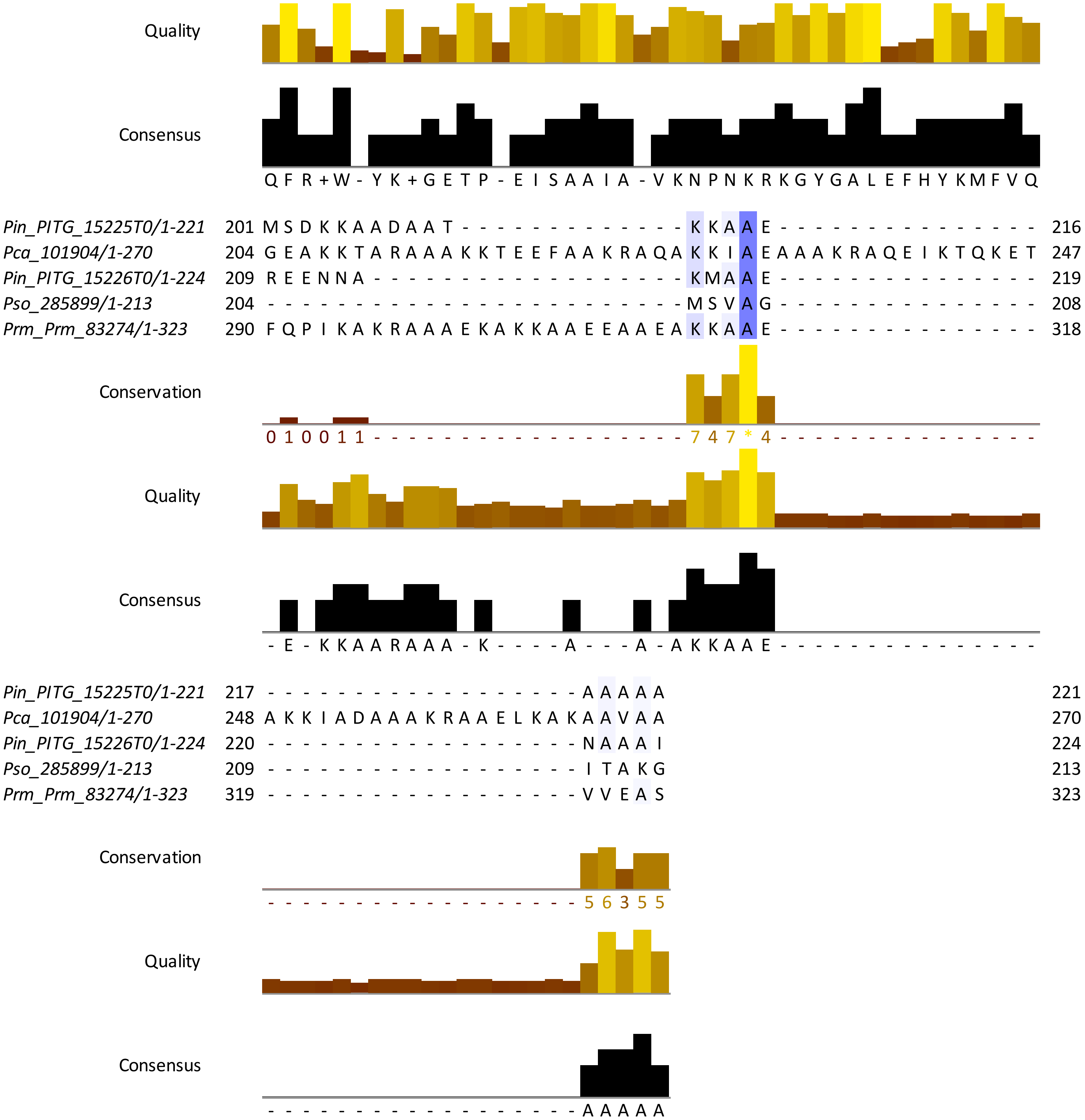
Quality
Consensus
Q F R + W - Y K + G E T P - E I S A A I A - V K N P N K R K G Y G A L E F H Y K M F V Q
Pin_PITG_15225T0/1-221 201 M S D K K A A D A A T - - - - - - - - - - - - - K K A A E - - - - - - - - - - - - - - - 216
Pca_101904/1-270 204 G E A K K T A R A A A K K T E E F A A K R A Q A K K I A E A A A K R A Q E I K T Q K E T 247
Pin_PITG_15226T0/1-224 209 R E E N N A - - - - - - - - - - - - - - - - - - K M A A E - - - - - - - - - - - - - - - 219
Pso_285899/1-213 204 - - - - - - - - - - - - - - - - - - - - - - - - M S V A G - - - - - - - - - - - - - - - 208
Prm_Prm_83274/1-323 290 F Q P I K A K R A A A E K A K K A A E E A A E A K K A A E - - - - - - - - - - - - - - - 318
Conservation
0 1 0 0 1 1 - - - - - - - - - - - - - - - - - - 7 4 7 * 4 - - - - - - - - - - - - - - -
Quality
Consensus
- E - K K A A R A A A - K - - - - A - - - A - A K K A A E - - - - - - - - - - - - - - -
Pin_PITG_15225T0/1-221 217 - - - - - - - - - - - - - - - - - - A A A A A 221
Pca_101904/1-270 248 A K K I A D A A A K R A A E L K A K A A V A A 270
Pin_PITG_15226T0/1-224 220 - - - - - - - - - - - - - - - - - - N A A A I 224
Pso_285899/1-213 209 - - - - - - - - - - - - - - - - - - I T A K G 213
Prm_Prm_83274/1-323 319 - - - - - - - - - - - - - - - - - - V V E A S 323
Conservation
- - - - - - - - - - - - - - - - - - 5 6 3 5 5
Quality
Consensus
- - - - - - - - - - - - - - - - - - A A A A A

Ortholog 18

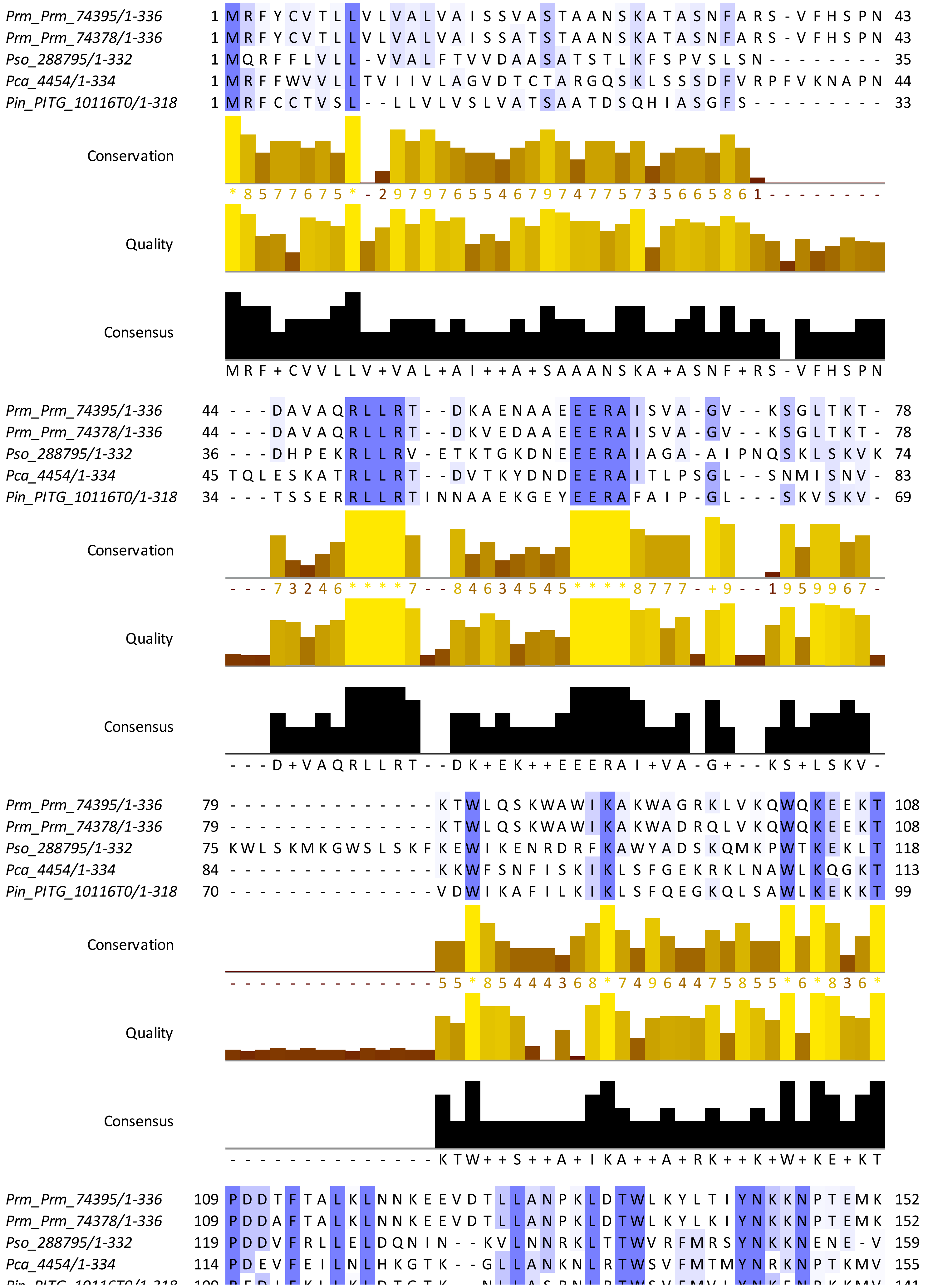
Prm_Prm_74395/1-336 1 MRFYCVTLLVLVALVAISSVASTAANSKATASNFARS-VFHSPN 43
Prm_Prm_74378/1-336 1 MRFYCVTLLVLVALVAISSATSTAANSKATASNFARS-VFHSPN 43
Pso_288795/1-332 1 MQRFFLVLL-VVALFTVVDAASATSTLKFSPVSLSN-------- 35
Pca_4454/1-334 1 MRFFWVVLLTVIIVLAGVDTCTARGQSKLSSSDFVRPFVKNAPN 44
Pin_PITG_10116T0/1-318 1 MRFCCTVSL--LLVLVSLVATSAATDSQHIASGFS--------- 33
Conservation
*85776 75*-2979765546797477573566586 1--------
Quality
Consensus
MRF+CVVLLV+VAL+AI++A+SAAANSKA+ASNF+RS-VFHSPN
Prm_Prm_74395/1-336 44 ---DAVAQRLLRT--DKAENAAEEERAISVA-GV--KSGLTKT- 78
Prm_Prm_74378/1-336 44 ---DAVAQRLLRT--DKVEDAAEEERAISVA-GV--KSGLTKT- 78
Pso_288795/1-332 36 ---DHPEKRLLRV-ETKTGKDNEEERAIAGA-AIPNQSKLSKVK 74
Pca_4454/1-334 45 TQLESKATRLLRT--DVTKYDNDEERAITLPSGL--SNMISNV- 83
Pin_PITG_10116T0/1-318 34 ---TSSERRLLRTINNAAEKGEYEERAFAIP-GL---SKVSKV- 69
Conservation
---73246****7--84634545****8777-+9--195996 7-
Quality
Consensus
---D+VAQRLLRT--DK+EK++EEERAI+VA-G+--KS+LSKV-
Prm_Prm_74395/1-336 79 ---------------KTWLQSKWAWIKAKWAGRKLVKQWQKEEKT 108
Prm_Prm_74378/1-336 79 ---------------KTWLQSKWAWIKAKWADRQLVKQWQKEEKT 108
Pso_288795/1-332 75 KWLSKMKGWSLSKFKEWIKENRDRFKAWYADSKQMKPWTKEKLT 118
Pca_4454/1-334 84 ---------------KKWFSNFISKIKLSFGEKRKLNAWLKQGKT 113
Pin_PITG_10116T0/1-318 70 ---------------VDWIKAFILKIKLSFQEGKQLSAWLKEKKT 99
Conservation
---------------55*85444368*7496447585 5*6*836*
Quality
Consensus
---------------KTW++S++A+IKA++A+RK++K+W+KE+KT
Prm_Prm_74395/1-336 109 PDDTFTALKLNNKEEVDTLLANPKLDTWLKYLTIYNKKNPTEMK 152
Prm_Prm_74378/1-336 109 PDDAFTALKLNNKEEVDTLLANPKLDTWLKYLKIYNKKNPTEMK 152
Pso_288795/1-332 119 PDDVFRLLELDQNIN--KVLNNRKLTTWVRFMRSYNKKNENE-V 159
Pca_4454/1-334 114 PDEVFEILNLHKGTK--GLLANKNLRTWSVFMTMYNRKNPTKMV 155

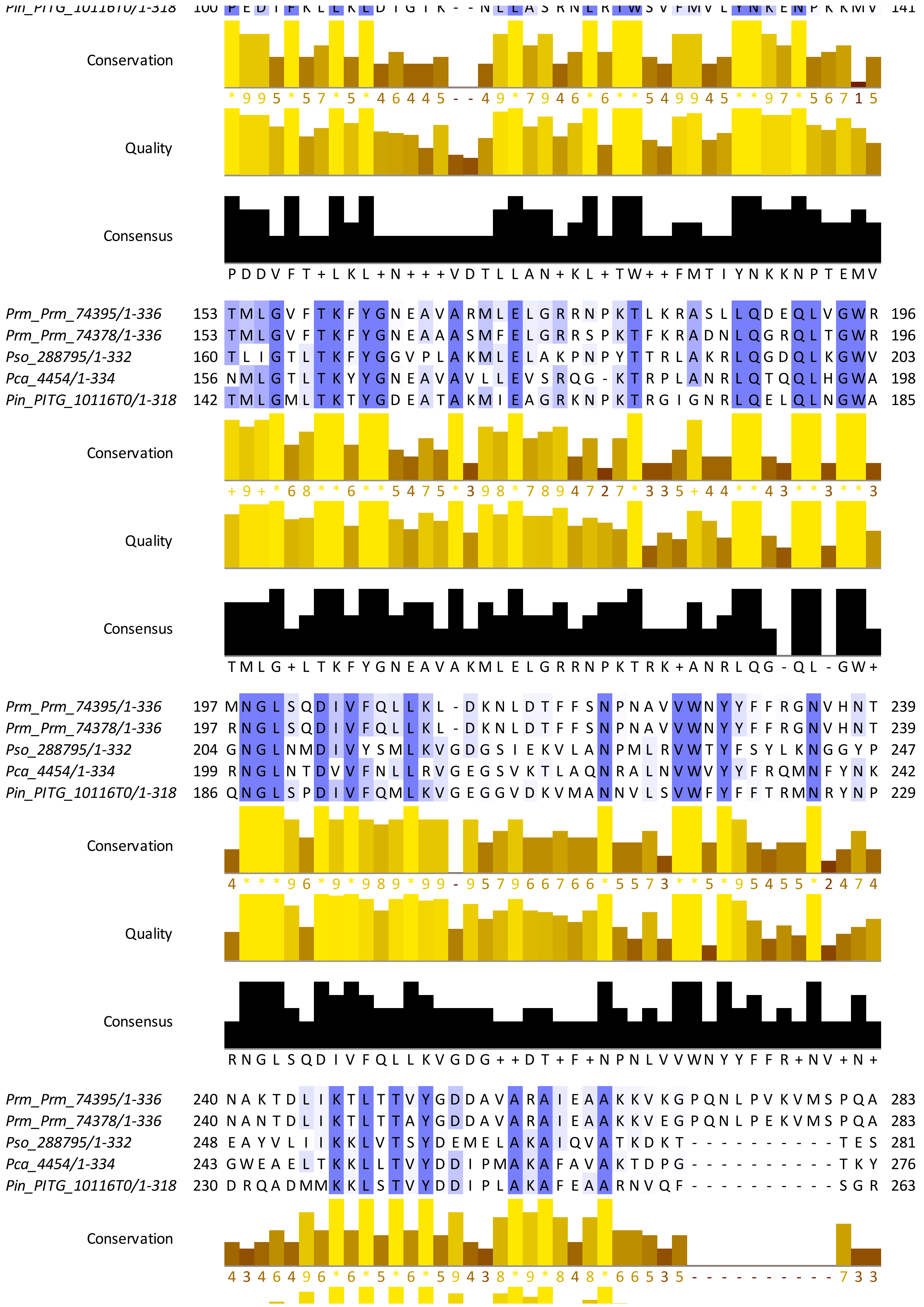

Pin_PITG_10116T0/1-318 100 P E D I F K L L K L D I G I K - - N L L A S R N L R I W S V F M V L Y N K E N P K K M V 141
Conservation
* 9 9 5 * 5 7 * 5 * 4 6 4 4 5 - - 4 9 * 7 9 4 6 * 6 * * 5 4 9 9 4 5 * * 9 7 * 5 6 7 1 5
Quality
Consensus
P D D V F T + L K L + N + + + V D T L L A N + K L + T W + + F M T I Y N K K N P T E M V
Prm_Prm_74395/1-336 153 T M L G V F T K F Y G N E A V A R M L E L G R R N P K T L K R A S L L Q D E Q L V G W R 196
Prm_Prm_74378/1-336 153 T M L G V F T K F Y G N E A A A S M F E L G R R S P K T F K R A D N L Q G R Q L T G W R 196
Pso_288795/1-332 160 T L I G T L T K F Y G G V P L A K M L E L A K P N P Y T T R L A K R L Q G D Q L K G W V 203
Pca_4454/1-334 156 N M L G T L T K Y Y G N E A V A V L L E V S R Q G - K T R P L A N R L Q T Q Q L H G W A 198
Pin_PITG_10116T0/1-318 142 T M L G M L T K T Y G D E A T A K M I E A G R K N P K T R G I G N R L Q E L Q L N G W A 185
Conservation
+ 9 + * 6 8 * * 6 * * 5 4 7 5 * 3 9 8 * 7 8 9 4 7 2 7 * 3 3 5 + 4 4 * * 4 3 * * 3 * * 3
Quality
Consensus
T M L G + L T K F Y G N E A V A K M L E L G R R N P K T R K + A N R L Q G - Q L - G W +
Prm_Prm_74395/1-336 197 M N G L S Q D I V F Q L L K L - D K N L D T F F S N P N A V V W N Y Y F F R G N V H N T 239
Prm_Prm_74378/1-336 197 R N G L S Q D I V F Q L L K L - D K N L D T F F S N P N A V V W N Y Y F F R G N V H N T 239
Pso_288795/1-332 204 G N G L N M D I V Y S M L K V G D G S I E K V L A N P M L R V W T Y F S Y L K N G G Y P 247
Pca_4454/1-334 199 R N G L N T D V V F N L L R V G E G S V K T L A Q N R A L N V W V Y Y F R Q M N F Y N K 242
Pin_PITG_10116T0/1-318 186 Q N G L S P D I V F Q M L K V G E G G V D K V M A N N V L S V W F Y F F T R M N R Y N P 229
Conservation
4 * * * 9 6 * 9 * 9 8 9 * 9 9 - 9 5 7 9 6 6 7 6 6 * 5 5 7 3 * * 5 * 9 5 4 5 5 * 2 4 7 4
Quality
Consensus
R N G L S Q D I V F Q L L K V G D G + + D T + F + N P N L V V W N Y Y F F R + N V + N +
Prm_Prm_74395/1-336 240 N A K T D L I K T L T T V Y G D D A V A R A I E A A K K V K G P Q N L P V K V M S P Q A 283
Prm_Prm_74378/1-336 240 N A N T D L I K T L T T A Y G D D A V A R A I E A A K K V E G P Q N L P E K V M S P Q A 283
Pso_288795/1-332 248 E A Y V L I I K K L V T S Y D E M E L A K A I Q V A T K D K T - - - - - - - - - - T E S 281
Pca_4454/1-334 243 G W E A E L T K K L L T V Y D D I P M A K A F A V A K T D P G - - - - - - - - - - T K Y 276
Pin_PITG_10116T0/1-318 230 D R Q A D M M K K L S T V Y D D I P L A K A F E A A R N V Q F - - - - - - - - - - S G R 263
Conservation
4 3 4 6 4 9 6 * 6 * 5 * 6 * 5 9 4 3 8 * 9 * 8 4 8 * 6 6 5 3 5 - - - - - - - - - - 7 3 3

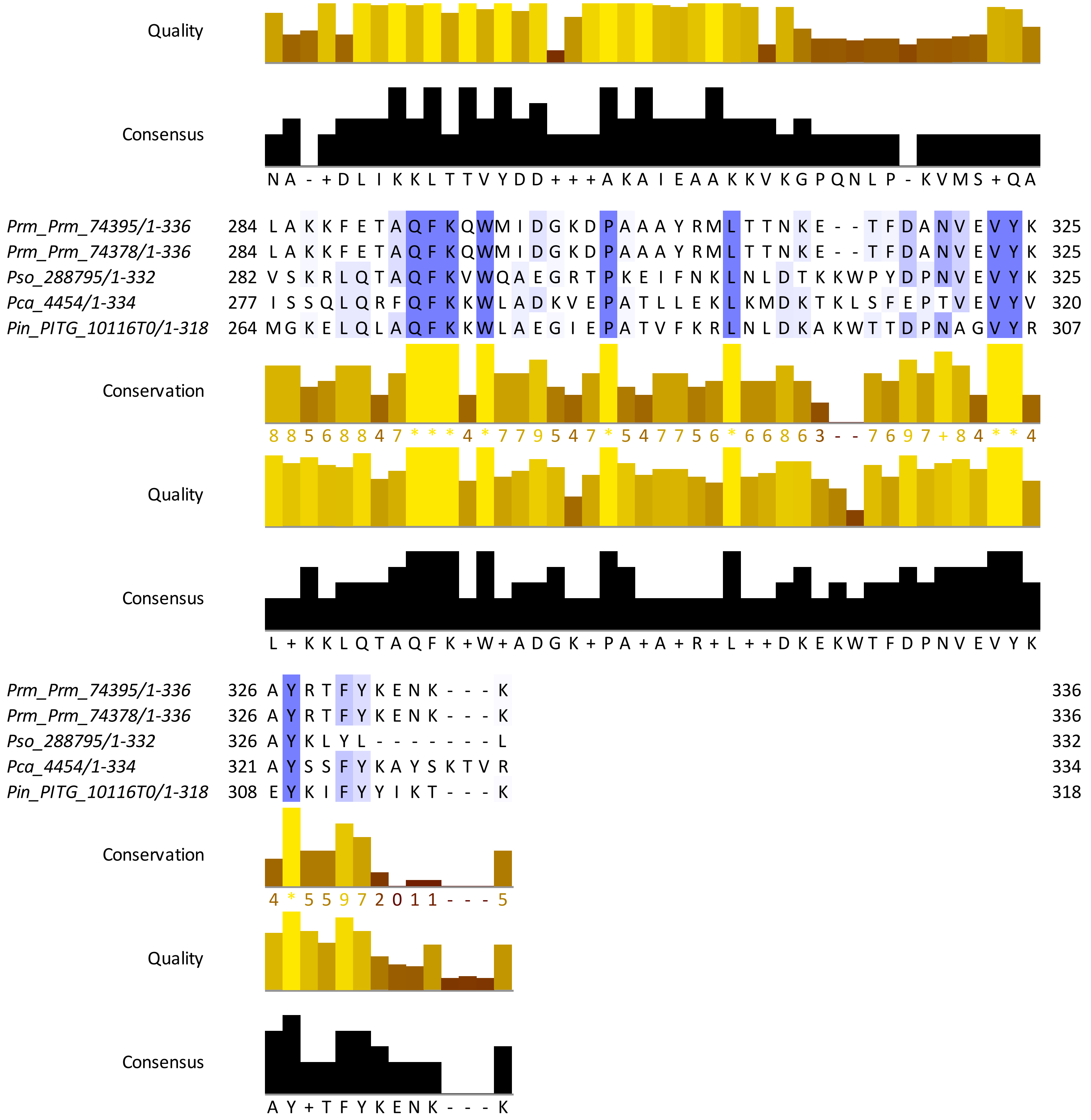
Quality
Consensus
N A - + D L I K K L T T V Y D D + + + A K A I E A A K K V K G P Q N L P - K V M S + Q A
Prm_Prm_74395/1-336 284 L A K K F E T A Q F K Q W M I D G K D P A A A Y R M L T T N K E - - T F D A N V E V Y K 325
Prm_Prm_74378/1-336 284 L A K K F E T A Q F K Q W M I D G K D P A A A Y R M L T T N K E - - T F D A N V E V Y K 325
Pso_288795/1-332 282 V S K R L Q T A Q F K V W Q A E G R T P K E I F N K L N L D T K K W P Y D P N V E V Y K 325
Pca_4454/1-334 277 I S S Q L Q R F Q F K K W L A D K V E P A T L L E K L K M D K T K L S F E P T V E V Y V 320
Pin_PITG_10116T0/1-318 264 M G K E L Q L A Q F K K W L A E G I E P A T V F K R L N L D K A K W T T D P N A G V Y R 307
Conservation
8 8 5 6 8 8 4 7 * * * 4 * 7 7 9 5 4 7 * 5 4 7 7 5 6 * 6 6 8 6 3 - - 7 6 9 7 + 8 4 * * 4
Quality
Consensus
L + K K L Q T A Q F K + W + A D G K + P A + A + R + L + + D K E K W T F D P N V E V Y K
Prm_Prm_74395/1-336 326 A Y R T F Y K E N K - - - K 336
Prm_Prm_74378/1-336 326 A Y R T F Y K E N K - - - K 336
Pso_288795/1-332 326 A Y K L Y L - - - - - - - L 332
Pca_4454/1-334 321 A Y S S F Y K A Y S K T V R 334
Pin_PITG_10116T0/1-318 308 E Y K I F Y Y I K T - - - K 318
Conservation
4 * 5 5 9 7 2 0 1 1 - - - 5
Quality
Consensus
A Y + T F Y K E N K - - - K

## Ortholog 19

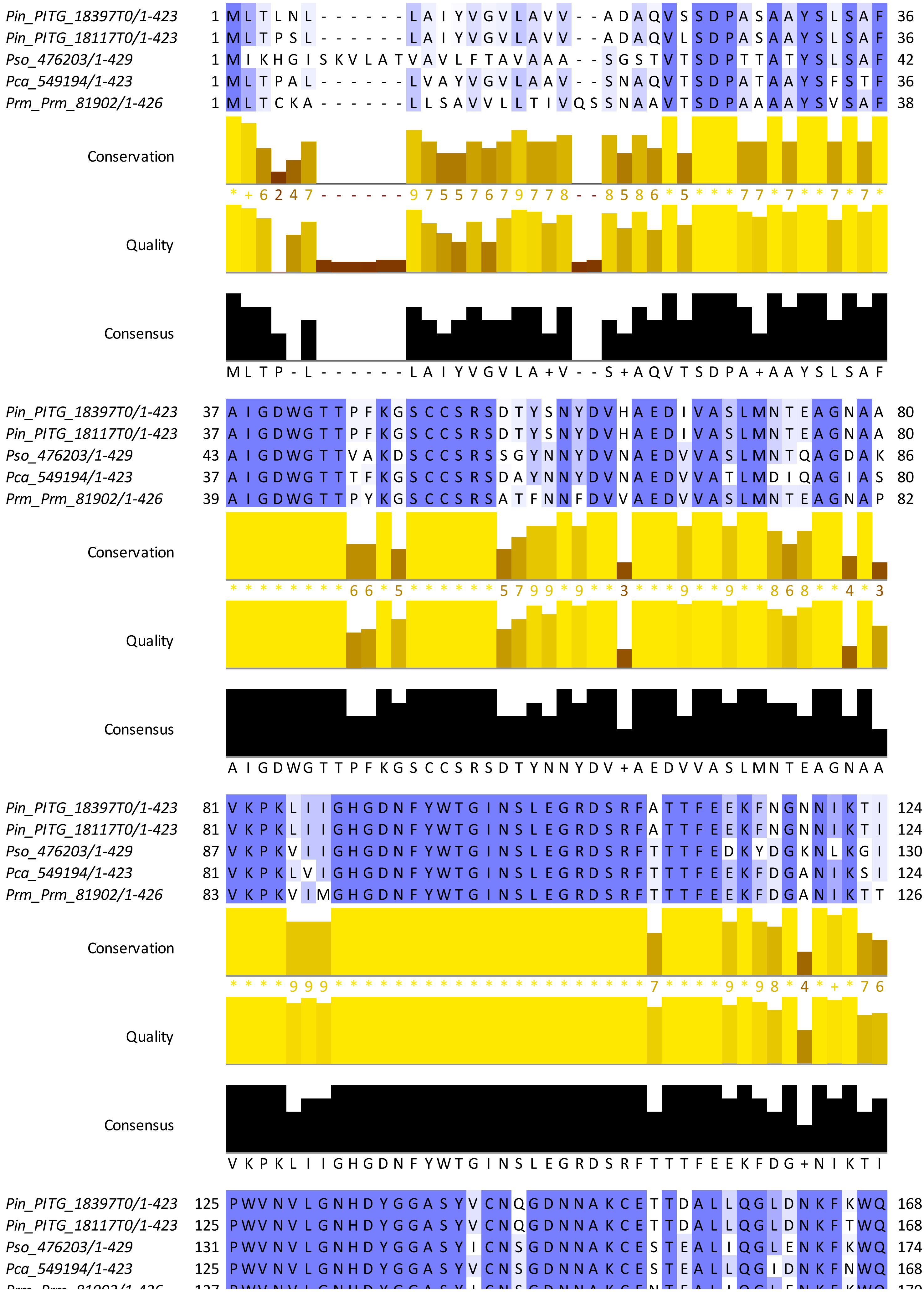
Pin_PITG_18397T0/1-423 1 MLTLNL------LAIYVGVLAVV--ADAQVSSDPASAAYSLSAF 36
Pin_PITG_18117T0/1-423 1 MLTPSL------LAIYVGVLAVV--ADAQVLSDPASAAYSLSAF 36
Pso_476203/1-429 1 MIKHGISKVLATVAVLFTAVAAA--SGSTVTSDPTTATYSLSAF 42
Pca_549194/1-423 1 MLTPAL------LVAYVGVLAAV--SNAQVTSDPATAAYSFSTF 36
Prm_Prm_81902/1-426 1 MLTCKA------LLSAVVLLTIVQSSNAAVTSDPAAAAYSVSAF 38
Conservation
*+6247------97557679778--8586*5***77*7**7*7*
Quality
Consensus
MLTP-L------LAIYVGVLA+V--S+AQVTSDPA+AAYSLSAF
Pin_PITG_18397T0/1-423 37 AIGDWGTTPFKGSCCSRSDTYSNYDVHAEDIVASLMNTEAGNAA 80
Pin_PITG_18117T0/1-423 37 AIGDWGTTPFKGSCCSRSDTYSNYDVHAEDIVASLMNTEAGNAA 80
Pso_476203/1-429 43 AIGDWGTTVAKDSCCSRSSGYNNYDVNAEDVVASLMNTQAGDAK 86
Pca_549194/1-423 37 AIGDWGTTTFKGSCCSRSDAYNNYDVNAEDVVATLMDIQAGIAS 80
Prm_Prm_81902/1-426 39 AIGDWGTTPYKGSCCSRSATFNNFDVVAEDVVASLMNTEAGNAP 82
Conservation
********66*5******5799*9**3***9**9**868**4*3
Quality
Consensus
AIGDWGTTPFKGSCCSRSDTYNNYDV+AEDVVASLMNTEAGNAA
Pin_PITG_18397T0/1-423 81 VKPKLIIGHGDNFYWTGINSLEGRDSRFATTFEEKFNGNNIKTI 124
Pin_PITG_18117T0/1-423 81 VKPKLIIGHGDNFYWTGINSLEGRDSRFATTFEEKFNGNNIKTI 124
Pso_476203/1-429 87 VKPKVIIGHGDNFYWTGINSLEGRDSRFTTTFEDKYDGKNLKGI 130
Pca_549194/1-423 81 VKPKLVIGHGDNFYWTGINSLEGRDSRFTTTFEEKFDGANIKSI 124
Prm_Prm_81902/1-426 83 VKPKVIMGHGDNFYWTGINSLEGRDSRFTTTFEEKFDGANIKTT 126
Conservation
****999*********************7****9*98*4*+*76
Quality
Consensus
VKPKLIIGHGDNFYWTGINSLEGRDSRFTTTFEEKFDG+NIKTI
Pin_PITG_18397T0/1-423 125 PWVNVLGNHDYGGASYVCNQGDNNAKCETTDALLQGLDNKFKWQ 168
Pin_PITG_18117T0/1-423 125 PWVNVLGNHDYGGASYVCNQGDNNAKCETTDALLQGLDNKFTWQ 168
Pso_476203/1-429 131 PWVNVLGNHDYGGASYICNSGDNNAKCESTEALIQGLENKFKWQ 174
Pca_549194/1-423 125 PWVNVLGNHDYGGASYVCNSGDNNAKCESTEALLQGIDNKFNWQ 168

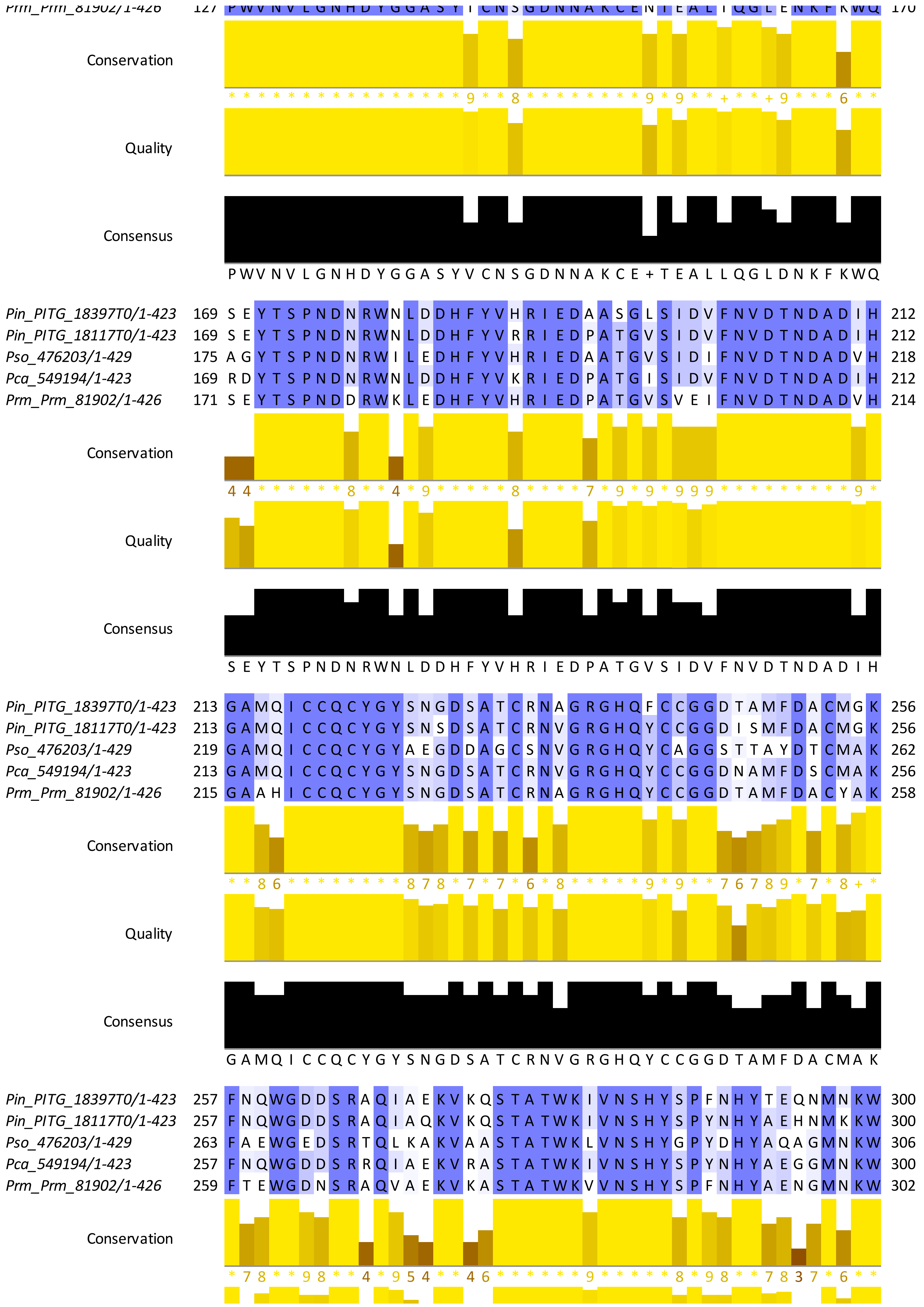

Prm_Prm_81902/1-426 127 PWVNVLGNHDYGGASYICNSGDNNAKCENIEALIQGLENKFKWQ 170
Conservation
* * * * * * * * * * * * * * * * 9 * * 8 * * * * * * * * 9 * 9 * * + * * + 9 * * * 6 * *
Quality
Consensus
PWVNVLGNHDYGGASYVCNSGDNNAKCE+TEALLQGLDNKFKWQ
Pin_PITG_18397T0/1-423 169 SEYTSPNDNRWNLDDHFYVHRIEDAASGLSIDVFNVDTNDADIH 212
Pin_PITG_18117T0/1-423 169 SEYTSPNDNRWNLDDHFYVRRIEDPATGVSIDVFNVDTNDADIH 212
Pso_476203/1-429 175 AGYTSPNDNRWILEDHFYVHRIEDAATGVSIDIFNVDTNDADVH 218
Pca_549194/1-423 169 RDYTSPNDNRWNLDDHFYVKRIEDPATGISIDVFNVDTNDADIH 212
Prm_Prm_81902/1-426 171 SEYTSPNDDRWKLEDHFYVHRIEDPATGVSVEIFNVDTNDADVH 214
Conservation
4 4 * * * * * * 8 * * 4 * 9 * * * * * 8 * * * * 7 * 9 * 9 * 9 9 9 * * * * * * * * * 9 *
Quality
Consensus
SEYTSPNDNRWNLDDHFYVHRIEDPATGVSIDVFNVDTNDADIH
Pin_PITG_18397T0/1-423 213 GAMQICCQCYGYSNGDSATCRNAGRGHQFCCGGDTAMFDACMGK 256
Pin_PITG_18117T0/1-423 213 GAMQICCQCYGYSNSDSATCRNVGRGHQYCCGGDISMFDACMGK 256
Pso_476203/1-429 219 GAMQICCQCYGYAEGDDAGCSNVGRGHQYCAGGSTTAYDTCMAK 262
Pca_549194/1-423 213 GAMQICCQCYGYSNGDSATCRNVGRGHQYCCGGDNAMFDSCMAK 256
Prm_Prm_81902/1-426 215 GAAHICCQCYGYSNGDSATCRNAGRGHQYCCGGDTAMFDACYAK 258
Conservation
* * 8 6 * * * * * * * * 8 7 8 * 7 * 7 * 6 * 8 * * * * * 9 * 9 * * 7 6 7 8 9 * 7 * 8 + *
Quality
Consensus
GAMQICCQCYGYSNGDSATCRNVGRGHQYCCGGDTAMFDACMAK
Pin_PITG_18397T0/1-423 257 FNQWGDDSRAQIAEKVKQSTATWKIVNSHYSPFNHYTEQNMNKW 300
Pin_PITG_18117T0/1-423 257 FNQWGDDSRAQIAQKVKQSTATWKIVNSHYSPYNHYAEHNMKKW 300
Pso_476203/1-429 263 FAEWGEDSRTQLKAKVAASTATWKLVNSHYGPYDHYAQAGMNKW 306
Pca_549194/1-423 257 FNQWGDDSRRQIAEKVRASTATWKIVNSHYSPYNHYAEGGMNKW 300
Prm_Prm_81902/1-426 259 FTEWGDNSRAQVAEKVKASTATWKVVNSHYSPFNHYAENGMNKW 302
Conservation
* 7 8 * * 9 8 * * 4 * 9 5 4 * * 4 6 * * * * * * 9 * * * * * 8 * 9 8 * * 7 8 3 7 * 6 * *

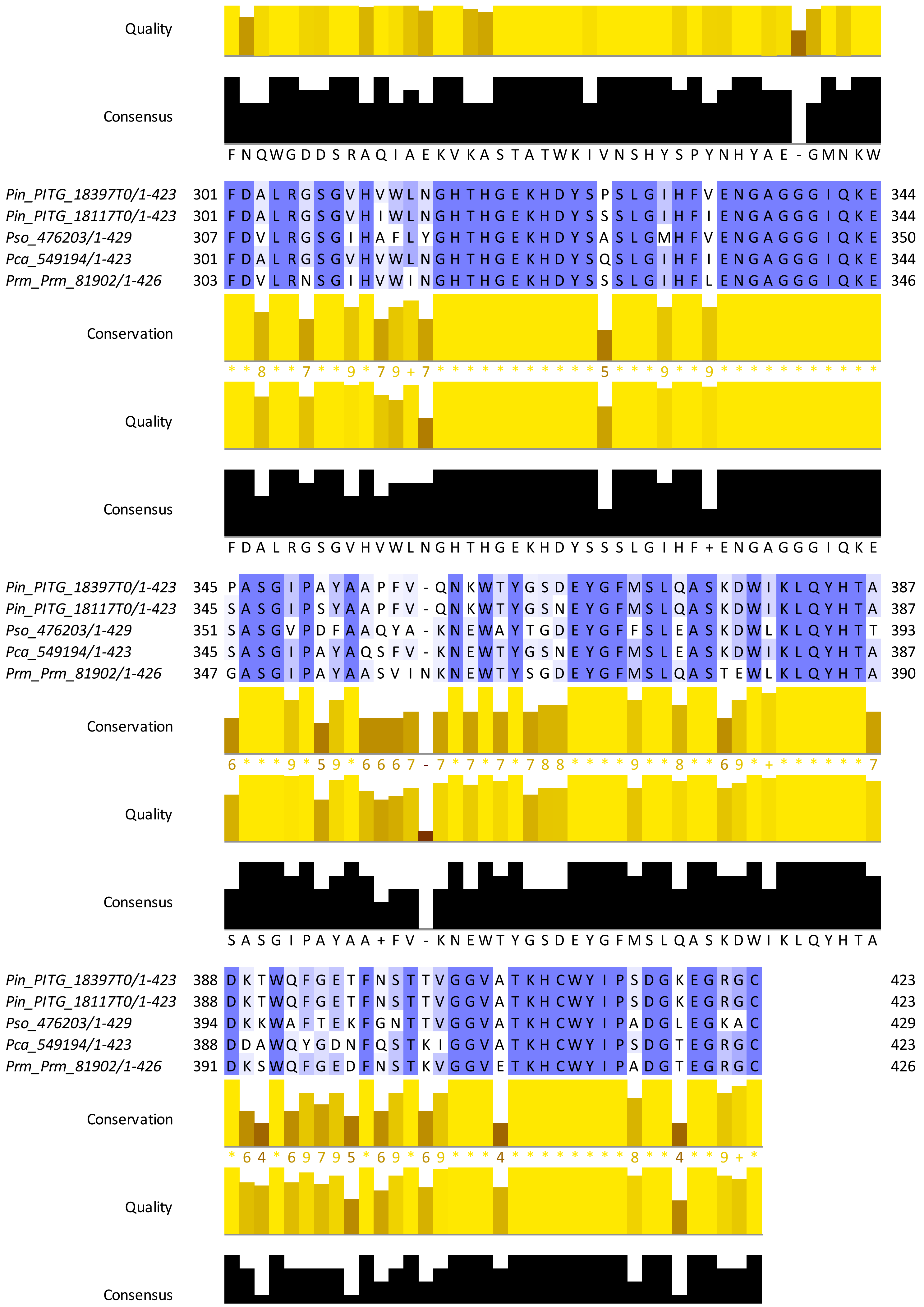
Quality
Consensus
F N Q W G D D S R A Q I A E K V K A S T A T W K I V N S H Y S P Y N H Y A E - G M N K W
Pin_PITG_18397T0/1-423 301 FDALRGSGVHVWLNGHTHGEKHDYSPSLGIHFVENGAGGGIQKE 344
Pin_PITG_18117T0/1-423 301 FDALRGSGVHIWLNGHTHGEKHDYSSSLGIHFIENGAGGGIQKE 344
Pso_476203/1-429 307 FDVLRGSGIHAFLYGHTHGEKHDYSASLGMHFVENGAGGGIQKE 350
Pca_549194/1-423 301 FDALRGSGVHVWLNGHTHGEKHDYSQSLGIHFIENGAGGGIQKE 344
Prm_Prm_81902/1-426 303 FDVLRNSGIHVWINGHTHGEKHDYSSSLGIHFLENGAGGGIQKE 346
Conservation
* * 8 * * 7 * * 9 * 7 9 + 7 * * * * * * * * * * * 5 * * * 9 * * 9 * * * * * * * * * * *
Quality
Consensus
F D A L R G S G V H V W L N G H T H G E K H D Y S S S L G I H F + E N G A G G G I Q K E
Pin_PITG_18397T0/1-423 345 PASGIPAYAAPFV-QNKWTYGSDEYGFMSLQASKDWIKLQYHTA 387
Pin_PITG_18117T0/1-423 345 SASGIPSYAAPFV-QNKWTYGSNEYGFMSLQASKDWIKLQYHTA 387
Pso_476203/1-429 351 SASGVPDFAAQYA-KNEWAYTGDEYGFFSLEASKDWLKLQYHTT 393
Pca_549194/1-423 345 SASGIPAYAQSFV-KNEWTYGSNEYGFMSLEASKDWIKLQYHTA 387
Prm_Prm_81902/1-426 347 GASGIPAYAASVINKNEWTYSGDEYGFMSLQASTEWLKLQYHTA 390
Conservation
6 * * * 9 * 5 9 * 6 6 6 7 - 7 * 7 * 7 * 7 8 8 * * * * 9 * * 8 * * 6 9 * + * * * * * * 7
Quality
Consensus
S A S G I P A Y A A + F V - K N E W T Y G S D E Y G F M S L Q A S K D W I K L Q Y H T A
Pin_PITG_18397T0/1-423 388 DKTWQFGETFNSTTVGGVATKHCWYIPSDGKEGRGC 423
Pin_PITG_18117T0/1-423 388 DKTWQFGETFNSTTVGGVATKHCWYIPSDGKEGRGC 423
Pso_476203/1-429 394 DKKWAFTEKFGNTTVGGVATKHCWYIPADGLEGKAC 429
Pca_549194/1-423 388 DDAWQYGDNFQSTKIGGVATKHCWYIPSDGTEGRGC 423
Prm_Prm_81902/1-426 391 DKSWQFGEDFNSTKVGGVETKHCWYIPADGTEGRGC 426
Conservation
* 6 4 * 6 9 7 9 5 * 6 9 * 6 9 * * * 4 * * * * * * * * * 8 * * 4 * * 9 + *
Quality
Consensus

D K T W Q F G E T F N S T T V G G V A T K H C W Y I P S D G + E G R G C

## Ortholog 20

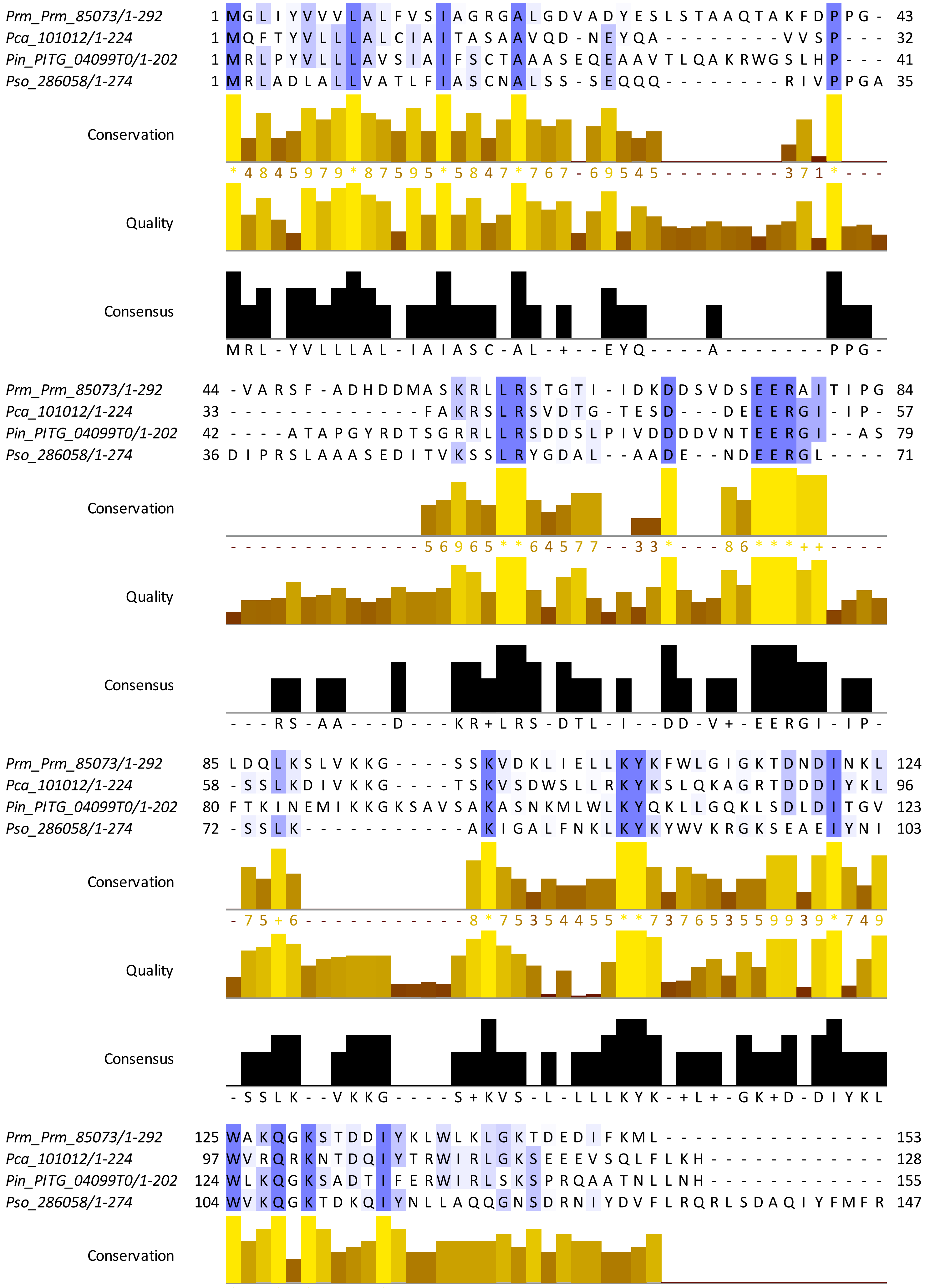
Prm_Prm_85073/1-292 1 M G L I Y V V V L A L F V S I A G R G A L G D V A D Y E S L S T A A Q T A K F D P P G - 43
Pca_101012/1-224 1 M Q F T Y V L L L A L C I A I T A S A A V Q D - N E Y Q A - - - - - - - - V V S P - - - 32
Pin_PITG_04099T0/1-202 1 M R L P Y V L L L A V S I A I F S C T A A A S E Q E A A V T L Q A K R W G S L H P - - - 41
Pso_286058/1-274 1 M R L A D L A L L V A T L F I A S C N A L S S - S E Q Q Q - - - - - - - - R I V P P G A 35
Conservation
* 4 8 4 5 9 7 9 * 8 7 5 9 5 * 5 8 4 7 * 7 6 7 - 6 9 5 4 5 - - - - - - - - 3 7 1 * - - -
Quality
Consensus
M R L - Y V L L L A L - I A I A S C - A L - + - - E Y Q - - - - A - - - - - - - P P G -
Prm_Prm_85073/1-292 44 - V A R S F - A D H D D M A S K R L L R S T G T I - I D K D D S V D S E E R A I T I P G 84
Pca_101012/1-224 33 - - - - - - - - - - - - - F A K R S L R S V D T G - T E S D - - - D E E E R G I - I P - 57
Pin_PITG_04099T0/1-202 42 - - - - A T A P G Y R D T S G R R L L R S D D S L P I V D D D D V N T E E R G I - - A S 79
Pso_286058/1-274 36 D I P R S L A A A S E D I T V K S S L R Y G D A L - - A A D E - - N D E E R G L - - - - 71
Conservation
- - - - - - - - - - - - - 5 6 9 6 5 * * 6 4 5 7 7 - - 3 3 * - - - 8 6 * * * + + - - - -
Quality
Consensus
- - - R S - A A - - - D - - - K R + L R S - D T L - I - - D D - V + - E E R G I - I P -
Prm_Prm_85073/1-292 85 L D Q L K S L V K K G - - - - S S K V D K L I E L L K Y K F W L G I G K T D N D I N K L 124
Pca_101012/1-224 58 - S S L K D I V K K G - - - - T S K V S D W S L L R K Y K S L Q K A G R T D D D I Y K L 96
Pin_PITG_04099T0/1-202 80 F T K I N E M I K K G K S A V S A K A S N K M L W L K Y Q K L L G Q K L S D L D I T G V 123
Pso_286058/1-274 72 - S S L K - - - - - - - - - - - A K I G A L F N K L K Y K Y W V K R G K S E A E I Y N I 103
Conservation
- 7 5 + 6 - - - - - - - - - - - 8 * 7 5 3 5 4 4 5 5 * * 7 3 7 6 5 3 5 5 9 9 3 9 * 7 4 9
Quality
Consensus
- S S L K - - V K K G - - - - S + K V S - L - L L L K Y K - + L + - G K + D - D I Y K L
Prm_Prm_85073/1-292 125 W A K Q G K S T D D I Y K L W L K L G K T D E D I F K M L - - - - - - - - - - - - - - - 153
Pca_101012/1-224 97 W V R Q R K N T D Q I Y T R W I R L G K S E E E V S Q L F L K H - - - - - - - - - - - - 128
Pin_PITG_04099T0/1-202 124 W L K Q G K S A D T I F E R W I R L S K S P R Q A A T N L L N H - - - - - - - - - - - - 155
Pso_286058/1-274 104 W V K Q G K T D K Q I Y N L L A Q Q G N S D R N I Y D V F L R Q R L S D A Q I Y F M F R 147
Conservation

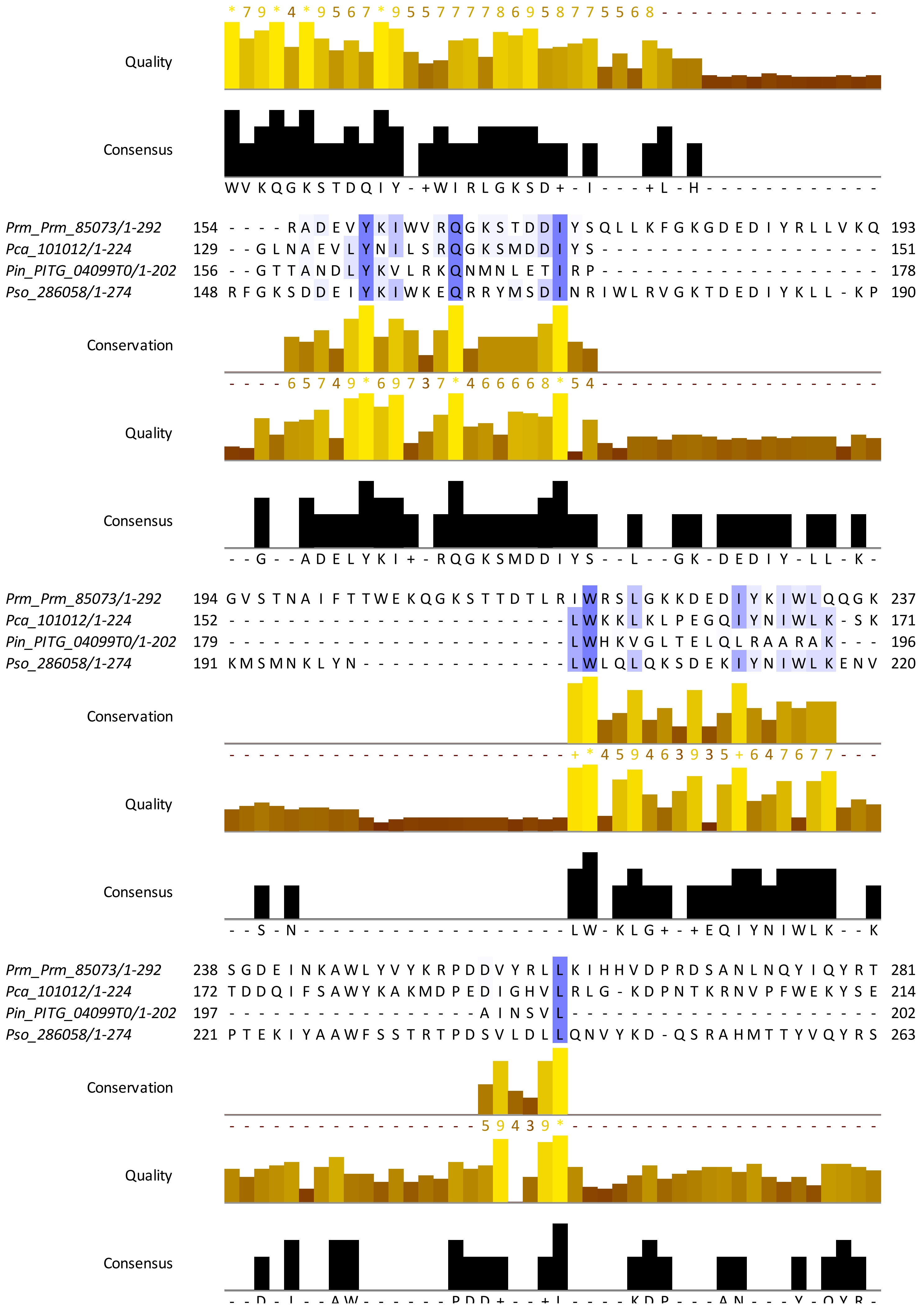
* 7 9 * 4 * 9 5 6 7 * 9 5 5 7 7 7 7 8 6 9 5 8 7 7 5 5 6 8 - - - - - - - - - - - - - - -
Quality
Consensus
W V K Q G K S T D Q I Y - + W I R L G K S D + - I - - - + L - H - - - - - - - - - - - - -
Prm_Prm_85073/1-292 154 - - - - R A D E V Y K I W V R Q G K S T D D I Y S Q L L K F G K G D E D I Y R L L V K Q 193
Pca_101012/1-224 129 - - G L N A E V L Y N I L S R Q G K S M D D I Y S - - - - - - - - - - - - - - - - - - - 151
Pin_PITG_04099T0/1-202 156 - - G T T A N D L Y K V L R K Q N M N L E T I R P - - - - - - - - - - - - - - - - - - - 178
Pso_286058/1-274 148 R F G K S D D E I Y K I W K E Q R R Y M S D I N R I W L R V G K T D E D I Y K L L - K P 190
Conservation
- - - - 6 5 7 4 9 * 6 9 7 3 7 * 4 6 6 6 6 8 * 5 4 - - - - - - - - - - - - - - - - - - -
Quality
Consensus
- - G - - A D E L Y K I + - R Q G K S M D D I Y S - - L - - G K - D E D I Y - L L - K -
Prm_Prm_85073/1-292 194 G V S T N A I F T T W E K Q G K S T T D T L R I W R S L G K K D E D I Y K I W L Q Q G K 237
Pca_101012/1-224 152 - - - - - - - - - - - - - - - - - - - - - - - L W K K L K L P E G Q I Y N I W L K - S K 171
Pin_PITG_04099T0/1-202 179 - - - - - - - - - - - - - - - - - - - - - - - L W H K V G L T E L Q L R A A R A K - - - 196
Pso_286058/1-274 191 K M S M N K L Y N - - - - - - - - - - - - - - L W L Q L Q K S D E K I Y N I W L K E N V 220
Conservation
- - - - - - - - - - - - - - - - - - - - - - - + * 4 5 9 4 6 3 9 3 5 + 6 4 7 6 7 7 - - -
Quality
Consensus
- - S - N - - - - - - - - - - - - - - - - - - L W - K L G + - + E Q I Y N I W L K - - K
Prm_Prm_85073/1-292 238 S G D E I N K A W L Y V Y K R P D D V Y R L L K I H H V D P R D S A N L N Q Y I Q Y R T 281
Pca_101012/1-224 172 T D D Q I F S A W Y K A K M D P E D I G H V L R L G - K D P N T K R N V P F W E K Y S E 214
Pin_PITG_04099T0/1-202 197 - - - - - - - - - - - - - - - - - A I N S V L - - - - - - - - - - - - - - - - - - - - - 202
Pso_286058/1-274 221 P T E K I Y A A W F S S T R T P D S V L D L L Q N V Y K D - Q S R A H M T T Y V Q Y R S 263
Conservation
- - - - - - - - - - - - - - - - - 5 9 4 3 9 * - - - - - - - - - - - - - - - - - - - - -
Quality
Consensus
- - D - I - - A W - - - - - - - P D D + - - + L - - - - - K D P - - - A N - - - Y - Q Y R -

[illegible]

| | | | |
|---|---|---|---|
| *Prm_Prm_85073/1-292* | 282 | A Y N D M L G I S N L | 292 |
| *Pca_101012/1-224* | 215 | F Y R - A K K Y R T S | 224 |
| *Pin_PITG_04099T0/1-202* | | - - - - - - - - - - - | |
| *Pso_286058/1-274* | 264 | Y F N E N Y G I R E W | 274 |

Conservation

Quality

Consensus

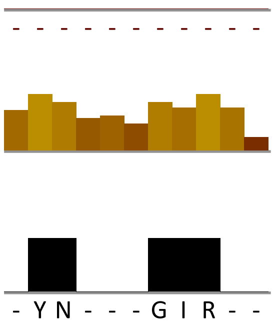

Ortholog 21

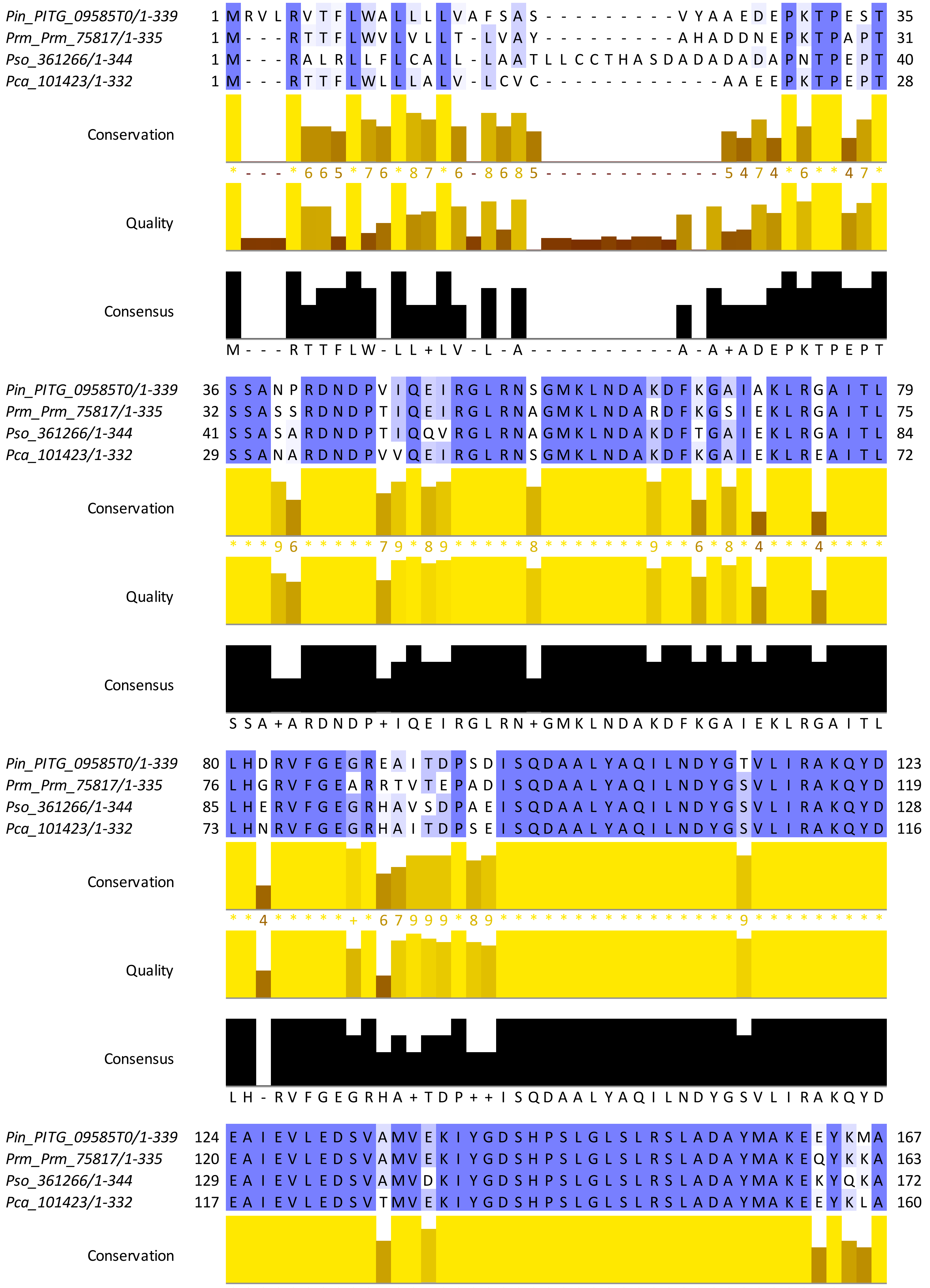
Pin_PITG_09585T0/1-339 1 MRVLRVTFLWALLLLVAFSAS---------VYAAEDEPKTPEST 35
Prm_Prm_75817/1-335 1 M---RTTFLWVLVLLT-LVAY---------AHADDNEPKTPAPT 31
Pso_361266/1-344 1 M---RALRLLFLCALL-LAATLLCCTHASDADADADAPNTPEPT 40
Pca_101423/1-332 1 M---RTTFLWLLLALV-LCVC------------AAEEPKTPEPT 28
Conservation
*---*665*76*87*6-8685-------------5474*6**47*
Quality
Consensus
M---RTTFLW-LL+LV-L-A----------A-A+ADEPKTPEPT
Pin_PITG_09585T0/1-339 36 SSANPRDNDPVIQEIRGLRNSGMKLNDAKDFKGAIAKLRGAITL 79
Prm_Prm_75817/1-335 32 SSASSRDNDPTIQEIRGLRNAGMKLNDARDFKGSIEKLRGAITL 75
Pso_361266/1-344 41 SSASARDNDPTIQQVRGLRNAGMKLNDAKDFTGAIEKLRGAITL 84
Pca_101423/1-332 29 SSANARDNDPVVQEIRGLRNSGMKLNDAKDFKGAIEKLREAITL 72
Conservation
***96*****79*89*****8*******9**6*8*4***4****
Quality
Consensus
SSA+ARDNDP+IQEIRGLRN+GMKLNDAKDFKGAIEKLRGAITL
Pin_PITG_09585T0/1-339 80 LHDRVFGEGREAITDPSDISQDAALYAQILNDYGTVLIRAKQYD 123
Prm_Prm_75817/1-335 76 LHGRVFGEARRTVTEPADISQDAALYAQILNDYGSVLIRAKQYD 119
Pso_361266/1-344 85 LHERVFGEGRHAVSDPAEISQDAALYAQILNDYGSVLIRAKQYD 128
Pca_101423/1-332 73 LHNRVFGEGRHAITDPSEISQDAALYAQILNDYGSVLIRAKQYD 116
Conservation
**4*****+*67999*89***************9**********
Quality
Consensus
LH-RVFGEGRHA+TDP++ISQDAALYAQILNDYGSVLIRAKQYD
Pin_PITG_09585T0/1-339 124 EAIEVLEDSVAMVEKIYGDSHPSLGLSLRSLADAYMAKEEYKMA 167
Prm_Prm_75817/1-335 120 EAIEVLEDSVAMVEKIYGDSHPSLGLSLRSLADAYMAKEQYKKA 163
Pso_361266/1-344 129 EAIEVLEDSVAMVDKIYGDSHPSLGLSLRSLADAYMAKEKYQKA 172
Pca_101423/1-332 117 EAIEVLEDSVTMVEKIYGDSHPSLGLSLRSLADAYMAKEEYKLA 160
Conservation

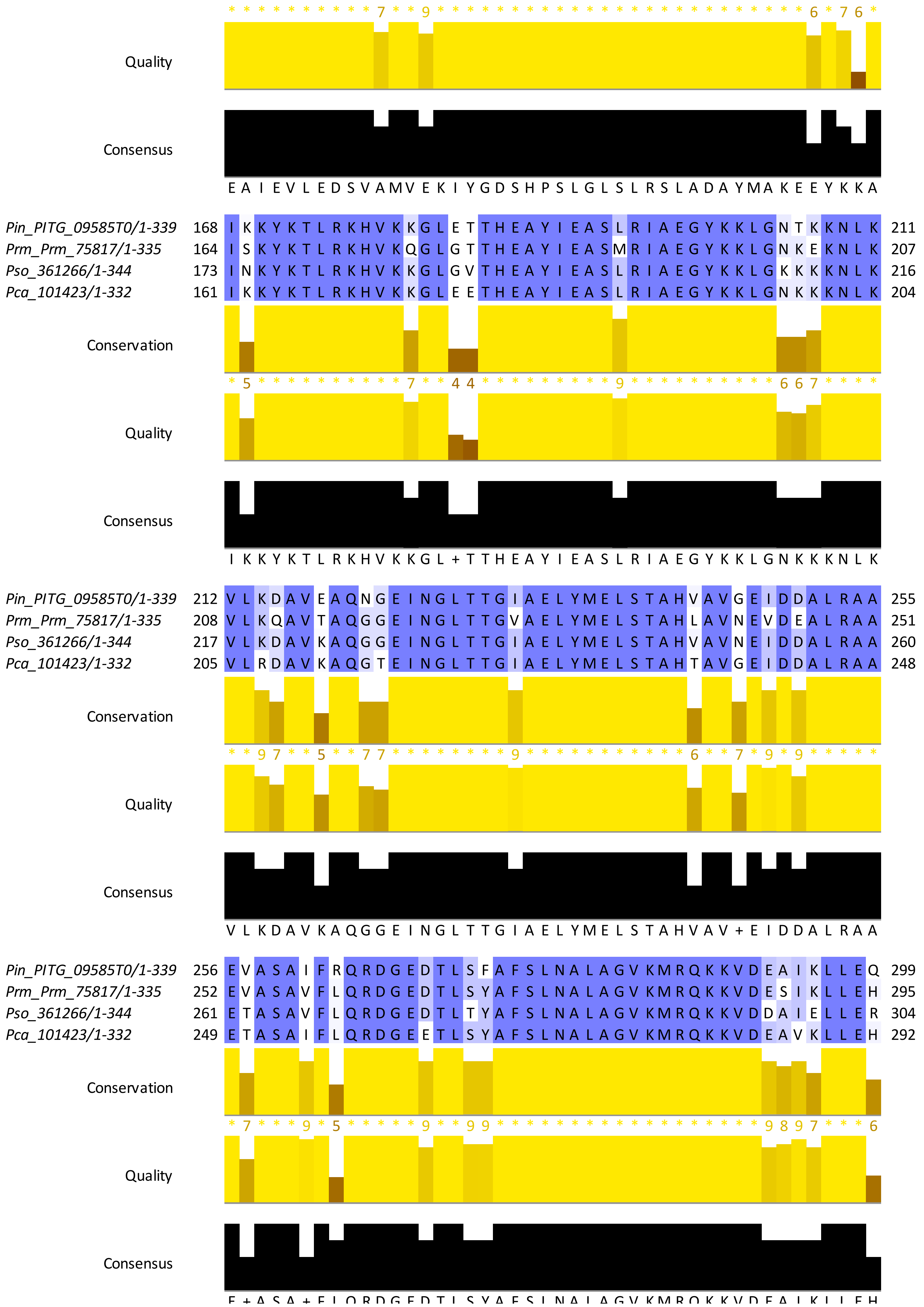

* * * * * * * * * * 7 * * 9 * * * * * * * * * * * * * * * * * * * * * * * * 6 * 7 6 *
Quality
Consensus
EAIEVLEDSVAMVEKIYGDSHPSLGLSLRSLADAYMAKEEYKKA
Pin_PITG_09585T0/1-339 168 IKKYKTLRKHVKKGLETTHEAYIEASLRIAEGYKKLGNTKKNLK 211
Prm_Prm_75817/1-335 164 ISKYKTLRKHVKQGLGTTHEAYIEASMRIAEGYKKLGNKEKNLK 207
Pso_361266/1-344 173 INKYKTLRKHVKKGLGVTHEAYIEASLRIAEGYKKLGKKKKNLK 216
Pca_101423/1-332 161 IKKYKTLRKHVKKGLEETHEAYIEASLRIAEGYKKLGNKKKNLK 204
Conservation
* 5 * * * * * * * * * * 7 * * 4 4 * * * * * * * * * 9 * * * * * * * * * * 6 6 7 * * * *
Quality
Consensus
IKKYKTLRKHVKKGL+TTHEAYIEASLRIAEGYKKLGNKKKNLK
Pin_PITG_09585T0/1-339 212 VLKDAVEAQNGEINGLTTGIAELYMELSTAHVAVGEIDDALRAA 255
Prm_Prm_75817/1-335 208 VLKQAVTAQGGEINGLTTGVAELYMELSTAHLAVNEVDEALRAA 251
Pso_361266/1-344 217 VLKDAVKAQGGEINGLTTGIAELYMELSTAHVAVNEIDDALRAA 260
Pca_101423/1-332 205 VLRDAVKAQGTEINGLTTGIAELYMELSTAHTAVGEIDDALRAA 248
Conservation
* * 9 7 * * 5 * * 7 7 * * * * * * * * 9 * * * * * * * * * * * 6 * * 7 * 9 * 9 * * * * *
Quality
Consensus
VLKDAVKAQGGEINGLTTGIAELYMELSTAHVAV+EIDDALRAA
Pin_PITG_09585T0/1-339 256 EVASAIFRQRDGEDTLSFAFSLNALAGVKMRQKKVDEAIKLLEQ 299
Prm_Prm_75817/1-335 252 EVASAVFLQRDGEDTLSYAFSLNALAGVKMRQKKVDESIKLLEH 295
Pso_361266/1-344 261 ETASAVFLQRDGEDTLTYAFSLNALAGVKMRQKKVDDAIELLER 304
Pca_101423/1-332 249 ETASAIFLQRDGEETLSYAFSLNALAGVKMRQKKVDEAVKLLEH 292
Conservation
* 7 * * * 9 * 5 * * * * * 9 * * 9 9 * * * * * * * * * * * * * * * * * * 9 8 9 7 * * * 6
Quality
Consensus
E+ASA+FLQRDGEDTLSYAFSLNALAGVKMRQKKVDEAIKLLEH

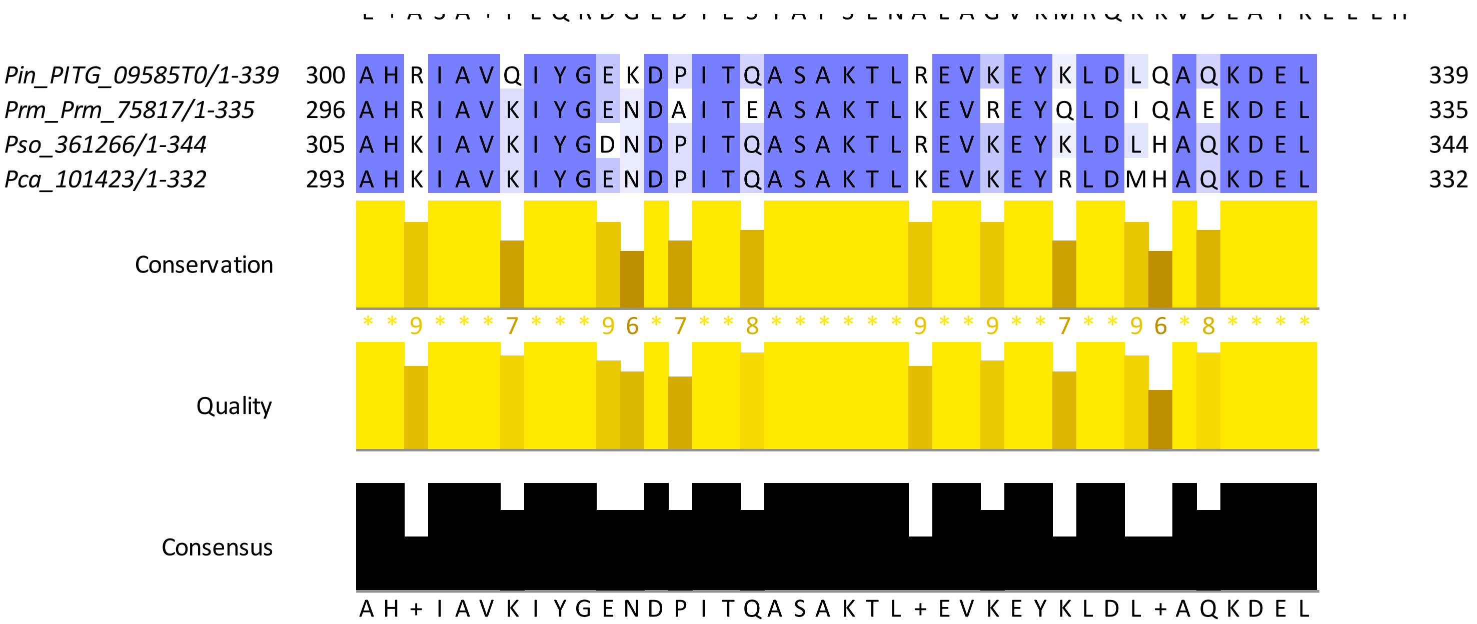
Pin_PITG_09585T0/1-339 300 AHRIAVQIYGEKDPITQASAKTLREVKEYKLDLQAQKDEL 339
Prm_Prm_75817/1-335 296 AHRIAVKIYGENDAITEASAKTLKEVREYQLDIQAEKDEL 335
Pso_361266/1-344 305 AHKIAVKIYGDNDPITQASAKTLREVKEYKLDLHAQKDEL 344
Pca_101423/1-332 293 AHKIAVKIYGENDPITQASAKTLKEVKEYRLDMHAQKDEL 332
Conservation
* * 9 * * * 7 * * * 9 6 * 7 * * 8 * * * * * * 9 * * 9 * * 7 * * 9 6 * 8 * * * *
Quality
Consensus
AH+IAVKIYGENDPITQASAKTL+EVKEYKLDL+AQKDEL

## Ortholog 22

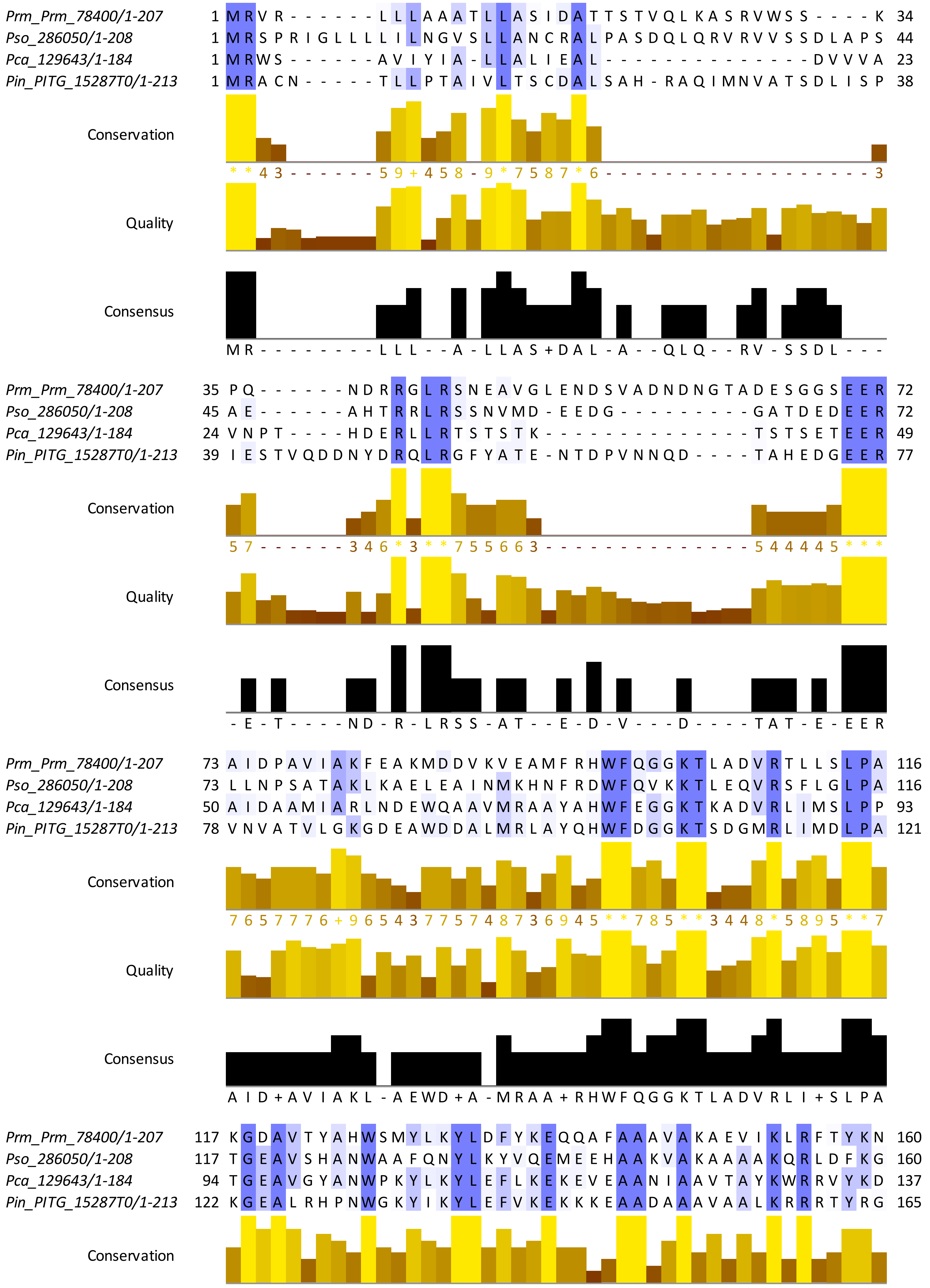
Prm_Prm_78400/1-207 1 MRVR------LLLAAATLLASIDATTSTVQLKASRVWSS----K 34
Pso_286050/1-208 1 MRSPRIGLLLLILNGVSLLANCRALPASDQLQRVRVVSSDLAPS 44
Pca_129643/1-184 1 MRWS------AVIYIA-LLALIEAL--------------DVVVA 23
Pin_PITG_15287T0/1-213 1 MRACN-----TLLPTAIVLTSCDALSAH-RAQIMNVATSDLISP 38
Conservation
* * 4 3 - - - - - - 5 9 + 4 5 8 - 9 * 7 5 8 7 * 6 - - - - - - - - - - - - - - - - - - 3
Quality
Consensus
MR--------LLL--A-LLAS+DAL-A--QLQ--RV-SSDL---
Prm_Prm_78400/1-207 35 PQ------NDRRGLRSNEAVGLENDSVADNDNGTADESGGSEER 72
Pso_286050/1-208 45 AE------AHTRRLRSSNVMD-EEDG---------GATDEDEER 72
Pca_129643/1-184 24 VNPT----HDERLLRTSTSTK--------------TSTSETEER 49
Pin_PITG_15287T0/1-213 39 IESTVQDDNYDRQLRGFYATE-NTDPVNNQD----TAHEDGEER 77
Conservation
5 7 - - - - - - 3 4 6 * 3 * * 7 5 5 6 6 3 - - - - - - - - - - - - - - 5 4 4 4 4 5 * * *
Quality
Consensus
-E-T----ND-R-LRSS-AT--E-D-V---D----TAT-E-EER
Prm_Prm_78400/1-207 73 AIDPAVIAKFEAKMDDVKVEAMFRHWFQGGKTLADVRTLLSLPA 116
Pso_286050/1-208 73 LLNPSATAKLKAELEAINMKHNFRDWFQVKKTLEQVRSFLGLPA 116
Pca_129643/1-184 50 AIDAAMIARLNDEWQAAVMRAAYAHWFEGGKTKADVRLIMSLPP 93
Pin_PITG_15287T0/1-213 78 VNVATVLGKGDEAWDDALMRLAYQHWFDGGKTSDGMRLIMDLPA 121
Conservation
7 6 5 7 7 7 6 + 9 6 5 4 3 7 7 5 7 4 8 7 3 6 9 4 5 * * 7 8 5 * * 3 4 4 8 * 5 8 9 5 * * 7
Quality
Consensus
AID+AVIAKL-AEWD+A-MRAA+RHWFQGGKTLADVRLI+SLPA
Prm_Prm_78400/1-207 117 KGDAVTYAHWSMYLKYLDFYKEQQAFAAAVAKAEVIKLRFTYKN 160
Pso_286050/1-208 117 TGEAVSHANWAAFQNYLKYVQEMEEHAAKVAKAAAAKQRLDFKG 160
Pca_129643/1-184 94 TGEAVGYANWPKYLKYLEFLKEKEVEAANIAAVTAYKWRRVYKD 137
Pin_PITG_15287T0/1-213 122 KGEALRHPNWGKYIKYLEFVKEKKKEAADAAAVAALKRRRTYRG 165
Conservation

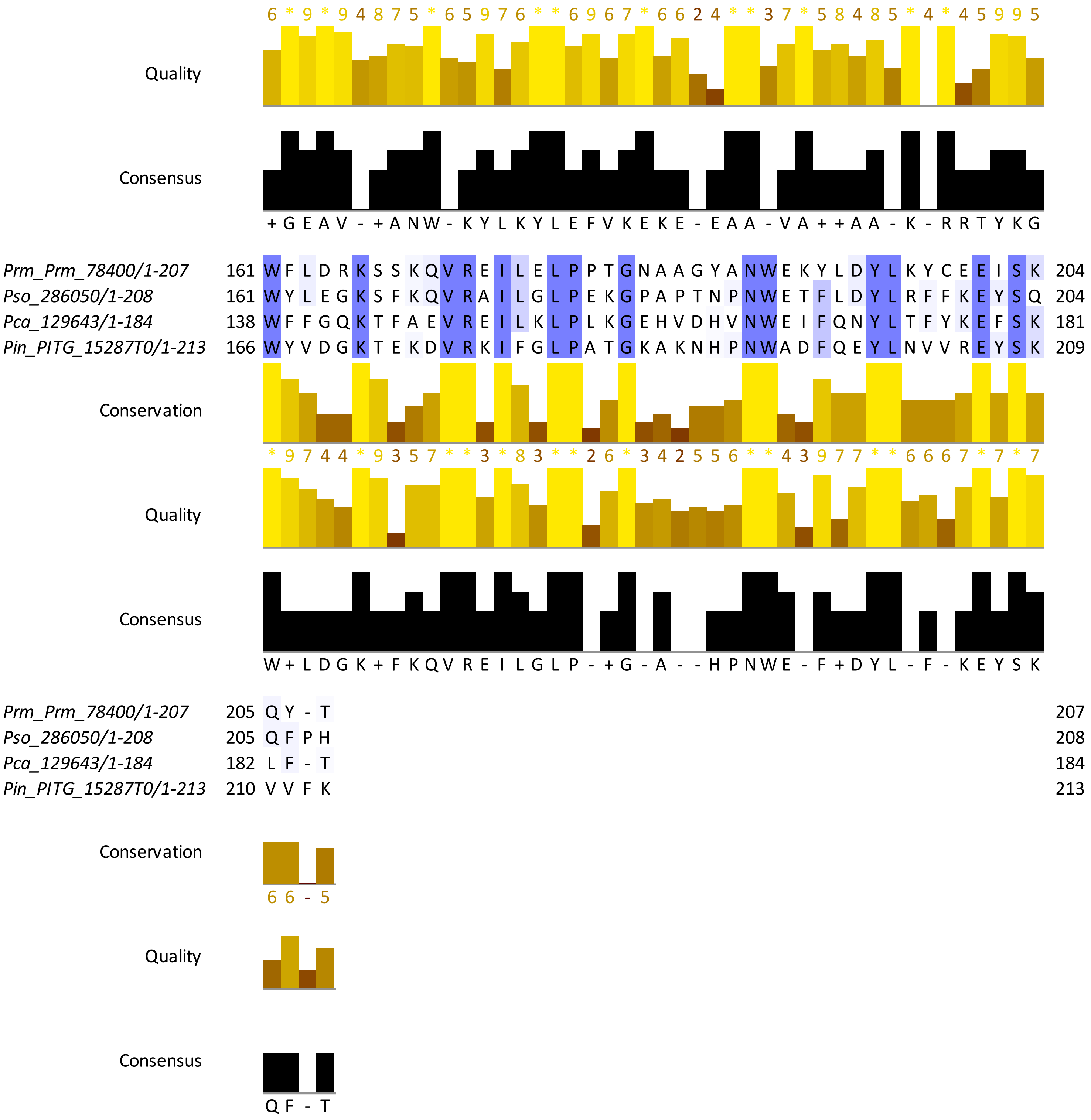
6 * 9 * 9 4 8 7 5 * 6 5 9 7 6 * * 6 9 6 7 * 6 6 2 4 * * 3 7 * 5 8 4 8 5 * 4 * 4 5 9 9 5
Quality
Consensus
+ G E A V - + A N W - K Y L K Y L E F V K E K E - E A A - V A + + A A - K - R R T Y K G
Prm_Prm_78400/1-207 161 W F L D R K S S K Q V R E I L E L P P T G N A A G Y A N W E K Y L D Y L K Y C E E I S K 204
Pso_286050/1-208 161 W Y L E G K S F K Q V R A I L G L P E K G P A P T N P N W E T F L D Y L R F F K E Y S Q 204
Pca_129643/1-184 138 W F F G Q K T F A E V R E I L K L P L K G E H V D H V N W E I F Q N Y L T F Y K E F S K 181
Pin_PITG_15287T0/1-213 166 W Y V D G K T E K D V R K I F G L P A T G K A K N H P N W A D F Q E Y L N V V R E Y S K 209
Conservation
* 9 7 4 4 * 9 3 5 7 * * 3 * 8 3 * * 2 6 * 3 4 2 5 5 6 * * 4 3 9 7 7 * * 6 6 6 7 * 7 * 7
Quality
Consensus
W + L D G K + F K Q V R E I L G L P - + G - A - - H P N W E - F + D Y L - F - K E Y S K
Prm_Prm_78400/1-207 205 Q Y - T 207
Pso_286050/1-208 205 Q F P H 208
Pca_129643/1-184 182 L F - T 184
Pin_PITG_15287T0/1-213 210 V V F K 213
Conservation
6 6 - 5
Quality
Consensus
Q F - T

# Ortholog 23

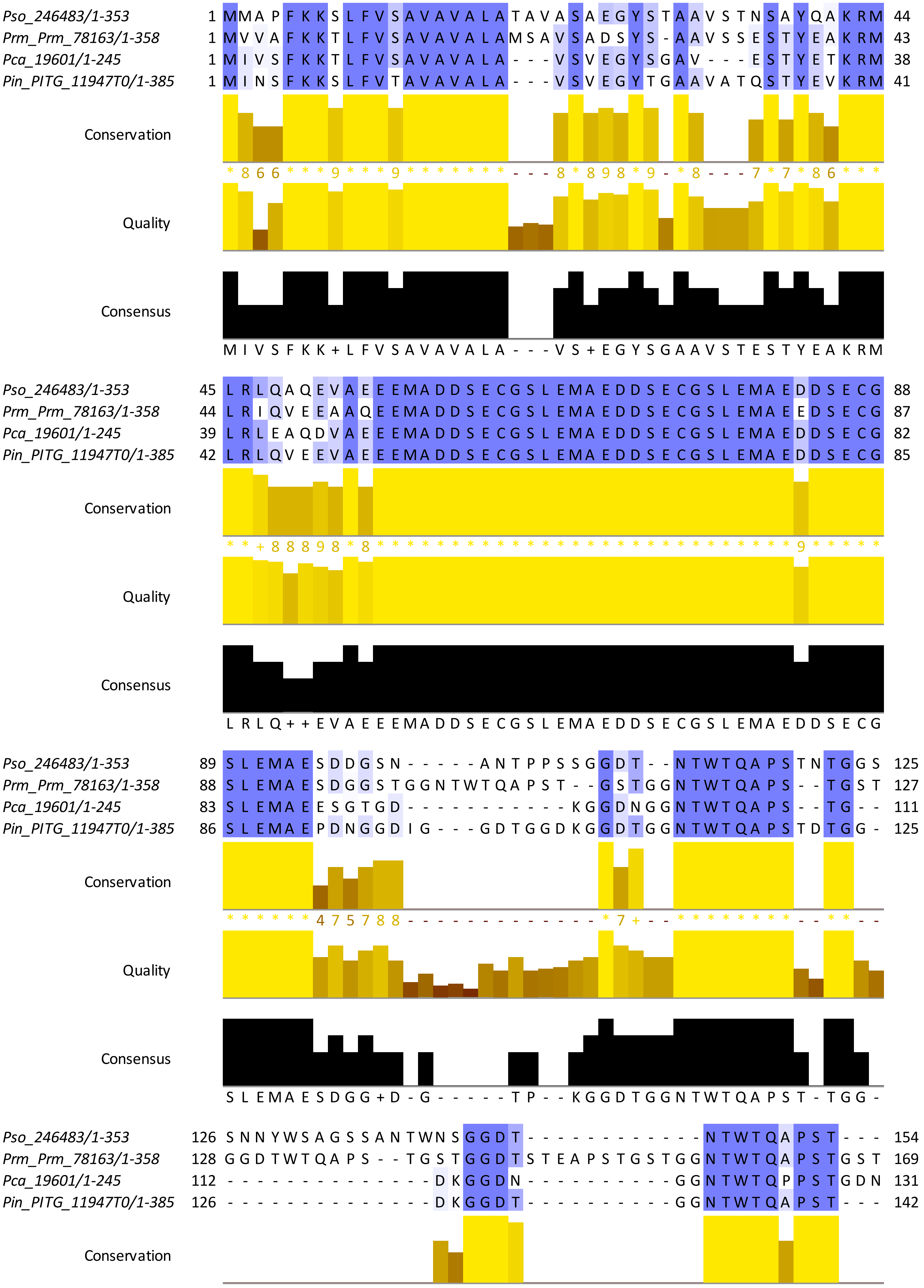
Pso_246483/1-353 1 MMAPFKKSLFVSAVAVALATAVASAEGYSTAAVSTNSAYQAKRM 44
Prm_Prm_78163/1-358 1 MVVAFKKTLFVSAVAVALAMSAVSADSYS-AAVSSESTYEAKRM 43
Pca_19601/1-245 1 MIVSFKKTLFVSAVAVALA---VSVEGYSGAV---ESTYETKRM 38
Pin_PITG_11947T0/1-385 1 MINSFKKSLFVTAVAVALA---VSVEGYTGAAVATQSTYEVKRM 41
Conservation
*866***9***9*******---8*898*9-*8---7*7*86***
Quality
Consensus
MIVSFKK+LFVSAVAVALA---VS+EGYSGAAVSTESTYEAKRM
Pso_246483/1-353 45 LRLQAQEVAEEEMADDSECGSLEMAEDDSECGSLEMAEDDSECG 88
Prm_Prm_78163/1-358 44 LRIQVEEAAQEEMADDSECGSLEMAEDDSECGSLEMAEEDSECG 87
Pca_19601/1-245 39 LRLEAQDVAEEEMADDSECGSLEMAEDDSECGSLEMAEDDSECG 82
Pin_PITG_11947T0/1-385 42 LRLQVEEVAEEEMADDSECGSLEMAEDDSECGSLEMAEDDSECG 85
Conservation
**+88898*8****************************9*****
Quality
Consensus
LRLQ++EVAEEEMADDSECGSLEMAEDDSECGSLEMAEDDSECG
Pso_246483/1-353 89 SLEMAESDDGSN-----ANTPPSSGGDT--NTWTQAPSTNTGGS 125
Prm_Prm_78163/1-358 88 SLEMAESDGGSTGGNTWTQAPST--GSTGGNTWTQAPS--TGST 127
Pca_19601/1-245 83 SLEMAEESGTGD-----------KGGDNGGNTWTQAPS--TG-- 111
Pin_PITG_11947T0/1-385 86 SLEMAEPDNGGDIG---GDTGGDKGGDTGGNTWTQAPSTDTGG- 125
Conservation
******475788-------------*7+--********--**--
Quality
Consensus
SLEMAESDGG+D-G-----TP--KGGDTGGNTWTQAPST-TGG-
Pso_246483/1-353 126 SNNYWSAGSSANTWNSGGDT------------NTWTQAPST--- 154
Prm_Prm_78163/1-358 128 GGDTWTQAPS--TGSTGGDTSTEAPSTGSTGGNTWTQAPSTGST 169
Pca_19601/1-245 112 --------------DKGGDN----------GGNTWTQPPSTGDN 131
Pin_PITG_11947T0/1-385 126 --------------DKGGDT----------GGNTWTQAPST--- 142
Conservation

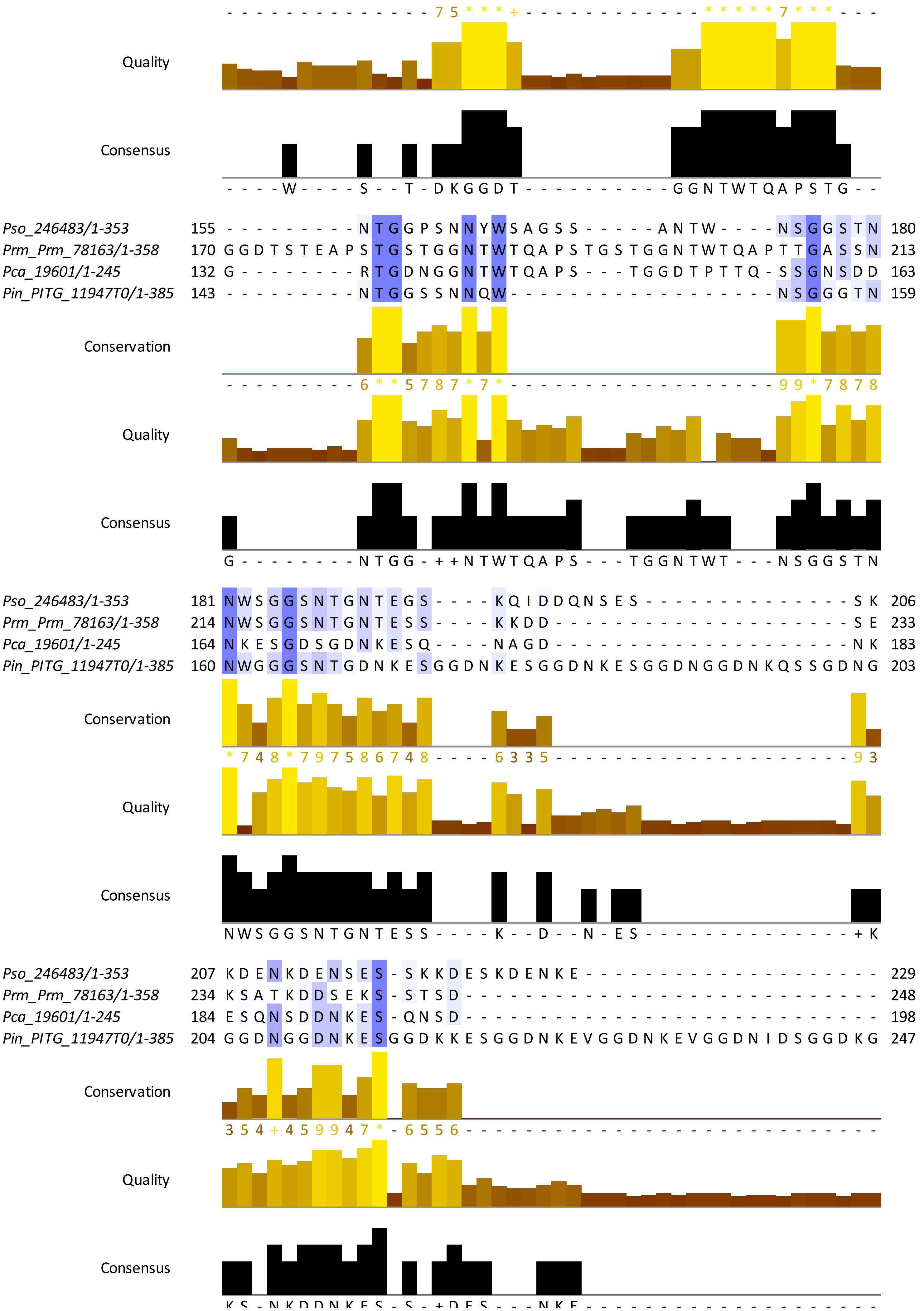

Quality
Consensus
- - - - W - - - - S - - T - D K G G D T - - - - - - - - - - G G N T W T Q A P S T G - -
Pso_246483/1-353 155 - - - - - - - - - N T G G P S N N Y W S A G S S - - - - - A N T W - - - - N S G G S T N 180
Prm_Prm_78163/1-358 170 G G D T S T E A P S T G S T G G N T W T Q A P S T G S T G G N T W T Q A P T T G A S S N 213
Pca_19601/1-245 132 G - - - - - - - - R T G D N G G N T W T Q A P S - - - T G G D T P T T Q - S S G N S D D 163
Pin_PITG_11947T0/1-385 143 - - - - - - - - - N T G G S S N N Q W - - - - - - - - - - - - - - - - - - N S G G G T N 159
Conservation
Quality
Consensus
G - - - - - - - - N T G G - + + N T W T Q A P S - - - T G G N T W T - - - N S G G S T N
Pso_246483/1-353 181 N W S G G S N T G N T E G S - - - - K Q I D D Q N S E S - - - - - - - - - - - - - - S K 206
Prm_Prm_78163/1-358 214 N W S G G S N T G N T E S S - - - - K K D D - - - - - - - - - - - - - - - - - - - - S E 233
Pca_19601/1-245 164 N K E S G D S G D N K E S Q - - - - N A G D - - - - - - - - - - - - - - - - - - - - N K 183
Pin_PITG_11947T0/1-385 160 N W G G G S N T G D N K E S G G D N K E S G G D N K E S G G D N G G D N K Q S S G D N G 203
Conservation
Quality
Consensus
N W S G G S N T G N T E S S - - - - K - - D - - N - E S - - - - - - - - - - - - - - + K
Pso_246483/1-353 207 K D E N K D E N S E S - S K K D E S K D E N K E - - - - - - - - - - - - - - - - - - - - 229
Prm_Prm_78163/1-358 234 K S A T K D D S E K S - S T S D - - - - - - - - - - - - - - - - - - - - - - - - - - - - 248
Pca_19601/1-245 184 E S Q N S D D N K E S - Q N S D - - - - - - - - - - - - - - - - - - - - - - - - - - - - 198
Pin_PITG_11947T0/1-385 204 G G D N G G D N K E S G G D K K E S G G D N K E V G G D N K E V G G D N I D S G G D K G 247
Conservation
Quality
Consensus
K S - N K D D N K E S - S - + D E S - - - N K E - - - - - - - - - - - - - - - - - - - -

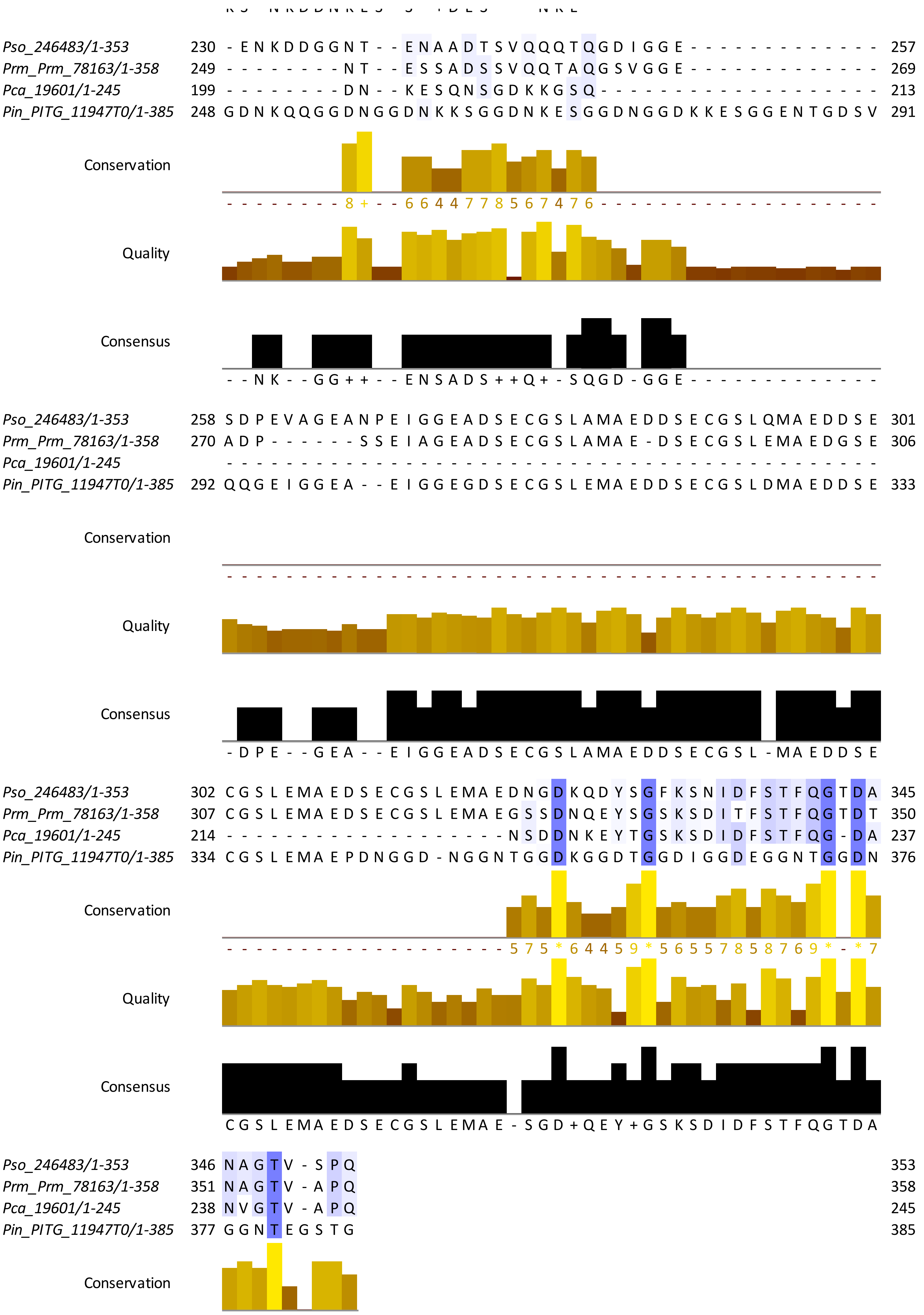
Pso_246483/1-353 230 -ENKDDGGNT--ENAADTSVQQQTQGDIGGE-------------- 257
Prm_Prm_78163/1-358 249 --------NT--ESSADSSVQQTAQGSVGGE-------------- 269
Pca_19601/1-245 199 --------DN--KESQNSGDKKGSQ-------------------- 213
Pin_PITG_11947T0/1-385 248 GDNKQQGGDNGGDNKKSGGDNKESGGDNGGDKKESGGENTGDSV 291
Conservation
--------8+--6644778567476--------------------
Quality
Consensus
--NK--GG++--ENSADS++Q+-SQGD-GGE--------------
Pso_246483/1-353 258 SDPEVAGEANPEIGGEADSECGSLAMAEDDSECGSLQMAEDDSE 301
Prm_Prm_78163/1-358 270 ADP------SSEIAGEADSECGSLAMAE-DSECGSLEMAEDGSE 306
Pca_19601/1-245 --------------------------------------------
Pin_PITG_11947T0/1-385 292 QQGEIGGEA--EIGGEGDSECGSLEMAEDDSECGSLDMAEDDSE 333
Conservation
--------------------------------------------
Quality
Consensus
-DPE--GEA--EIGGEADSECGSLAMAEDDSECGSL-MAEDDSE
Pso_246483/1-353 302 CGSLEMAEDSECGSLEMAEDNGDKQDYSGFKSNIDFSTFQGTDA 345
Prm_Prm_78163/1-358 307 CGSLEMAEDSECGSLEMAEGSSDNQEYSGSKSDITFSTFQGTDT 350
Pca_19601/1-245 214 -------------------NSDDNKEYTGSKSDIDFSTFQG-DA 237
Pin_PITG_11947T0/1-385 334 CGSLEMAEPDNGGD-NGGNTGGDKGGDTGGDIGGDEGGNTGGDN 376
Conservation
-------------------575*64459*56557858769*-*7
Quality
Consensus
CGSLEMAEDSECGSLEMAE-SGD+QEY+GSKSDIDFSTFQGTDA
Pso_246483/1-353 346 NAGTV-SPQ 353
Prm_Prm_78163/1-358 351 NAGTV-APQ 358
Pca_19601/1-245 238 NVGTV-APQ 245
Pin_PITG_11947T0/1-385 377 GGNTEGSTG 385
Conservation

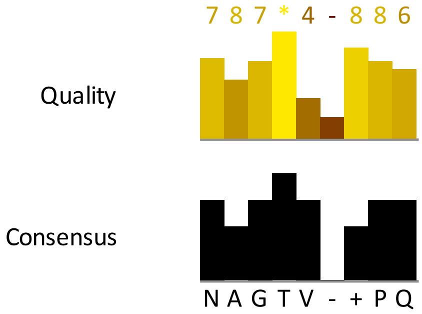

7 8 7 * 4 - 8 8 6
Quality
Consensus
N A G T V - + P Q

Ortholog 24

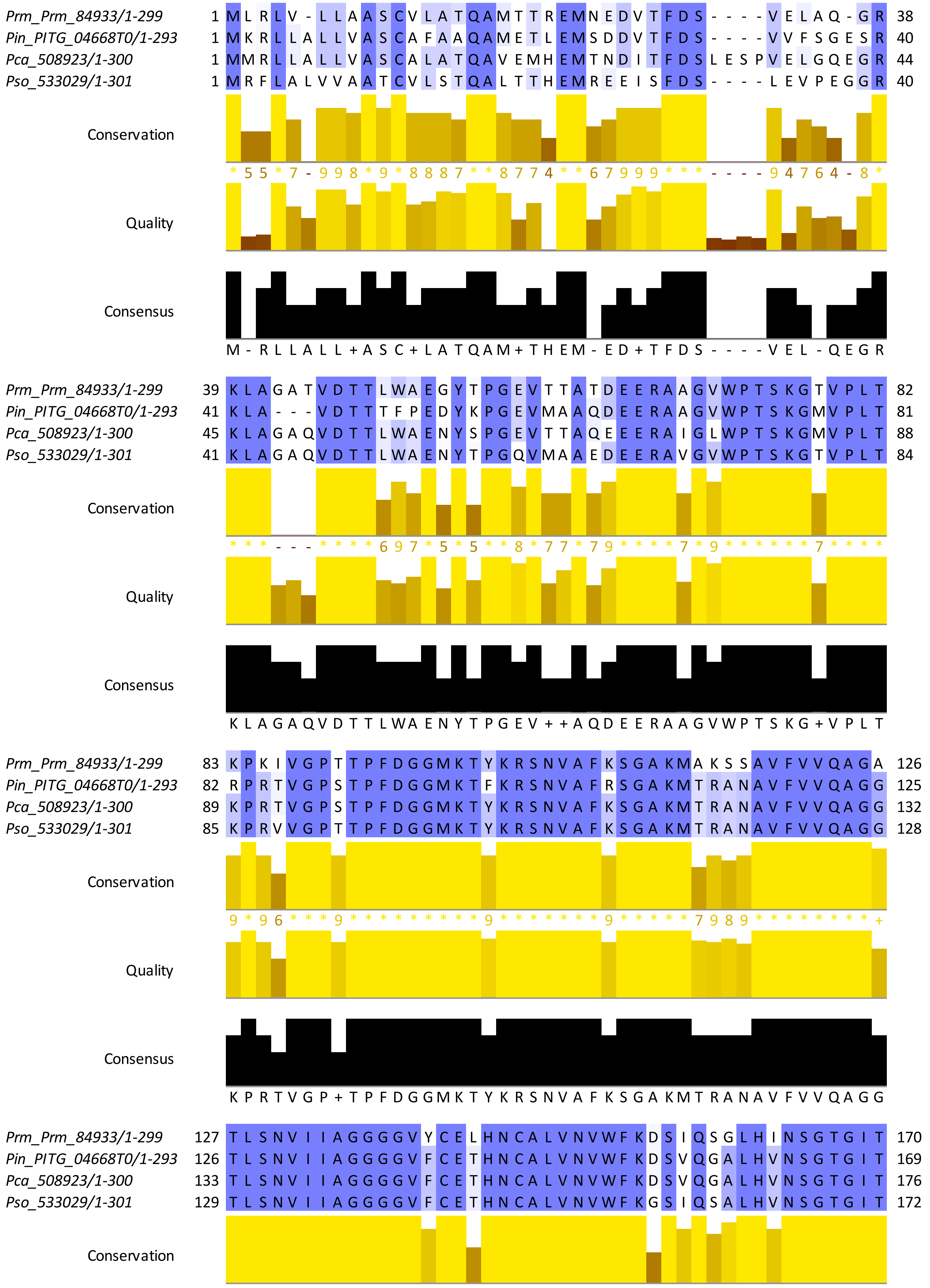
Prm_Prm_84933/1-299 1 MLRLV-LLAASCVLATQAMTTREMNEDVTFDS----VELAQ-GR 38
Pin_PITG_04668T0/1-293 1 MKRLLALLVASCAFAAQAMETLEMSDDVTFDS----VVFSGESR 40
Pca_508923/1-300 1 MMRLLALLVASCALATQAVEMHEMTNDITFDSLESPVELGQEGR 44
Pso_533029/1-301 1 MRFLALVVAATCVLSTQALTTHEMREEISFDS----LEVPEGGR 40
Conservation
* 5 5 * 7 - 9 9 8 * 9 * 8 8 8 7 * * 8 7 7 4 * * 6 7 9 9 * * * - - - - 9 4 7 6 4 - 8 *
Quality
Consensus
M-RLLALL+ASC+LATQAM+THEM-ED+TFDS----VEL-QEGR
Prm_Prm_84933/1-299 39 KLAGATVDTTLWAEGYTPGEVTTATDEERAAGVWPTSKGTVPLT 82
Pin_PITG_04668T0/1-293 41 KLA---VDTTTFPEDYKPGEVMAAQDEERAAGVWPTSKGMVPLT 81
Pca_508923/1-300 45 KLAGAQVDTTLWAENYSPGEVTTAQEEERAIGLWPTSKGMVPLT 88
Pso_533029/1-301 41 KLAGAQVDTTLWAENYTPGQVMAAEDEERAVGVWPTSKGTVPLT 84
Conservation
* * * - - - * * * * 6 9 7 * 5 * 5 * * 8 * 7 7 * 7 9 * * * * 7 * 9 * * * * * * 7 * * * *
Quality
Consensus
KLAGAQVDTTLWAENYTPGEV++AQDEERAAGVWPTSKG+VPLT
Prm_Prm_84933/1-299 83 KPKIVGPTTPFDGGMKTYKRSNVAFKSGAKMAKSSAVFVVQAGA 126
Pin_PITG_04668T0/1-293 82 RPRTVGPSTPFDGGMKTFKRSNVAFRSGAKMTRANAVFVVQAGG 125
Pca_508923/1-300 89 KPRTVGPSTPFDGGMKTYKRSNVAFKSGAKMTRANAVFVVQAGG 132
Pso_533029/1-301 85 KPRVVGPTTPFDGGMKTYKRSNVAFKSGAKMTRANAVFVVQAGG 128
Conservation
9 * 9 6 * * * 9 * * * * * * * * * 9 * * * * * * * 9 * * * * * 7 9 8 9 * * * * * * * * +
Quality
Consensus
KPRTVGP+TPFDGGMKTYKRSNVAFKSGAKMTRANAVFVVQAGG
Prm_Prm_84933/1-299 127 TLSNVIIAGGGGVYCELHNCALVNVWFKDSIQSGLHINSGTGIT 170
Pin_PITG_04668T0/1-293 126 TLSNVIIAGGGGVFCETHNCALVNVWFKDSVQGALHVNSGTGIT 169
Pca_508923/1-300 133 TLSNVIIAGGGGVFCETHNCALVNVWFKDSVQGALHVNSGTGIT 176
Pso_533029/1-301 129 TLSNVIIAGGGGVFCETHNCALVNVWFKGSIQSALHVNSGTGIT 172
Conservation

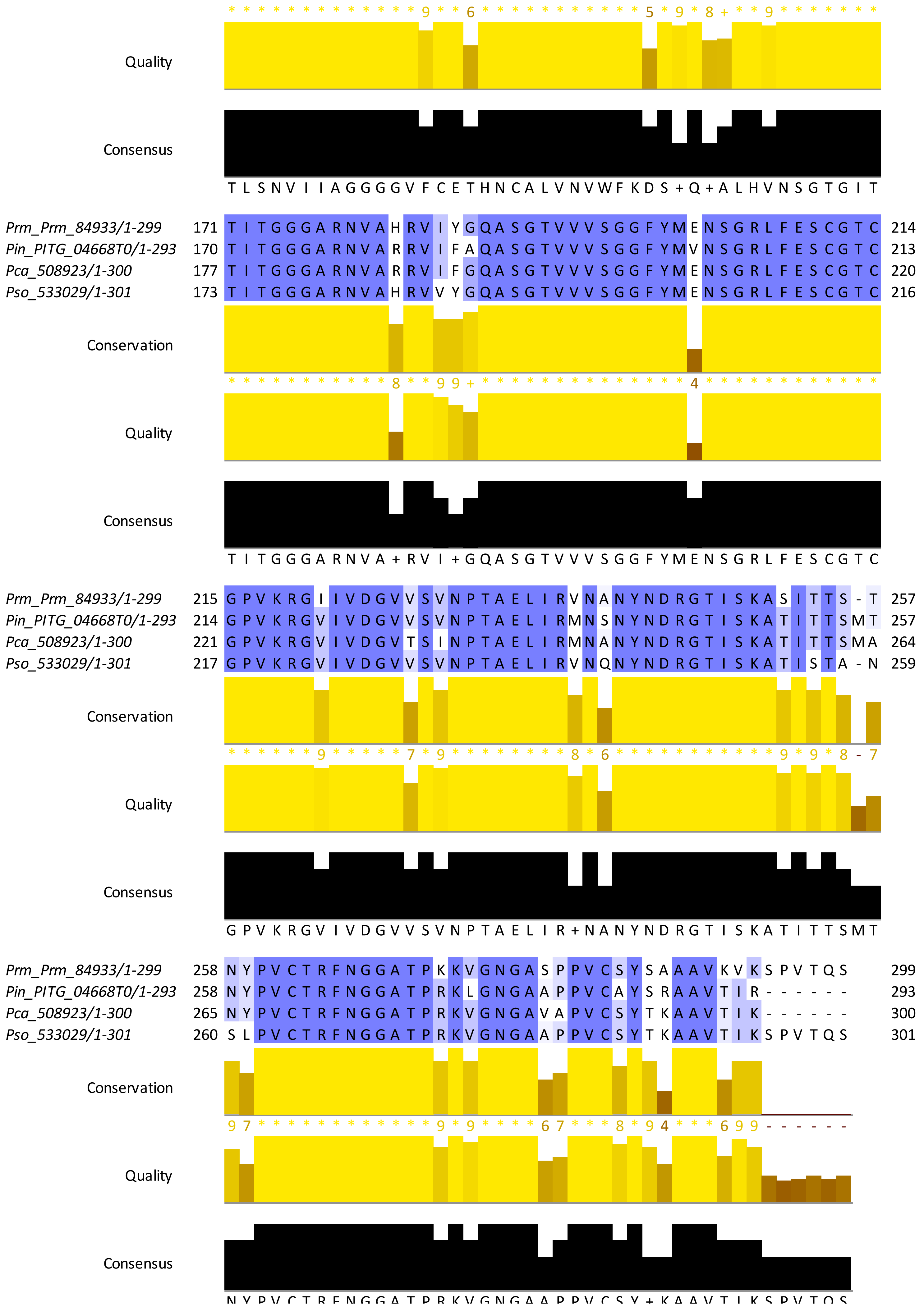
Quality
Consensus
TLSNVIIAGGGGVFCETHNCALVNVWFKDS+Q+ALHVNSGTGIT
Prm_Prm_84933/1-299 171 TITGGGARNVAHRVIYGQASGTVVVSGGFYMENSGRLFESCGTC 214
Pin_PITG_04668T0/1-293 170 TITGGGARNVARRVIFAQASGTVVVSGGFYMVNSGRLFESCGTC 213
Pca_508923/1-300 177 TITGGGARNVARRVIFGQASGTVVVSGGFYMENSGRLFESCGTC 220
Pso_533029/1-301 173 TITGGGARNVAHRVVYGQASGTVVVSGGFYMENSGRLFESCGTC 216
Conservation
Quality
Consensus
TITGGGARNVA+RVI+GQASGTVVVSGGFYMENSGRLFESCGTC
Prm_Prm_84933/1-299 215 GPVKRGIIVDGVVSVNPTAELIRVNANYNDRGTISKASITTS-T 257
Pin_PITG_04668T0/1-293 214 GPVKRGVIVDGVVSVNPTAELIRMNSNYNDRGTISKATITTSMT 257
Pca_508923/1-300 221 GPVKRGVIVDGVTSINPTAELIRMNANYNDRGTISKATITTSMA 264
Pso_533029/1-301 217 GPVKRGVIVDGVVSVNPTAELIRVNQNYNDRGTISKATISTA-N 259
Conservation
Quality
Consensus
GPVKRGVIVDGVVSVNPTAELIR+NANYNDRGTISKATITTSMT
Prm_Prm_84933/1-299 258 NYPVCTRFNGGATPKKVGNGASPPVCSYSAAAVKVKSPVTQS 299
Pin_PITG_04668T0/1-293 258 NYPVCTRFNGGATPRKLGNGAAPPVCAYSRAAVTIR------ 293
Pca_508923/1-300 265 NYPVCTRFNGGATPRKVGNGAVAPVCSYTKAAVTIK------ 300
Pso_533029/1-301 260 SLPVCTRFNGGATPRKVGNGAAPPVCSYTKAAVTIKSPVTQS 301
Conservation
Quality
Consensus
NYPVCTRFNGGATPRKVGNGAAPPVCSY+KAAVTIKSPVTQS

[illegible]

## Ortholog 25

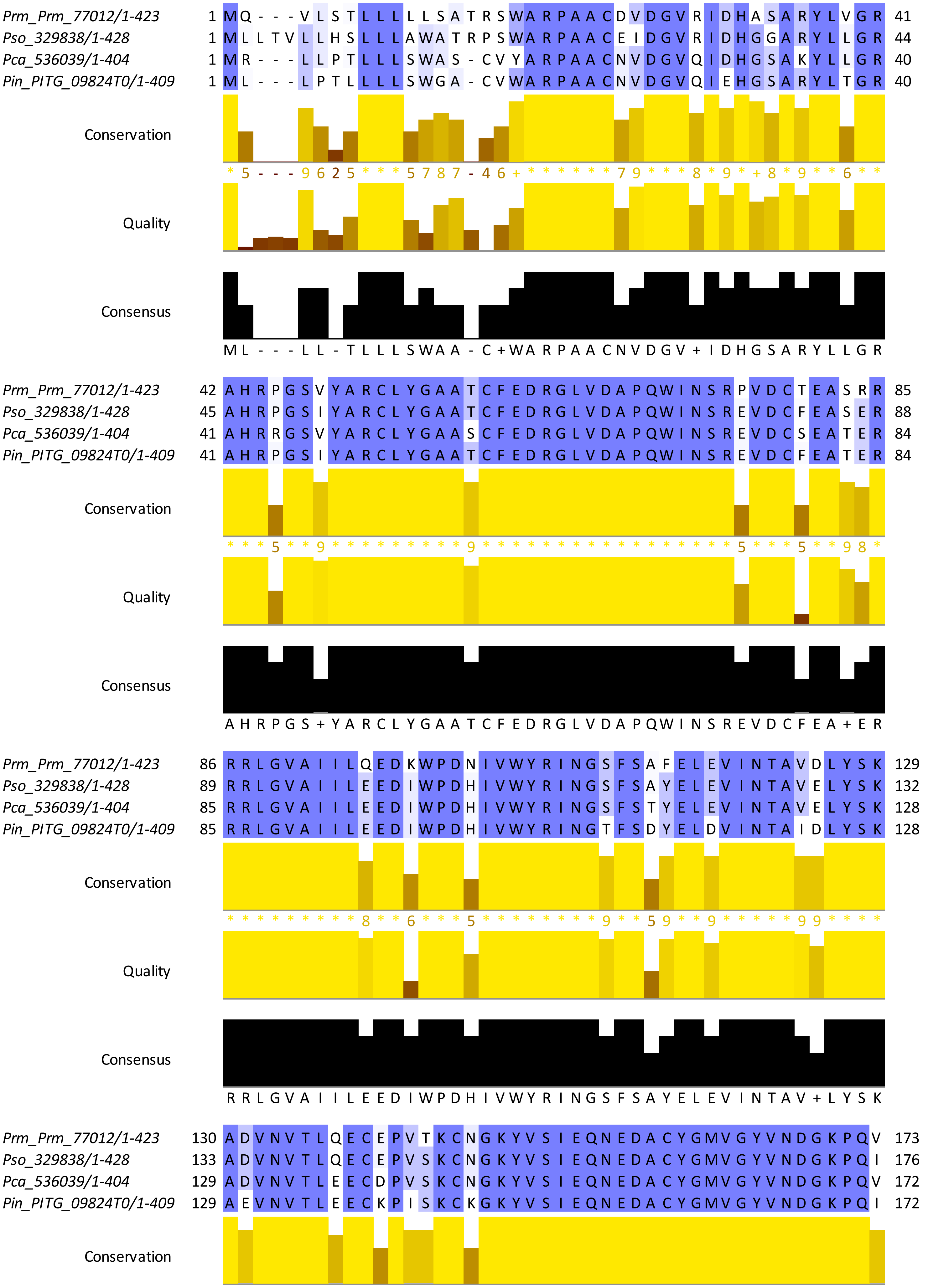
Prm_Prm_77012/1-423 1 MQ---VLSTLLLLLSATRSWARPAACDVDGVRIDHASARYLVGR 41
Pso_329838/1-428 1 MLLTVLLHSLLLAWATRPSWARPAACEIDGVRIDHGGARYLLGR 44
Pca_536039/1-404 1 MR---LLPTLLLSWAS-CVYARPAACNVDGVQIDHGSAKYLLGR 40
Pin_PITG_09824T0/1-409 1 ML---LPTLLLLSWGA-CVWARPAACNVDGVQIEHGSARYLTGR 40
Conservation
*5---9625***5787-46+******79***8*9*+8*9**6**
Quality
Consensus
ML---LL-TLLLSWAA-C+WARPAACNVDGV+IDHGSARYLLGR
Prm_Prm_77012/1-423 42 AHRPGSVYARCLYGAATCFEDRGLVDAPQWINSRPVDCTEASRR 85
Pso_329838/1-428 45 AHRPGSIYARCLYGAATCFEDRGLVDAPQWINSREVDCFEASER 88
Pca_536039/1-404 41 AHRRGSVYARCLYGAASCFEDRGLVDAPQWINSREVDCSEATER 84
Pin_PITG_09824T0/1-409 41 AHRPGSIYARCLYGAATCFEDRGLVDAPQWINSREVDCFEATER 84
Conservation
***5**9*********9*****************5***5**98*
Quality
Consensus
AHRPGS+YARCLYGAATCFEDRGLVDAPQWINSREVDCFEA+ER
Prm_Prm_77012/1-423 86 RRLGVAIILQEDKWPDNIVWYRINGSFSAFELEVINTAVDLYSK 129
Pso_329838/1-428 89 RRLGVAIILEEDIWPDHIVWYRINGSFSAYELEVINTAVELYSK 132
Pca_536039/1-404 85 RRLGVAIILEEDIWPDHIVWYRINGSFSTYELEVINTAVELYSK 128
Pin_PITG_09824T0/1-409 85 RRLGVAIILEEDIWPDHIVWYRINGTFSDYELDVINTAIDLYSK 128
Conservation
*********8**6***5********9**59**9*****99****
Quality
Consensus
RRLGVAIILEEDIWPDHIVWYRINGSFSAYELEVINTAV+LYSK
Prm_Prm_77012/1-423 130 ADVNVTLQECEPVTKCNGKYVSIEQNEDACYGMVGYVNDGKPQV 173
Pso_329838/1-428 133 ADVNVTLQECEPVSKCNGKYVSIEQNEDACYGMVGYVNDGKPQI 176
Pca_536039/1-404 129 ADVNVTLEECDPVSKCNGKYVSIEQNEDACYGMVGYVNDGKPQV 172
Pin_PITG_09824T0/1-409 129 AEVNVTLEECKPISKCKGKYVSIEQNEDACYGMVGYVNDGKPQI 172
Conservation

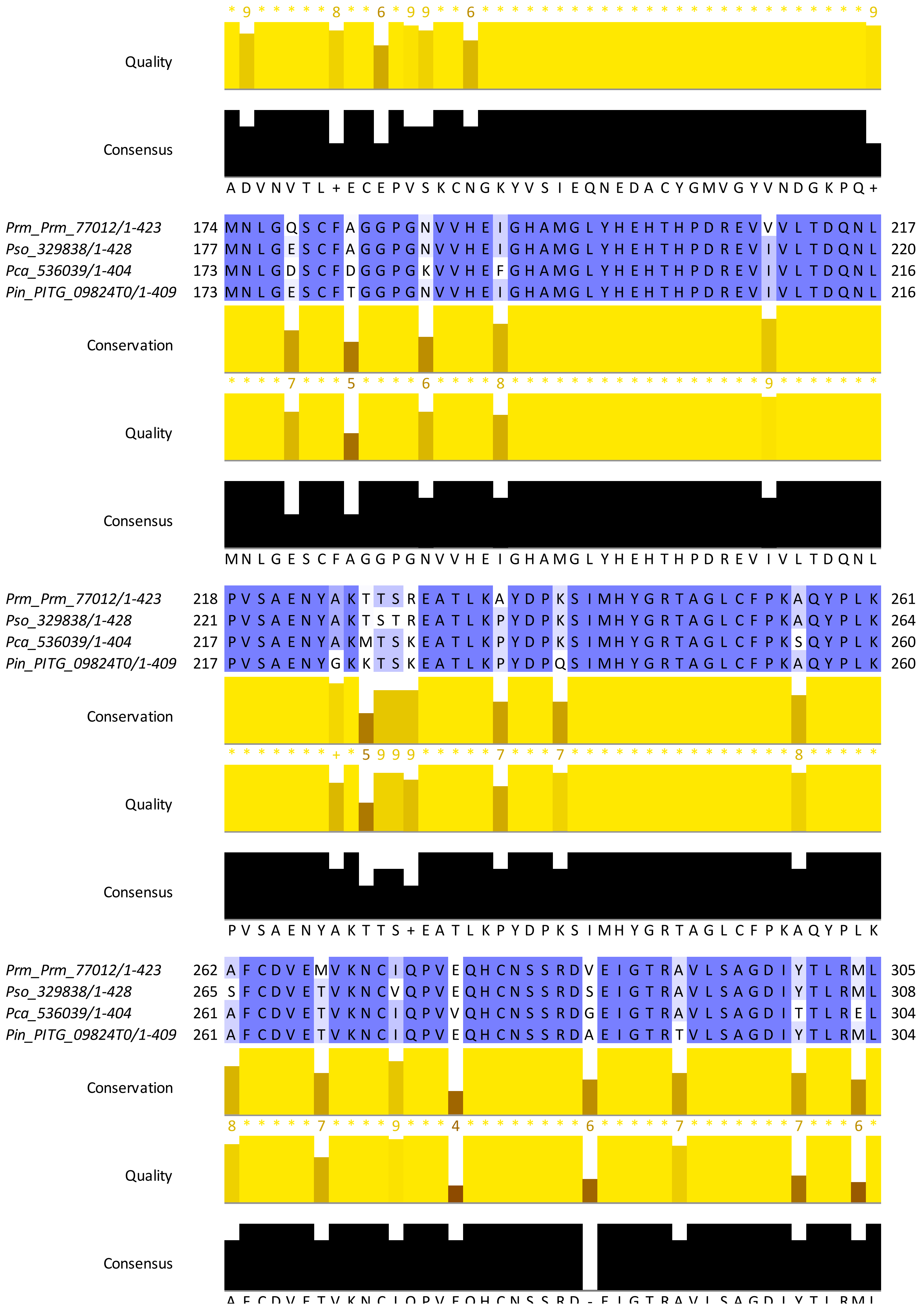
Quality
Consensus
A D V N V T L + E C E P V S K C N G K Y V S I E Q N E D A C Y G M V G Y V N D G K P Q +
Prm_Prm_77012/1-423 174 MNLGQSCFAGGPGNVVHEIGHAMGLYHEHTHPDREVVVLTDQNL 217
Pso_329838/1-428 177 MNLGESCFAGGPGNVVHEIGHAMGLYHEHTHPDREVIVLTDQNL 220
Pca_536039/1-404 173 MNLGDSCFDGGPGKVVHEFGHAMGLYHEHTHPDREVIVLTDQNL 216
Pin_PITG_09824T0/1-409 173 MNLGESCFTGGPGNVVHEIGHAMGLYHEHTHPDREVIVLTDQNL 216
Conservation
Quality
Consensus
MNLGESCFAGGPGNVVHEIGHAMGLYHEHTHPDREVIVLTDQNL
Prm_Prm_77012/1-423 218 PVSAENYAKTTSREATLKAYDPKSIMHYGRTAGLCFPKAQYPLK 261
Pso_329838/1-428 221 PVSAENYAKTSTREATLKPYDPKSIMHYGRTAGLCFPKAQYPLK 264
Pca_536039/1-404 217 PVSAENYAKMTSKEATLKPYDPKSIMHYGRTAGLCFPKSQYPLK 260
Pin_PITG_09824T0/1-409 217 PVSAENYGKKTSKEATLKPYDPQSIMHYGRTAGLCFPKAQYPLK 260
Conservation
Quality
Consensus
PVSAENYAKTTS+EATLKPYDPKSIMHYGRTAGLCFPKAQYPLK
Prm_Prm_77012/1-423 262 AFCDVEMVKNCIQPVEQHCNSSRDVEIGTRAVLSAGDIYTLRML 305
Pso_329838/1-428 265 SFCDVETVKNCVQPVEQHCNSSRDSEIGTRAVLSAGDIYTLRML 308
Pca_536039/1-404 261 AFCDVETVKNCIQPVVQHCNSSRDGEIGTRAVLSAGDITTLREL 304
Pin_PITG_09824T0/1-409 261 AFCDVETVKNCIQPVEQHCNSSRDAEIGTRTVLSAGDIYTLRML 304
Conservation
Quality
Consensus
AFCDVETVKNCIQPVEQHCNSSRD-EIGTRAVLSAGDIYTLRML

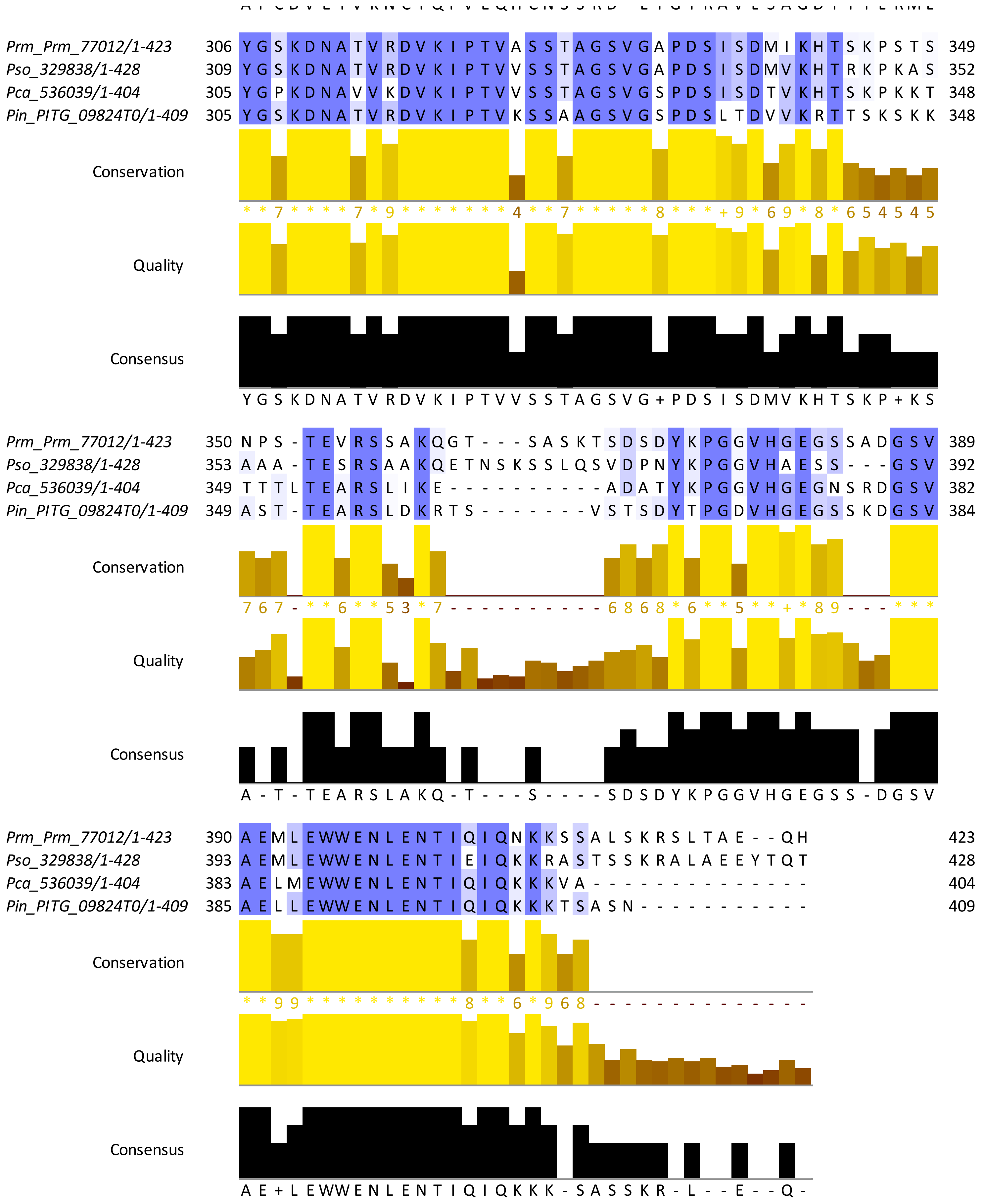

Prm_Prm_77012/1-423 306 YGSKDNATVRDVKIPTVASSTAGSVGAPDSISDMIKHTSKPSTS 349
Pso_329838/1-428 309 YGSKDNATVRDVKIPTVVSSTAGSVGAPDSISDMVKHTRKPKAS 352
Pca_536039/1-404 305 YGPKDNAVVKDVKIPTVVSSTAGSVGSPDSISDTVKHTSKPKKT 348
Pin_PITG_09824T0/1-409 305 YGSKDNATVRDVKIPTVKSSAAGSVGSPDSLTDVVKRTTSKSKK 348
Conservation
**7****7*9*******4**7*****8***+9*69*8*654545
Quality
Consensus
YGSKDNATVRDVKIPTVVSSTAGSVG+PDSISDMVKHTSKP+KS
Prm_Prm_77012/1-423 350 NPS-TEVRSSAKQGT---SASKTSDSDYKPGGVHGEGSSADGSV 389
Pso_329838/1-428 353 AAA-TESRSAAKQETNSKSSLQSVDPNYKPGGVHAESS---GSV 392
Pca_536039/1-404 349 TTTLTEARSLIKE----------ADATYKPGGVHGEGNSRDGSV 382
Pin_PITG_09824T0/1-409 349 AST-TEARSLDKRTS-------VSTSDYTPGDVHGEGSSKDGSV 384
Conservation
767-**6**53*7----------6868*6**5**+*89---***
Quality
Consensus
A-T-TEARSLAKQ-T---S----SDSDYKPGGVHGEGSS-DGSV
Prm_Prm_77012/1-423 390 AEMLEWWENLENTIQIQNKKSSALSKRSLTAE--QH 423
Pso_329838/1-428 393 AEMLEWWENLENTIEIQKKRASTSSKRALAEEYTQT 428
Pca_536039/1-404 383 AELMEWWENLENTIQIQKKKVA-------------- 404
Pin_PITG_09824T0/1-409 385 AELLEWWENLENTIQIQKKKTSASN----------- 409
Conservation
**99**********8**6*968--------------
Quality
Consensus
AE+LEWWENLENTIQIQKKK-SASSKR-L--E--Q-

Ortholog 26

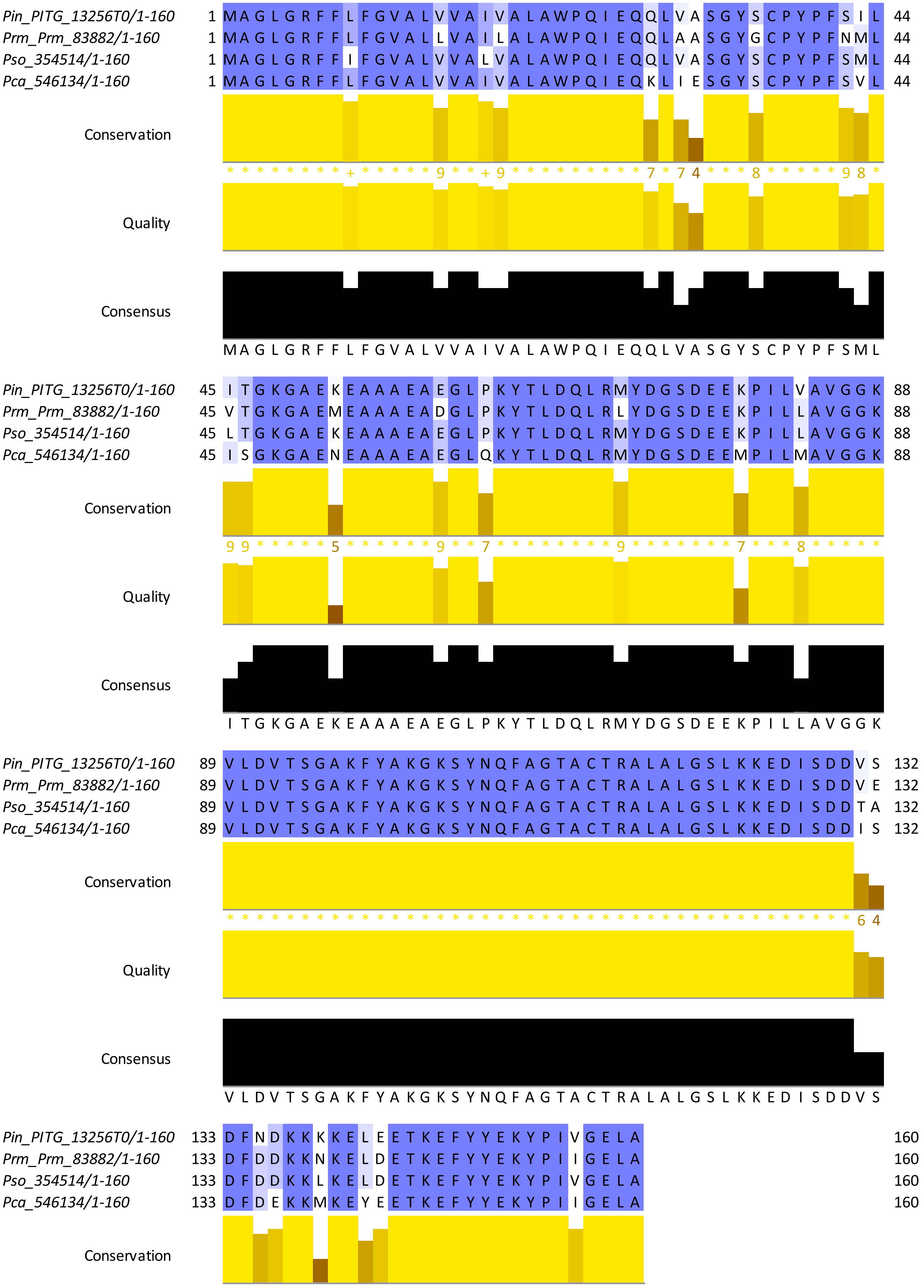
Pin_PITG_13256T0/1-160 1 MAGLGRFFLFGVALVVAIVALAWPQIEQQLVASGYSCPYPFSIL 44
Prm_Prm_83882/1-160 1 MAGLGRFFLFGVALLVAILLALAWPQIEQQLAASGYGCPYPFNML 44
Pso_354514/1-160 1 MAGLGRFFIFGVALVVALVALAWPQIEQQLVASGYSCPYPFSML 44
Pca_546134/1-160 1 MAGLGRFFLFGVALVVAIVALAWPQIEQKLIESGYSCPYPFSVL 44
Conservation
* * * * * * * * + * * * * * 9 * * + 9 * * * * * * * * * 7 * 7 4 * * * 8 * * * * * 9 8 *
Quality
Consensus
MAGLGRFFLFGVALVVAIVALAWPQIEQQLVASGYSCPYPFSML
Pin_PITG_13256T0/1-160 45 ITGKGAEKEAAAEAEGLPKYTLDQLRMYDGSDEEKPILVAVGGK 88
Prm_Prm_83882/1-160 45 VTGKGAEMEAAAEADGLPKYTLDQLRLYDGSDEEKPILLAVGGK 88
Pso_354514/1-160 45 LTGKGAEKEAAAEAEGLPKYTLDQLRMYDGSDEEKPILLAVGGK 88
Pca_546134/1-160 45 ISGKGAENEAAAEAEGLQKYTLDQLRMYDGSDEEMPILMAVGGK 88
Conservation
9 9 * * * * * 5 * * * * * * 9 * * 7 * * * * * * * * 9 * * * * * * * 7 * * * 8 * * * * *
Quality
Consensus
ITGKGAEKEAAAEAEGLPKYTLDQLRMYDGSDEEKPILLAVGGK
Pin_PITG_13256T0/1-160 89 VLDVTSGAKFYAKGKSYNQFAGTACTRALALGSLKKEDISDDVS 132
Prm_Prm_83882/1-160 89 VLDVTSGAKFYAKGKSYNQFAGTACTRALALGSLKKEDISDDVE 132
Pso_354514/1-160 89 VLDVTSGAKFYAKGKSYNQFAGTACTRALALGSLKKEDISDDTA 132
Pca_546134/1-160 89 VLDVTSGAKFYAKGKSYNQFAGTACTRALALGSLKKEDISDDIS 132
Conservation
* * * * * * * * * * * * * * * * * * * * * * * * * * * * * * * * * * * * * * * * * * 6 4
Quality
Consensus
VLDVTSGAKFYAKGKSYNQFAGTACTRALALGSLKKEDISDDVS
Pin_PITG_13256T0/1-160 133 DFNDKKKKELEETKEFYYEKYPIVGELA 160
Prm_Prm_83882/1-160 133 DFDDKKNKELDETKEFYYEKYPIIGELA 160
Pso_354514/1-160 133 DFDDKKLKELDETKEFYYEKYPIVGELA 160
Pca_546134/1-160 133 DFDEKKMKEYEETKEFYYEKYPIIGELA 160
Conservation

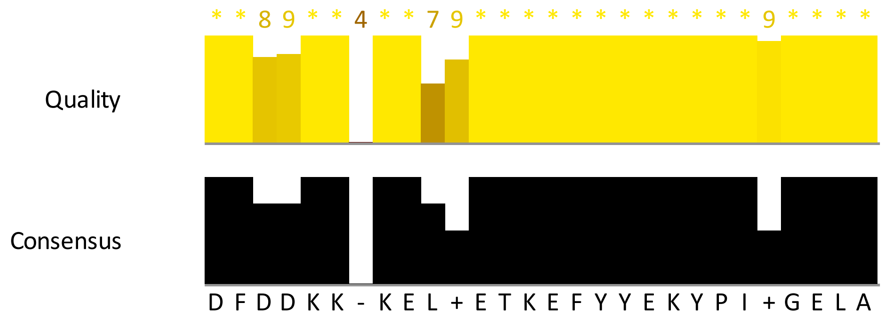
* * 8 9 * * 4 * * 7 9 * * * * * * * * * * * * 9 * * * *
Quality
Consensus
D F D D K K - K E L + E T K E F Y Y E K Y P I + G E L A

Ortholog 27

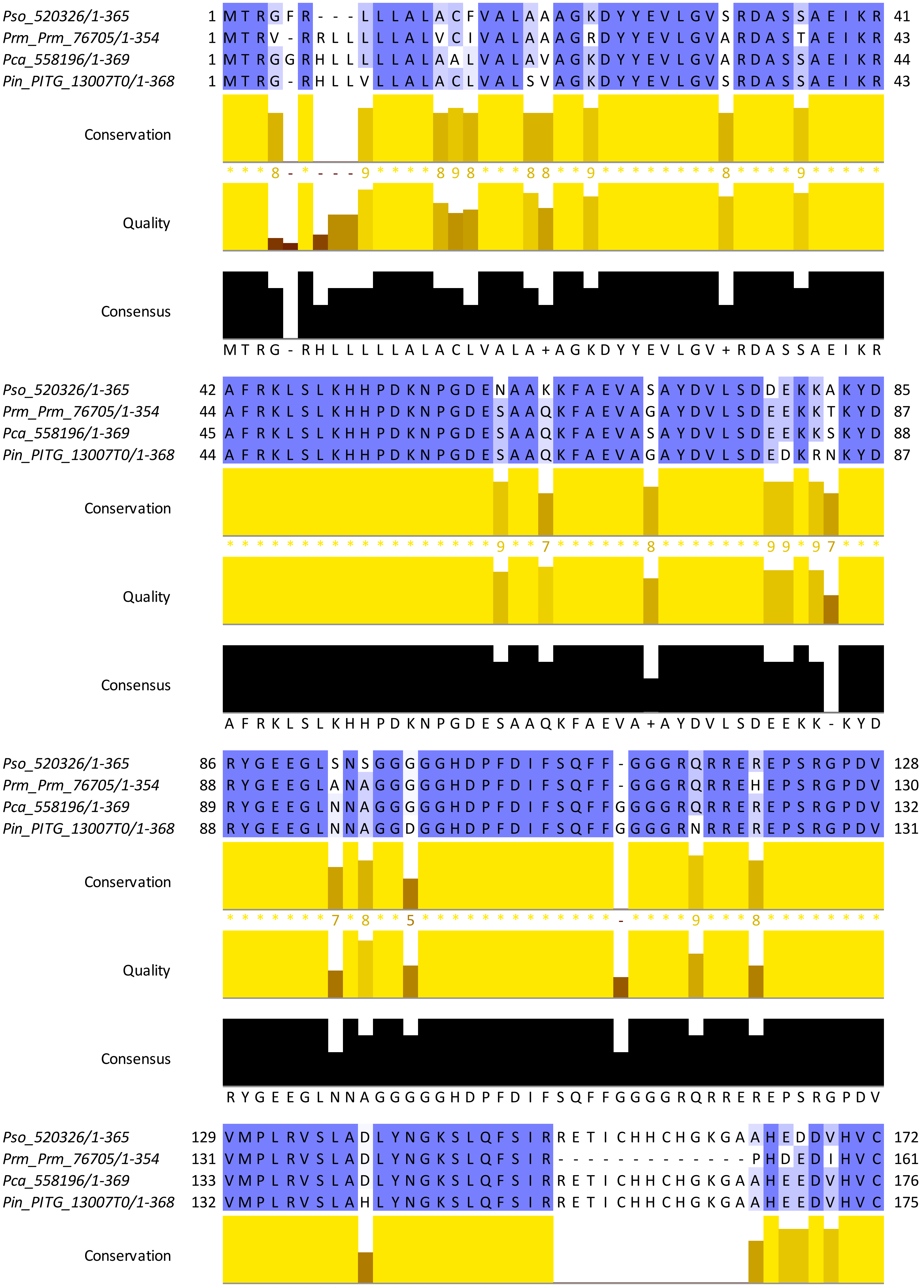
Pso_520326/1-365 1 MTRGFR---LLLALACFVALAAAGKDYYEVLGVSRDASSAEIKR 41
Prm_Prm_76705/1-354 1 MTRV-RRLLLLLALVCIVALAAAGRDYYEVLGVARDASTAEIKR 43
Pca_558196/1-369 1 MTRGGRHLLLLLALAALVALAVAGKDYYEVLGVARDASSAEIKR 44
Pin_PITG_13007T0/1-368 1 MTRG-RHLLVLLALACLVALSVAGKDYYEVLGVSRDASSAEIKR 43
Conservation
***8-*---9****898***88**9********8****9*****
Quality
Consensus
MTRG-RHLLLLLALACLVALA+AGKDYYEVLGV+RDASSAEIKR
Pso_520326/1-365 42 AFRKLSLKHHPDKNPGDENAAKKFAEVASAYDVLSDDEKKAKYD 85
Prm_Prm_76705/1-354 44 AFRKLSLKHHPDKNPGDESAAQKFAEVAGAYDVLSDEEKKTKYD 87
Pca_558196/1-369 45 AFRKLSLKHHPDKNPGDESAAQKFAEVASAYDVLSDEEKKSKYD 88
Pin_PITG_13007T0/1-368 44 AFRKLSLKHHPDKNPGDESAAQKFAEVAGAYDVLSDEDKRNKYD 87
Conservation
******************9**7******8*******99*97***
Quality
Consensus
AFRKLSLKHHPDKNPGDESAAQKFAEVA+AYDVLSDEEKK-KYD
Pso_520326/1-365 86 RYGEEGLSNSGGGGGHDPFDIFSQFF-GGGRQRREREPSRGPDV 128
Prm_Prm_76705/1-354 88 RYGEEGLANAGGGGGHDPFDIFSQFF-GGGRQRREHEPSRGPDV 130
Pca_558196/1-369 89 RYGEEGLNNAGGGGGHDPFDIFSQFFGGGGRQRREREPSRGPDV 132
Pin_PITG_13007T0/1-368 88 RYGEEGLNNAGGDGGHDPFDIFSQFFGGGGRNRREREPSRGPDV 131
Conservation
*******7*8**5**************-****9***8********
Quality
Consensus
RYGEEGLNNAGGGGGHDPFDIFSQFFGGGGRQRREREPSRGPDV
Pso_520326/1-365 129 VMPLRVSLADLYNGKSLQFSIRRETICHHCHGKGAAHEDDVHVC 172
Prm_Prm_76705/1-354 131 VMPLRVSLADLYNGKSLQFSIR-------------PHDEDIHVC 161
Pca_558196/1-369 133 VMPLRVSLADLYNGKSLQFSIRRETICHHCHGKGAAHEEDVHVC 176
Pin_PITG_13007T0/1-368 132 VMPLRVSLAHLYNGKSLQFSIRRETICHHCHGKGAAHEEDVHVC 175
Conservation

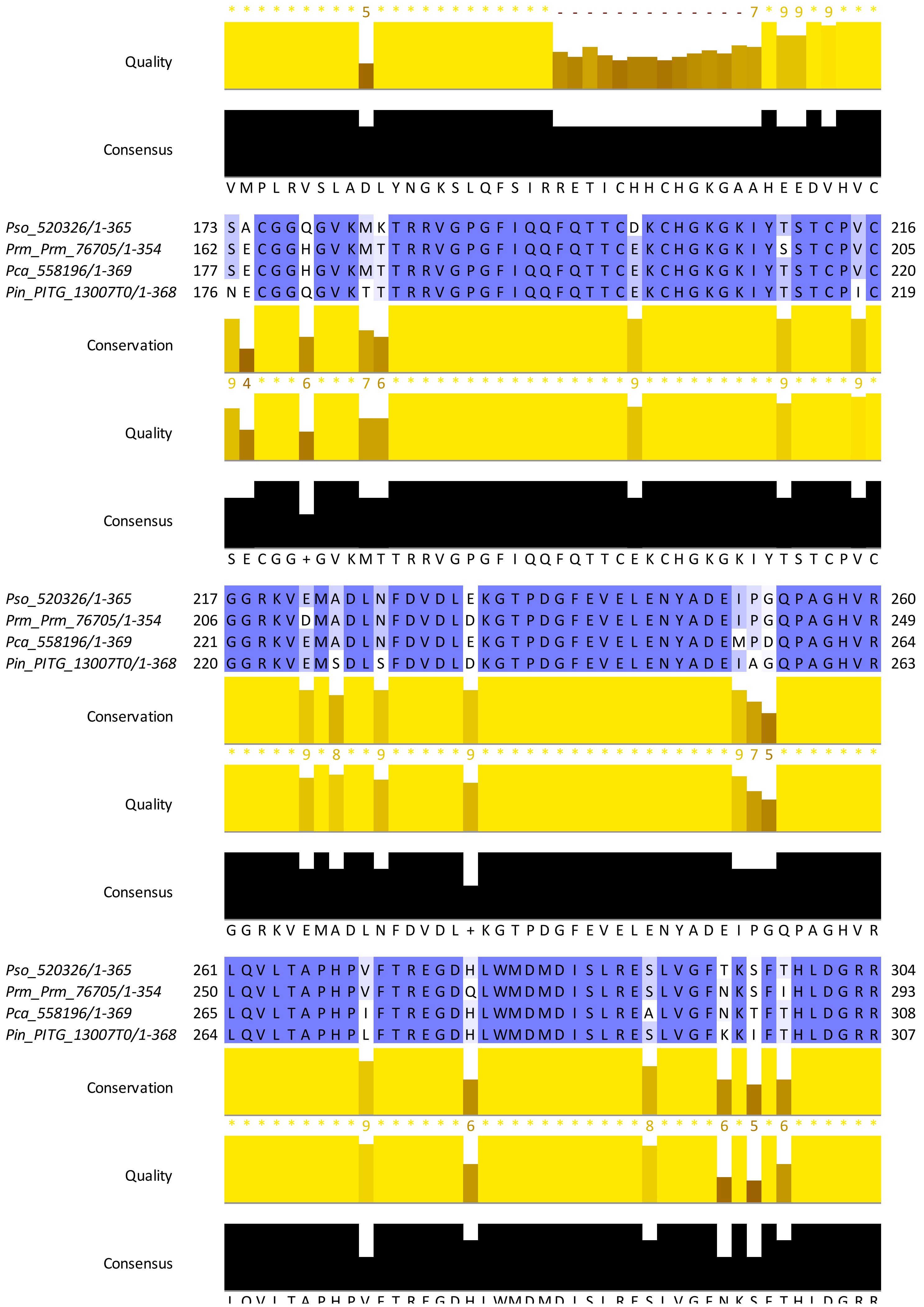
* * * * * * * * * 5 * * * * * * * * * * * * - - - - - - - - - - - - - - 7 * 9 9 * 9 * * *
Quality
Consensus
VMPLRVSLADLYNGKSLQFSIRRETICHHCHGKGAAHEEDVHVC
Pso_520326/1-365 173 SACGGQGVKMKTRRVGPGFIQQFQTTCDKCHGKGKIYTSTCPVC 216
Prm_Prm_76705/1-354 162 SECGGHGVKMTTRRVGPGFIQQFQTTCEKCHGKGKIYSSTCPVC 205
Pca_558196/1-369 177 SECGGHGVKMTTRRVGPGFIQQFQTTCEKCHGKGKIYTSTCPVC 220
Pin_PITG_13007T0/1-368 176 NECGGQGVKTTTRRVGPGFIQQFQTTCEKCHGKGKIYTSTCPIC 219
Conservation
9 4 * * * 6 * * * 7 6 * * * * * * * * * * * * * * * * 9 * * * * * * * * * 9 * * * * 9 *
Quality
Consensus
SECGG+GVKMTTRRVGPGFIQQFQTTCEKCHGKGKIYTSTCPVC
Pso_520326/1-365 217 GGRKVEMADLNFDVDLEKGTPDGFEVELENYADEIPGQPAGHVR 260
Prm_Prm_76705/1-354 206 GGRKVDMADLNFDVDLDKGTPDGFEVELENYADEIPGQPAGHVR 249
Pca_558196/1-369 221 GGRKVEMADLNFDVDLEKGTPDGFEVELENYADEMPDQPAGHVR 264
Pin_PITG_13007T0/1-368 220 GGRKVEMSDLSFDVDLDKGTPDGFEVELENYADEIAGQPAGHVR 263
Conservation
* * * * * 9 * 8 * * 9 * * * * * 9 * * * * * * * * * * * * * * * * * 9 7 5 * * * * * * *
Quality
Consensus
GGRKVEMADLNFDVDL+KGTPDGFEVELENYADEIPGQPAGHVR
Pso_520326/1-365 261 LQVLTAPHPVFTREGDHLWMDMDISLRESLVGFTKSFTHLDGRR 304
Prm_Prm_76705/1-354 250 LQVLTAPHPVFTREGDQLWMDMDISLRESLVGFNKSFIHLDGRR 293
Pca_558196/1-369 265 LQVLTAPHPIFTREGDHLWMDMDISLREALVGFNKTFTHLDGRR 308
Pin_PITG_13007T0/1-368 264 LQVLTAPHPLFTREGDHLWMDMDISLRESLVGFKKIFTHLDGRR 307
Conservation
* * * * * * * * * 9 * * * * * * 6 * * * * * * * * * * * * * 8 * * * * 6 * 5 * 6 * * * * * * *
Quality
Consensus
LQVLTAPHPVFTREGDHLWMDMDISLRESLVGFNKSFTHLDGRR

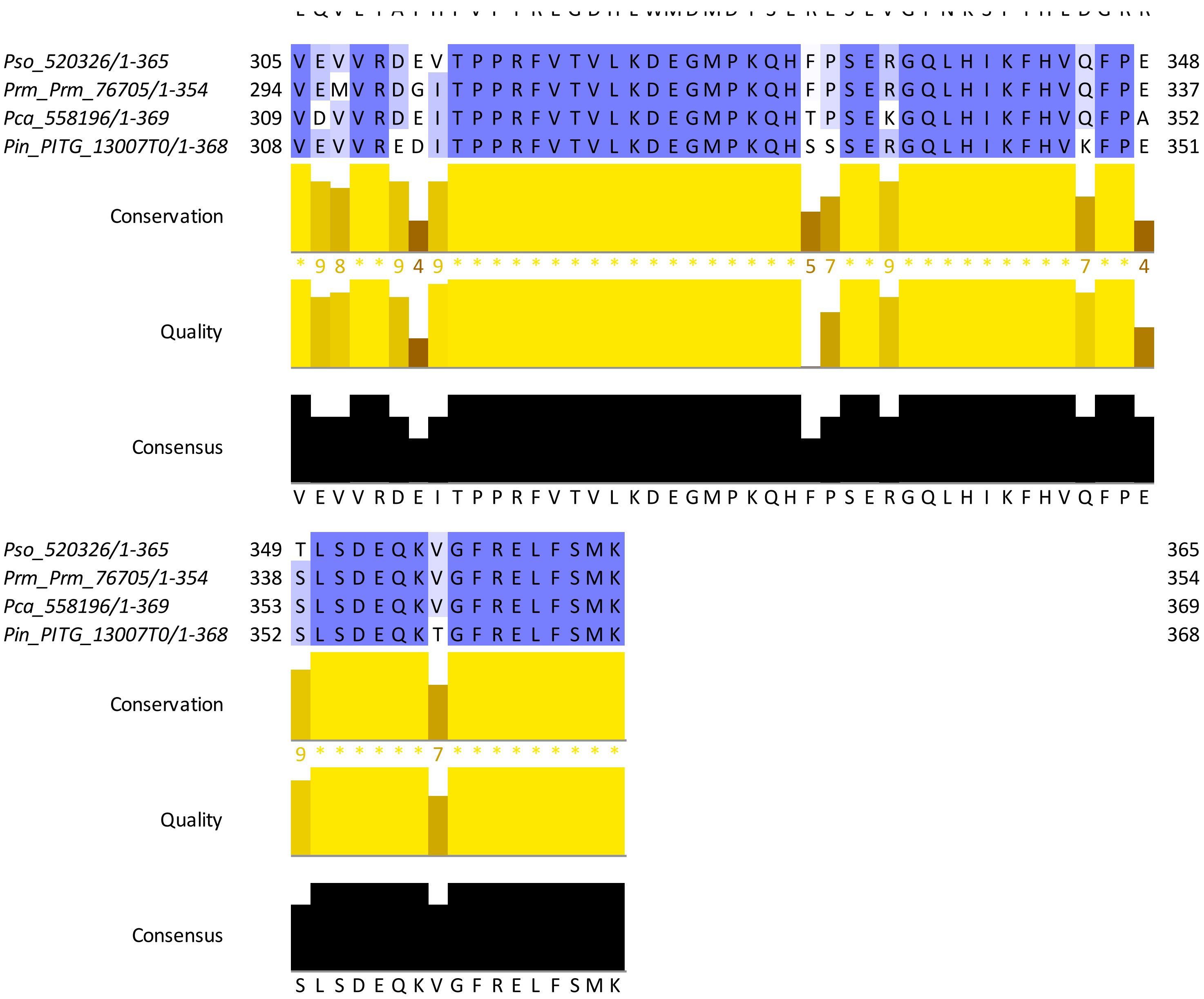
Pso_520326/1-365 305 VEVVRDEVTPPRFVTVLKDEGMPKQHFPSERGQLHIKFHVQFPE 348
Prm_Prm_76705/1-354 294 VEMVRDGITPPRFVTVLKDEGMPKQHFPSERGQLHIKFHVQFPE 337
Pca_558196/1-369 309 VDVVRDEITPPRFVTVLKDEGMPKQHTPSEKGQLHIKFHVQFPA 352
Pin_PITG_13007T0/1-368 308 VEVVREDITPPRFVTVLKDEGMPKQHSSSERGQLHIKFHVKFPE 351
Conservation
* 9 8 * * 9 4 9 * * * * * * * * * * * * * * * * * * 5 7 * * 9 * * * * * * * * * 7 * * 4
Quality
Consensus
VEVVRDEITPPRFVTVLKDEGMPKQHFPSERGQLHIKFHVQFPE
Pso_520326/1-365 349 TLSDEQKVGFRELFSMK 365
Prm_Prm_76705/1-354 338 SLSDEQKVGFRELFSMK 354
Pca_558196/1-369 353 SLSDEQKVGFRELFSMK 369
Pin_PITG_13007T0/1-368 352 SLSDEQKTGFRELFSMK 368
Conservation
9 * * * * * * 7 * * * * * * * * *
Quality
Consensus
SLSDEQKVGFRELFSMK

Ortholog 28

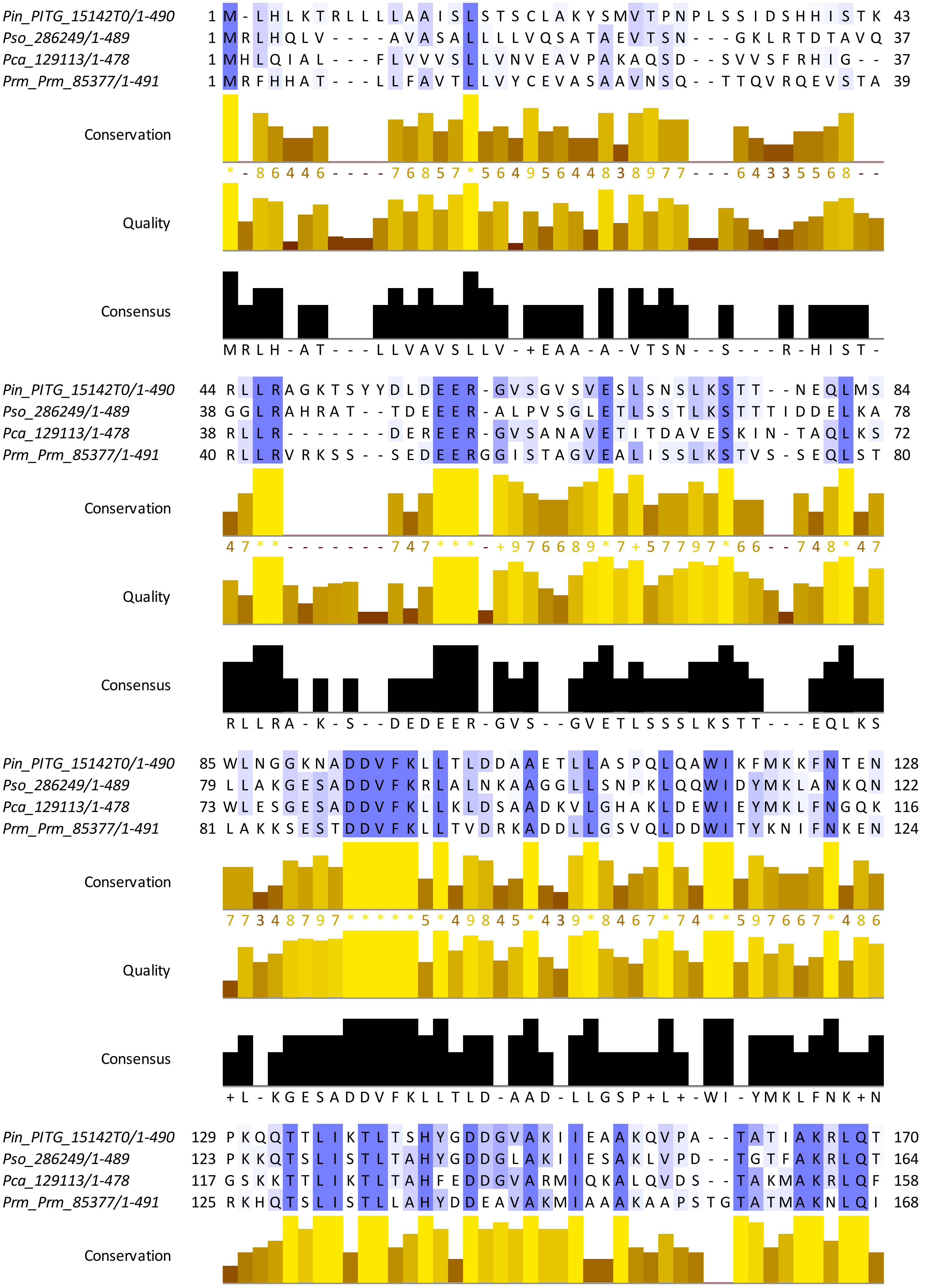
Pin_PITG_15142T0/1-490 1 M-LHLKTRLLLLAAISLSTSCLAKYSMVTPNPLSSIDSHHISTK 43
Pso_286249/1-489 1 MRLHQLV----AVASALLLLVQSATAEVTSN---GKLRTDTAVQ 37
Pca_129113/1-478 1 MHLQIAL---FLVVVSLLVNVEAVPAKAQSD--SVVSFRHIG-- 37
Prm_Prm_85377/1-491 1 MRFHHAT---LLFAVTLLVYCEVASAAVNSQ--TTQVRQEVSTA 39
Conservation
*-86446----76857*5649564483897 7---64335568--
Quality
Consensus
MRLH-AT---LLVAVSLLV-+EAA-A-VTSN--S---R-HIST-
Pin_PITG_15142T0/1-490 44 RLLRAGKTSYYDLDEER-GVSGVSVESLSNSLKSTT--NEQLMS 84
Pso_286249/1-489 38 GGLRAHRAT--TDEEEER-ALPVSGLETLSSTLKSTTTIDDELKA 78
Pca_129113/1-478 38 RLLR-------DEREER-GVSANAVETITDAVESKIN-TAQLKS 72
Prm_Prm_85377/1-491 40 RLLRVRKSS--SEDEERGGISTAGVEALISSLKSTVS-SEQLST 80
Conservation
47**--------747***-+976689*7+57797*66--748*47
Quality
Consensus
RLLRA-K-S--DEDEER-GVS--GVETLSSSLKSTT---EQLKS
Pin_PITG_15142T0/1-490 85 WLNGGKNADDVFKLLTLDDAAETLLASPQLQAWIKFMKKFNTEN 128
Pso_286249/1-489 79 LLAKGESADDVFKRLALNKAAGGLLSNPKLQQWIDYMKLANKQN 122
Pca_129113/1-478 73 WLESGESADDVFKLLKLDSAADKVLGHAKLDEWIEYMKLFNGQK 116
Prm_Prm_85377/1-491 81 LAKKSESTDDVFKLLTVDRKADDLLGSVQLDDWITYKNIFNKEN 124
Conservation
77348797*****5*49845*439*8467*74**597667*486
Quality
Consensus
+L-KGESADDVFKLLTLD-AAD-LLGSP+L+-WI-YMKLFNK+N
Pin_PITG_15142T0/1-490 129 PKQQTTLIKTLTSHYGDDGVAKIIEAAKQVPA--TATIAKRLQT 170
Pso_286249/1-489 123 PKKQTSLISTLTAHYGDDGLAKIIESAKLVPD--TGTFAKRLQT 164
Pca_129113/1-478 117 GSKKTTLIKTLTAHFEDDGVARMIQKALQVDS--TAKMAKRLQF 158
Prm_Prm_85377/1-491 125 RKHQTSLISTLLAHYDDEAVAKMIAAAKAAPSTGTATMAKNLQI 168
Conservation

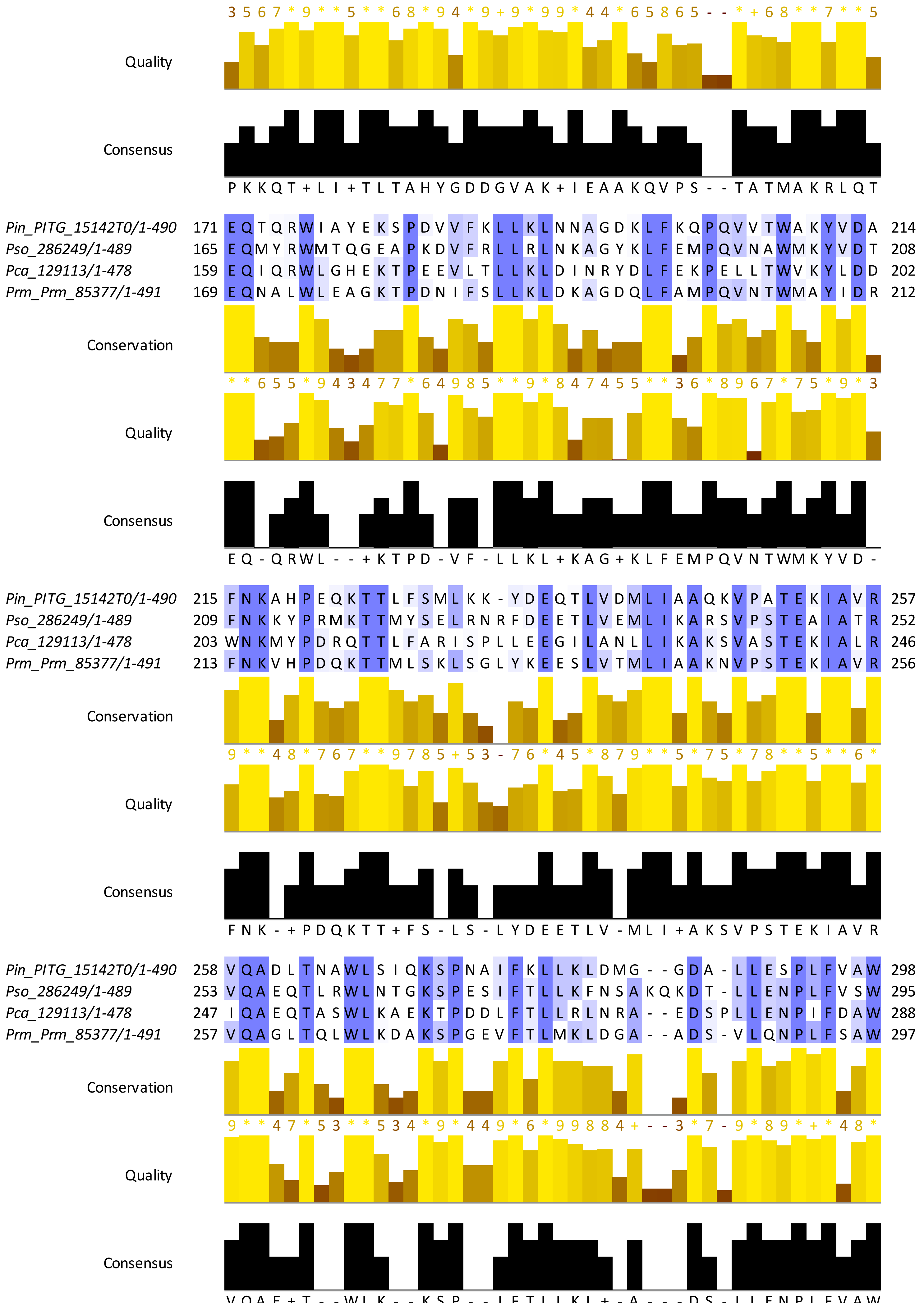

3 5 6 7 * 9 * * 5 * * 6 8 * 9 4 * 9 + 9 * 9 9 * 4 4 * 6 5 8 6 5 - - * + 6 8 * * 7 * * 5
Quality
Consensus
PKKQT+LI+TLTAHYGDDGVAK+IEAAKQVPS--TATMAKRLQT
Pin_PITG_15142T0/1-490 171 EQTQRWIAYEKSPDVVFKLLKLNNAGDKLFKQPQVVTWAKYVDA 214
Pso_286249/1-489 165 EQMYRWMTQGEAPKDVFRLLRLNKAGYKLFEMPQVNAWMKYVDT 208
Pca_129113/1-478 159 EQIQRWLGHEKTPEEVLTLLKLDINRYDLFEKPELLTWVKYLDD 202
Prm_Prm_85377/1-491 169 EQNALWLEAGKTPDNIFSLLKLDKAGDQLFAMPQVNTWMAYIDR 212
Conservation
* * 6 5 5 * 9 4 3 4 7 7 * 6 4 9 8 5 * * 9 * 8 4 7 4 5 5 * * 3 6 * 8 9 6 7 * 7 5 * 9 * 3
Quality
Consensus
EQ-QRWL--+KTPD-VF-LLKL+KAG+KLFEMPQVNTWMKYVD-
Pin_PITG_15142T0/1-490 215 FNKAHPEQKTTLFSMLKK-YDEQTLVDMLIAAQKVPATEKIAVR 257
Pso_286249/1-489 209 FNKKYPRMKTTMYSELRNRFDEETLVEMLIKARSVPSTEAIATR 252
Pca_129113/1-478 203 WNKMYPDRQTTLFARISPLLEEGILANLLIKAKSVASTEKIALR 246
Prm_Prm_85377/1-491 213 FNKVHPDQKTTMLSKLSGLYKEESLVTMLIAAKNVPSTEKIAVR 256
Conservation
9 * * 4 8 * 7 6 7 * * 9 7 8 5 + 5 3 - 7 6 * 4 5 * 8 7 9 * * 5 * 7 5 * 7 8 * * 5 * * 6 *
Quality
Consensus
FNK-+PDQKTT+FS-LS-LYDEETLV-MLI+AKSVPSTEKIAVR
Pin_PITG_15142T0/1-490 258 VQADLTNAWLSIQKSPNAIFKLLKLDMG--GDA-LLESPLFVAW 298
Pso_286249/1-489 253 VQAEQTLRWLNTGKSPESIFTLLKFNSAKQKDT-LLENPLFVSW 295
Pca_129113/1-478 247 IQAEQTASWLKAEKTPDDLFTLLRLNRA--EDSPLLENPIFDAW 288
Prm_Prm_85377/1-491 257 VQAGLTQLWLKDAKSPGEVFTLMKLDGA--ADS-VLQNPLFSAW 297
Conservation
9 * * 4 7 * 5 3 * * 5 3 4 * 9 * 4 4 9 * 6 * 9 9 8 8 4 + - - 3 * 7 - 9 * 8 9 * + * 4 8 *
Quality
Consensus

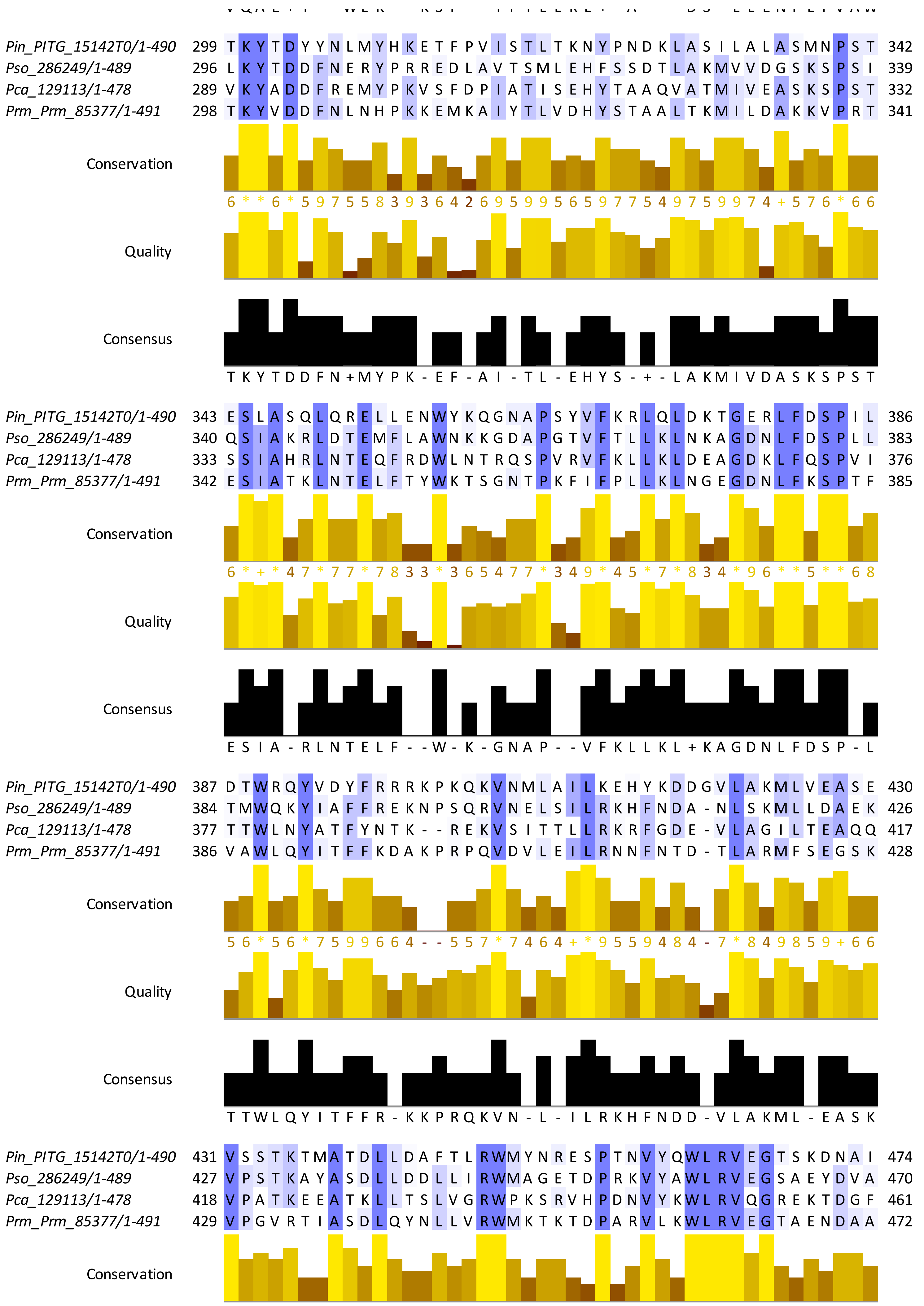

Pin_PITG_15142T0/1-490 299 TKYTDYYNLMYHKETFPVISTLTKNYPNDKLASILALASMNPST 342
Pso_286249/1-489 296 LKYTDDFNERYPRREDLAVTSMLEHFSSDTLAKMVVDGSKSPSI 339
Pca_129113/1-478 289 VKYADDFREMYPKVSFDPIATISEHYTAAQVATMIVEASKSPST 332
Prm_Prm_85377/1-491 298 TKYVDDFNLNHPKKEMKAIYTLVDHYSTAALTKMILDAKKVPRT 341
Conservation
6**6*59755839364269599565977549759974+576*66
Quality
Consensus
TKYTDDFN+MYPK-EF-AI-TL-EHYS-+-LAKMIVDASKSPST
Pin_PITG_15142T0/1-490 343 ESLASQLQRELLENWYKQGNAPSYVFKRLQLDKTGERLFDSPIL 386
Pso_286249/1-489 340 QSIAKRLDTEMFLAWNKKGDAPGTVFTLLKLNKAGDNLFDSPLL 383
Pca_129113/1-478 333 SSIAHRLNTEQFRDWLNTRQSPVRVFKLLKLDEAGDKLFQSPVI 376
Prm_Prm_85377/1-491 342 ESIATKLNTELFTYWKTSGNTPKFIFPLLKLNGEGDNLFKSPTF 385
Conservation
6*+*47*77*7833*3654777*349*45*7*834*96**5**68
Quality
Consensus
ESIA-RLNTELF--W-K-GNAP--VFKLLKL+KAGDNLFDSP-L
Pin_PITG_15142T0/1-490 387 DTWRQYVDYFRRRKPKQKVNMLAILKEHYKDDGVLAKMLVEASE 430
Pso_286249/1-489 384 TMWQKYIAFFREKNPSQRVNELSILRKHFNDA-NLSKMLLDAEK 426
Pca_129113/1-478 377 TTWLNYATFYNTK--REKVSITTLLRKRFGDE-VLAGILTEAQQ 417
Prm_Prm_85377/1-491 386 VAWLQYITFFKDAKPRPQVDVLEILRNNFNTD-TLARMFSEGSK 428
Conservation
56*56*759966 4--557*7464+*9559484-7*849859+66
Quality
Consensus
TTWLQYITFFR-KKPRQKVN-L-ILRKHFNDD-VLAKML-EASK
Pin_PITG_15142T0/1-490 431 VSSTKTMATDLLDAFTLRWMYNRESPTNVYQWLRVEGTSKDNAI 474
Pso_286249/1-489 427 VPSTKAYASDLLDDLLIRWMAGETDPRKVYAWLRVEGSAEYDVA 470
Pca_129113/1-478 418 VPATKEEATKLLTSLVGRWPKSRVHPDNVYKWLRVQGREKTDGF 461
Prm_Prm_85377/1-491 429 VPGVRTIASDLQYNLLVRWMKTKTDPARVLKWLRVEGTAENDAA 472
Conservation

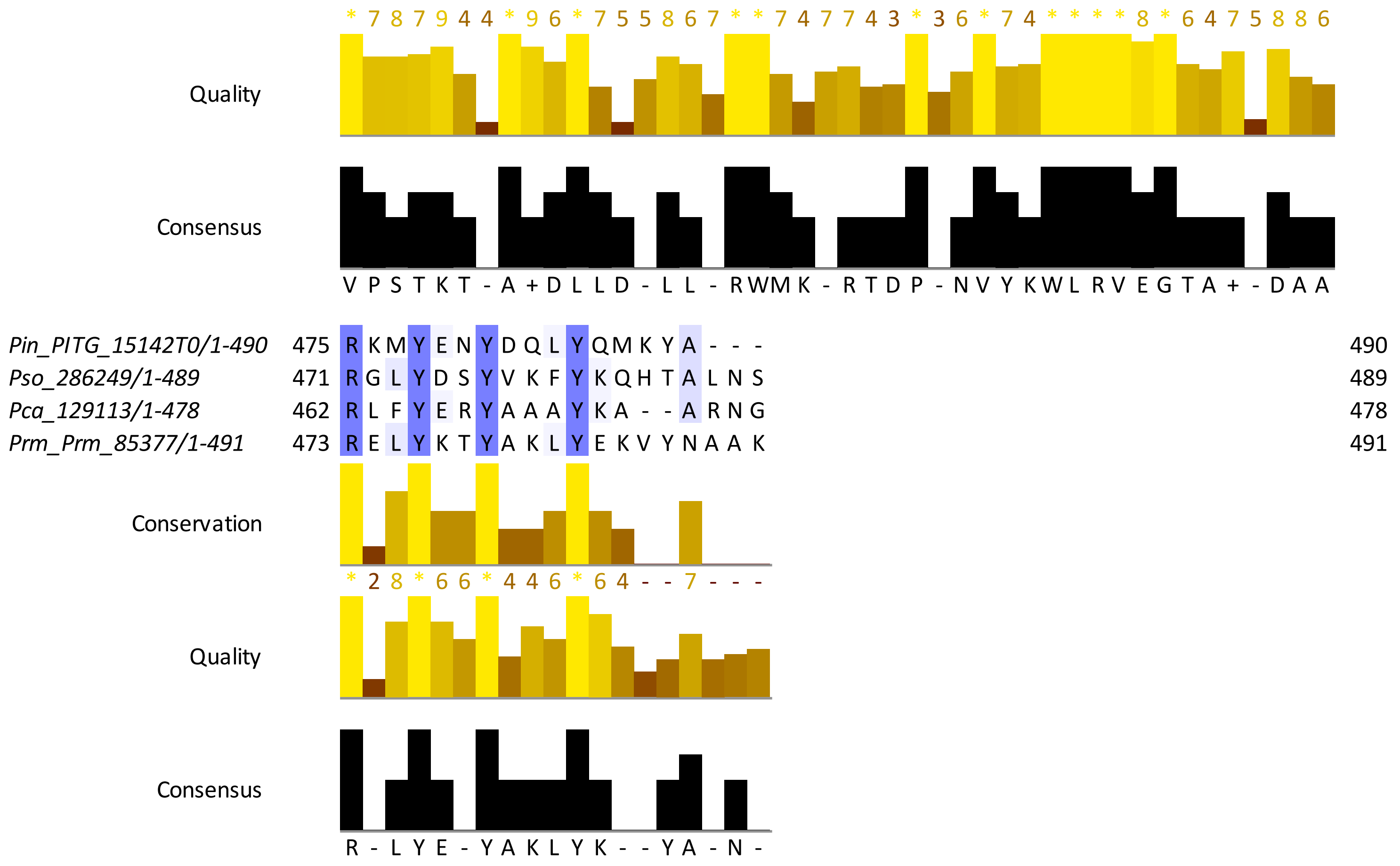


Supplementary Figure 23: Multiple sequence alignments of the 28 orthologs of RxLR-like effectors.

# Supplementary Figure 24

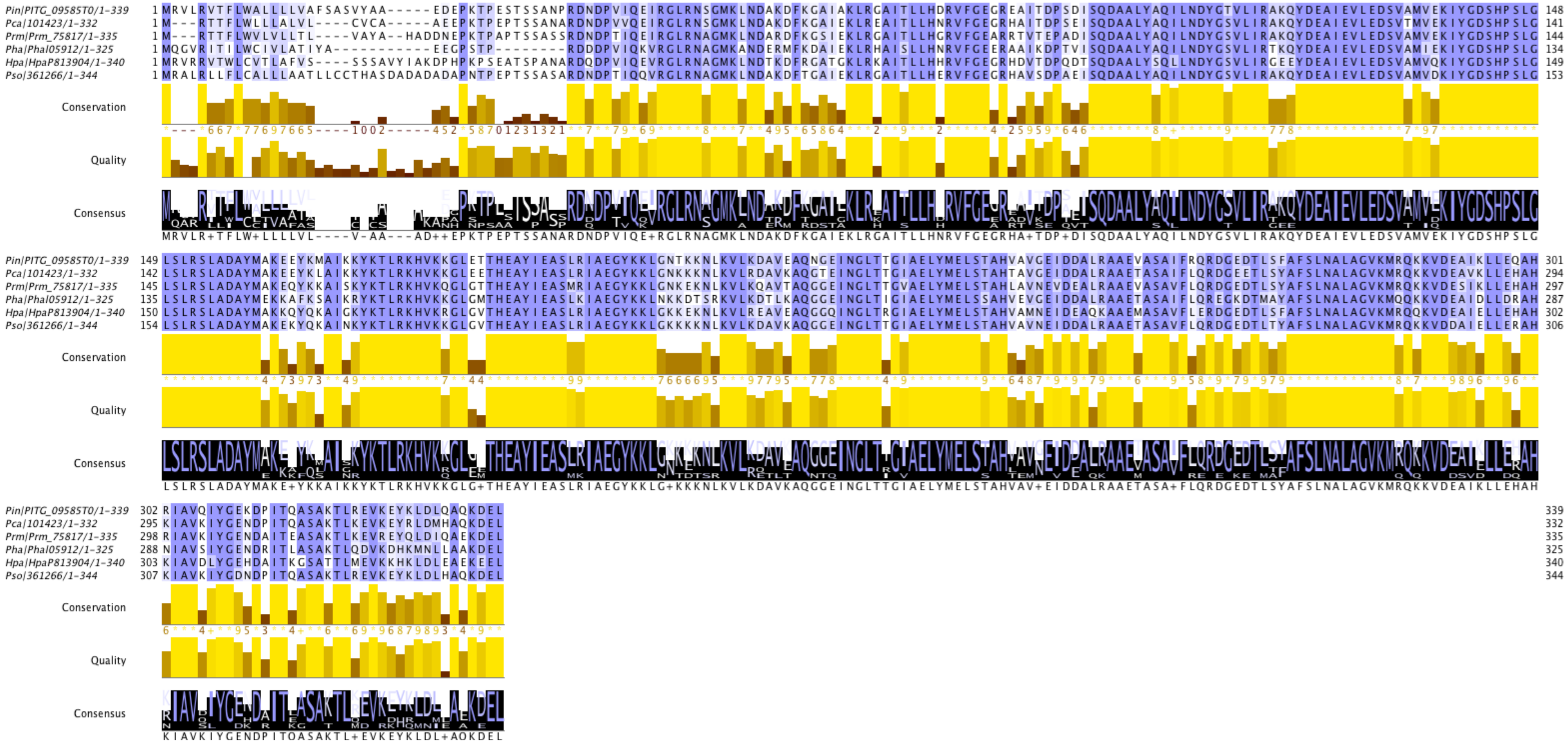

**Supplementary Figure 24:** Multiple sequence alignment of the RxLR-like proteins of Ortholog 1.

# Supplementary Figure 25

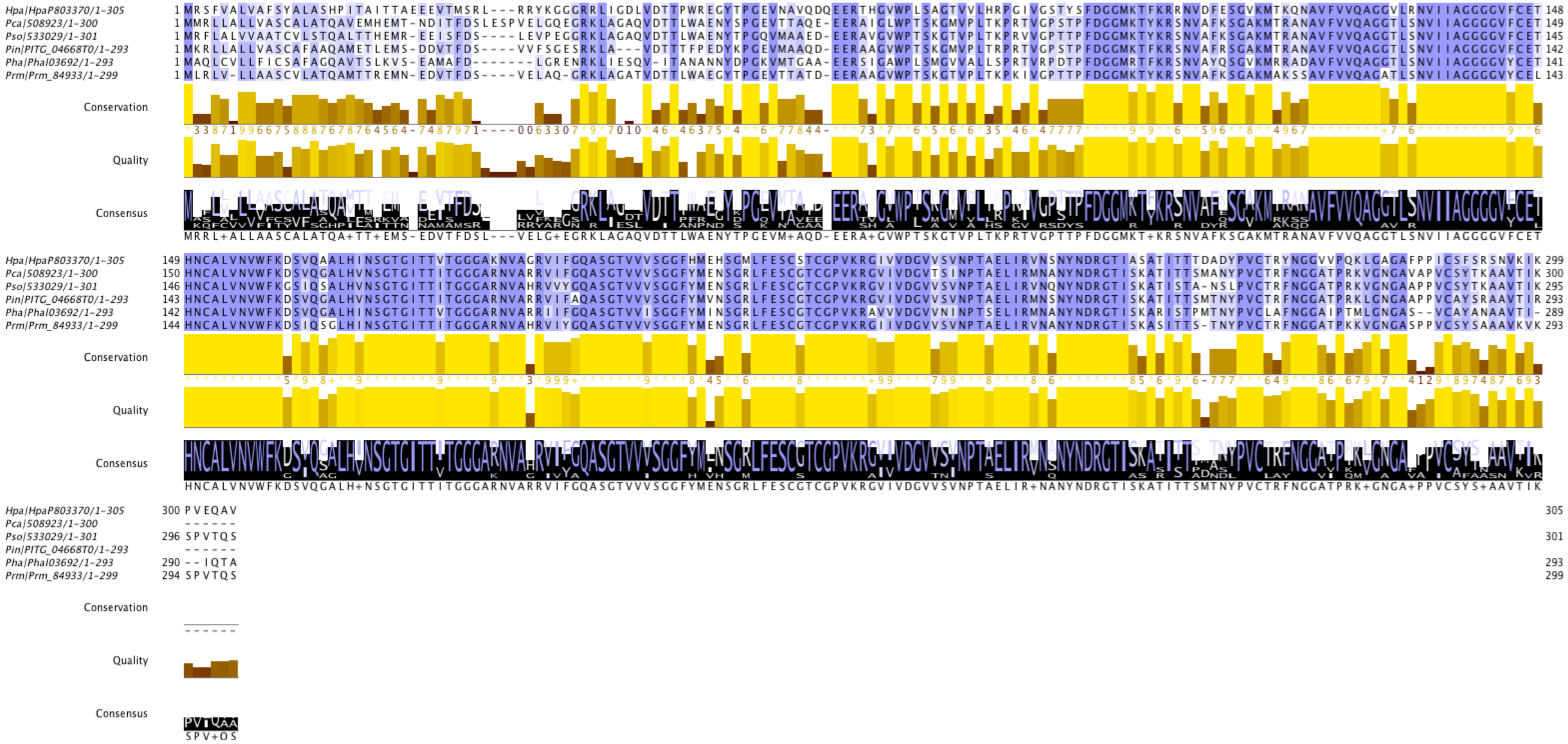


**Supplementary Figure 25:** Multiple sequence alignment of the RxLR-like proteins of Ortholog 2.

# Supplementary Figure 26

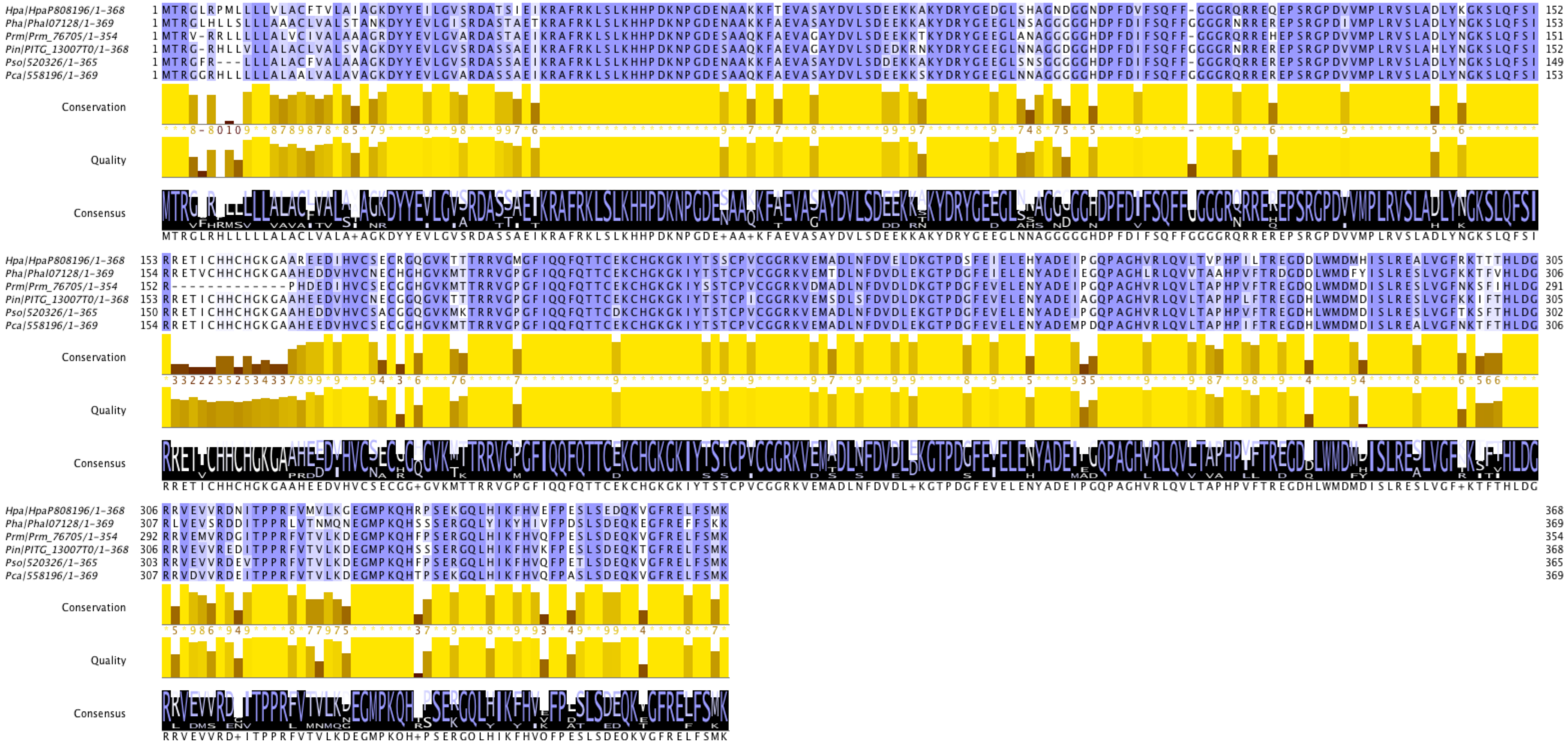

**Supplementary Figure 26:** Multiple sequence alignment of the RxLR-like proteins of Ortholog 3.

## Supplementary Figure 27

**A** RxLR effectors up-regulated during infection
**B** RxLR effectors down-regulated during infection
**C** CRN effectors up-regulated during infection
**D** CRN effectors down-regulated during infection
**Threshold : 2**

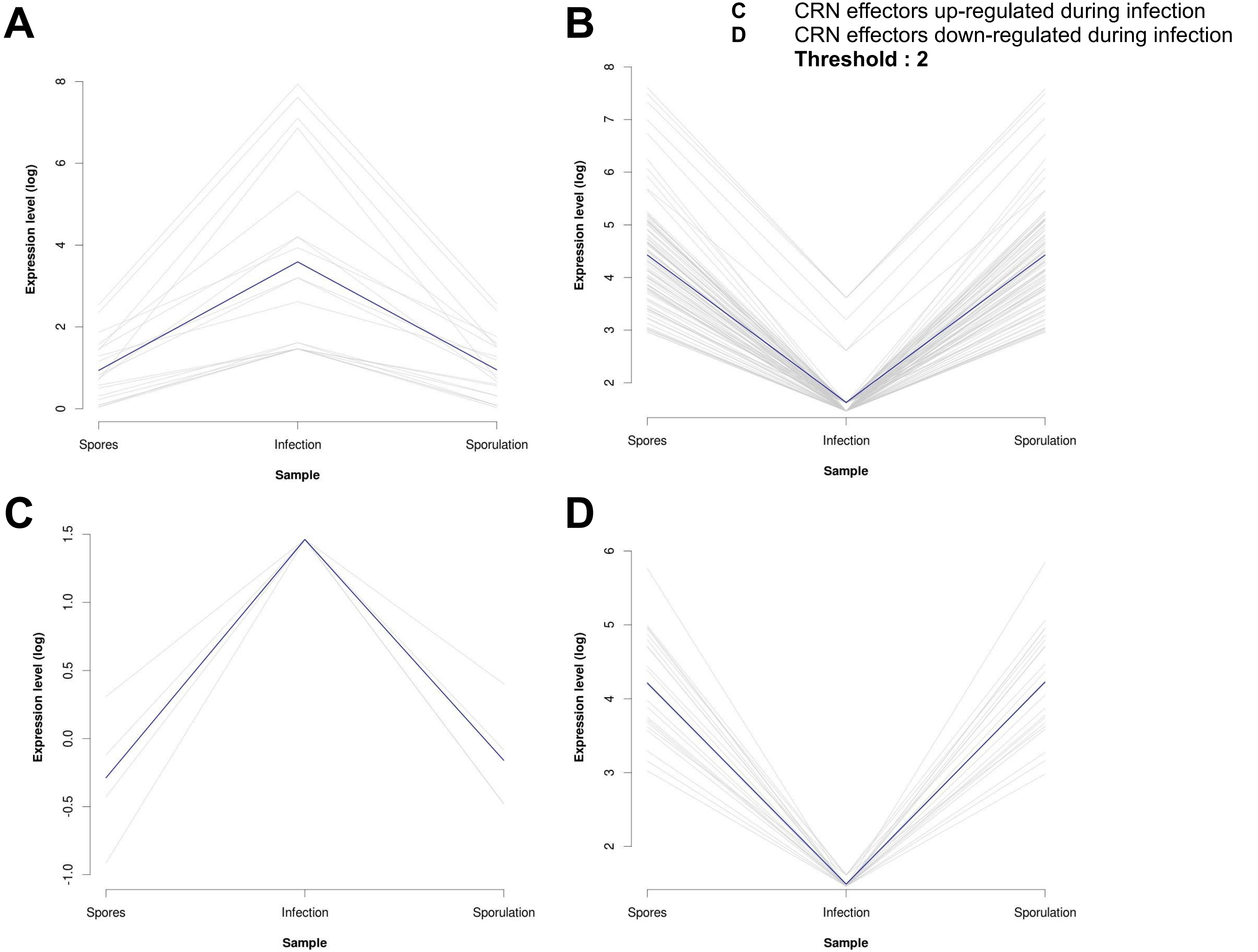


**Supplementary figure 27:** Expression profiling of the candidate RxLR and CRNs encoding genes. Expression values have been estimated in terms of log2.

## Supplementary Figure 28

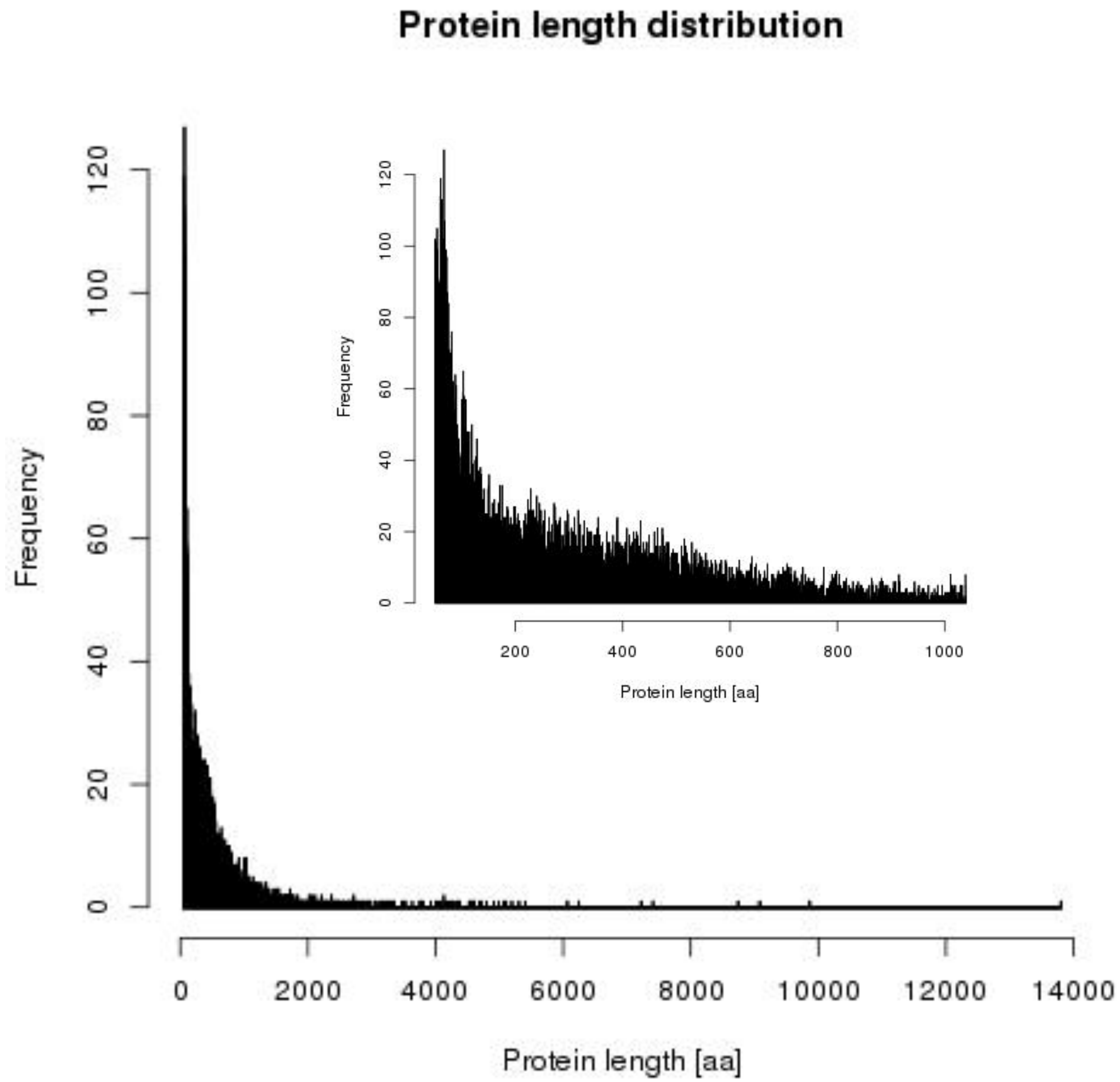


**Supplementary Figure 28:** Protein length distribution.
