# Supplementary material for: Genome analyses of the sunflower pathogen Plasmopara halstedii provide insights into effector evolution in downy mildews and Phytophthora
Source: BMC Genomics. 2015 Oct 5;16:741. doi: 10.1186/s12864-015-1904-7 (PMC4594904; doi:10.1186/s12864-015-1904-7)
Supplement: Additional file 4: — Statistics of repetitive motifs in Pl. halstedii genome. (DOCX 38 kb) [file 12864_2015_1904_MOESM4_ESM.docx]

Supplementary File 3. Statistics of repetitive motifs in *Pl. halstedii* genome

| Motif(-mer) | nuclear | mitochondrial |
| --- | --- | --- |
| 2 | 12805 | 16 |
| 3 | 1170 | 1 |
| 5 | 151 | 0 |
| 4 | 90 | 0 |
| 6 | 45 | 0 |
| 7 | 26 | 0 |
| 8 | 15 | 0 |
| 9 | 13 | 0 |
| 10 | 4 | 0 |
| 12 | 2 | 0 |
| other | 5 | 0 |
| Sum: | 14326 | 17 |
