# Supplementary material for: Genome analyses of the sunflower pathogen Plasmopara halstedii provide insights into effector evolution in downy mildews and Phytophthora
Source: BMC Genomics. 2015 Oct 5;16:741. doi: 10.1186/s12864-015-1904-7 (PMC4594904; doi:10.1186/s12864-015-1904-7)
Supplement: Additional file 5: — Repeat motifs and their frequencies. (DOCX 57 kb) [file 12864_2015_1904_MOESM5_ESM.docx]

Supplementary File 4. The results list grouped motif types and corresponding occurrence in ranked order. Only motifs with occurence over 15 are provided.

| repeat | total |  | repeat | total |
| --- | --- | --- | --- | --- |
| GT/AC | 2778 |  | CAG/CTG | 43 |
| AT/AT | 2271 |  | ATT/AAT | 42 |
| TG/CA | 1945 |  | GAC/GTC | 41 |
| TA/TA | 1687 |  | TGC/GCA | 39 |
| GA/TC | 1623 |  | TAT/ATA | 34 |
| AG/CT | 1351 |  | TAA/TTA | 32 |
| GC/GC | 690 |  | GTG/CAC | 31 |
| CG/CG | 460 |  | AAC/GTT | 31 |
| TTC/GAA | 126 |  | TCG/CGA | 28 |
| AGA/TCT | 97 |  | ACC/GGT | 24 |
| AAG/CTT | 96 |  | CCA/TGG | 23 |
| TGA/TCA | 84 |  | GAG/CTC | 23 |
| AGC/GCT | 64 |  | TTCTC/GAGAA | 20 |
| ATC/GAT | 63 |  | ACG/CGT | 19 |
| TTG/CAA | 59 |  | TCTTC/GAAGA | 18 |
| TGT/ACA | 51 |  | TTTA/TAAA | 17 |
| ATG/CAT | 49 |  | AGAAG/CTTCT | 15 |
