# Supplementary material for: Genome analyses of the sunflower pathogen Plasmopara halstedii provide insights into effector evolution in downy mildews and Phytophthora
Source: BMC Genomics. 2015 Oct 5;16:741. doi: 10.1186/s12864-015-1904-7 (PMC4594904; doi:10.1186/s12864-015-1904-7)
Supplement: Additional file 10: — PMSE genes among the oomycete genomes. (DOCX 50 kb) [file 12864_2015_1904_MOESM10_ESM.docx]

| PMSE gene | *Pl. halstedii* | *Ph. infestans** | *Hy. arabidopsidis* | *Py. ultimum* | *Sa. parasitica* |
| --- | --- | --- | --- | --- | --- |
| PIS | 1 | 1 | 1 | 1 | 1 |
| PIK | 22 | 24 | 17 | 26 | 27 |
| PIPK | 16 | 16 | 15 | 16 | 16 |
| PLC | 0 | 0 | 0 | 0 | 1 |
| DGK | 1 | 1 | 1 | 1 | 2 |
| PLD | >7 | 18 | 9 | 9 | 8 |
| Total | 47 (or more) | 60 | 43 | 53 | 55 |

*due to high overlap in numbers of genes, *Ph. infestans* was taken as representative for all *Phytophthora* spp.

Phosphatidylinositol synthase (PIS); Phosphatidyl inositol kinase (PIK); Phosphatidylinositol phosphate kinase (PIPK); Diacylglycerol kinase (DGK); Phospholipase D (PLD); Phospholipase C (PLC)
