# Supplementary material for: Genome analyses of the sunflower pathogen Plasmopara halstedii provide insights into effector evolution in downy mildews and Phytophthora
Source: BMC Genomics. 2015 Oct 5;16:741. doi: 10.1186/s12864-015-1904-7 (PMC4594904; doi:10.1186/s12864-015-1904-7)
Supplement: Additional file 12: — Potential capacity of biosynthesis of plant hormones in Pl. halstedii. (DOCX 40 kb) [file 12864_2015_1904_MOESM12_ESM.docx]

**Supplementary File 11:** Potential capacity of biosynthesis of plant hormones in *Pl. halstedii* as derived from genome sequences.

| Potential production of: | salicylate | auxin | gibberellins | abscisic acid | cytokinin | brassinolide |
| --- | --- | --- | --- | --- | --- | --- |
| *Pl. halstedii* | - | - | - | - | + | + |
