# Supplementary material for: Genome analyses of the sunflower pathogen Plasmopara halstedii provide insights into effector evolution in downy mildews and Phytophthora
Source: BMC Genomics. 2015 Oct 5;16:741. doi: 10.1186/s12864-015-1904-7 (PMC4594904; doi:10.1186/s12864-015-1904-7)
Supplement: Additional file 14: — Properties of candidate NLPs of Pl. halstedii. (DOCX 72 kb) [file 12864_2015_1904_MOESM14_ESM.docx]

**Supplementary Table 13:** Properties of PhalNLP genes

|  | | |  |  |  |
| --- | --- | --- | --- | --- | --- |
| **Gene** | **Scaffold** | **length (nt)** | **length (aa)** | **SP (D-score)** | **type** |
| PHALS_06162 | 2956 | 1467 | 488 | 0,79 | 1 |
| PHALS_06084 | 292 | 783 | 260 | 0,806 | 1 |
| PHALS_05247 | 2881 | 750 | 249 | 0,87 | 1a |
| PHALS_01213 | 1343 | 756 | 251 | 0,836 | 1a |
| PHALS_13274 | 658 | 759 | 252 | 0,81 | 1a |
| PHALS_12600 | 614 | 804 | 267 | 0,434 | 1a |
| PHALS_13512 | 658 | 732 | 243 | 0,655 | 1a |
| PHALS_13513 | 658 | 723 | 240 | 0,888 | 1a |
| PHALS_14423 | 816 | 804 | 267 | 0,646 | 1a |
| PHALS_08480 | 322 | 768 | 255 | 0,381 | 1a |
| PHALS_14425 | 816 | 909 | 302 | 0,767 | 1a |
| PHALS_14424 | 816 | 891 | 296 | 0,853 | 1a |
| PHALS_08447 | 322 | 729 | 242 | 0,872 | 1a |
| PHALS_08445 | 322 | 729 | 242 | 0,804 | 1a |
| PHALS_08945 | 323 | 729 | 242 | 0,806 | 1a |
| PHALS_08947 | 323 | 729 | 242 | 0,707 | 1a |
| PHALS_02474 | 1963 | 729 | 242 | 0,677 | 1a |
| PHALS_03592 | 2386 | 729 | 242 | 0,842 | 1a |
| PHALS_05391 | 289 | 729 | 242 | 0,868 | 1a |
| PhalPseudoNLP1 | 2904 | 790 | - | 0,627 | 1a |
