# Supplementary material for: Genome analyses of the sunflower pathogen Plasmopara halstedii provide insights into effector evolution in downy mildews and Phytophthora
Source: BMC Genomics. 2015 Oct 5;16:741. doi: 10.1186/s12864-015-1904-7 (PMC4594904; doi:10.1186/s12864-015-1904-7)
Supplement: Additional file 15: — Protease inhibitors effector families predicted in the genome of Plasmopara halstedii. (DOCX 95 kb) [file 12864_2015_1904_MOESM15_ESM.docx]

**Supplementary Table 14.** Protease inhibitors effector families predicted in the genome of *Plasmopara halstedii*

| **Gene ID** | **Other gene name** | **Effector family** | **P1 active amino acid residue** | **No. of domains** | **Comments** |
| --- | --- | --- | --- | --- | --- |
| PHALS_00020 |  | Kazal-like serine protease inhibitor effector | D | 1 |  |
| PHALS_00334 |  | Kazal-like serine protease inhibitor effector | R | 1 |  |
| PHALS_00335 |  | Kazal-like serine protease inhibitor effector | V | 1 |  |
| PHALS_00336 |  | Kazal-like serine protease inhibitor effector | R | 1 |  |
| PHALS_00402 |  | Kazal-like serine protease inhibitor effector | D, A, E | 3 |  |
| PHALS_01542 |  | Kazal-like serine protease inhibitor effector | D | 1 |  |
| PHALS_02422 |  | Kazal-like serine protease inhibitor effector | Q, E | 2 |  |
| PHALS_02736 |  | Kazal-like serine protease inhibitor effector | A, D, K | 3 |  |
| PHALS_06103 |  | Kazal-like serine protease inhibitor effector | D | 1 |  |
| PHALS_06782 |  | Kazal-like serine protease inhibitor effector | D | 1 |  |
| PHALS_06850 |  | Kazal-like serine protease inhibitor effector | E | 1 |  |
| PHALS_07228 |  | Kazal-like serine protease inhibitor effector | I, A, R, D | 4 |  |
| PHALS_09920 |  | Kazal-like serine protease inhibitor effector | T, D, D | 3 |  |
| PHALS_10391 |  | Kazal-like serine protease inhibitor effector | D | 1 |  |
| PHALS_11175 |  | Kazal-like serine protease inhibitor effector | D | 1 |  |
| PHALS_11779 |  | Kazal-like serine protease inhibitor effector | D, Y | 2 |  |
| PHALS_13385 |  | Kazal-like serine protease inhibitor effector | D | 1 |  |
| PHALS_13337 |  | Kazal-like serine protease inhibitor effector | D | 1 |  |
| PHALS_01594_1 | GenBank accession CB174657 [[7](#_ENREF_7)] | Kazal-like serine protease inhibitor effector | E | 1 | Not predicted in the gene models |
| PHALS_10211 |  | Cystatin-like cysteine protease inhibitor effector | NA | 1 |  |
| PHALS_00779 | GenBank accession CB174713 [[7](#_ENREF_7)] | Cystatin-like cysteine protease inhibitor effector | NA | 1 |  |
| PHALS_14945_pse |  | Cystatin-like cysteine protease inhibitor effector | NA | 1 |  |
| PHALS_00322_1 |  | Cystatin-like cysteine protease inhibitor effector | NA | 1 | Not predicted in the gene models |
